# Pain and Suicide Behavior in Cancer Patients: Implications for Personalized Treatment—A Systematic Review

**DOI:** 10.3390/jpm16010042

**Published:** 2026-01-08

**Authors:** Alessio Simonetti, Davide Tripaldella, Francesca Bardi, Mario Pinto, Romina Caso, Gianmarco Stella, Leonardo Monacelli, Giovanni Camardese, Antonio Maria D’Onofrio, Silvia Montanari, Delfina Janiri, Gabriele Sani

**Affiliations:** 1 Department of Neurosciences, Head-Neck and Chest, Section of Psychiatry, Fondazione Policlinico Universitario Agostino Gemelli IRCCS, 00168 Rome, Italy; g.camardese@unilink.it (G.C.);; 2 Menninger Department of Psychiatry and Behavioral Sciences, Baylor College of Medicine, Houston, TX 77030, USA; 3 Department of Neurosciences, Section of Psychiatry, Università Cattolica del Sacro Cuore, 00168 Rome, Italymario.pinto@guest.policlinicogemelli.it (M.P.);; 4 Department of Life Sciences, Health and Health Professions, Link Campus University, 00165 Rome, Italy

**Keywords:** suicide, depression, oncology, psychiatry

## Abstract

**Objective:** Pain is among the most common and debilitating symptoms experienced by oncology patients and has been associated with adverse mental health outcomes, including depression and suicide. Nevertheless, the relationship between pain and suicide in oncology populations remains insufficiently characterized. A clearer understanding of this interplay is essential to guide personalized approaches aimed at reducing cancer-related burden and improving quality of life. **Methods:** We searched PubMed and PsycInfo without imposing limits regarding publication date using pain* AND (suicid* OR “self-harm” OR “self-injurious behavior” OR “self-inflicted injury” or “self-killing”) AND (cancer* OR oncolog* OR tumor* OR neoplasm* OR metasta*). A total of 832 articles were identified, and 15 of them were included in our review. **Results:** Inadequately managed pain in cancer patients is associated with a significantly elevated risk of suicidal ideation. This association is further exacerbated in individuals presenting with depressive symptoms, advanced-stage disease, or limited access to timely psychological support. These factors may interact synergistically, intensifying the emotional and cognitive burden of pain, thereby increasing vulnerability in cancer patients. **Conclusions**: Cancer-related pain should be conceptualized as a highly variable indicator of psychological vulnerability. Factors influencing this variability include cancer type and severity, as well as the presence of past psychopathology. These findings support the need for a personalized medicine approach, whereby pain management and psychosocial interventions are tailored to patient-specific factors such as disease stage, psychological comorbidity, and access to supportive care.

## 1. Introduction

Cancer represents one of the major global health challenges and is currently the second leading cause of death worldwide. Recent epidemiological data indicate that, in 2022, newly diagnosed cases exceeded 20 million, while the disease accounted for an estimated 9.7 million deaths [1]. A central aspect of the cancer experience is the substantial psychological, social, and physical distress that can affect nearly every area of a patient’s life [2,3]. A cancer diagnosis is often highly stressful, partly because of the persistent stigma surrounding the disease and partly owing to the uncertainty associated with prognosis [4]. In addition, physical symptoms related to tumor progression or treatment, such as pain, fatigue, and functional limitations, can profoundly compromise quality of life [5,6]. Among the most relevant sources of distress, and one of the strongest determinants of patient well-being, is pain. Cancer-related pain, arising either from the disease itself or from treatment side effects, is a complex and highly variable condition [7]. Its intensity, characteristics, and responsiveness to analgesic interventions differ substantially among patients [8,9,10]. Although pain is typically a protective mechanism, persistent pain becomes pathological, severely affecting quality of life and mental health [11,12]. Cancer-related pain deserves particular attention because of both its severity and its emotional meaning. Often interpreted by patients as an indicator of disease progression, it is strongly associated with psychological distress, including anxiety, depression, and a perceived loss of control [7,13]. Cancer-related pain fits within the broader framework of chronic pain and shares several pathophysiological mechanisms and psychological effects observed across other persistent pain conditions. Examining the features of chronic pain in different clinical contexts is therefore essential for clarifying processes and consequences that also manifest in oncology, highlighting the impact that persistent pain can exert on patients’ emotional and functional well-being. Among the conditions that present the most severe forms of chronic pain are neuropathic pain, rheumatologic disorders, and inflammatory bowel diseases. Neuropathic pain is one of the most clinically significant forms, often moderate to severe in intensity and difficult to manage [14]. Persistent neuropathic symptoms are strongly linked to anxiety, depression, and social impairment, as shown in numerous clinical and epidemiological studies [15,16]. Similar patterns emerge in rheumatologic conditions such as rheumatoid arthritis and psoriatic arthritis, where chronic and sometimes intense pain, especially during flare, is closely associated with anxiety and depressive symptoms, which in turn amplify pain perception and negatively influence disease course [17,18,19]. In inflammatory bowel diseases (IBD), including Crohn’s disease and ulcerative colitis, chronic abdominal pain is a major and highly disabling symptom [20]. This pain frequently co-occurs with fatigue, anxiety, and reduced psychological well-being, contributing substantially to impaired quality of life. Beyond its profound impact on quality of life, severe chronic pain is also a major risk factor for suicidal ideation and behavior. Epidemiological evidence indicates that individuals with chronic pain have a two- to three-fold higher risk of suicide compared with the general population [21,22], partly mediated by persistent suffering, affective comorbidities, and hopelessness [23].

Given this complexity, the adoption of a personalized medicine framework is essential for accurately characterizing pain across clinical conditions and for guiding the selection of appropriately targeted, evidence-based management strategies. Precision pain medicine has been proposed to empirically match individual patients with optimal treatments, including treatment combinations, to maximize efficacy [24]. A systematic, multidimensional assessment of physical, psychological, and social determinants of quality of life is thus indispensable for designing individualized interventions that reflect each patient’s unique clinical profile and needs. Personalized pain assessment grounded in the biopsychosocial model has been shown to capture the biological, psychological, and social dimensions of pain, enabling such tailored interventions [25]. Such an approach allows for the early identification of individuals at increased risk of distress, depression, or suicidal ideation and supports the implementation of tailored interventions. In chronic pain, personalized and process-based psychological treatments have been advocated to account for individual variability and to improve long-term outcomes [26]. Although cancer, like other chronic conditions, is associated with a markedly elevated risk of suicide, the literature still provides an incomplete characterization of suicidality in oncology. Prevalence estimates, clinical manifestations, and the underlying psychological and biological mechanisms remain insufficiently defined, limiting the development of targeted prevention strategies. Several systematic reviews and meta-analyses have examined suicidal ideation, suicide attempts, and mortality in cancer patients. Chen et al. [27] highlighted pain as one of the significant risk factors for suicidal ideation in oncology populations. Reviews on the global prevalence of suicide in cancer patients have estimated the burden across broad tumor types [28]. An overview published in 2025 aggregated data from multiple reviews, offering a synthesis of prevalence, incidence, and risk across different tumor sites [29]. However, none systematically examines the specific role of cancer-related pain or how pain intensity affects suicidal risk. The present review aims to fill this gap by focusing on the interplay between pain severity and suicidal outcomes, while integrating underrepresented clinical and mechanistic perspectives. Understanding this relationship is essential for advancing personalized medicine and for informing prevention and support strategies tailored to the specific needs of patients.

## 2. Materials and Methods

### 2.1. Search Strategy

A search strategy on PubMed and PsycInfo databases was performed of all literature published before 1 October 2025. The search was conducted by two researchers independently using the following search terms: (a) for PubMed pain* AND (suicid* OR “self-harm” OR “self-injurious behavior” OR “self-inflicted injury” or “self-killing”) AND (cancer* OR oncolog* OR tumor* OR neoplasm* OR metasta*); (b) for PsycInfo databases pain* AND (suicid* OR “self-harm” OR “self-injurious behavior” OR “self-inflicted injury” or “self-killing”) AND (cancer* OR oncolog* OR tumor* OR neoplasm* OR metasta*).

Abstracts were screened according to inclusion and exclusion criteria. Papers included in this review met the following criteria: (I) original research articles (no review or meta-analyses); (II) conducted on adult populations (≥18 years of age); (III) studies conducted on populations with a confirmed cancer diagnosis; (IV) Studies in which physical pain and suicidal risk or suicidal ideation were assessed using validated psychometric instruments or structured/semi-structured clinical interviews. Exclusion criteria were (I) reviews and meta-analyses (labeled as “Review”); (II) editorials, comments or conferences, (labeled as “Editorial, Comments, Conference”); (III) studies not involving human subjects, including in vitro or animal research (“In vitro” or “Animal”); (IV) Case report or case series (“Case”); (V) studies involving participants under 18 years of age (“Lumping”); (VI) studies that did not report specific findings on the association between pain and suicide risk in patients with a cancer diagnosis (“Unfocused”); (VII) studies whose primary focus was inconsistent with the scope of this review, such as those exploring suicide risk not related to pain in patients with cancer (“Unfocused”); (VIII) studies providing only narrative description of pain without a scientific methodology supporting their assessment or conclusions (“Unfocused”); and (IX) duplicate publications or multiple reports of the same study population without additional original data (“Duplicate”). All the studies that met the inclusion criteria were categorized as “Included”.

### 2.2. Study Selection

Titles and abstracts were independently screened by two reviewers based on predefined inclusion and exclusion criteria. Full-text articles were retrieved when eligibility could not be determined from the abstract alone. Discrepancies were resolved through structured consensus discussions among all authors, using Delphi rounds until unanimity was achieved. Two rounds were sufficient to reach a complete agreement for paper inclusion or exclusion. Details of the selection process are provided in the Supplementary Materials (Tables S1 and S2).

### 2.3. Data Extraction

Data were independently extracted by two reviewers using a standardized extraction form. Extracted variables included study design, sample characteristics, cancer type, pain assessment methods, measures of suicidal ideation or risk, and main findings on the association between pain and suicidality. Any discrepancies were resolved through discussion and consensus between reviewers.

### 2.4. Reporting Standards and Data Synthesis

**Figure 1:**
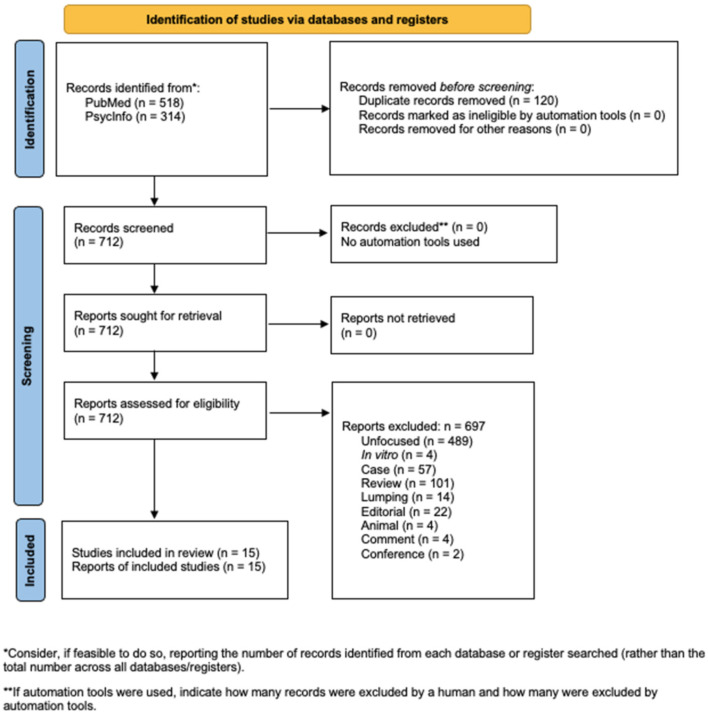
Flowchart of the systematic literature search according to PRISMA guidelines.

In developing this systematic review, we adopted the Preferred Reporting Items for Systematic reviews and Meta-Analyses statement [30]. The PRISMA flow diagram is presented in the main manuscript (Figure 1), while the PRISMA checklist is reported in the Supplementary Material (Table S3). No meta-analyses, heterogeneity analyses, or sensitivity analyses were conducted, as this review is purely descriptive.

### 2.5. Risk of Bias Assessment

**Figure 2:**
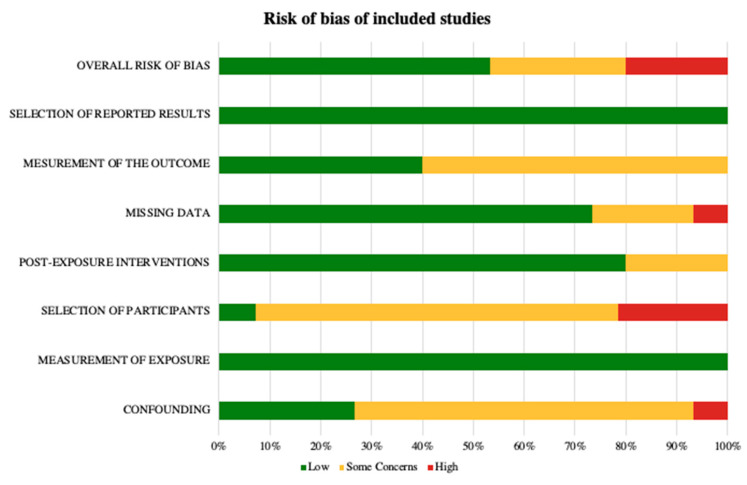
Risk of Bias according to the Cochrane ROBINS-E tool.

To assess Risk of Bias (RoB), i.e., the risk of an overestimation or an underestimation of an outcome or effect due to flaws in design, conduct, analyses, and reporting, we followed the Cochrane RoB method as described in the Cochrane Handbook [31,32]. We used RoB2, a revised Cochrane risk-of-bias tool for randomized trials, to evaluate longitudinal studies [33]. We registered our review on OSF, ID 10.17605/OSF.IO/GUCX7. No amendments were made to the protocol after registration. Overall judgments and reviewer comments for each rated study are provided in the Supplementary Materials. Risk of bias assessments are reported in the Supplementary Material (Table S4), with a summary of RoB assessments presented in the main article (Figure 2).

## 3. Results

### 3.1. Search Results

The systematic search yielded a total of 832 records, comprising 518 from PubMed and 314 from PsycInfo. After removing 120 duplicates, 712 records were considered for full-text assessment. Following the application of exclusion criteria, including animal, case report or case control, comment, conference, duplicate, editorial, in vitro, lumping, review, and unfocused, 697 records were excluded (Figure 1). As a result, 15 studies met the inclusion criteria and were retained for qualitative synthesis (Table 1 and Table 2).

### 3.2. Study Characteristics

Fourteen studies were cross-sectional, and one was longitudinal. The included studies investigated cancer patients across different tumor sites and disease stages.

### 3.3. Assessment of Pain and Suicidality

Physical pain was evaluated using a range of standardized psychometric instruments, both self-reported and clinician-administered, including McGill Pain Questionnaire (MGPQ), the Visual Analogue Scale (VAS), the Verbal Rating Scale (VRS), the European Organization for Research and Treatment of Cancer Quality of Life Questionnaire—Core 30 (EORTC QLQ-C30), EuroQol-5 Dimensions (EQ-5D), the Pain Numeric Rating Scale (NRS), the Self-Reported Single-Item Rating (SR-SIR), Self-Reported Frequency-Based Pain Assessment (SRFPA), and the Bodily Pain subscale (PB subscale), and complemented by structured or semi-structured clinical interviews: Structured Clinical Interview for DSM (SCID) and Socio-Demographic and Clinical Questionnaire (SCDQ). Suicidal risk and ideation were similarly assessed through standardized measures, encompassing self-reported and clinician-administered scales such as Patient Health Questionnaire-9 (PHQ-9), the Hamilton Depression Rating Scale (HAMD), the Suicidal Ideation Scale (SIS), the Brief Symptom Inventory-18 (BSI-18), the Columbia-Suicide Severity Rating Scale (C-SSRS), the Multi-item Closed-Response Questionnaire for the Assessment of Suicidal Ideation (MICRQ-SI), the Mini International Neuropsychiatric Interview (MINI), the Mini International Neuropsychiatric Interview—Suicide Risk (MINI-SR), the Plutchik Suicide Risk Scale (PSRS), the Suicidal Self-Directed Violence (SSDV) and the Verbal Rating Scale (VRS), and complemented by the Socio-Demographic and Clinical Questionnaire.

### 3.4. Association Between Pain and Suicidal Ideation

In all the studies examined, the presence of pain in cancer patients, regardless of tumor site or disease stage, was associated with suicidal ideation. Across nearly all the studies reviewed, a significant association emerged between pain in cancer patients and the risk of suicidal ideation or suicidal behavior, regardless of cancer site or disease stage [34,35,37]. Only one study reported suicidal ideation in 25.1% of patients without finding an association with pain [44]. Several studies also demonstrated a clear graded association relationship between pain intensity and the likelihood of suicidal ideation. In a sample of 256 patients with lung cancer, 8.2% reported suicidal ideation: 59.3% experienced mild pain, and 22.7% moderate-to-severe pain. Patients with suicidal ideation had significantly higher pain scores than those without ideation [40,47]. Similarly, another study reported that suicidal ideation occurred in 5.8% of patients without pain, 12.1% of those with mild pain, and 21.4% of those with severe pain, showing a nearly fourfold increase in ideation with severe pain [42]. Consistent evidence also emerges from studies examining overall suicide risk, which likewise report a stronger association between suicidality and higher levels of pain severity [22,43]. Multiple investigations have shown that more intense pain is linked to an increased likelihood of suicidal ideation and behavior. For instance, a study reported that individuals experiencing severe or extreme pain had markedly higher odds of both suicidal thoughts and suicide attempts [49]. In clinical pain populations, greater pain intensity has been associated with a higher risk of suicide-related outcomes [50].

### 3.5. Clinical and Psychological Factors

In most studies, the association between pain and suicidal ideation remained significant even after adjusting for demographic variables, psychological symptoms such as anxiety and depression, and medical comorbidities, suggesting an independent contribution of pain [42,45]. On the other hand, some authors have shown that the association between pain and suicidal ideation may be partly mediated by other clinical factors. In the study by Tuan et al. [47] on 256 patients with lung cancer, the association between pain and suicidal ideation was significant in univariate analyses but weakened in multivariate models, where depression emerged as a stronger mediating factor, although pain still contributed to suicidal risk. A similar pattern was observed in another investigation of 89 lung cancer patients, where 50% reported pain and 15% endorsed pain-related suicidal ideation; however, this association did not correlate with functional impairment, suggesting that additional psychological or clinical variables may play a central role in shaping suicidal vulnerability [35,51].

### 3.6. Results by Cancer Type

When examining cancer site-specific samples, notable differences emerged. In colorectal cancer, pain was strongly associated with suicidal risk, with up to 65% of patients experiencing pain classified as high-risk [38]. Prostate cancer studies similarly showed a clinically relevant association: among 693 patients, 12.4% reported suicidal ideation, which was significantly associated with pain and remained independent of demographic, clinical, and treatment-related variables [42].

### 3.7. Results of Risk of Bias

The risk of bias assessment of the included studies showed that most studies had “some concerns” in at least one domain, particularly regarding participant selection and confounding. Only a few studies were rated as “high risk” in specific domains [34,35,40]. More recent study [46] was rated as low risk across all domains, probably indicating an improvement in methodological quality. Overall, the available evidence is considered of moderate methodological quality, with heterogeneity in risk of bias reflecting differences in study design, assessment methods, and reporting practices.

## 4. Discussion

### 4.1. Pain Severity and Suicidality in Cancer Patients

Pain consistently emerges as one of the strongest correlates of suicidal ideation and suicide risk in patients with cancer. Studies of adult cancer populations indicate that about 61% of patients report experiencing some level of pain: 32% reporting mild, 18% moderate, and 11% severe or extreme pain. Suicidal ideation is relatively uncommon among patients without pain (around 0.6%), but its prevalence rises progressively with pain severity, reaching nearly 15% among those reporting severe or extreme pain [50]. Similarly, Recklitis et al. [39] found that cancer survivors with severe pain had substantially higher rates of suicidal ideation compared with those reporting no or mild pain, with up to a four-fold increase in prevalence. The association between pain and suicidal ideation often remains significant even after adjusting for depression, anxiety, and key socio-demographic or clinical variables, suggesting that higher pain intensity may directly increase the likelihood of suicidal thoughts in patients with cancer.

### 4.2. General Pathways Linking Pain and Suicidality

Several mechanisms may explain the relationship between pain and suicidality. Persistent and severe pain may become psychologically intolerable when symptom intensity exceeds the individual’s coping resources. This process, described in the chronic non-cancer pain literature, has been linked to suicidal ideation [52,53]. As pain intensifies, the imbalance between perceived demands and available coping capacity grows, increasing emotional strain and thereby heightening suicidal thoughts in a dose-dependent manner. Cancer-related pain often leads to functional decline, including reduced autonomy, mobility, and engagement in valued activities, which can foster hopelessness, a well-established predictor of suicidal behavior [23,54]. Thus, greater pain severity can lead to more profound functional impairment, which in turn amplifies suicide risk. This interplay may contribute to a dose–response pattern in which greater pain severity leads to reduced functioning and elevated suicide risk, although dedicated studies are needed to confirm this in oncology populations.

At the neurobiological level, persistent pain engages brain circuits, including the anterior insula, anterior cingulate cortex, salience network, and limbic regions, that subserve threat appraisal and subjective suffering [55,56]. Increasing activation of these systems is associated with heightened negative affect and aversive motivation, providing another pathway through which pain intensity may elevate suicidal vulnerability [57]. Tumors may release mediators such as nerve growth factor, bradykinin, and endothelin that sensitize peripheral nociceptors and induce neurochemical changes in the spinal cord, enhancing hypersensitivity and spontaneous activity [58,59]. In parallel, neuroinflammatory processes involving microglial and astrocytic activation and pro-inflammatory cytokines contribute to central sensitization and development of chronic pain [60,61]. These mechanisms also interact with dysregulation of the hypothalamic–pituitary–adrenal axis, a feature shared by individuals with chronic pain and those at elevated suicide risk [62,63]. Because HPA dysregulation intensifies under sustained nociceptive and inflammatory stress, this provides an additional mechanism by which increasing pain severity may progressively elevate suicidal vulnerability.

### 4.3. Cancer-Specific Pathways and Clinical Modifiers

Chemotherapy-induced peripheral neuropathy (CIPN) represents an additional contributor to the pain–suicide relationship. As one of the most persistent and disabling cancer-related pain syndromes—characterized by A-delta and C fiber dysfunction and symptoms such as burning, allodynia, and electric-shock sensations—CIPN may persist in 30–40% of patients long after treatment completion [64]. It is consistently associated with psychological distress, depression, and catastrophizing [65,66], all of which increase vulnerability to suicidal thoughts. Although direct evidence linking CIPN to suicidal ideation is limited, its chronic neuropathic nature likely contributes to risk, particularly in colorectal and gastrointestinal cancers treated with oxaliplatin-based regimens.

Such processes may therefore heighten affective and motivational vulnerability, helping to explain why pain can be independently associated with suicidality [67].

From a cognitive–behavioral perspective, pain catastrophizing has emerged as a salient mediator in cancer populations. Catastrophic thinking amplifies perceived threat and undermines perceived control, predicting suicidal ideation even after adjusting for depressive symptoms [68,69]. Nevertheless, the independent role of pain is not universally supported; one included study [44] found no association between pain and suicidal ideation, likely reflecting methodological differences, cohort characteristics, or unmeasured psychosocial protective factors [70].

### 4.4. Variability Across Cancer Types

Across cancer types, the relationship between pain and suicidality shows substantial heterogeneity. Colorectal cancer shows some of the highest rates, with suicidal ideation reported in up to 65% of patients experiencing pain [38], whereas prostate cancer exhibits more moderate levels, at around 12% [42]. In lung cancer, ideation rates vary between 8% and 15%, a variability that may reflect differences in sample characteristics and clinical severity [35,40,47]. The degree of pain reported by patients also differs markedly across cancer types. In colorectal cancer, one potential mechanism underlying tumor-related pain is perineural invasion (PNI), defined as the infiltration of cancer cells into or around nerve fibers. PNI is well documented and is consistently associated with more aggressive disease and poorer prognosis. In addition, PNI has been proposed as a contributor to cancer-related pain. However, direct clinical evidence linking PNI presence with greater pain intensity in colorectal cancer patients is limited, and further research is needed [71]. At the molecular level, colorectal cancer cells and other elements of the tumor microenvironment can release neurotrophic factors such as nerve growth factor and brain-derived neurotrophic factor [72]. These factors may interact with specific receptors on nearby nerve fibers, potentially promoting nerve growth and increasing the sensitivity of sensory neurons. Although this mechanism is biologically plausible and supported by experimental studies, direct clinical evidence linking it to increased pain in colorectal cancer patients remains limited [73,74]. In metastatic prostate cancer, patients frequently experience intense pain due to the high prevalence of bone metastases, particularly in the spine, pelvis, and femur. Bone metastases are generally more painful than metastases in other sites, such as the liver or lungs. Pain is driven by tumor-induced bone remodeling, local inflammation, and activation of nociceptive fibers in the periosteum, and often significantly impacts patients’ quality of life [75,76]. These findings highlight the need to consider each patient’s individual characteristics when assessing suicidal risk. Pain intensity and its psychological impact can vary greatly across cancer types and patient profiles.

Some of the included studies focused on a specific type of cancer, providing data only for that tumor. Other studies included patients with multiple cancer types but did not stratify pain or psychological outcomes by tumor type, preventing comparisons across subgroups. Similarly, the potential impact of cancer treatments on pain or psychological outcomes was not clearly investigated in most studies, which limits the interpretation of the reported associations. Consequently, our synthesis reflects findings across oncology populations in general, and caution is warranted when generalizing to specific tumor types or treatment settings. Future research should aim to investigate pain and suicidality stratified by tumor type and explicitly assess the influence of specific treatments to better inform personalized interventions.

### 4.5. Implication for Personalized Medicine

Given the heterogeneity of cancer-related pain and its strong association with suicidal risk, precision pain medicine provides a framework to identify patients at the highest risk and optimize individualized interventions. Tailoring pain management strategies to each patient’s unique biological, psychological, and functional profile allows clinicians to anticipate and address psychological distress, potentially reducing the risk of suicidal ideation. Cancer patients differ widely in how they perceive and respond to pain. Stratifying patients based on clinical, demographic, and psychological characteristics may help clinicians recognize those likely to experience severe or persistent pain and consequently an elevated risk of suicidal thoughts.

Genetic polymorphisms in genes such as CYP2D6, OPRM1, and COMT influence opioid metabolism, receptor sensitivity, and pain perception. These variants have demonstrated clinical relevance in cancer pain management [77]. Identifying patients with variants associated with increased pain sensitivity allows clinicians to anticipate psychological distress and implement timely interventions to mitigate suicidal risk. Quantitative sensory testing (QST) further refines patient stratification. Martland et al. [78] reviewed evidence showing that many cancer patients exhibit abnormal thermal, mechanical, or punctate sensitivity, indicative of the presence of central sensitization. Integrating these functional data with genetic and clinical information allows clinicians to define more precise pain phenotypes. This combined approach allows for the identification of patients at risk for severe pain and increased likelihood of suicidal ideation. Predictive approaches that consider patient-specific characteristics, including genetic profiles and sensory biomarkers, are being explored to identify individuals at higher risk and guide future personalized interventions [79,80].

Beyond classical manifestations of suicidal ideation and attempts, some cancer patients may engage in maladaptive coping strategies or refuse medical treatments, behaviors that are not always captured by standard questionnaires but may indicate significant psychological distress. Observational studies suggest that approximately 22–23% of cancer patients employ maladaptive coping, such as hopelessness or anxious preoccupation, which is associated with higher psychological distress and may reflect underlying suicidal vulnerability [81,82]. Although relatively uncommon, treatment refusal is documented in clinical cohorts: about 1.7% of patients with head and neck cancers declined potentially curative treatment, and among patients with small cell lung cancer, refusal rates for chemoradiotherapy or chemotherapy were 1.3% and 4.7%, respectively [83,84].

While these behaviors are not standard measures of suicidality, they may signal severe psychological distress and a potentially underestimated suicide risk. From a precision medicine perspective, incorporating observable behavioral indicators alongside genetic and sensory data can enhance suicide risk stratification and guide targeted, individualized interventions. Systematic assessment of these behaviors, together with traditional measures of ideation and attempts, allows clinicians to identify high-risk patients and tailor psychological support and pain management strategies in a personalized manner.

In this way, precision pain medicine may provide a comprehensive and clinically meaningful framework to not only optimize pain management but also reduce associated psychological distress and vulnerability to suicidal ideation in patients with cancer-related pain.

## 5. Limitations

Several limitations must be acknowledged. First, the heterogeneity across the included studies, particularly regarding sample size, assessment tools, and cancer stage at the time of evaluation, limits the generalizability of prevalence estimates and of the associations between disease stage, pain, and suicidal ideation or risk. Only one study employed a longitudinal design, restricting the ability to understand how the relationship between pain and suicidal ideation evolves over the course of the illness. The limited number of cancer site-specific studies further complicates the interpretation of the relationship between pain and suicidal ideation, as many investigations include patients with multiple tumor types. In addition, staging and pain intensity are not consistently differentiated or reported. Lastly, the methodological quality of the included studies indicates that several were rated as having “some concerns” regarding potential sources of bias, particularly in outcome measurement and participant selection. One additional limitation is the absence of a meta-analysis, which might have provided more robust quantitative estimates. However, given the heterogeneity of studies in terms of cancer types, outcomes, and assessment tools, we opted for a qualitative synthesis to provide a clear and meaningful interpretation of the available evidence.

## 6. Conclusions

The evidence reviewed clearly demonstrates that cancer pain is a principal driver of suicidal ideation, with risk increasing as pain severity intensifies and as neuropathic or central sensitization mechanisms become more prominent. These findings highlight the need for diagnostic and therapeutic approaches capable of capturing the biological and psychological heterogeneity that characterizes cancer pain. Within this framework, personalized medicine—integrating genetic, sensory, and psychological profiles—emerges as a robust, biologically grounded strategy for strengthening clinical decision-making. By enabling more accurate pain phenotyping and more targeted interventions, personalized approaches have the potential not only to improve pain control but also to reduce key determinants of suicidal ideation. At the same time, the current evidence base remains constrained by the limited number of tumor-specific studies and by inconsistencies in how pain severity and mechanisms are characterized across investigations. Addressing these gaps should be a priority for future research, particularly through cancer site-specific cohorts and more rigorous assessment of pain phenotypes, in order to clarify how tumor characteristics shape both pain trajectories and suicide risk. A more complete implementation of advanced, personalized strategies—supported by precise and tumor-specific evidence—could represent a decisive step toward a more effective model of care that is genuinely oriented toward preventing suicide risk in patients with cancer.

## Figures and Tables

**Table 1:** Summary of clinical characteristics of selected studies.

Study	Design	Sample Characteristics	Tumor Type	Metastasis/Stage	Type of Therapy	Pain Assessment	Suicidal Ideation and Risk Assessment
Ciaramella A et al., 2001 [34]	CS	$100\;\mathrm{pts}\;(50♀,\;50♂,\;\bar{x}$ age = 64 yr)	GI (47%), GU (37%), LC (16%)	NA	NA	MGPQ, VAS	HAMD
Akechi T et al., 2002 [35]	Long (6 mo)	$89\;\mathrm{pts}\;(25♀,\;64♂,\;\bar{x}$ age = 61 ± 9 yr)	LC	IIIa = 1; IIIb = 45; IV = 43	NA	VRS	HAMD
Latha KS et al., 2005 [36]	CS	$54\;\mathrm{pts}\;(27♀,\;27♂,\;\bar{x}$ age = 48.2 ± 12.0 yr)	GI (44.4%), GU (33.3%), LC (9.3%), BC (3.7%), HM (3.7%), other (5.6%)	NA	NA	VRS	SIS
Walker J et al., 2008 [37]	CS	$2924\;\mathrm{pts}\;(1874♀,\;1050♂,\;\bar{x}$ age = 60.3 ± 13.9 yr)	BC (32%), CRC (15.7%), OC (10.4%), OGYN (10.7%), TC (8.4%), PCa (12.4%), MISC (10.4%)	NA	HT, CHT, RT	EORTC, QLQ-C30	PHQ-9
Nuhu FT et al., 2009 [38]	CS	$210\;\mathrm{pts}\;(147♀,\;63♂,\;\bar{x}$ age = 53.3 ± 3.7 yr)	BC (32.4%), CC (28.1%), CRC (19%), PCa (20.5%)	NA	NA	SCDQ, SCID	SCDQ
Recklitis CJ et al., 2010 [39]	CS	9126 pts (47.3% ♀, 52.7% ♂)	Leuk, HL, CNS, Bone, SAR, NHL, WT, NB	NA	Sx, CHT, RT	SR-SIR	BSI-18
Maneeton B et al., 2012 [40]	CS	108 pts, (61♀, 47♂)	HNC 20 (18.5%), BC 5 (4.6%), Resp 4 (3.7%), GI 23 (21.3%), GU 6 (5.6%), GYN 27 (25.0%), MSK 8 (7.4%), Heme 15 (13.9%)	I 13 (12.0%), II 16 (14.8%), III 29 (26.9%), IV 39 (36.1%), Leuk 11 (10.2%)	Sx, CHT, RT	VAS	MINI
Han DH et al., 2018 [41]	CS	490 pts	NA	NA	NA	EQ-5D	VRS
Recklitis CJ et al., 2014 [42]	CS	$693\;\mathrm{pts}\;(\bar{x}$ age = 67.1 yr)	PCa	NA	HT, RT, CHT, Sx	SRFPA	MICRQ-SI
Park SA et al., 2016 [43]	CS	$457\;\mathrm{pts}\;(116♂,\;341♀;\;\bar{x}$ age = 52.5 ± 9.3 yr)	BC (31.7%), UC (24.1%), GI (22.3%), LC (21.9%).	NA	NA	VRS	MINI-SR
Johnson CC et al., 2020 [44]	CS	$175\;\mathrm{pts}\;(175♂,\;0♀;\;\bar{x}$ age = 61.8 yr)	BC (10.9%), HNC (18.3%), NHL/HL (12.0%), LC (11.4%), PCa (18.9%), Other (28.5%)	NA	NA	NRS	PHQ-9
Zhang Y et al., 2020 [45]	CS	$603\;\mathrm{pts}\;(60♂,\;543♀;\;\bar{x}$ age = 47.7 ± 11.5 yr)	BC (78.4%), LC (10.8%), CRC (8.1%), NPC (2.7%)	I (7.1%), II (43.8%), III (40.3%), IV (8.8%)	CHT (86.1%), RT (87.4%), Sx (92.9%)	MGPQ-VAS	PHQ-9
Nugent SM et al., 2021 [46]	CS	$7803\;\mathrm{pts}\;(7685♂,\;118♀;\;\bar{x}$ age = 65 ± 10.7 yr)	NPC (44.0%), LxCA (30.3%), LOCC (23.8%), NCPS(1.9%)	I (25.8%), II (12.1%), III (15.2%), IVA/IVB: (46.9%)	Sx (37.2%), RT (66.2%), CHT (42.1%), ITH (3.5%)	NRS	SSDV
Tuan NV et al., 2024 [47]	CS	256 pts (♂ 76.6%; ♀ 23.4%)	LC	NA	Sx, CHT, RT	VAS	C-SSRS
Espuig A et al., 2024 [48]	CS	$71\;\mathrm{pts}\;(♂\;76\%,\;24\%\;♀,\;\bar{x}$ age = 65.18 ± 12.02 yr)	CRC	NA	NA	PB subscale	PSRS

Abbreviations: ♂, males; ♀, females; $\bar{x}$, mean; pts, patients. CS, cross-sectional; Long, longitudinal; mo, month(s); yr, year(s); FU, follow-up; BL, baseline; TMD, temporomandibular disorders. Assessment: BP subscale, Bodily Pain subscale; BSI-18, Brief Symptom Inventory-18; C-SSRS, Columbia-Suicide Severity Rating Scale; EORTC QLQ-C30, European Organization for Research and Treatment of Cancer Quality of Life Questionnaire—Core 30; EQ-5D, Health-related Quality of Life; HAMD, Hamilton Depression Rating Scale; MICRQ-SI, Multi-Item Closed-Response Questionnaire for the Assessment of Suicidal Ideation; MINI, Mini International Neuropsychiatric Interview; MINI-SR, Mini International Neuropsychiatric Interview—Suicide Risk; MGPQ, McGill Pain Questionnaire; NRS, Pain Numeric Rating Scale; PHQ-9, Patient Health Questionnaire-9; PSRS, Plutchik Suicide Risk Scale; SCDQ, Socio-Demographic and Clinical Questionnaire; SCID, Structured Clinical Interview for DSM; SIS, Suicidal Ideation Scale; SRFPA, Self-Reported Frequency-Based Pain Assessment; SR-SIR, Self-Reported Single-Item Rating; SSDV, Suicidal Self-Directed Violence; VAS, Visual Analogue Scale; VRS, Verbal Rating Scale. Cancer: BC, breast cancer; Bone, bone tumor; CC, cervical cancer; CNS, central nervous system tumor; CRC, colorectal cancer; GI, gastrointestinal cancer; GU, genitourinary cancer; GYN, gynecologic; Heme, hematological; HL, Hodgkin lymphoma; HM, hematologic malignancy; HNC, head–neck cancer; LC, lung cancer; Leuk, leukemia; LOCC, lip and oral cavity cancer; LxCA, laryngeal cancer; MISC, miscellaneous; MSK, musculoskeletal; NB, neuroblastoma; NCPS, nasal cavity and paranasal sinuses; NHL, non-Hodgkin lymphoma; NPC, nasopharyngeal carcinoma; OC, ovarian cancer; OGYN, other gynecologic; PCa, prostate cancer; Resp, respiratory; SAR, sarcoma; TC, testicular cancer; UC, uterus cancer; WT, Wilms tumor. Therapies: CHT, chemotherapy; HT, hormone therapy; ITH, immunotherapy; RT, radiotherapy; Sx, surgery; PB, Bodily Pain subscale.

**Table 2:** Summary of aims and findings of selected studies.

Study	Aim of the Study	Results of the Study
Ciaramella A et al., 2001 [34]	The aim of the study was to compare the prevalence of current major depressive episodes in cancer outpatients using two different assessment methods—SCID and the Endicott criteria—and to examine how demographic and clinical factors such as cancer site, presence of pain, and ongoing treatments might affect the likelihood of being diagnosed with major depression.	37% of the patients reported pain, which was significantly more intense among those with depression. Although no patients had attempted suicide, approximately 10% had developed suicidal plans, which were significantly associated with current depression, pain, and metastatic disease. These findings underscore the critical importance of comprehensive assessment of pain, depressive symptomatology, and suicide risk in oncology settings.
Akechi T et al., 2002 [35];	The study examines the prevalence of suicidal ideation and its predictive factors in patients with unresectable non-small cell lung carcinoma, focusing on pain, depression, and loss of autonomy to inform risk identification and preventive interventions.	Approximately 50% of patients reported the presence of pain at the time of cancer diagnosis, whereas 15% developed suicidal ideation within six months (8% reported that “life was not worth living,” 2% expressed a desire for death, and 5% reported more structured suicidal thoughts). No suicide attempts were recorded. Univariate analysis showed that baseline pain levels were significantly higher among patients who subsequently developed suicidal ideation (2.1 ± 0.6 vs. 1.6 ± 0.6). The multivariate logistic regression confirmed that baseline pain was an independent predictive factor for suicidal ideation (β = 1.31, OR = 3.72, 95% CI: 1.12–14.69), whereas changes in pain intensity over the following six months were not significantly associated. The authors concluded that pain experienced immediately after diagnosis, even when mild to moderate, may have a persistent psychological impact, contributing to the emergence of suicidal ideation. Thus, it is not the severity of pain but its presence and persistence over time that may undermine the patient’s emotional resilience and increase vulnerability to suicidal thoughts.
Latha KS et al., 2005 [36]	The study investigates the prevalence and characteristics of suicidal behavior among terminally ill cancer patients in India, examining associated factors such as pain, family support, depression, and other psychosocial variables, in order to identify patients at risk and provide guidance for more appropriate treatment.	In this study, 51.8% of terminal cancer patients reported experiencing pain, often of a distressing nature. Mean scores of depression, hopelessness, and suicidal ideation varied significantly with pain severity, reaching their highest levels in patients whose pain was poorly controlled. In this study, suicidal ideation was relatively uncommon: 43 patients (79.7%) denied having any suicidal thoughts, while 10 patients (18.5%) reported moderate-to-severe ideation. Among these, 4 patients (40%) experienced recurrent thoughts, whereas the remainder reported only fleeting thoughts. These results underscore the critical importance of effective pain management in patients with terminal cancer.
Walker J et al., 2008 [37]	The study aims to determine the prevalence of thoughts of being better off dead or self-harm among cancer outpatients and to identify associated demographic and clinical factors. Using Item 9 of the Patient Health Questionnaire-9 (PHQ-9), it examines the frequency of these thoughts and their relationship with pain severity, emotional distress, and other clinical variables in patients attending the Edinburgh Cancer Centre.	Univariate analysis showed that suicidal ideation was more likely in patients who were unmarried, had active disease, clinically significant emotional distress, or substantial pain, with the latter two being the main determinants. In multivariate analysis, emotional distress and pain remained strongly associated with suicidal thoughts, whereas age showed a weaker association. An interaction was also found between pain and emotional distress: in patients with emotional distress, pain further increased the likelihood of suicidal ideation, while in those without distress, the effect of pain was even greater, particularly among older patients. In summary, about 8% of cancer outpatients report suicidal or self-harm thoughts, with pain and emotional distress as the key associated factors.
Nuhu FT et al., 2009 [38]	The study aims to evaluate the prevalence of pain in cancer patients and to investigate its impact on their psychological well-being and physical functioning.	Pain was more common in patients with colon/rectal cancer (90%) and in those with advanced-stage disease (81.7%) compared to patients in early (70.1%) or intermediate stages (59%). No significant associations were observed between pain and age, sex, or duration of illness. Suicidal ideation was present in 65.2% of patients with pain, compared to 36.4% of those without pain.
Recklitis CJ et al., 2010 [39]	This study assesses the prevalence of suicidal ideation (SI) in adult survivors of childhood cancer compared with sibling controls and examines whether survivors’ physical health and functioning are associated with SI, independent of demographics, treatment variables, and depression.	The data demonstrate a strong, graded association between cancer-related pain and suicidal ideation. Prevalence increased in parallel with pain intensity: 5.8% in survivors without pain, 12.1% with mild pain, 14.4% with moderate pain, and 21.4% among those experiencing severe or excruciating pain. Even mild pain more than doubled the risk (OR = 2.3), while severe pain increased it nearly four-fold (OR = 4.5). This dose–response pattern remained significant after adjusting for age, sex, and depression, with all pain categories independently predicting suicidal ideation in the multivariate model (OR 1.7–2.0). These findings confirm that cancer-related pain is a strong and autonomous contributor to suicide risk.
Maneeton B et al., 2012 [40]	To investigate the prevalence of depression among Thai cancer patients and identify the associated contributing factors.	The study highlighted a significant correlation between pain and the risk of suicidal ideation in Thai oncology patients. Mean pain scores were significantly higher in patients with depression compared to those without depression (4.4 ± 3.2 vs. 2.1 ± 2.4). This greater intensity of pain was also reflected in suicidal risk: depressed patients had a mean suicidality score of 5.5 ± 9.5, whereas non-depressed patients scored 1.4 ± 4.7. The Mann-Whitney test confirmed that these differences were statistically significant, indicating that pain is associated with an increased risk of suicidal ideation. Furthermore, logistic regression analysis showed that pain scores serve as an independent predictor of suicidal risk, with the likelihood of suicidality rising proportionally with increases in perceived pain. In other words, each increment in pain significantly increases the probability that an oncology patient will experience suicidal thoughts.
Han DH et al., 2018 [41]	To examine, in a representative sample of South Koreans, the prevalence and interrelationships among general pain, TMD, and suicidal ideation, and whether these associations vary based on individuals’ cancer history.	In this study, 2.2% of the participants had a history of cancer. The analysis showed that cancer patients have a significantly higher risk of suicidal ideation compared to individuals without cancer, especially when chronic pain and TMD are present. Generalized pain, measured using the EQ-PD , was also associated with an increased risk of suicidal ideation. These findings suggest that pain and TMD are significant risk factors for suicidal ideation in cancer patients, highlighting the importance of careful assessment and clinical management of these symptoms to help prevent potential suicidal behavior.
Recklitis CJ et al., 2014 [42]	The study examines the relationship between suicidal symptoms and long-term health outcomes in prostate cancer survivors, focusing on whether impaired physical functioning and treatment-related late effects are associated with an increased risk of suicidal ideation, independent of self-reported depression.	Among 693 prostate cancer survivors, 12.4% reported suicidal ideation (SI): 5.8% passive SI, 5.2% active SI, and 1.4% serious SI. Serious SI was significantly more frequent than in the general male population (1.4% vs. 0.7%). SI was not associated with demographic or treatment-related variables. Disabled survivors had markedly higher odds of SI (OR = 11.5). Physical health factors were strongly linked to SI: moderate-to-severe pain was significantly more common among survivors with SI, and pain occurring ≥4 days/week was an important predictor (OR = 2.71). Notably, 46.5% of survivors with SI did not present clinically significant depressive symptoms, suggesting that pain and physical impairment contribute to suicidality independently of depression.
Park SA et al., 2016 [43]	To evaluate the existence of suicidal thoughts and level of suicide risk among patients with advanced cancer, and investigate what level of pain, performance in regard to daily life activities, and psychological state in regard to anxiety and depression affect these factors.	This study demonstrated a clear dose-response relationship between cancer-related pain and suicide risk. Mean suicidality scores increased steadily across pain categories, rising from 2.3 in patients with no pain to 4.0, 4.6, 5.6, and peaking at 9.2 in those with the highest pain level. Pain remained an independent predictor of suicide risk in the regression model (β = 0.1), even after adjusting for anxiety, depression, functional status, and socio-demographic variables.
Johnson CC et al., 2020 [44]	To identify factors associated with suicidal ideation among 175 cancer-affected veterans.	In this study, 25.1% of participants reported current suicidal ideation during the evaluation. Contrary to initial expectations, pain was not significantly associated with suicidal ideation: average pain ratings did not differ between those with and without ideation, and pain did not emerge as a predictor in the regression analyses.
Zhang Y et al., 2020 [45]	To investigate the prevalence and associates of suicidal ideation in newly diagnosed Chinese cancer patients.	Among 603 cancer patients, 15.1% reported suicidal ideation (SI). Cancer-related pain was independently associated with SI (OR = 1.828), even after adjusting for depression, anxiety, childhood adversity, and medical comorbidities. Patients with SI also reported higher average pain intensity. No associations were found with cancer type, treatment history, or socio-demographic factors. Pain emerged as a significant physical correlate of SI, although anxiety showed the strongest statistical effect.
Nugent SM et al., 2021 [46]	To examine the associations between pre-cancer mental health and pain and post-cancer receipt of mental health, substance use disorder, or palliative care services with risk of suicidal self-directed violence in cancer pts.	Among survivors of head and neck cancer, about one-third of patients (33%) reported chronic pain within the two years following diagnosis, highlighting the persistence of this symptom beyond the acute phase. Among those who later engaged in suicidal behavior, the prevalence of chronic pain was strikingly higher, reaching 70.1%. Less than 1% of patients exhibited suicidal behavior, yet most of these events were fatal, with 51 out of 72 resulting in completed suicide. Chronic pain was found to be an independent risk factor for suicidal ideation and behavior, with more than double the risk compared to those without pain. These findings indicate that oncologic pain is not only a physical symptom but also significantly contributes to psychological vulnerability.
Tuan NV et al., 2024 [47]	This study aims to assess the quality of life among lung cancer patients and examine its association with suicidal ideation in a tertiary hospital in Vietnam.	In a sample of 256 lung cancer patients, 8.2% reported suicidal ideation. Pain was highly prevalent, with 59.3% of patients reporting mild pain and 22.7% reporting moderate-to-severe pain. Patients with suicidal ideation showed significantly higher pain scores on the EORTC QLQ-C30 compared to those without suicidal ideation (35.71 vs. 23.33). Univariate regression confirmed a positive association between pain and suicidal ideation. However, in the multivariate model, adjusted for anxiety, depression, and clinical variables, the effect of pain was less significant, suggesting that psychological distress may partially mediate the relationship. Overall, the findings indicate that pain contributes to suicidal ideation in lung cancer patients, but its impact is intertwined with broader psychological and symptom-related factors.
Espuig A et al., 2024 [48]	This study aims to identify the key predictors of suicide risk among individuals diagnosed with colorectal cancer, with a specific focus on the roles of pain, illness threat perception, and emotional distress.	In patients with colorectal cancer, suicide risk was strongly linked to pain, perceived threat of illness, and emotional distress. Overall, 16.5% of patients were classified as being at high suicide risk. Pain was also associated with greater perceived illness threat and higher emotional distress, and 63.6% of the sample reported clinically significant pain impairment. Together, these results show that higher levels of pain and stronger perceptions of illness threat contribute to greater emotional distress and a higher risk of suicide in this population.

SCID, Structured Clinical Interview for DSM;.OR, Odd Ratio; CI, Confidence Interval; TMD, Temporomandibular Disorders; EQ-PD, EuroQol 5 dimensions - Pain/Discomfort; SI, Suicidal Ideation; EORTC, European Organization for Research and Treatment of Cancer; QLQ-C30, Quality of Life Questionnaire—Core 30.

## Data Availability

The original contributions presented in this study are included in the article and Supplementary Materials. Further inquiries can be directed to the corresponding author.

## Supplementary Materials

The following supporting information can be downloaded at: https://www.mdpi.com/article/10.3390/jpm16010042/s1, Table S1: Results from the PubMed database search with final decision label; Table S2: Results from the PsycInfo database search with final decision label; Table S3: PRISMA 2020 checklist; Table S4: Risk of Bias according to the Cochrane ROBINS-E tool.

## Abbreviations

The following abbreviations are used in this manuscript:

PTS	Patients
CS	Cross-Sectional
LONG	Longitudinal
MO	Month(s)
YR	Year(s)
FU	Follow-up
BL	Baseline
TMB	Temporomandibular disorders
BP SUBSCALE	Bodily Pain subscale
BSI-18	Brief Symptom Inventory-18
C-SSRS	Columbia-Suicide Severity Rating Scale
EORTC QLQ-C30	European Organization for Research and Treatment of Cancer Quality of Life Questionnaire—Core 30
EQ-5D	Health-related Quality of Life
HAMD	Hamilton Depression Rating Scale
MICRQ-SI	Multi-Item Closed-Response Questionnaire for the Assessment of Suicidal Ideation
MINI	Mini International Neuropsychiatric Interview
MINI-SR	Mini International Neuropsychiatric Interview—Suicide Risk
MGPQ	McGill Pain Questionnaire
NRS	Pain Numeric Rating Scale
PHQ-9	Patient Health Questionnaire-9; PSRS, Plutchik Suicide Risk Scale
SCDQ	Socio-Demographic and Clinical Questionnaire
SCID	Structured Clinical Interview for DSM; SIS, Suicidal Ideation Scale
SRFPA	Self-Reported Frequency-Based Pain Assessment
SR-SIR	Self-Reported Single-Item Rating
SSDV	Suicidal Self-Directed Violence
VAS	Visual Analogue Scale
VRS	Verbal Rating Scale
BC	Breast Cancer
BONE	Bone Tumor
CC	Cervical Cancer
CNS	Central Nervous system tumor
CRC	Colorectal Cancer
GI	Gastrointestinal Cancer
GU	Genitourinary Cancer
GYN	Gynecologic
HEME	Hematological
HL	Hodgkin Lymphoma
HM	Hematologic Malignancy
HNC	Head-Neck Cancer
LC	Lung Cancer
LEUK	Leukemia
LOCC	Lip and Oral Cavity Cancer
LxCA	Laryngeal Cancer
MISC	Miscellaneous
MSK	Musculoskeletal
NB	Neuroblastoma
NCPS	Nasal Cavity and Paranasal Sinuses
NHL	Non-Hodgkin Lymphoma
NPC	Nasopharyngeal Carcinoma
OC	Ovarian Cancer
OGYN	Other Gynecologic
PCa	Prostate Cancer
RESP	Respiratory
SAR	Sarcoma
TC	Testicular Cancer
UC	Uterus Cancer
WT	Wilms Tumor
CHT	Chemotherapy
HT	Hormone Therapy
ITH	Immunotherapy
RT	Radiotherapy
Sx	Surgery
IBD	Inflammatory Bowel Diseases
PRISMA	Preferred Reporting Items for Systematic Reviews and Meta-Analyses
RoB	Risk of Bias
HPS	Hypothalamic-Pituitary–Adrenal Axis
CIPN	Chemotherapy-Induced Peripheral Neuropathy
QST	Quantitative Sensory Testing
