# Supplementary material for: Pain and Suicide Behavior in Cancer Patients: Implications for Personalized Treatment—A Systematic Review
Source: J Pers Med. 2026 Jan 8;16(1):42. doi: 10.3390/jpm16010042 (PMC12842880; doi:10.3390/jpm16010042)
Supplement: Supplementary file 1 [file jpm-16-00042-s001.zip › jpm-4046039-supplementary.pdf]

Supplementary Material

Index	Page
Table S1. Results from the PubMed database search with final decision label.	1
Table S2. Results from the PsycInfo database search with final decision label.	48
Table S3. PRISMA 2020 checklist.	61
Table S4. Risk of Bias according to the Cochrane ROBINS-E tool.	62

Table S1. Results from the PubMed database search with final decision label. (pain* AND [suicid* OR "self-harm" OR "self-injurious behavior" OR "self-inflicted injury" or "self-killing"] AND [cancer* OR oncolog* OR tumor* OR neoplasm* OR metasta*]. → 518 results (Oct 1st, 2025) → 15 included.

	References	Label
1	Tabachnick, N. Theories of self-destruction. *Am J Psychoanal*. **1972**, *32*, 53-61. https://doi.org/10.1007/BF01872484	UNFOCUSED
2	Koenig, R. Dying vs. well-being. *Omega (Westport).* **1973**, *4*,181-194. https://doi.org/10.2190/E1X8-TXQL-QABW-TVH4	UNFOCUSED
3	Sweet, W.H., Wepsic, J.G. Stimulation of the posterior columns of the spinal cord for pain control: indications, technique, and results. *Clin Neurosurg*. **1974**, *21*, 278-310. https://doi.org/10.1093/neurosurgery/21.cn_suppl_1.278	UNFOCUSED
4	Kango, G. **1978**, *24*, 150-177.	CASE
5	Twycross, R.G. Debate: euthanasia--a physician's viewpoint. *J Med Ethics*. **1982**, *8*, 86-95. https://doi.org/10.1136/jme.8.2.86	UNFOCUSED
6	Higgs, R. Case conference. Cutting the thread and pulling the wool--a request for euthanasia in general practice. *J Med Ethics*. **1983**, *9*, 45-49. https://doi.org/10.1136/jme.9.1.45	CASE
7	Diekstra, R.F. The significance of Nico Speijer's suicide: how and when should suicide be prevented?. *Suicide Life Threat Behav*. **1986**, *16*, 13-15. https://doi.org/10.1111/j.1943-278x.1986.tb00716.x	UNFOCUSED
8	Siegel, K. Psychosocial aspects of rational suicide. *Am J Psychother*. **1986**, *40*, 405-418. https://doi.org/10.1176/appi.psychotherapy.1986.40.3.405	CASE
9	Smith, H.L. Dying with style. Anglican *Theol Rev.* **1988**, *70*, 327-345.	UNFOCUSED
10	Stiefel, F.; Volkenandt, M.; Breitbart, W. Suizid und Krebserkrankung [Suicide and cancer]. *Schweiz Med Wochenschr.* **1989**, *119*, 891-895.	REVIEW
11	Fitzgerald, E.F.; Weinstein, A.L.; Youngblood, L.G.; Standfast, S.J.; Melius, J.M. Health effects three years after potential exposure to the toxic contaminants of an electrical transformer fire. *Arch Environ Health.* **1989**, *44*, 214-221. https://doi.org/10.1080/00039896.1989.9935886	UNFOCUSED
12	Usui, Y.; Matsukawa, M.; Hamada, T.; et al. Corrosive gastritis mimicking linitis plastica carcinoma. *Gastroenterol Jpn.* **1989**, *24*, 398-401. https://doi.org/10.1007/BF02774346	CASE
13	Coyle, N.; Adelhardt, J.; Foley, K.M.; Portenoy, R.K. Character of terminal illness in the advanced cancer patient: pain and other symptoms during the last four weeks of life. *J Pain Symptom Manage.* **1990**, *5*, 83-93. https://doi.org/10.1016/s0885-3924(05)80021-1	UNFOCUSED

14	Massie MJ, Holland JC. Depression and the cancer patient. J Clin Psychiatry. 1990;51 Suppl:12-19.	REVIEW
15	Hoos A, Rust M. Schmerztherapie und Depression bei Karzinompatienten [Pain therapy and depression in cancer patients]. Fortschr Med. 1990;108(24):462-466.	UNFOCUSED
16	Coyle N. The last four weeks of life. Am J Nurs. 1990;90(12):75-78.	UNFOCUSED
17	Leikin S, McCormick RA. Terminal illness and suicide. Ethics Behav. 1991;1(1):63-68. doi:10.1207/s15327019eb0101_6	CASE
18	Foley KM. The relationship of pain and symptom management to patient requests for physician-assisted suicide. J Pain Symptom Manage. 1991;6(5):289-297. doi:10.1016/0885-3924(91)90052-6	REVIEW
19	Stiefel F. Leiden und Suizid bei Tumorkranken [Suffering and suicide in tumor patients]. Schweiz Rundsch Med Prax. 1993;82(9):260-262.	UNFOCUSED
20	Baile WF, DiMaggio JR, Schapira DV, Janofsky JS. The request for assistance in dying. The need for psychiatric consultation. Cancer. 1993;72(9):2786-2791. doi:10.1002/1097-0142(19931101)72:9<2786::aid-cncr2820720942>3.0.co;2-2	CASE
21	Eibach U. Suizidwünsche bei Schwerkranken [Suicide wishes in severely ill patients]. Pflege Z. 1994;47(1):23-27.	UNFOCUSED
22	Logue BJ. When hospice fails: the limits of palliative care. Omega (Westport). 1994;29(4):291-301. doi:10.2190/EG0J-T0G5-1LJ4-KTPW	UNFOCUSED
23	Horn M. Embracing the night: one woman's struggle to maintain control of her destiny to the very end. US News World Rep. 1994;116(16):40-41.	CASE
24	Bruns AD, Fishkin PA, Johnson EA, Lee YT. Munchausen's syndrome and cancer. J Surg Oncol. 1994;56(2):136-138. doi:10.1002/jso.2930560219	UNFOCUSED
25	Massie MJ, Gagnon P, Holland JC. Depression and suicide in patients with cancer. J Pain Symptom Manage. 1994;9(5):325-340. doi:10.1016/0885-3924(94)90192-9	REVIEW
26	Patterson WB, Emanuel EJ, Wolf SM. Euthanasia and the care of cancer patients. J Clin Oncol. 1994;7(12):1516-1521.	CASE
27	Bellini M, Capannini D. Cancro e suicidio: aumento del rischio dei suicidio nei pazienti neoplastici [Increased suicide risk in cancer patients]. Minerva Psichiatr. 1994;35(3):175-186.	REVIEW
28	Emanuel EJ. The history of euthanasia debates in the United States and Britain. Ann Intern Med. 1994;121(10):793-802. doi:10.7326/0003-4819-121-10-199411150-00010	UNFOCUSED
29	Pribilla O. Medical legal and ethical questions in palliative medicine and euthanasia. Forensic Sci Int. 1994;69(3):299-306. doi:10.1016/0379-0738(94)90395-6	UNFOCUSED
30	Surh YJ, Lee SS. Capsaicin, a double-edged sword: toxicity, metabolism, and chemopreventive potential. Life Sci. 1995;56(22):1845-1855. doi:10.1016/0024-3205(95)00159-4	REVIEW
31	Breitbart W, Bruera E, Chochinov H, Lynch M. Neuropsychiatric syndromes and psychological symptoms in patients with advanced cancer. J Pain Symptom Manage. 1995;10(2):131-141. doi:10.1016/0885-3924(94)00075-v	REVIEW
32	Foley KM. Misconceptions and controversies regarding the use of opioids in cancer pain. Anticancer Drugs. 1995;6 Suppl 3:4-13. doi:10.1097/00001813-199504003-00002	REVIEW
33	Ersek M, Scanlon C, Glass E, Ferrell BR, Steeves R. Priority ethical issues in oncology nursing: current approaches and future directions. Oncol Nurs Forum. 1995;22(5):803-807.	UNFOCUSED
34	Chochinov HM, Wilson KG, Enns M, et al. Desire for death in the terminally ill. Am J Psychiatry. 1995;152(8):1185-1191. doi:10.1176/ajp.152.8.1185	UNFOCUSED

35	Clinicians not providing necessary pain relief for AIDS patients. AIDS Alert. 1995;10(8):97-100.	UNFOCUSED
36	Brown M, King E, Barraclough B. Nine suicide pacts. A clinical study of a consecutive series 1974-93. Br J Psychiatry. 1995;167(4):448-451. doi:10.1192/bjp.167.4.448	CASE
37	McFadden M. Owning death. Hum Life Rev. 1995;21(4):37-45.	UNFOCUSED
38	McKhann CF. Is there a role for physician-assisted suicide in cancer? Yes. Important Adv Oncol. 1996;267-279.	REVIEW
39	Siegler M. Is there a role for physician-assisted suicide in cancer? No. Important Adv Oncol. 1996;281-291.	REVIEW
40	Admiraal PV. Euthanasia in the Netherlands. Free Inq. 1996;17(1):5-8.	UNFOCUSED
41	Breitbart W, Rosenfeld BD, Passik SD. Interest in physician-assisted suicide among ambulatory HIV-infected patients. Am J Psychiatry. 1996;153(2):238-242. doi:10.1176/ajp.153.2.238	UNFOCUSED
42	Emanuel EJ. Pain and symptom control. Patient rights and physician responsibilities. Hematol Oncol Clin North Am. 1996;10(1):41-56. doi:10.1016/s0889-8588(05)70326-9	REVIEW
43	Roth AJ, Breitbart W. Psychiatric emergencies in terminally ill cancer patients. Hematol Oncol Clin North Am. 1996;10(1):235-259. doi:10.1016/s0889-8588(05)70337-3	REVIEW
44	Butler RN, Burt R, Foley KM, Morris J, Morrison RS. A peaceful death: how to manage pain and provide quality care. A roundtable discussion: Part 2. Geriatrics. 1996;51(6):32-42.	UNFOCUSED
45	Chevlen EM. Mock medicine, mock law. First Things. 1996;64:16-18.	UNFOCUSED
46	Emanuel EJ, Fairclough DL, Daniels ER, Clarridge BR. Euthanasia and physician-assisted suicide: attitudes and experiences of oncology patients, oncologists, and the public. Lancet. 1996;347(9018):1805-1810. doi:10.1016/s0140-6736(96)91621-9	UNFOCUSED
47	Cherny NI. The problem of inadequately relieved suffering. J Soc Issues. 1996;52(2):13-30. doi:10.1111/j.1540-4560.1996.tb01565.x	UNFOCUSED
48	Colburn D. Survey reveals differences on doctor-assisted suicide: patients facing choice want relief from pain. Washington Post. 1996;.	UNFOCUSED
49	Kaiser J. Depressing questions. Science. 1996;273(5271):39.	UNFOCUSED
50	Passik SD, Breitbart WS. Depression in patients with pancreatic carcinoma. Diagnostic and treatment issues. Cancer. 1996;78(3 Suppl):615-626. doi:10.1002/(SICI)1097-0142(19960801)78:3<615::AID-CNCR42>3.0.CO;2-Z	REVIEW
51	Borneman T, Ferrell BR. Ethical issues in pain management. Clin Geriatr Med. 1996;12(3):615-628.	REVIEW
52	Lester D, Yang B. An approach for examining the rationality of suicide. Psychol Rep. 1996;79(2):405-406. doi:10.2466/pr0.1996.79.2.405	UNFOCUSED
53	van der Maas PJ, van der Wal G, Haverkate I, et al. Euthanasia, physician-assisted suicide, and other medical practices involving the end of life in the Netherlands, 1990-1995. N Engl J Med. 1996;335(22):1699-1705. doi:10.1056/NEJM199611283352227	UNFOCUSED
54	Goldstein FJ. Inadequate pain management: a suicidogen (Dr. Jack Kevorkian: friend or foe?). J Clin Pharmacol. 1997;37(1):1-3. doi:10.1177/009127009703700101	EDITORIAL
55	Rich BA. A legacy of silence: bioethics and the culture of pain. J Med Humanit. 1997;18(4):233-259. doi:10.1023/a:1025697920944	REVIEW
56	Emanuel EJ. The painful truth about euthanasia. Wall St J (East Ed). 1997;A16.	UNFOCUSED
57	Webb CM, Sheckard D, Wilder P, et al. Over the past year, how many patients have asked you to inject drugs to intentionally end their lives? Have you ever injected drugs to intentionally end a patient's life?. J Gerontol Nurs. 1997;23(3):57-59.	UNFOCUSED

58	Collins JJ, Brennan FT. Euthanasia and the potential adverse effects for Northern Territory aborigines. Lancet. 1997;349(9069):1907-1908. doi:10.1016/S0140-6736(96)12388-6	UNFOCUSED
59	Severson KT. Dying cancer patients: choices at the end of life. J Pain Symptom Manage. 1997;14(2):94-98. doi:10.1016/s0885-3924(97)00110-3	CASE
60	Sullivan M, Rapp S, Fitzgibbon D, Chapman CR. Pain and the choice to hasten death in patients with painful metastatic cancer. J Palliat Care. 1997;13(3):18-28.	UNFOCUSED
61	Voltz R, Borasio GD. Palliative therapy in the terminal stage of neurological disease. J Neurol. 1997;244 Suppl 4:S2-S10. doi:10.1007/PL00007721	REVIEW
62	Henderson JM, Ord RA. Suicide in head and neck cancer patients. J Oral Maxillofac Surg. 1997;55(11):1217-1222. doi:10.1016/s0278-2391(97)90170-1	CASE
63	Fish P. A harder better death. Health (N Y). 1997;11(8):108-114.	UNFOCUSED
64	Kumakura N. Seishin Shinkeigaku Zasshi. 1998;100(2):113-122.	CASE
65	Strang P. Cancer pain--a provoker of emotional, social and existential distress. Acta Oncol. 1998;37(7-8):641-644. doi:10.1080/028418698429973	REVIEW
66	Lossignol D. La fin de vie digne du patient cancéreux [Death with dignity in cancer patients]. Rev Med Brux. 1998;19(1):A34-A36.	UNFOCUSED
67	Passik SD, Dugan W, McDonald MV, Rosenfeld B, Theobald DE, Edgerton S. Oncologists' recognition of depression in their patients with cancer. J Clin Oncol. 1998;16(4):1594-1600. doi:10.1200/JCO.1998.16.4.1594	UNFOCUSED
68	Abramson N, Stokes J, Weinreb NJ, Clark WS. Euthanasia and doctor-assisted suicide: responses by oncologists and non-oncologists. South Med J. 1998;91(7):637-642. doi:10.1097/00007611-199807000-00006	UNFOCUSED
69	McCormack P. Quality of life and the right to die: an ethical dilemma. J Adv Nurs. 1998;28(1):63-69. doi:10.1046/j.1365-2648.1998.00762.x	CASE
70	Emanuel EJ, Daniels ER, Fairclough DL, Clarridge BR. The practice of euthanasia and physician-assisted suicide in the United States: adherence to proposed safeguards and effects on physicians. JAMA. 1998;280(6):507-513. doi:10.1001/jama.280.6.507	UNFOCUSED
71	Smith TJ, Schnipper LJ. The American Society of Clinical Oncology program to improve end-of-life care. J Palliat Med. 1998;1(3):221-230. doi:10.1089/jpm.1998.1.221	UNFOCUSED
72	O'Shea EM, Lintz KC, Penson RT, Seiden MV, Chabner BA, Lynch TJ. A staff dialogue on caring for a cancer patient who commits suicide: psychosocial issues faced by patients, their families, and caregivers. Oncologist. 1999;4(2):122-127.	CASE
73	Chin AE, Hedberg K, Higginson GK, Fleming DW. Legalized physician-assisted suicide in Oregon--the first year's experience. N Engl J Med. 1999;340(7):577-583. doi:10.1056/NEJM199902183400724	UNFOCUSED
74	Breitbart W, Rosenfeld BD. Physician-Assisted Suicide: The Influence of Psychosocial Issues. Cancer Control. 1999;6(2):146-161. doi:10.1177/107327489900600203	UNFOCUSED
75	Wolfe J, Fairclough DL, Clarridge BR, Daniels ER, Emanuel EJ. Stability of attitudes regarding physician-assisted suicide and euthanasia among oncology patients, physicians, and the general public. J Clin Oncol. 1999;17(4):1274. doi:10.1200/JCO.1999.17.4.1274	UNFOCUSED
76	Steenland K, Palu S. Cohort mortality study of 57,000 painters and other union members: a 15 year update. Occup Environ Med. 1999;56(5):315-321. doi:10.1136/oem.56.5.315	UNFOCUSED
77	Briner V. Sterbehilfe--interdisziplinär betrachtet--aus der Sicht der Spitalärztin [Interdisciplinary discussion about euthanasia--viewpoint of the clinical physicians]. Praxis (Bern 1994). 1999;88(29-30):1229-1234.	UNFOCUSED

78	Chochinov HM, Tataryn D, Clinch JJ, Dudgeon D. Will to live in the terminally ill. Lancet. 1999;354(9181):816-819. doi:10.1016/S0140-6736(99)80011-7	UNFOCUSED
79	Linder JF, Blais J, Enders SR, Melberg SE, Meyers FJ. Palliative education: a didactic and experiential approach to teaching end-of-life care. J Cancer Educ. 1999;14(3):154-160. doi:10.1080/08858199909528607	UNFOCUSED
80	Akechi T, Kugaya A, Okamura H, et al. Suicidal thoughts in cancer patients: clinical experience in psycho-oncology. Psychiatry Clin Neurosci. 1999;53(5):569-573. doi:10.1046/j.1440-1819.1999.00607.x	UNFOCUSED
81	Ahronheim JC, Davol SB. Pursuit of assisted dying: a pilot study of inquiries made to a national consumer-based organization. J Pain Symptom Manage. 1999;18(6):401-405. doi:10.1016/s0885-3924(99)00106-2	UNFOCUSED
82	Ferrell B, Virani R, Grant M, Coyne P, Uman G. Beyond the Supreme Court decision: nursing perspectives on end-of-life care. Oncol Nurs Forum. 2000;27(3):445-455.	UNFOCUSED
83	Wein S. Sedation in the imminently dying patient. Oncology (Williston Park). 2000;14(4):585-601.	REVIEW
84	Schopper D, Torres AM, Pereira J, et al. Setting health priorities in a Swiss canton: what do different methods tell us?. J Epidemiol Community Health. 2000;54(5):388-393. doi:10.1136/jech.54.5.388	UNFOCUSED
85	Rosenfeld B, Breitbart W, Galietta M, et al. The schedule of attitudes toward hastened death: Measuring desire for death in terminally ill cancer patients. Cancer. 2000;88(12):2868-2875. doi:10.1002/1097-0142(20000615)88:12<2868::aid-cncr30>3.0.co;2-k	UNFOCUSED
86	Bauduer F, Capdupuy C, Renoux M. Characteristics of deaths in a department of oncohaematology within a general hospital. A study of 81 cases. Support Care Cancer. 2000;8(4):302-306. doi:10.1007/s005209900087	UNFOCUSED
87	Schopper D, Ammon C, Ronchi A, Rougemont A. When providers and community leaders define health priorities: the results of a Delphi survey in the canton of Geneva. Soc Sci Med. 2000;51(3):335-342. doi:10.1016/s0277-9536(99)00457-8	UNFOCUSED
88	Wilson KG, Scott JF, Graham ID, et al. Attitudes of terminally ill patients toward euthanasia and physician-assisted suicide. Arch Intern Med. 2000;160(16):2454-2460. doi:10.1001/archinte.160.16.2454	UNFOCUSED
89	Lloyd-Williams M, Friedman T, Rudd N. Criterion validation of the Edinburgh postnatal depression scale as a screening tool for depression in patients with advanced metastatic cancer. J Pain Symptom Manage. 2000;20(4):259-265. doi:10.1016/s0885-3924(00)00182-2	UNFOCUSED
90	Emanuel EJ, Fairclough D, Clarridge BC, et al. Attitudes and practices of U.S. oncologists regarding euthanasia and physician-assisted suicide. Ann Intern Med. 2000;133(7):527-532. doi:10.7326/0003-4819-133-7-200010030-00011	UNFOCUSED
91	Alvarez RD, Gomez-Navarro J, Wang M, et al. Adenoviral-mediated suicide gene therapy for ovarian cancer. Mol Ther. 2000;2(5):524-530. doi:10.1006/mthe.2000.0194	IN VITRO
92	Hilden JM, Emanuel EJ, Fairclough DL, et al. Attitudes and practices among pediatric oncologists regarding end-of-life care: results of the 1998 American Society of Clinical Oncology survey. J Clin Oncol. 2001;19(1):205-212. doi:10.1200/JCO.2001.19.1.205	UNFOCUSED
93	Volker DL. Oncology nurses' experiences with requests for assisted dying from terminally ill patients with cancer. Oncol Nurs Forum. 2001;28(1):39-49.	UNFOCUSED
94	Musgrave CF, Margalith I, Goldsmidt L. Israeli oncology and nononcology nurses' attitudes toward physician-assisted dying: a comparison study. Oncol Nurs Forum. 2001;28(1):50-57.	UNFOCUSED
95	McKhann CF. A perspective on physician-assisted dying. Surg Oncol Clin N Am. 2001;10(1):221-231.	REVIEW
96	Löhr JM. Palliative Therapie des Pankreasadenokarzinoms [Palliative therapy of pancreatic adenocarcinoma]. Z Gastroenterol. 2001;39(2):181-190. doi:10.1055/s-2001-11483	REVIEW
97	Ciaramella A, Poli P. Assessment of depression among cancer patients: the role of pain, cancer type and treatment. Psychooncology. 2001;10(2):156-165. doi:10.1002/pon.505	INCLUDED

98	Kohlwes RJ, Koepsell TD, Rhodes LA, Pearlman RA. Physicians' responses to patients' requests for physician-assisted suicide. Arch Intern Med. 2001;161(5):657-663. doi:10.1001/archinte.161.5.657	UNFOCUSED
99	Filiberti A, Ripamonti C, Totis A, et al. Characteristics of terminal cancer patients who committed suicide during a home palliative care program. J Pain Symptom Manage. 2001;22(1):544-553. doi:10.1016/s0885-3924(01)00295-0	UNFOCUSED
100	Chochinov HM. Depression in cancer patients. Lancet Oncol. 2001;2(8):499-505. doi:10.1016/S1470-2045(01)00456-9	REVIEW
101	Ensink FB, Bautz MT, Hanekop GG. Optimierung der ambulanten palliativmedizinischen Betreuung terminal kranker Tumorpatienten am Beispiel SUPPORT - ethisch zu präferierende nach aktiver Sterbehilfe [Improvement of palliative outpatient treatment of terminally ill cancer patients - SUPPORT as example - The ethically preferable alternative to euthanasia]. Anasthesiol Intensivmed Notfallmed Schmerzther. 2001;36(9):530-537. doi:10.1055/s-2001-17256	UNFOCUSED
102	Martin-Du Pan R. Les bienfaits du vieillissement. II. Vieillir et mourir de nos jours: des raisons d'être optimiste? [The benefits of aging. II. Aging and dying in our time: reasons for optimism?]. Rev Med Suisse Romande. 2001;121(9):689-695.	REVIEW
103	Macfarlane GJ, McBeth J, Silman AJ. Widespread body pain and mortality: prospective population based study. BMJ. 2001;323(7314):662-665. doi:10.1136/bmj.323.7314.662	UNFOCUSED
104	Tremblay A, Breitbart W. Psychiatric dimensions of palliative care. Neurol Clin. 2001;19(4):949-967. doi:10.1016/s0733-8619(05)70055-4	REVIEW
105	Neumann JL. Ethical issues confronting oncology nurses. Nurs Clin North Am. 2001;36(4):827-viii.	REVIEW
106	Chodorowski Z, Sein Anand J. Wpływ propagowania eutanazji przez media na próby samobójcze wśród nieuleczalnie chorych [The influence of media propagation of euthanasia on suicide attempts among terminally ill patients]. Przegl Lek. 2002;59(4-5):388-389.	UNFOCUSED
107	Suarez-Almazor ME, Newman C, Hanson J, Bruera E. Attitudes of terminally ill cancer patients about euthanasia and assisted suicide: predominance of psychosocial determinants and beliefs over symptom distress and subsequent survival. J Clin Oncol. 2002;20(8):2134-2141. doi:10.1200/JCO.2002.08.025	UNFOCUSED
108	Filiberti A, Ripamonti C. Suicide and suicidal thoughts in cancer patients. Tumori. 2002;88(3):193-199. doi:10.1177/030089160208800303	REVIEW
109	Akechi T, Okamura H, Nishiwaki Y, Uchitomi Y. Predictive factors for suicidal ideation in patients with unresectable lung carcinoma. Cancer. 2002;95(5):1085-1093. doi:10.1002/cncr.10769	INCLUDED
110	Goldstein FJ. Adjuncts to opioid therapy. J Am Osteopath Assoc. 2002;102(9 Suppl 3):S15-S21.	REVIEW
111	Ryynänen OP, Myllykangas M, Viren M, Heino H. Attitudes towards euthanasia among physicians, nurses and the general public in Finland. Public Health. 2002;116(6):322-331. doi:10.1038/sj.ph.1900875	UNFOCUSED
112	Fishbain DA. Predictive factors for suicidal ideation in patients with unresectable lung carcinoma. A 6-month follow-up study. Cancer. 2003;97(12):3127-3129. doi:10.1002/cncr.11429	COMMENT
113	Caffo E, Belaise C. Psychological aspects of traumatic injury in children and adolescents. Child Adolesc Psychiatr Clin N Am. 2003;12(3):493-535. doi:10.1016/s1056-4993(03)00004-x	REVIEW
114	Valentine AD. Cancer pain and depression: management of the dual-diagnosed patient. Curr Pain Headache Rep. 2003;7(4):262-269. doi:10.1007/s11916-003-0046-9	REVIEW
115	Schotsmans PT. The ethical claim of a dying brother. Christ Bioeth. 2003;9(2-3):331-336. doi:10.1076/chbi.9.2.331.30291	UNFOCUSED
116	Smith MT, Perlis ML, Haythornthwaite JA. Suicidal ideation in outpatients with chronic musculoskeletal pain: an exploratory study of the role of sleep onset insomnia and pain intensity. Clin J Pain. 2004;20(2):111-118. doi:10.1097/00002508-200403000-00008	UNFOCUSED

117	Matulonis UA. End of life issues in older patients. Semin Oncol. 2004;31(2):274-281. doi:10.1053/j.seminoncol.2003.12.036	REVIEW
118	Grassi L, Biancosino B, Marmai L, Righi R. Effect of reboxetine on major depressive disorder in breast cancer patients: an open-label study. J Clin Psychiatry. 2004;65(4):515-520. doi:10.4088/jcp.v65n0410	UNFOCUSED
119	Bilsen J, Stichele RV, Mortier F, Bernheim J, Deliens L. The incidence and characteristics of end-of-life decisions by GPs in Belgium. Fam Pract. 2004;21(3):282-289. doi:10.1093/fampra/cmh312	UNFOCUSED
120	Arnold EM, Artin KA, Person JL, Griffith DL. Consideration of hastening death among hospice patients and their families. J Pain Symptom Manage. 2004;27(6):523-532. doi:10.1016/j.jpainsymman.2003.10.010	UNFOCUSED
121	Valente SM. End-of-life challenges: honoring autonomy. Cancer Nurs. 2004;27(4):314-319. doi:10.1097/00002820-200407000-00008	REVIEW
122	Mwanda WO, Abdallah FK, Obondo A, Musau FM. Quality of-life in male cancer patients at Kenyatta National Hospital, Nairobi. East Afr Med J. 2004;81(7):341-347. doi:10.4314/eamj.v81i7.9188	LUMPING
123	Coyle N, Sculco L. Expressed desire for hastened death in seven patients living with advanced cancer: a phenomenologic inquiry. Oncol Nurs Forum. 2004;31(4):699-709. Published 2004 Jul 13. doi:10.1188/04.ONF.699-709	UNFOCUSED
124	Sundararaghavan S, Suarez WA. Oral benadryl and central venous catheter abuse-a potentially "lethal combination". Pediatr Emerg Care. 2004;20(9):604-606. doi:10.1097/01.pec.0000139743.38968.7f	CASE
125	Mystakidou K, Rosenfeld B, Parpa E, et al. The schedule of attitudes toward hastened death: validation analysis in terminally ill cancer patients. Palliat Support Care. 2004;2(4):395-402. doi:10.1017/s1478951504040520	UNFOCUSED
126	Akizuki N, Yamawaki S, Akechi T, Nakano T, Uchitomi Y. Development of an Impact Thermometer for use in combination with the Distress Thermometer as a brief screening tool for adjustment disorders and/or major depression in cancer patients. J Pain Symptom Manage. 2005;29(1):91-99. doi:10.1016/j.jpainsymman.2004.04.016	UNFOCUSED
127	Okereke OI, Folsom DP. Medical comorbidity in geriatric psychiatry. Am J Geriatr Psychiatry. 2005;13(3):177-179. doi:10.1176/appi.ajgp.13.3.177	EDITORIAL
128	Llorente MD, Burke M, Gregory GR, et al. Prostate cancer: a significant risk factor for late-life suicide. Am J Geriatr Psychiatry. 2005;13(3):195-201. doi:10.1176/appi.ajgp.13.3.195	UNFOCUSED
129	Latha KS, Bhat SM. Suicidal behaviour among terminally ill cancer patients in India. Indian J Psychiatry. 2005;47(2):79-83. doi:10.4103/0019-5545.55950	INCLUDED
130	Schlosser W, Rau BM, Poch B, Beger HG. Surgical treatment of pancreas divisum causing chronic pancreatitis: the outcome benefits of duodenum-preserving pancreatic head resection. J Gastrointest Surg. 2005;9(5):710-715. doi:10.1016/j.gassur.2004.11.009	UNFOCUSED
131	Painter RG, Lanson NA Jr, Jin Z, Park F, Wang G. Conditional expression of a suicide gene by the telomere reverse transcriptase promoter for potential post-therapeutic deletion of tumorigenesis. Cancer Sci. 2005 Sep;96(9):607-13. doi: 10.1111/j.1349-7006.2005.00085.x. PMID: 16128746; PMCID: PMC11158881.	IN VITRO
132	Johansen S, Hølen JC, Kaasa S, Loge HJ, Materstvedt LJ. Attitudes towards, and wishes for, euthanasia in advanced cancer patients at a palliative medicine unit. Palliat Med. 2005 Sep;19(6):454-60. doi: 10.1191/0269216305pm1048oa. PMID: 16218157.	UNFOCUSED
133	Symons FJ, Danov SE. A prospective clinical analysis of pain behavior and self-injurious behavior. Pain. 2005 Oct;117(3):473-477. doi: 10.1016/j.pain.2005.07.010. PMID: 16154695.	CASE
134	Goblirsch M, Lynch C, Mathews W, Manivel JC, Mantyh PW, Clohisy DR. Radiation treatment decreases bone cancer pain through direct effect on tumor cells. Radiat Res. 2005 Oct;164(4 Pt 1):400-8. doi: 10.1667/rr3439.1. PMID: 16187742.	ANIMAL

135	Clarke DM, McLeod JE, Smith GC, Trauer T, Kissane DW. A comparison of psychosocial and physical functioning in patients with motor neurone disease and metastatic cancer. J Palliat Care. 2005 Autumn;21(3):173-9. PMID: 16334972.	UNFOCUSED
136	Chamizo-Carmona E. ¿Existe asociación entre la fibromialgia, el aumento de la comorbilidad por enfermedad neoplásica, cardiovascular e infecciones, y el de la mortalidad? [Is there an association between fibromyalgia and an increase in comorbidity: neoplastic and cardiovascular diseases, infections and mortality?]. Reumatol Clin. 2005 Dec;1(4):200-10. Spanish. doi: 10.1016/S1699-258X(05)72745-5. Epub 2008 Dec 20. PMID: 21794265.	REVIEW
137	Gibson CA, Lichtenthal W, Berg A, Breitbart W. Psychologic issues in palliative care. Anesthesiol Clin. 2006 Mar;24(1):61-80, viii. doi: 10.1016/j.atc.2005.12.001. PMID: 16487896.	REVIEW
138	Sade B, Budur K, Lee DK, Franco K, Lee JH. Major depression with psychosis after resection of a giant middle fossa hemangiopericytoma. Surg Neurol. 2006 Mar;65(3):290-2; discussion 292. doi: 10.1016/j.surneu.2005.06.041. PMID: 16488253.	CASE
139	Denny CC, Emanuel EJ. "Physician-assisted suicide among Oregon cancer patients": a fading issue. J Clin Ethics. 2006 Spring;17(1):39-42; discussion 43-5. PMID: 16689112.	COMMENT
140	Lefetz C, Reich M. La crise suicidaire en cancérologie: évaluation et prise en charge [Suicidal crisis in oncology: assessment and care]. Bull Cancer. 2006 Jul;93(7):709-13. French. PMID: 16873080.	UNFOCUSED
141	Parpa E, Mystakidou K, Tsilika E, Sakkas P, Patiraki E, Pistevou-Gombaki K, Galanos A, Vlahos L. The attitudes of Greek physicians and lay people on euthanasia and physician-assisted suicide in terminally ill cancer patients. Am J Hosp Palliat Care. 2006 Aug-Sep;23(4):297-303. doi: 10.1177/1049909106290247. PMID: 17060293.	UNFOCUSED
142	Recklitis CJ, Lockwood RA, Rothwell MA, Diller LR. Suicidal ideation and attempts in adult survivors of childhood cancer. J Clin Oncol. 2006 Aug 20;24(24):3852-7. doi: 10.1200/JCO.2006.06.5409. PMID: 16921037.	UNFOCUSED
143	Ganzini L, Beer TM, Brouns MC. Views on physician-assisted suicide among family members of Oregon cancer patients. J Pain Symptom Manage. 2006 Sep;32(3):230-6. doi: 10.1016/j.jpainsymman.2006.04.004. PMID: 16939847.	UNFOCUSED
144	Yu DS, Huang HZ, Hu XW, Liu XQ, Tang HK, Wang AX. [Radiation-inducible promoters-mediated cdglytk gene in the treatment of buccal carcinoma in golden hamster]. Zhonghua Kou Qiang Yi Xue Za Zhi. 2006 Sep;41(9):549-52. Chinese. PMID: 17129429.	ANIMAL
145	Rosenfeld B, Breitbart W, Gibson C, Kramer M, Tomarken A, Nelson C, Pessin H, Esch J, Galietta M, Garcia N, Brechtl J, Schuster M. Desire for hastened death among patients with advanced AIDS. Psychosomatics. 2006 Nov- Dec;47(6):504-12. doi: 10.1176/appi.psy.47.6.504. PMID: 17116952.	UNFOCUSED
146	Tyner TR, Parks N, Faria S, Simons M, Stapp B, Curtis B, Sian K, Yamaguchi KT. Effects of collagen nerve guide on neuroma formation and neuropathic pain in a rat model. Am J Surg. 2007 Jan;193(1):e1-6. doi: 10.1016/j.amjsurg.2006.08.026. PMID: 17188077.	ANIMAL
147	Carter GL, Clover KA, Parkinson L, Rainbird K, Kerridge I, Ravenscroft P, Cavenagh J, McPhee J. Mental health and other clinical correlates of euthanasia attitudes in an Australian outpatient cancer population. Psychooncology. 2007 Apr;16(4):295-303. doi: 10.1002/pon.1058. PMID: 16921477.	UNFOCUSED
148	Sareen J, Cox BJ, Stein MB, Afifi TO, Fleet C, Asmundson GJ. Physical and mental comorbidity, disability, and suicidal behavior associated with posttraumatic stress disorder in a large community sample. Psychosom Med. 2007 Apr;69(3):242-8. doi: 10.1097/PSY.0b013e31803146d8. Epub 2007 Mar 30. PMID: 17401056.	UNFOCUSED
149	Prime C, Abdelmoumène N, Rullon I. L'évaluation des pratiques professionnelles dans la deuxième procédure de certification des établissements de santé [Clinical practices appraisal in the second certification process of French healthcare organisations]. Nephrol Ther. 2007 Jun;3 Spec No. 2:4-9. French. PMID: 17763639.	UNFOCUSED

150	Coopman V, Cordonnier J, Pien K, Van Varenbergh D. LC-MS/MS analysis of fentanyl and norfentanyl in a fatality due to application of multiple Durogesic transdermal therapeutic systems. Forensic Sci Int. 2007 Jul 4;169(2-3):223-7. doi: 10.1016/j.forsciint.2006.03.018. Epub 2006 May 2. PMID: 16650707.	CASE
151	Sela RA. Screening for depression in palliative cancer patients attending a pain and symptom control clinic. Palliat Support Care. 2007 Sep;5(3):207-17. doi: 10.1017/s1478951507000375. PMID: 17969824.	LUMPING
152	Takayanagi K. Laughter education and the psycho-physical effects: introduction of smile-sun method. Jpn Hosp. 2007 Dec;(26):31-5. PMID: 19195158.	UNFOCUSED
153	Murphy TF. The ethics of responding to pain and suffering. Cancer Treat Res. 2008;140:117-35. doi: 10.1007/978-0-387-73639-6_8. PMID: 18283773.	REVIEW
154	Denny CC, Emanuel EJ. "Physician-assisted suicide among Oregon cancer patients": a fading issue. J Clin Ethics. 2006 Spring;17(1):39-42; discussion 43-5. PMID: 16689112.	COMMENT
155	Stenager E, Stenager E. Selvmordsadfaerd ved somatiske sygdom [Suicidal behaviour and somatic disorders]. Ugeskr Laeger. 2008 Feb 11;170(7):517-22. Danish. PMID: 18291078.	REVIEW
156	Harth W, Hillert A, Hermes B, Seikowski K, Niemeier V, Freudenmann RW. Suizidalität in der Dermatologie [Suicidal behavior in dermatology]. Hautarzt. 2008 Apr;59(4):289-96. German. doi: 10.1007/s00105-008-1509-z. PMID: 18338146.	UNFOCUSED
157	Reiter-Theil S, Graf-Baumann T, Kutzer K, Müller-Busch HC, Stutzki R, Traue HC, Willweber-Strumpf A, Zimmermann M, Zenz M. Ethik-Charta der Deutschen Gesellschaft zum Studium des Schmerzes (DGSS) [Ethic charter of the German Society for the Study of Pain (DGSS)]. Schmerz. 2008 Apr;22(2):191-206. German. doi: 10.1007/s00482-008-0648-6. PMID: 18351397.	UNFOCUSED
158	Moussas GI, Karkanias AP, Papadopoulou A. [Psychological and psychiatric problems in cancer patients in the general hospital]. Psychiatriki. 2008 Apr;19(2):124-44. Greek, Modern. PMID: 22217929.	UNFOCUSED
159	de Veer AJ, Francke AL, Poortvliet EP. Nurses' involvement in end-of-life decisions. Cancer Nurs. 2008 May-Jun;31(3):222-8. doi: 10.1097/01.NCC.0000305724.83271.f9. PMID: 18453879.	UNFOCUSED
160	Dongre A, Deshmukh P, Murali N, Garg B. Tobacco consumption among adolescents in rural Wardha: where and how tobacco control should focus its attention? Indian J Cancer. 2008 Jul-Sep;45(3):100-6. doi: 10.4103/0019-509x.44065. PMID: 19018113.	UNFOCUSED
161	Parpa E, Mystakidou K, Tsilika E, Sakkas P, Patiraki E, Pistevou-Gombaki K, Govina O, Vlahos L. Euthanasia and physician-assisted suicide in cases of terminal cancer: the opinions of physicians and nurses in Greece. Med Sci Law. 2008 Oct;48(4):333-41. doi: 10.1258/rsmmsl.48.4.333. PMID: 19051672.	UNFOCUSED
162	Walker J, Waters RA, Murray G, Swanson H, Hibberd CJ, Rush RW, Storey DJ, Strong VA, Fallon MT, Wall LR, Sharpe M. Better off dead: suicidal thoughts in cancer patients. J Clin Oncol. 2008 Oct 10;26(29):4725-30. doi: 10.1200/JCO.2007.11.8844. Epub 2008 Aug 11. PMID: 18695258.	INCLUDED
163	Nuhu FT, Odejide OA, Adebayo KO, Yusuf AJ. Psychological and physical effects of pain on cancer patients in Ibadan, Nigeria. Afr J Psychiatry (Johannesbg). 2009 Feb;12(1):64-70. doi: 10.4314/ajpsy.v12i1.30281. PMID: 19526649.	INCLUDED
164	Lydiatt WM, Moran J, Burke WJ. A review of depression in the head and neck cancer patient. Clin Adv Hematol Oncol. 2009 Jun;7(6):397-403. PMID: 19606075.	REVIEW
165	Ganzini L, Goy ER, Dobscha SK, Prigerson H. Mental health outcomes of family members of Oregonians who request physician aid in dying. J Pain Symptom Manage. 2009 Dec;38(6):807-15. doi: 10.1016/j.jpainsymman.2009.04.026. PMID: 19783401.	UNFOCUSED
166	Pousset G, Bilsen J, De Wilde J, Benoit Y, Verlooy J, Bomans A, Deliens L, Mortier F. Attitudes of adolescent cancer survivors toward end-of-life decisions for minors. Pediatrics. 2009 Dec;124(6):e1142-8. doi: 10.1542/peds.2009-0621. PMID: 19948616.	LUMPING

167	Seale C. Continuous deep sedation in medical practice: a descriptive study. J Pain Symptom Manage. 2010 Jan;39(1):44-53. doi: 10.1016/j.jpainsymman.2009.06.007. Epub 2009 Oct 24. PMID: 19854611.	UNFOCUSED
168	Dube P, Kurt K, Bair MJ, Theobald D, Williams LS. The p4 screener: evaluation of a brief measure for assessing potential suicide risk in 2 randomized effectiveness trials of primary care and oncology patients. Prim Care Companion J Clin Psychiatry. 2010;12(6):PCC.10m00978. doi: 10.4088/PCC.10m00978blu. PMID: 21494337; PMCID: PMC3067996.	UNFOCUSED
169	Recklitis CJ, Diller LR, Li X, Najita J, Robison LL, Zeltzer L. Suicide ideation in adult survivors of childhood cancer: a report from the Childhood Cancer Survivor Study. J Clin Oncol. 2010 Feb 1;28(4):655-61. doi: 10.1200/JCO.2009.22.8635. Epub 2009 Oct 19. PMID: 19841325; PMCID: PMC2816000.	INCLUDED
170	Fanger PC, Azevedo RC, Mauro ML, Lima DD, Gaspar KC, Silva VF, Nascimento WT, Botega NJ. Depressão e comportamento suicida em pacientes oncológicos hospitalizados: prevalência e fatores associados [Depression and suicidal behavior of cancer inpatients: prevalence and associated factors]. Rev Assoc Med Bras (1992). 2010 Mar-Apr;56(2):173-8. Portuguese. doi: 10.1590/s0104-42302010000200015. PMID: 20498991.	UNFOCUSED
171	Akechi T, Okamura H, Nakano T, Akizuki N, Okamura M, Shimizu K, Okuyama T, Furukawa TA, Uchitomi Y. Gender differences in factors associated with suicidal ideation in major depression among cancer patients. Psychooncology. 2010 Apr;19(4):384-9. doi: 10.1002/pon.1587. PMID: 19472294.	LUMPING
172	Hirsch G, Hérisson B, Lacour F. Réflexions sur la légalisation de l'euthanasie [Reflections on the legalization of euthanasia]. Rev Infirm. 2010 Apr;(159):32-4. French. PMID: 20432851.	UNFOCUSED
173	Fry E, Counselman FL. A right scrotal abscess and foreign body ingestion in a schizophrenic patient. J Emerg Med. 2010 Jun;38(5):587-92. doi: 10.1016/j.jemermed.2007.07.018. Epub 2007 Dec 26. PMID: 18155384.	CASE
174	Maessen M, Veldink JH, van den Berg LH, Schouten HJ, van der Wal G, Onwuteaka-Philipsen BD. Requests for euthanasia: origin of suffering in ALS, heart failure, and cancer patients. J Neurol. 2010 Jul;257(7):1192-8. doi: 10.1007/s00415-010-5474-y. Epub 2010 Feb 11. PMID: 20148336.	UNFOCUSED
175	Onwuteaka-Philipsen BD, Rurup ML, Pasman HR, van der Heide A. The last phase of life: who requests and who receives euthanasia or physician-assisted suicide? Med Care. 2010 Jul;48(7):596-603. doi: 10.1097/MLR.0b013e3181dbea75. PMID: 20508530.	UNFOCUSED
176	Scott KM, Hwang I, Chiu WT, Kessler RC, Sampson NA, Angermeyer M, Beautrais A, Borges G, Bruffaerts R, de Graaf R, Florescu S, Fukao A, Haro JM, Hu C, Kovess V, Levinson D, Posada-Villa J, Scocco P, Nock MK. Chronic physical conditions and their association with first onset of suicidal behavior in the world mental health surveys. Psychosom Med. 2010 Sep;72(7):712-9. doi: 10.1097/PSY.0b013e3181e3333d. Epub 2010 May 24. PMID: 20498290.	UNFOCUSED
177	Botega NJ, Mitsuushi GN, Azevedo RC, Lima DD, Fanger PC, Mauro ML, Gaspar KC, Silva VF. Depression, alcohol use disorders and nicotine dependence among patients at a general hospital. Braz J Psychiatry. 2010 Sep;32(3):250-6. doi: 10.1590/s1516-44462010005000016. Epub 2010 Jul 2. PMID: 20602014.	UNFOCUSED
178	Krakauer EL, Wenk R, Buitrago R, Jenkins P, Scholten W. Opioid inaccessibility and its human consequences: reports from the field. J Pain Palliat Care Pharmacother. 2010 Sep;24(3):239-43. doi: 10.3109/15360288.2010.501852. PMID: 20718644.	CASE
179	Parpa E, Mystakidou K, Tsilika E, Sakkas P, Patiraki E, Pistevou-Gombaki K, Govina O, Panagiotou I, Galanos A, Gouliamos A. Attitudes of health care professionals, relatives of advanced cancer patients and public towards euthanasia and physician assisted suicide. Health Policy. 2010 Oct;97(2-3):160-5. doi: 10.1016/j.healthpol.2010.04.008. Epub 2010 May 21. PMID: 20488575.	UNFOCUSED
180	van Tol D, Rietjens J, van der Heide A. Judgment of unbearable suffering and willingness to grant a euthanasia request by Dutch general practitioners. Health Policy. 2010 Oct;97(2-3):166-72. doi: 10.1016/j.healthpol.2010.04.007. Epub 2010 May 21. PMID: 20488576.	UNFOCUSED

181	Clin B, Ophélie F. Law of 22 April 2005 on patients' rights and the end of life in France: setting the boundaries of euthanasia, with regard to current legislation in other European countries. Med Sci Law. 2010 Oct;50(4):183-8. doi: 10.1258/msl.2010.010037. PMID: 21539283.	UNFOCUSED
182	Schmidt SC, Strauch S, Rösch T, Veltzke-Schlieker W, Jonas S, Pratschke J, Weidemann H, Neuhaus P, Schumacher G. Management of esophageal perforations. Surg Endosc. 2010 Nov;24(11):2809-13. doi: 10.1007/s00464-010-1054-6. Epub 2010 Apr 29. PMID: 20428896.	UNFOCUSED
183	Madeira N, Albuquerque E, Santos T, Mendes A, Roque M. Death ideation in cancer patients: contributing factors. J Psychosoc Oncol. 2011;29(6):636-42. doi: 10.1080/07347332.2011.615381. PMID: 22035536.	UNFOCUSED
184	Barni S, Maltoni M, Tuveri G, Pronzato P, Cortesi E, Massidda B, Colucci G, Iacono C, Lorusso V, Gridelli C, Aitini E, Simoni L, Torta R. Attitude of Italian medical oncologists toward palliative care for patients with advanced cancer: results of the SIO project. Support Care Cancer. 2011 Mar;19(3):381-9. doi: 10.1007/s00520-010-0831-z. Epub 2010 Mar 4. PMID: 20204421.	UNFOCUSED
185	Dettmore D, Gabriele LC. Don't just do something, stand there: responding to unrelieved patient suffering. J Psychosoc Nurs Ment Health Serv. 2011 Apr;49(4):34-8. doi: 10.3928/02793695-20110302-01. Epub 2011 Mar 16. PMID: 21410090.	CASE
186	Chambaere K, Bilsen J, Cohen J, Onwuteaka-Philipsen BD, Mortier F, Deliens L. Trends in medical end-of-life decision making in Flanders, Belgium 1998-2001-2007. Med Decis Making. 2011 May-Jun;31(3):500-10. doi: 10.1177/0272989X10392379. Epub 2010 Dec 29. PMID: 21191121.	UNFOCUSED
187	Yun YH, Han KH, Park S, Park BW, Cho CH, Kim S, Lee DH, Lee SN, Lee ES, Kang JH, Kim SY, Lee JL, Heo DS, Lee CG, Lim YK, Kim SY, Choi JS, Jeong HS, Chun M. Attitudes of cancer patients, family caregivers, oncologists and members of the general public toward critical interventions at the end of life of terminally ill patients. CMAJ. 2011 Jul 12;183(10):E673-9. doi: 10.1503/cmaj.110020. Epub 2011 May 30. PMID: 21624907; PMCID: PMC3134758.	UNFOCUSED
188	Monforte-Royo C, Villavicencio-Chávez C, Tomás-Sábado J, Balaguer A. The wish to hasten death: a review of clinical studies. Psychooncology. 2011 Aug;20(8):795-804. doi: 10.1002/pon.1839. Epub 2010 Sep 6. PMID: 20821377.	REVIEW
189	Zenz M, Rissing-van Saan R. Grenzen der Schmerztherapie: Medizinische und Juristische Aspekte [Limits of pain treatment: medical and judicial aspects]. Schmerz. 2011 Aug;25(4):377-80, 382-92. German. doi: 10.1007/s00482-011-1073-9. PMID: 21698434.	REVIEW
190	Tektonidou MG, Dasgupta A, Ward MM. Suicidal ideation among adults with arthritis: prevalence and subgroups at highest risk. Data from the 2007-2008 National Health and Nutrition Examination Survey. Arthritis Care Res (Hoboken). 2011 Sep;63(9):1322-33. doi: 10.1002/acr.20516. PMID: 21671421; PMCID: PMC3169713.	UNFOCUSED
191	Prati C, Claudepierre P, Pham T, Wendling D. Mortality in spondylarthritis. Joint Bone Spine. 2011 Oct;78(5):466-70. doi: 10.1016/j.jbspin.2011.02.012. Epub 2011 Mar 29. PMID: 21450507.	REVIEW
192	Capobianco A, Monno A, Cottone L, Venneri MA, Biziato D, Di Puppo F, Ferrari S, De Palma M, Manfredi AA, Rovere-Querini P. Proangiogenic Tie2(+) macrophages infiltrate human and murine endometriotic lesions and dictate their growth in a mouse model of the disease. Am J Pathol. 2011 Nov;179(5):2651-9. doi: 10.1016/j.ajpath.2011.07.029. Epub 2011 Sep 13. PMID: 21924227; PMCID: PMC3204092.	ANIMAL
193	Maneeton B, Maneeton N, Mahathep P. Prevalence of depression and its correlations: a cross-sectional study in Thai cancer patients. Asian Pac J Cancer Prev. 2012;13(5):2039-43. doi: 10.7314/apjcp.2012.13.5.2039. PMID: 22901168.	INCLUDED
194	Ferrand E, Dreyfus JF, Chastrusse M, Ellien F, Lemaire F, Fischler M. Evolution of requests to hasten death among patients managed by palliative care teams in France: a multicentre cross-sectional survey (DemandE). Eur J Cancer. 2012 Feb;48(3):368-76. doi: 10.1016/j.ejca.2011.09.020. Epub 2011 Oct 28. PMID: 22036873.	UNFOCUSED

195	Yang P, Wang L, Xie XQ. Latest advances in novel cannabinoid CB(2) ligands for drug abuse and their therapeutic potential. Future Med Chem. 2012 Feb;4(2):187-204. doi: 10.4155/fmc.11.179. PMID: 22300098; PMCID: PMC3501202.	REVIEW
196	Johnson TV, Garlow SJ, Brawley OW, Master VA. Peak window of suicides occurs within the first month of diagnosis: implications for clinical oncology. Psychooncology. 2012 Apr;21(4):351-6. doi: 10.1002/pon.1905. Epub 2011 Jan 24. PMID: 21264989.	UNFOCUSED
197	Fukui S, Alberti V, Mallios A, Soury P, Gigou F. Early and mid-term results of total laparoscopic bypass for aortoiliac occlusive lesions. J Cardiovasc Surg (Torino). 2012 Apr;53(2):235-9. PMID: 22456647.	UNFOCUSED
198	Sinha VK, Basu S, Sarkhel S. Euthanasia: An Indian perspective. Indian J Psychiatry. 2012 Apr;54(2):177-83. doi: 10.4103/0019-5545.99537. PMID: 22988327; PMCID: PMC3440914.	REVIEW
199	Jansson C, Mittendorfer-Rutz E, Alexanderson K. Sickness absence because of musculoskeletal diagnoses and risk of all-cause and cause-specific mortality: a nationwide Swedish cohort study. Pain. 2012 May;153(5):998-1005. doi: 10.1016/j.pain.2012.01.028. Epub 2012 Mar 14. PMID: 22421427.	UNFOCUSED
200	Lyttle MD, Verma S, Isaac R. Transdermal fentanyl in deliberate overdose in pediatrics. Pediatr Emerg Care. 2012 May;28(5):463-4. doi: 10.1097/PEC.0b013e31825358b4. PMID: 22561319.	CASE
201	McPherson T. My mum wanted assisted dying but we watched her die slowly and in pain. BMJ. 2012 Jun 13;344:e4007. doi: 10.1136/bmj.e4007. PMID: 22695305.	COMMENT
202	Kim KH, Dmitriev I, O'Malley JP, Wang M, Saddekni S, You Z, Preuss MA, Harris RD, Aurigemma R, Siegal GP, Zinn KR, Curiel DT, Alvarez RD. A phase I clinical trial of Ad5.SSTR/TK.RGD, a novel infectivity-enhanced bicistronic adenovirus, in patients with recurrent gynecologic cancer. Clin Cancer Res. 2012 Jun 15;18(12):3440-51. doi: 10.1158/1078-0432.CCR-11-2852. Epub 2012 Apr 17. PMID: 22510347; PMCID: PMC4065594.	UNFOCUSED
203	Manchikanti L, Abdi S, Atluri S, Balog CC, Benyamin RM, Boswell MV, Brown KR, Bruel BM, Bryce DA, Burks PA, Burton AW, Calodney AK, Caraway DL, Cash KA, Christo PJ, Damron KS, Datta S, Deer TR, Diwan S, Eriator I, Falco FJ, Fellows B, Geffert S, Gharibo CG, Glaser SE, Grider JS, Hameed H, Hameed M, Hansen H, Harned ME, Hayek SM, Helm S 2nd, Hirsch JA, Janata JW, Kaye AD, Kaye AM, Kloth DS, Koyyalagunta D, Lee M, Malla Y, Manchikanti KN, McManus CD, Pampati V, Parr AT, Pasupuleti R, Patel VB, Sehgal N, Silverman SM, Singh V, Smith HS, Snook LT, Solanki DR, Tracy DH, Vallejo R, Wargo BW; American Society of Interventional Pain Physicians. American Society of Interventional Pain Physicians (ASIPP) guidelines for responsible opioid prescribing in chronic non-cancer pain: Part 2 --guidance. Pain Physician. 2012 Jul;15(3 Suppl):S67-116. PMID: 22786449.	UNFOCUSED
204	Manchikanti L, Helm S 2nd, Fellows B, Janata JW, Pampati V, Grider JS, Boswell MV. Opioid epidemic in the United States. Pain Physician. 2012 Jul;15(3 Suppl):ES9-38. PMID: 22786464.	REVIEW
205	Kowal J, Wilson KG, McWilliams LA, Péloquin K, Duong D. Self-perceived burden in chronic pain: relevance, prevalence, and predictors. Pain. 2012 Aug;153(8):1735-1741. doi: 10.1016/j.pain.2012.05.009. Epub 2012 Jun 14. PMID: 22703692; PMCID: PMC3999031.	UNFOCUSED
206	De Sanctis V, Filati G, Fiscina B, Marsciani A, Piacentini G, Timoncini G, Reggiani L, Zucchini A. Adolescent health care in Italy: a mini-review. Georgian Med News. 2012 Sep;(210):8-12. English, Georgian. PMID: 23045413.	REVIEW
207	Sankar V, Shakeel M, Keh S, Ah-See KW. A case of a 'lost' nasogastric tube. J Laryngol Otol. 2012 Dec;126(12):1296-8. doi: 10.1017/S0022215112002447. Epub 2012 Oct 26. PMID: 23098087.	CASE
208	Koenig HG. Religion, spirituality, and health: the research and clinical implications. ISRN Psychiatry. 2012 Dec 16;2012:278730. doi: 10.5402/2012/278730. PMID: 23762764; PMCID: PMC3671693.	REVIEW
209	Madadi P, Hildebrandt D, Lauwers AE, Koren G. Characteristics of opioid- users whose death was related to opioid-toxicity: a population-based study in Ontario, Canada. PLoS One. 2013;8(4):e60600. doi: 10.1371/journal.pone.0060600. Epub 2013 Apr 5. PMID: 23577131; PMCID: PMC3618438.	UNFOCUSED

210	Longerich B. Niemand hat das Recht, über andere zu urteilen [No one has the right to judge others]. Krankenpfl Soins Infirm. 2013;106(11):10-4, 60-4, 84-8. French, German, Italian. PMID: 24479218.	UNFOCUSED
211	Renaud G. Es braucht ein Herz und viel Zeit [It takes a heart and a lot of time]. Krankenpfl Soins Infirm. 2013;106(11):12-3, 62-3, 86-7. French, German, Italian. PMID: 24479219.	EDITORIAL
212	Allah KC, Yéo S, Kossoko H, Assi Djè Bi Djè V, Richard Kadio M. Cicatrices chéloïdes sur peau noire : mythe ou réalité [Keloid scars on black skin: myth or reality]. Ann Chir Plast Esthet. 2013 Apr;58(2):115-22. French. doi: 10.1016/j.anplas.2012.02.005. Epub 2012 Apr 27. PMID: 22542368.	UNFOCUSED
213	Ukai K, Okajima A, Yamauchi A, Sasaki E, Yamaguchi Y, Kimura H, Aleksic B, Ozaki N. Total palliative care for a patient with multiple cerebral infarctions that occurred repeatedly in association with gastric cancer (Trousseau's syndrome). Palliat Support Care. 2013 Apr;11(2):169-72. doi: 10.1017/S1478951512000624. Epub 2012 Jul 30. PMID: 22840285.	CASE
214	Gamondi C, Pott M, Payne S. Families' experiences with patients who died after assisted suicide: a retrospective interview study in southern Switzerland. Ann Oncol. 2013 Jun;24(6):1639-44. doi: 10.1093/annonc/mdt033. Epub 2013 Feb 27. PMID: 23446094.	UNFOCUSED
215	Lowery AE, Starr T, Dhingra LK, Rogak L, Hamrick-Price JR, Farberov M, Kirsh KL, Saltz LB, Breitbart WS, Passik SD. Frequency, characteristics, and correlates of pain in a pilot study of colorectal cancer survivors 1-10 years post-treatment. Pain Med. 2013 Nov;14(11):1673-80. doi: 10.1111/pme.12223. Epub 2013 Sep 6. PMID: 24010414; PMCID: PMC3971863.	UNFOCUSED
216	Jaiswal R, Alici Y, Breitbart W. A comprehensive review of palliative care in patients with cancer. Int Rev Psychiatry. 2014 Feb;26(1):87-101. doi: 10.3109/09540261.2013.868788. PMID: 24716503.	REVIEW
217	Duffy TP. Physician assistance in dying: a subtler slippery slope. Hastings Cent Rep. 2014 Mar-Apr;44(2):1p following 48. doi: 10.1002/hast.290. PMID: 24634049.	EDITORIAL
218	Mosich V, Müller-Busch HC. Suizid durch Intoxikation während einer mobilen palliativen Begleitung eines Patienten mit Bronchuskarzinom [Suicidal drug overdose while receiving palliative home care: a case report]. Wien Med Wochenschr. 2014 May;164(9-10):184-8. German. doi: 10.1007/s10354-014-0273-1. Epub 2014 Apr 15. PMID: 24733303.	CASE
219	Cheatle MD, Wasser T, Foster C, Olugbodi A, Bryan J. Prevalence of suicidal ideation in patients with chronic non-cancer pain referred to a behaviorally based pain program. Pain Physician. 2014 May-Jun;17(3):E359-67. PMID: 24850117.	UNFOCUSED
220	Lehuluante A, Fransson P. Are there specific health-related factors that can accentuate the risk of suicide among men with prostate cancer? Support Care Cancer. 2014 Jun;22(6):1673-8. doi: 10.1007/s00520-014-2150-2. Epub 2014 Feb 11. PMID: 24515278; PMCID: PMC4008778.	UNFOCUSED
221	Usenko O. A Russian tragedy. J Pain Palliat Care Pharmacother. 2014 Jun;28(2):178-9. doi: 10.3109/15360288.2014.911803. Epub 2014 Apr 30. PMID: 24784494.	EDITORIAL
222	Abbott CH, Prigerson HG, Maciejewski PK. The influence of patients' quality of life at the end of life on bereaved caregivers' suicidal ideation. J Pain Symptom Manage. 2014 Sep;48(3):459-64. doi: 10.1016/j.jpainsymman.2013.09.011. Epub 2013 Dec 8. PMID: 24321508; PMCID: PMC4048331.	UNFOCUSED
223	Sharpe M, Walker J, Holm Hansen C, Martin P, Symeonides S, Gourley C, Wall L, Weller D, Murray G; SMaRT (Symptom Management Research Trials) Oncology-2 Team. Integrated collaborative care for comorbid major depression in patients with cancer (SMaRT Oncology-2): a multicentre randomised controlled effectiveness trial. Lancet. 2014 Sep 20;384(9948):1099-108. doi: 10.1016/S0140-6736(14)61231-9. Epub 2014 Aug 27. PMID: 25175478.	UNFOCUSED
224	Han DH. The association between temporomandibular disorders and suicide ideation in a representative sample of the South Korean population. J Oral Facial Pain Headache. 2014 Fall;28(4):338-45. doi: 10.11607/ofph.1229. PMID: 25347169.	INCLUDED

225	Recklitis CJ, Zhou ES, Zwemer EK, Hu JC, Kantoff PW. Suicidal ideation in prostate cancer survivors: understanding the role of physical and psychological health outcomes. Cancer. 2014 Nov 1;120(21):3393-400. doi: 10.1002/cncr.28880. Epub 2014 Jun 24. PMID: 24962506.	INCLUDED
226	Castaneto MS, Gorelick DA, Desrosiers NA, Hartman RL, Pirard S, Huestis MA. Synthetic cannabinoids: epidemiology, pharmacodynamics, and clinical implications. Drug Alcohol Depend. 2014 Nov 1;144:12-41. doi: 10.1016/j.drugalcdep.2014.08.005. Epub 2014 Aug 18. PMID: 25220897; PMCID: PMC4253059.	REVIEW
227	Doron D, Wexler ID, Shabtai E, Corn BW. Israeli Dying Patient Act: physician knowledge and attitudes. Am J Clin Oncol. 2014 Dec;37(6):597-602. doi: 10.1097/COC.0b013e318295b022. PMID: 23660598.	UNFOCUSED
228	Omerov P, Steineck G, Dyregrov K, Runeson B, Nyberg U. The ethics of doing nothing. Suicide-bereavement and research: ethical and methodological considerations. Psychol Med. 2014 Dec;44(16):3409-20. doi: 10.1017/S0033291713001670. Epub 2013 Jul 19. PMID: 23867073; PMCID: PMC4255316.	UNFOCUSED
229	Ekholm O, Kurita GP, Hjsted J, Juel K, Sjgren P. Chronic pain, opioid prescriptions, and mortality in Denmark: A population-based cohort study. Pain. 2014 Dec;155(12):2486-2490. doi: 10.1016/j.pain.2014.07.006. Epub 2014 Jul 11. PMID: 25020002.	UNFOCUSED
230	Radbruch L, Ostgathe C, Nauck F. Unerträgliche Schmerzen–wie gut ist die Schmerzmedizin? Schmerz. 2014 Dec;28(6):571-2. German. PMID: 25610933.	EDITORIAL
231	Parpa E, Tsilika E, Gennimata V, Mystakidou K. Elderly cancer patients' psychopathology: a systematic review: aging and mental health. Arch Gerontol Geriatr. 2015 Jan-Feb;60(1):9-15. doi: 10.1016/j.archger.2014.09.008. Epub 2014 Sep 22. PMID: 25266607.	REVIEW
232	Schiff LB, Holland KM, Stone DM, Logan J, Marshall KJ, Martell B, Bartholow B. Acute and Chronic Risk Preceding Suicidal Crises Among Middle-Aged Men Without Known Mental Health and/or Substance Abuse Problems: An Exploratory Mixed-Methods Analysis. Crisis. 2015;36(5):304-15. doi: 10.1027/0227-5910/a000329. Epub 2015 Jun 30. PMID: 26122257; PMCID: PMC6113685.	UNFOCUSED
233	Campbell G, Nielsen S, Bruno R, Lintzeris N, Cohen M, Hall W, Larance B, Mattick RP, Degenhardt L. The Pain and Opioids IN Treatment study: characteristics of a cohort using opioids to manage chronic non-cancer pain. Pain. 2015 Feb;156(2):231-242. doi: 10.1097/01.j.pain.0000460303.63948.8e. PMID: 25599444.	UNFOCUSED
234	Smith KA, Harvath TA, Goy ER, Ganzini L. Predictors of pursuit of physician-assisted death. J Pain Symptom Manage. 2015 Mar;49(3):555-61. doi: 10.1016/j.jpainsymman.2014.06.010. Epub 2014 Aug 10. PMID: 25116913.	UNFOCUSED
235	Nau JY. Pour apprendre à mourir, la France devra cesser de regarder vers la Suisse [To learn how to die, France will have to stop looking towards Switzerland]. Rev Med Suisse. 2015 Mar 25;11(467):742-3. French. PMID: 26027207.	UNFOCUSED
236	Fujimura Y, Nakahara O, Ohshima S, Baba H. [Efficacy of a fentanyl citrate buccal tablet for esophageal cancer pain management in a patient unable to take oral medication]. Gan To Kagaku Ryoho. 2015 Apr;42(4):515-7. Japanese. PMID: 25963705.	CASE
237	Erdek M. Pain medicine and palliative care as an alternative to euthanasia in end-of-life cancer care. Linacre Q. 2015 May;82(2):128-34. doi: 10.1179/2050854915Y.0000000003. PMID: 25999611; PMCID: PMC4434784.	REVIEW
238	Smolensky MH, Portaluppi F, Manfredini R, Hermida RC, Tiseo R, Sackett- Lundeen LL, Haus EL. Diurnal and twenty-four hour patterning of human diseases: acute and chronic common and uncommon medical conditions. Sleep Med Rev. 2015 Jun;21:12-22. doi: 10.1016/j.smrv.2014.06.005. Epub 2014 Jul 18. PMID: 25129839.	REVIEW
239	Fishbain DA, Lewis JE, Gao J. The pain--suffering association, a review. Pain Med. 2015 Jun;16(6):1057-72. doi: 10.1111/pme.12686. Epub 2015 Jan 13. PMID: 25586894.	REVIEW
240	LiPuma SH, DeMarco JP. Expanding the Use of Continuous Sedation Until Death: Moving Beyond the Last Resort for the Terminally Ill. J Clin Ethics. 2015 Summer;26(2):121-31. PMID: 26132059.	CASE

241	Campbell G, Darke S, Bruno R, Degenhardt L. The prevalence and correlates of chronic pain and suicidality in a nationally representative sample. Aust N Z J Psychiatry. 2015 Sep;49(9):803-11. doi: 10.1177/0004867415569795. Epub 2015 Feb 19. PMID: 25698809.	UNFOCUSED
242	Campbell G, Bruno R, Darke S, Degenhardt L. Associations of borderline personality with pain, problems with medications and suicidality in a community sample of chronic non-cancer pain patients prescribed opioids for pain. Gen Hosp Psychiatry. 2015 Sep-Oct;37(5):434-40. doi: 10.1016/j.genhosppsych.2015.05.004. Epub 2015 May 16. PMID: 26112358.	UNFOCUSED
243	Fricton J, Anderson K, Clavel A, Fricton R, Hathaway K, Kang W, Jaeger B, Maixner W, Pesut D, Russell J, Weisberg MB, Whitebird R. Preventing Chronic Pain: A Human Systems Approach-Results From a Massive Open Online Course. Glob Adv Health Med. 2015 Sep;4(5):23-32. doi: 10.7453/gahmj.2015.048. Epub 2015 Sep 1. PMID: 26421231; PMCID: PMC4563888.	UNFOCUSED
244	Verma R, Mohan B, Attri JP, Chatrath V, Bala A, Singh M. Anesthesiologist: The silent force behind the scene. Anesth Essays Res. 2015 Sep-Dec;9(3):293-7. doi: 10.4103/0259-1162.159775. PMID: 26712962; PMCID: PMC4683484.	REVIEW
245	Diagnosing, Screening, and Monitoring Depression in the Elderly: A Review of Guidelines [Internet]. Ottawa (ON): Canadian Agency for Drugs and Technologies in Health; 2015 Sep 8. PMID: 26468558.	REVIEW
246	Imboden C, Hatzinger M. Suizidalität im somatischen Spital - Perspektive der Konsiliar- und Liaisonpsychiatrie [Suicidality at the general hospital – perspective of consultation and liaison psychiatry]. Ther Umsch. 2015 Oct;72(10):637-42. German. doi: 10.1024/0040-5930/a000730. PMID: 26423882.	REVIEW

247	GBD 2013 DALYs and HALE Collaborators; Murray CJ, Barber RM, Foreman KJ, Abbasoglu Ozgoren A, Abd-Allah F, Abera SF, Aboyans V, Abraham JP, Abubakar I, Abu-Raddad LJ, Abu-Rmeileh NM, Achoki T, Ackerman IN, Ademi Z, Adou AK, Adsuar JC, Afshin A, Agardh EE, Alam SS, Alasfoor D, Albittar MI, Alegretti MA, Alemu ZA, Alfonso-Cristancho R, Alhabib S, Ali R, Alla F, Allebeck P, Almazroa MA, Alsharif U, Alvarez E, Alvis-Guzman N, Amare AT, Ameh EA, Amini H, Ammar W, Anderson HR, Anderson BO, Antonio CA, Anwari P, Arnlöv J, Arsic Arsenijevic VS, Artaman A, Asghar RJ, Assadi R, Atkins LS, Avila MA, Awuah B, Bachman VF, Badawi A, Bahit MC, Balakrishnan K, Banerjee A, Barker-Collo SL, Barquera S, Barregard L, Barrero LH, Basu A, Basu S, Basulaiman MO, Beardsley J, Bedi N, Beghi E, Bekele T, Bell ML, Benjet C, Bennett DA, Bensenor IM, Benzian H, Bernabé E, Bertozzi-Villa A, Beyene TJ, Bhala N, Bhalla A, Bhutta ZA, Bienhoff K, Bikbov B, Biryukov S, Blore JD, Blosser CD, Blyth FM, Bohensky MA, Bolliger IW, Bora Başara B, Bornstein NM, Bose D, Boufous S, Bourne RR, Boyers LN, Brainin M, Brayne CE, Brazinova A, Breitborde NJ, Brenner H, Briggs AD, Brooks PM, Brown JC, Brugha TS, Buchbinder R, Buckle GC, Budke CM, Bulchis A, Bulloch AG, Campos- Nonato IR, Carabin H, Carapetis JR, Cárdenas R, Carpenter DO, Caso V, Castañeda- Orjuela CA, Castro RE, Catalá-López F, Cavalleri F, Çavlin A, Chadha VK, Chang JC, Charlson FJ, Chen H, Chen W, Chiang PP, Chimed-Ochir O, Chowdhury R, Christensen H, Christophi CA, Cirillo M, Coates MM, Coffeng LE, Coggeshall MS, Colistro V, Colquhoun SM, Cooke GS, Cooper C, Cooper LT, Coppola LM, Cortinovis M, Criqui MH, Crump JA, Cuevas-Nasu L, Danawi H, Dandona L, Dandona R, Dansereau E, Dargan PI, Davey G, Davis A, Davitoiu DV, Dayama A, De Leo D, Degenhardt L, Del Pozo-Cruz B, Dellavalle RP, Deribe K, Derrett S, Des Jarlais DC, Dessalegn M, Dharmaratne SD, Dherani MK, Diaz-Torné C, Dicker D, Ding EL, Dokova K, Dorsey ER, Driscoll TR, Duan L, Duber HC, Ebel BE, Edmond KM, Elshrek YM, Endres M, Ermakov SP, Erskine HE, Eshrati B, Esteghamati A, Estep K, Faraon EJ, Farzadfar F, Fay DF, Feigin VL, Felson DT, Fereshtehnejad SM, Fernandes JG, Ferrari AJ, Fitzmaurice C, Flaxman AD, Fleming TD, Foigt N, Forouzanfar MH, Fowkes FG, Paleo UF, Franklin RC, Fürst T, Gabbe B, Gaffikin L, Gankpé FG, Geleijnse JM, Gessner BD, Gething P, Gibney KB, Giroud M, Giussani G, Gomez Dantes H, Gona P, González-Medina D, Gosselin RA, Gotay CC, Goto A, Gouda HN, Graetz N, Gugnani HC, Gupta R, Gupta R, Gutiérrez RA, Haagsma J, Hafezi-Nejad N, Hagan H, Halasa YA, Hamadeh RR, Hamavid H, Hammami M, Hancock J, Hankey GJ, Hansen GM, Hao Y, Harb HL, Haro JM, Havmoeller R, Hay SI, Hay RJ, Heredia-Pi IB, Heuton KR, Heydarpour P, Higashi H, Hijar M, Hoek HW, Hoffman HJ, Hosgood HD, Hossain M, Hotez PJ, Hoy DG, Hsairi M, Hu G, Huang C, Huang JJ, Husseini A, Huynh C, Iannarone ML, Iburg KM, Innos K, Inoue M, Islami F, Jacobsen KH, Jarvis DL, Jassal SK, Jee SH, Jeemon P, Jensen PN, Jha V, Jiang G, Jiang Y, Jonas JB, Juel K, Kan H, Karch A, Karema CK, Karimkhani C, Karthikeyan G, Kassebaum NJ, Kaul A, Kawakami N, Kazanjan K, Kemp AH, Kengne AP, Keren A, Khader YS, Khalifa SE, Khan EA, Khan G, Khang YH, Kieling C, Kim D, Kim S, Kim Y, Kinfu Y, Kinge JM, Kivipelto M, Knibbs LD, Knudsen AK, Kokubo Y, Kosen S, Krishnaswami S, Kuate Defo B, Kucuk Bicer B, Kuipers EJ, Kulkarni C, Kulkarni VS, Kumar GA, Kyu HH, Lai T, Lalloo R, Lallukka T, Lam H, Lan Q, Lansingh VC, Larsson A, Lawrynowicz AE, Leasher JL, Leigh J, Leung R, Levitz CE, Li B, Li Y, Li Y, Lim SS, Lind M, Lipshultz SE, Liu S, Liu Y, Lloyd BK, Lofgren KT, Logroscino G, Looker KJ, Lortet-Tieulent J, Lotufo PA, Lozano R, Lucas RM, Lunevicius R, Lyons RA, Ma S, Macintyre MF, Mackay MT, Majdan M, Malekzadeh R, Marcenes W, Margolis DJ, Margono C, Marzan MB, Masci JR, Mashal MT, Matzopoulos R, Mayosi BM, Mazorodze TT, Mcgill NW, Mcgrath JJ, Mckee M, Mclain A, Meaney PA, Medina C, Mehndiratta MM, Mekonnen W, Melaku YA, Meltzer M, Memish ZA, Mensah GA, Meretoja A, Mhimbira FA, Micha R, Miller TR, Mills EJ, Mitchell PB, Mock CN, Mohamed Ibrahim N, Mohammad KA, Mokdad AH, Mola GL, Monasta L, Montañez Hernandez JC, Montico M, Montine TJ, Mooney MD, Moore AR, Moradi-Lakeh M, Moran AE, Mori R, Moschandreas J, Moturi WN, Moyer ML, Mozaffarian D, Msemburi WT, Mueller UO, Mukaigawara M, Mullany EC, Murdoch ME, Murray J, Murthy KS, Naghavi M, Naheed A, Naidoo KS, Naldi L, Nand D, Nangia V, Narayan KM, Nejjari C, Neupane SP, Newton CR, Ng M, Ngalesoni FN, Nguyen G, Nisar MI, Nolte S, Norheim OF, Norman RE, Norrving B, Nyakarahuka L, Oh IH, Ohkubo T, Ohno SL, Olusanya BO, Opio JN, Ortblad K, Ortiz A, Pain AW, Pandian JD, Panelo CI, Papachristou C, Park EK, Park JH, Patten SB, Patton GC, Paul VK, Pavlin BI, Pearce N, Pereira DM, Perez-Padilla R, Perez-Ruiz F, Perico N, Pervaiz A, Pesudovs K, Peterson CB, Petzold M, Phillips MR, Phillips BK, Phillips DE, Piel FB, Plass D, Poenaru D, Polinder S, Pope D, Popova S, Poulton RG, Pourmalek F, Prabhakaran D, Prasad NM, Pullan RL, Qato DM, Quistberg DA, Rafay A, Rahimi K, Rahman SU, Raju M, Rana SM, Razavi H, Reddy KS, Refaat A, Remuzzi G, Resnikoff S, Ribeiro AL, Richardson L, Richardus JH, Roberts DA, Rojas-Rueda D, Ronfani L, Roth GA, Rothenbacher D, Rothstein DH, Rowley JT, Roy N, Ruhago GM, Saeedi MY, Saha S, Sahraian MA, Sampson UK, Sanabria JR, Sandar L, Santos IS, Satpathy M, Sawhney M, Scarborough P, Schneider IJ, Schöttker B, Schumacher AE, Schwebel DC, Scott JG, Seedat S, Sepanlou SG, Serina PT, Servan-Mori EE, Shackelford KA, Shaheen A, Shahraz S, Shamah Levy T, Shangguan S, She J, Sheikhbahaei S, Shi P, Shibuya K, Shinohara Y,	UNFOCUSED

	Shiri R, Shishani K, Shiue I, Shrime MG, Sigfusdottir ID, Silberberg DH, Simard EP, Sindi S, Singh A, Singh JA, Singh L, Skirbekk V, Slepak EL, Sliwa K, Soneji S, Søreide K, Soshnikov S, Sposato LA, Sreeramareddy CT, Stanaway JD, Stathopoulou V, Stein DJ, Stein MB, Steiner C, Steiner TJ, Stevens A, Stewart A, Stovner LJ, Stroumpoulis K, Sunguya BF, Swaminathan S, Swaroop M, Sykes BL, Tabb KM, Takahashi K, Tandon N, Tanne D, Tanner M, Tavakkoli M, Taylor HR, Te Ao BJ, Tediosi F, Temesgen AM, Templin T, Ten Have M, Tenkorang EY, Terkawi AS, Thomson B, Thorne-Lyman AL, Thrift AG, Thurston GD, Tillmann T, Tonelli M, Topouzis F, Toyoshima H, Traebert J, Tran BX, Trillini M, Truelsen T, Tsilimbaris M, Tuzcu EM, Uchendu US, Ukwaja KN, Undurraga EA, Uzun SB, Van Brakel WH, Van De Vijver S, van Gool CH, Van Os J, Vasankari TJ, Venketasubramanian N, Violante FS, Vlassov VV, Vollset SE, Wagner GR, Wagner J, Waller SG, Wan X, Wang H, Wang J, Wang L, Warouw TS, Weichenthal S, Weiderpass E, Weintraub RG, Wenzhi W, Werdecker A, Westerman R, Whiteford HA, Wilkinson JD, Williams TN, Wolfe CD, Wolock TM, Woolf AD, Wulf S, Wurtz B, Xu G, Yan LL, Yano Y, Ye P, Yentür GK, Yip P, Yonemoto N, Yoon SJ, Younis MZ, Yu C, Zaki ME, Zhao Y, Zheng Y, Zonies D, Zou X, Salomon JA, Lopez AD, Vos T. Global, regional, and national disability- adjusted life years (DALYs) for 306 diseases and injuries and healthy life expectancy (HALE) for 188 countries, 1990-quantifying the epidemiological transition. Lancet. 2015 Nov 28;386(10009):2145-91. doi: 10.1016/S0140-6736(15)61340-X. Epub 2015 Aug 28. PMID: 26321261; PMCID: PMC4673910.	
248	Umezawa S, Fujimori M, Matsushima E, Kinoshita H, Uchitomi Y. Preferences of advanced cancer patients for communication on anticancer treatment cessation and the transition to palliative care. Cancer. 2015 Dec 1;121(23):4240-9. doi: 10.1002/cncr.29635. Epub 2015 Aug 26. PMID: 26308376.	UNFOCUSED
249	Bitsko MJ, Cohen D, Dillon R, Harvey J, Krull K, Klosky JL. Psychosocial Late Effects in Pediatric Cancer Survivors: A Report From the Children's Oncology Group. Pediatr Blood Cancer. 2016 Feb;63(2):337-43. doi: 10.1002/pbc.25773. Epub 2015 Oct 21. PMID: 26488337; PMCID: PMC4715481.	REVIEW
250	Bruns F, Blumenthal S, Hohendorf G. Organisierte Suizidbeihilfe in Deutschland: Medizinische Diagnosen und persönliche Motive von 117 Suizidenten [Assisted suicide in Germany: medical diagnoses and personal reasons of 117 decedents]. Dtsch Med Wochenschr. 2016 Feb;141(4):e32-7. German. doi: 10.1055/s-0041-111182. Epub 2016 Feb 17. PMID: 26886045.	UNFOCUSED
251	Fegg M, Kraus S, Graw M, Bausewein C. Physical compared to mental diseases as reasons for committing suicide: a retrospective study. BMC Palliat Care. 2016 Feb 9;15:14. doi: 10.1186/s12904-016-0088-5. PMID: 26860949; PMCID: PMC4746811.	UNFOCUSED
252	Anneser J, Jox RJ, Thurn T, Borasio GD. Physician-assisted suicide, euthanasia and palliative sedation: attitudes and knowledge of medical students. GMS J Med Educ. 2016 Feb 15;33(1):Doc11. doi: 10.3205/zma001010. PMID: 26958648; PMCID: PMC4766939.	CASE
253	Spangenberg L, Zenger M, Garcia-Torres F, Mueller V, Reck M, Mehnert A, Vehling S. Dimensionality, Stability, and Validity of the Beck Hopelessness Scale in Cancer Patients Receiving Curative and Palliative Treatment. J Pain Symptom Manage. 2016 Mar;51(3):615-22. doi: 10.1016/j.jpainsymman.2015.11.008. Epub 2015 Dec 2. PMID: 26654946.	UNFOCUSED

254	Global Burden of Disease Pediatrics Collaboration; Kyu HH, Pinho C, Wagner JA, Brown JC, Bertozzi-Villa A, Charlson FJ, Coffeng LE, Dandona L, Erskine HE, Ferrari AJ, Fitzmaurice C, Fleming TD, Forouzanfar MH, Graetz N, Guinovart C, Haagsma J, Higashi H, Kassebaum NJ, Larson HJ, Lim SS, Mokdad AH, Moradi-Lakeh M, Odell SV, Roth GA, Serina PT, Stanaway JD, Misganaw A, Whiteford HA, Wolock TM, Wulf Hanson S, Abd-Allah F, Abera SF, Abu-Raddad LJ, AlBuhairan FS, Amare AT, Antonio CA, Artaman A, Barker-Collo SL, Barrero LH, Benjet C, Bensenor IM, Bhutta ZA, Bikbov B, Brazinova A, Campos-Nonato I, Castañeda-Orjuela CA, Catalá- López F, Chowdhury R, Cooper C, Crump JA, Dandona R, Degenhardt L, Dellavalle RP, Dharmaratne SD, Faraon EJ, Feigin VL, Fürst T, Geleijnse JM, Gessner BD, Gibney KB, Goto A, Gunnell D, Hankey GJ, Hay RJ, Hornberger JC, Hosgood HD, Hu G, Jacobsen KH, Jayaraman SP, Jeemon P, Jonas JB, Karch A, Kim D, Kim S, Kokubo Y, Kuate Defo B, Kucuk Bicer B, Kumar GA, Larsson A, Leasher JL, Leung R, Li Y, Lipshultz SE, Lopez AD, Lotufo PA, Lunevicius R, Lyons RA, Majdan M, Malekzadeh R, Mashal T, Mason-Jones AJ, Melaku YA, Memish ZA, Mendoza W, Miller TR, Mock CN, Murray J, Nolte S, Oh IH, Olusanya BO, Ortblad KF, Park EK, Paternina Caicedo AJ, Patten SB, Patton GC, Pereira DM, Perico N, Piel FB, Polinder S, Popova S, Pourmalek F, Quistberg DA, Remuzzi G, Rodriguez A, Rojas-Rueda D, Rothenbacher D, Rothstein DH, Sanabria J, Santos IS, Schwebel DC, Sepanlou SG, Shaheen A, Shiri R, Shiue I, Skirbekk V, Sliwa K, Sreeramareddy CT, Stein DJ, Steiner TJ, Stovner LJ, Sykes BL, Tabb KM, Terkawi AS, Thomson AJ, Thorne-Lyman AL, Towbin JA, Ukwaja KN, Vasankari T, Venketasubramanian N, Vlassov VV, Vollset SE, Weiderpass E, Weintraub RG, Werdecker A, Wilkinson JD, Woldeyohannes SM, Wolfe CD, Yano Y, Yip P, Yonemoto N, Yoon SJ, Younis MZ, Yu C, El Sayed Zaki M, Naghavi M, Murray CJ, Vos T. Global and National Burden of Diseases and Injuries Among Children and Adolescents Between 1990 and Findings From the Global Burden of Disease 2013 Study. JAMA Pediatr. 2016 Mar;170(3):267-87. doi: 10.1001/jamapediatrics.2015.4276. PMID: 26810619; PMCID: PMC5076765.	LUMPING
255	Gigon A. Forte augmentation des suicides assistés en 2015 [Surge in assisted suicides in 2015]. Rev Med Suisse. 2016 Mar 9;12(509):527. French. PMID: 27089649.	UNFOCUSED

256	Lewis C, Darnell D, Kerns S, Monroe-DeVita M, Landes SJ, Lyon AR, Stanick C, Dorsey S, Locke J, Marriott B, Puspitasari A, Dorsey C, Hendricks K, Pierson A, Fizur P, Comtois KA, Palinkas LA, Chamberlain P, Aarons GA, Green AE, Ehrhart MG, Trott EM, Willging CE, Fernandez ME, Woolf NH, Liang SL, Heredia NI, Kegler M, Risendal B, Dwyer A, Young V, Campbell D, Carvalho M, Kellar-Guenther Y, Damschroder LJ, Lowery JC, Ono SS, Carlson KF, Cottrell EK, O'Neil ME, Lovejoy TL, Arch JJ, Mitchell JL, Lewis CC, Marriott BR, Scott K, Coldiron JS, Bruns EJ, Hook AN, Graham BC, Jordan K, Hanson RF, Moreland A, Saunders BE, Resnick HS, Stirman SW, Gutner CA, Gamarra J, Vogt D, Suvak M, Wachen JS, Dondanville K, Yarvis JS, Mintz J, Peterson AL, Borah EV, Litz BT, Molino A, McCaughan SY, Resick PA, Pandhi N, Jacobson N, Serrano N, Hernandez A, Schreiter EZ, Wietfeldt N, Karp Z, Pullmann MD, Lucenko B, Pavelle B, Uomoto JA, Negrete A, Cevasco M, Kerns SEU, Franks RP, Bory C, Miech EJ, Damush TM, Satterfield J, Satre D, Wamsley M, Yuan P, O'Sullivan P, Best H, Velasquez S, Barnett M, Brookman-Frazee L, Regan J, Stadnick N, Hamilton A, Lau A, Regan J, Hamilton A, Stadnick N, Barnett M, Lau A, Brookman-Frazee L, Stadnick N, Lau A, Barnett M, Regan J, Roesch S, Brookman-Frazee L, Powell BJ, Waltz TJ, Chinman MJ, Damschroder L, Smith JL, Matthieu MM, Proctor EK, Kirchner JE, Waltz TJ, Powell BJ, Chinman MJ, Damschroder LJ, Smith JL, Matthieu MJ, Proctor EK, Kirchner JE, Matthieu MM, Rosen CS, Waltz TJ, Powell BJ, Chinman MJ, Damschroder LJ, Smith JL, Proctor EK, Kirchner JE, Walker SC, Bishop AS, Lockhart M, Rodriguez AL, Manfredi L, Nevedal A, Rosenthal J, Blonigen DM, Mauricio AM, Dishion TD, Rudo-Stern J, Smith JD, Locke J, Wolk CB, Harker C, Olsen A, Shingledecker T, Barg F, Mandell D, Beidas RS, Hansen MC, Aranda MP, Torres-Vigil I, Hartzler B, Steinfeld B, Gildred T, Harlin Z, Shephard F, Ditty MS, Doyle A, Bickel JA III, Cristaudo K, Fox D, Combs S, Lischner DH, Van Dorn RA, Tueller SJ, Hinde JM, Karuntzos GT, Monroe-DeVita M, Peterson R, Darnell D, Berliner L, Dorsey S, Murray LK, Botanov Y, Kikuta B, Chen T, Navarro-Haro M, DuBose A, Korslund KE, Linehan MM, Harker CM, Karp EA, Edmunds SR, Ibañez LV, Stone WL, Andrews JH, Johnides BD, Hausman EM, Hawley KM, Prusaczyk B, Ramsey A, Baumann A, Colditz G, Proctor EK, Botanov Y, Kikuta B, Chen T, Navarro-Haro M, DuBose A, Korslund KE, Linehan MM, Harker CM, Karp EA, Edmunds SR, Ibañez LV, Stone WL, Choy-Brown M, Andrews JH, Johnides BD, Hausman EM, Hawley KM, Prusaczyk B, Ramsey A, Baumann A, Colditz G, Proctor EK, Meza RD, Dorsey S, Wiltsey-Stirman S, Sedlar G, Lucid L, Dorsey C, Marriott B, Zounlome N, Lewis C, Gutner CA, Monson CM, Shields N, Mastlej M, Landy MSH, Lane J, Stirman SW, Finn NK, Torres EM, Ehrhart MG, Aarons GA, Malte CA, Lott A, Saxon AJ, Boyd M, Scott K, Lewis CC, Pierce JD, Lorthios-Guilledroit A, Richard L, Filiatrault J, Hallgren K, Crotwell S, Muñoz R, Gius B, Ladd B, McCrady B, Epstein E, Clapp JD, Ruderman DE, Barwick M, Barac R, Zlotkin S, Salim L, Davidson M, Bunger AC, Powell BJ, Robertson HA, Botsko C, Landes SJ, Smith BN, Rodriguez AL, Trent LR, Matthieu MM, Powell BJ, Proctor EK, Harned MS, Navarro- Haro M, Korslund KE, Chen T, DuBose A, Ivanoff A, Linehan MM, Garcia AR, Kim M, Palinkas LA, Snowden L, Landsverk J, Sweetland AC, Fernandes MJ, Santos E, Duarte C, Kritski A, Krawczyk N, Nelligan C, Wainberg ML, Aarons GA, Sommerfeld DH, Chi B, Ezeanolue E, Sturke R, Kline L, Guay L, Siberry G, Bennett IM, Beidas R, Gold R, Mao J, Powers D, Vredevoogd M, Unutzer J, Schroeder J, Volpe L, Steffen J, Dorsey S, Pullmann MD, Kerns SEU, Jungbluth N, Berliner L, Thompson K, Segell E, McGee-Vincent P, Liu N, Walser R, Runnals J, Shaw RK, Landes SJ, Rosen C, Schmidt J, Calhoun P, Varkovitzky RL, Landes SJ, Drahota A, Martinez JI, Brikho B, Meza R, Stahmer AC, Aarons GA, Williamson A, Rubin RM, Powell BJ, Hurford MO, Weaver SL, Beidas RS, Mandell DS, Evans AC, Powell BJ, Beidas RS, Rubin RM, Stewart RE, Wolk CB, Matlin SL, Weaver S, Hurford MO, Evans AC, Hadley TR, Mandell DS, Gerke DR, Prusaczyk B, Baumann A, Lewis EM, Proctor EK, McWilliam J, Brown J, Tucker M, Conte KP, Lyon AR, Boyd M, Melvin A, Lewis CC, Liu F, Jungbluth N, Kotte A, Hill KA, Mah AC, Korathu-Larson PA, Au JR, Izmirian S, Keir S, Nakamura BJ, Higa-McMillan CK, Cooper BR, Funaiole A, Dizon E, Hawkins EJ, Malte CA, Hagedorn HJ, Berger D, Frank A, Lott A, Achtmeyer CE, Mariano AJ, Saxon AJ, Wolitzky-Taylor K, Rawson R, Ries R, Roy-Byrne P, Craske M, Simmons D, Torrente C, Nathanson L, Carroll G, Smith JD, Brown K, Ramos K, Thornton N, Dishion TJ, Stormshak EA, Shaw DS, Wilson MN, Choy-Brown M, Tiderington E, Smith BT, Padgett DK, Rubin RM, Ray ML, Wandersman A, Lamont A, Hannah G, Alia KA, Hurford MO, Evans AC, Saldana L, Schaper H, Campbell M, Chamberlain P, Shapiro VB, Kim BKE, Fleming JL, LeBuffe PA, Landes SJ, Lewis CC, Rodriguez AL, Marriott BR, Comtois KA, Lewis CC, Stanick C, Weiner BJ, Halko H, Dorsey C. Proceedings of the 3rd Biennial Conference of the Society for Implementation Research Collaboration (SIRC) advancing efficient methodologies through community partnerships and team science : Seattle, WA, USA. 24-26 September 2015. Implement Sci. 2016 Jun 23;11 Suppl 1(Suppl 1):85. doi: 10.1186/s13012-016-0428-0. PMID: 27357964; PMCID: PMC4928139.	CONFERENCE

257	Emanuel EJ, Onwuteaka-Philipsen BD, Urwin JW, Cohen J. Attitudes and Practices of Euthanasia and Physician-Assisted Suicide in the United States, Canada, and Europe. JAMA. 2016 Jul 5;316(1):79-90. doi: 10.1001/jama.2016.8499. Erratum in: JAMA. 2016 Sep 27;316(12):1319. doi: 10.1001/jama.2016.12560. PMID: 27380345.	REVIEW

258	Tomás CC, Oliveira E, Sousa D, Uba-Chupel M, Furtado G, Rocha C, Teixeira A, Ferreira P, Alves C, Gisin S, Catarino E, Carvalho N, Coucelo T, Bonfim L, Silva C, Franco D, González JA, Jardim HG, Silva R, Baixinho CL, Presado MªH, Marques MªF, Cardoso ME, Cunha M, Mendes J, Xavier A, Galhardo A, Couto M, Frade JG, Nunes C, Mesquita JR, Nascimento MS, Gonçalves G, Castro C, Mártires A, Monteiro MªJ, Rainho C, Caballero FP, Monago FM, Guerrero JT, Monago RM, Trigo AP, Gutierrez ML, Milanés GM, Reina MG, Villanueva AG, Piñero AS, Aliseda IR, Ramirez FB, Ribeiro A, Quelhas A, Manso C, Caballero FP, Guerrero JT, Monago FM, Santos RB, Jimenez NR, Nuñez CG, Gomez IR, Fernandez MªJL, Marquez LA, Moreno AL, Huertas MªJT, Ramirez FB, Seabra D, Salvador MªC, Braga L, Parreira P, Salgueiro-Oliveira A, Arreguy-Sena C, Oliveira BF, Henriques MªA, Santos J, Lebre S, Marques A, Festas C, Rodrigues S, Ribeiro A, Lumini J, Figueiredo AG, Hernandez-Martinez FJ, Campi L, Quintana-Montesdeoca MªP, Jimenez-Diaz JF, Rodriguez-De-Vera BC, Parente A, Mata MªA, Pereira AMª, Fernandes A, Brás M, Pinto MªR, Parreira P, Basto ML, Rei AC, Mónico LM, Sousa G, Morna C, Freitas O, Freitas G, Jardim A, Vasconcelos R, Horta LG, Rosa RS, Kranz LF, Nugem RC, Siqueira MS, Bordin R, Kniess R, Lacerda JT, Guedes J, Machado I, Almeida S, Zilhão A, Alves H, Ribeiro Ó, Amaral AP, Santos A, Monteiro J, Rocha MªC, Cruz R, Amaral AP, Lourenço M, Rocha MªC, Cruz R, Antunes S, Mendonça V, Andrade I, Osório N, Valado A, Caseiro A, Gabriel A, Martins AC, Mendes F, Cabral L, Ferreira M, Gonçalves A, Luz TD, Luz L, Martins R, Morgado A, Vale-Dias ML, Porta-Nova R, Fleig TC, Reuter ÉM, Froemming MB, Guerreiro SL, Carvalho LL, Guedelha D, Coelho P, Pereira A, Calha A, Cordeiro R, Gonçalves A, Certo A, Galvão A, Mata MªA, Welter A, Pereira E, Ribeiro S, Kretzer M, Jiménez-Díaz JF, Jiménez-Rodríguez C, Hernández-Martínez FJ, Rodríguez-De-Vera BDC, Marques-Rodrigues A, Coelho P, Bernardes T, Pereira A, Sousa P, Filho JG, Nazario N, Kretzer M, Amaral O, Garrido A, Veiga N, Nunes C, Pedro AR, Pereira C, Almeia A, Fernandes HM, Vasconcelos C, Sousa N, Reis VM, Monteiro MJ, Mendes R, Pinto IC, Pires T, Gama J, Preto V, Silva N, Magalhães C, Martins M, Duarte M, Paúl C, Martín I, Pinheiro AA, Xavier S, Azevedo J, Bento E, Marques C, Marques M, Macedo A, Pereira AT, Almeida JP, Almeida A, Alves J, Sousa N, Saavedra F, Mendes R, Maia AS, Oliveira MT, Sousa AR, Ferreira PP, Lopes LS, Santiago EC, Monteiro S, Jesus Â, Colaço A, Carvalho A, Silva RP, Cruz A, Ferreira A, Marques C, Figueiredo JP, Paixão S, Ferreira A, Lopes C, Moreira F, Figueiredo JP, Ferreira A, Ribeiro D, Moreira F, Figueiredo JP, Paixão S, Fernandes T, Amado D, Leal J, Azevedo M, Ramalho S, Mangas C, Ribeiro J, Gonçalves R, Nunes AF, Tuna AR, Martins CR, Forte HD, Costa C, Tenedório JA, Santana P, Andrade JA, Pinto JL, Campofiorito C, Nunes S, Carmo A, Kaliniczenco A, Alves B, Mendes F, Jesus C, Fonseca F, Gehrke F, Albuquerque C, Batista R, Cunha M, Madureira A, Ribeiro O, Martins R, Madeira T, Peixoto-Plácido C, Santos N, Santos O, Bergland A, Bye A, Lopes C, Alarcão V, Goulão B, Mendonça N, Nicola P, Clara JG, Gomes J, Querido A, Tomás C, Carvalho D, Cordeiro M, Rosa MC, Marques A, Brandão D, Ribeiro Ó, Araújo L, Paúl C, Minghelli B, Richaud S, Mendes AL, Marta-Simões J, Trindade IA, Ferreira C, Carvalho T, Cunha M, Pinto-Gouveia J, Fernandes MC, Rosa RS, Nugem RC, Kranz LF, Siqueira MS, Bordin R, Martins AC, Medeiros A, Pimentel R, Fernandes A, Mendonça C, Andrade I, Andrade S, Menezes RL, Bravo R, Miranda M, Ugartemendia L, Tena JMª, Pérez-Caballero FL, Fuentes-Broto L, Rodríguez AB, Carmen B, Carneiro MA, Domingues JN, Paixão S, Figueiredo J, Nascimento VB, Jesus C, Mendes F, Gehrke F, Alves B, Azzalis L, Fonseca F, Martins AR, Nunes A, Jorge A, Veiga N, Amorim A, Silva A, Martinho L, Monteiro L, Silva R, Coelho C, Amaral O, Coelho I, Pereira C, Correia A, Rodrigues D, Marante N, Silva P, Carvalho S, Araujo AR, Ribeiro M, Coutinho P, Ventura S, Roque F, Calvo C, Reses M, Conde J, Ferreira A, Figueiredo J, Silva D, Seiça L, Soares R, Mourão R, Kraus T, Abreu AC, Padilha JM, Alves JM, Sousa P, Oliveira M, Sousa J, Novais S, Mendes F, Pinto J, Cruz J, Marques A, Duarte H, Dixe MDA, Sousa P, Cruz I, Bastos F, Pereira F, Carvalho FL, Oliveira TT, Raposo VR, Rainho C, Ribeiro JC, Barroso I, Rodrigues V, Neves C, Oliveira TC, Oliveira B, Morais MªC, Baylina P, Rodrigues R, Azeredo Z, Vicente C, Dias H, Sim-Sim M, Parreira P, Salgueiro-Oliveira A, Castilho A, Melo R, Graveto J, Gomes J, Vaquinhas M, Carvalho C, Mónico L, Brito N, Sarroeira C, Amendoeira J, Cunha F, Cândido A, Fernandes P, Silva HR, Silva E, Barroso I, Lapa L, Antunes C, Gonçalves A, Galvão A, Gomes MªJ, Escanciano SR, Freitas M, Parreira P, Marôco J, Fernandes AR, Cabral C, Alves S, Sousa P, Ferreira A, Príncipe F, Seppänen UM, Ferreira M, Carvalhais M, Silva M, Ferreira M, Silva J, Neves J, Costa D, Santos B, Duarte S, Marques S, Ramalho S, Mendes I, Louro C, Menino E, Dixe M, Dias SS, Cordeiro M, Tomás C, Querido A, Carvalho D, Gomes J, Valim FC, Costa JO, Bernardes LG, Prebianchi H, Rosa MC, Gonçalves N, Martins MM, Kurcgant P, Vieira A, Bento S, Deodato S, Rabiais I, Reis L, Torres A, Soares S, Ferreira M, Graça P, Leitão C, Abreu R, Bellém F, Almeida A, Ribeiro-Varandas E, Tavares A, Frade JG, Henriques C, Menino E, Louro C, Jordão C, Neco S, Morais C, Ferreira P, Silva CR, Brito A, Silva A, Duarte H, Dixe MDA, Sousa P, Postolache G, Oliveira R, Moreira I, Pedro L, Vicente S, Domingos S, Postolache O, Silva D, Filho JG, Nazario N, Kretzer M, Schneider D, Marques FM, Parreira P, Carvalho C, Mónico LM, Pinto C, Vicente S, Breda SJ, Gomes JH, Melo R, Parreira P, Salgueiro A, Graveto J, Vaquinhas M, Castilho A, Jesus Â, Duarte N, Lopes JC, Nunes H, Cruz A, Salgueiro-Oliveira A,	CONFERENCE

	Parreira P, Basto ML, Braga LM, Ferreira A, Araújo B, Alves JM, Ferreira M, Carvalhais M, Silva M, Novais S, Sousa AS, Ferrito C, Ferreira PL, Rodrigues A, Ferreira M, Oliveira I, Ferreira M, Neves J, Costa D, Duarte S, Silva J, Santos B, Martins C, Macedo AP, Araújo O, Augusto C, Braga F, Gomes L, Silva MA, Rosário R, Pimenta L, Carreira D, Teles P, Barros T, Tomás C, Querido A, Carvalho D, Gomes J, Cordeiro M, Carvalho D, Querido A, Tomás C, Gomes J, Cordeiro M, Jácome C, Marques A, Capelas S, Hall A, Alves D, Lousada M, Loureiro MªH, Camarneiro A, Silva M, Mendes A, Pedreiro A, G.Silva A, Coelho ES, Melo F, Ribeiro F, Torres R, Costa R, Pinho T, Jácome C, Marques A, Cruz B, Seabra D, Carreiras D, Ventura M, Cruz X, Brooks D, Marques A, Pinto MR, Parreira P, Lima-Basto M, Neves M, Mónico LM, Bizarro C, Cunha M, Galhardo A, Margarida C, Amorim AP, Silva E, Cruz S, Padilha JM, Valente J, Guerrero JT, Caballero FP, Santos RB, Gonzalez EP, Monago FM, Ugalde LU, Vélez MM, Tena MJ, Guerrero JT, Bravo R, Pérez-Caballero FL, Becerra IA, Agudelo MªE, Acedo G, Bajo R, Malheiro I, Gaspar F, Barros L, Furtado G, Uba-Chupel M, Marques M, Rama L, Braga M, Ferreira JP, Teixeira AMª, Cruz J, Barbosa T, Simões Â, Coelho L, Rodrigues A, Jiménez-Díaz JF, Martinez-Hernandez F, Rodriguez-De-Vera B, Ferreira P, Rodrigues A, Ramalho A, Petrica J, Mendes P, Serrano J, Santo I, Rosado A, Mendonça P, Freitas K, Ferreira D, Brito A, Fernandes R, Gomes S, Moreira F, Pinho C, Oliveira R, Oliveira AI, Mendonça P, Casimiro AP, Martins P, Silva I, Evangelista D, Leitão C, Velosa F, Carecho N, Coelho L, Menino E, Dixe A, Catarino H, Soares F, Gama E, Gordo C, Moreira E, Midões C, Santos M, Machado S, Oliveira VP, Santos M, Querido A, Dixe A, Marques R, Charepe Z, Antunes A, Santos S, Rosa MC, Rosa MC, Marques SF, Minghelli B, CaroMinghelli E, Luís MªJ, Brandão T, Mendes P, Marinho D, Petrica J, Monteiro D, Paulo R, Serrano J, Santo I, Monteiro L, Ramalho F, Santos-Rocha R, Morgado S, Bento T, Sousa G, Freitas O, Silva I, Freitas G, Morna C, Vasconcelos R, Azevedo T, Soares S, Pisco J, Ferreira PP, Olszewer EO, Oliveira MT, Sousa AR, Maia AS, Oliveira ST, Santos E, Oliveira AI, Maia C, Moreira F, Santos J, Mendes MF, Oliveira RF, Pinho C, Barreira E, Pereira A, Vaz JA, Novo A, Silva LD, Maia B, Ferreira E, Pires F, Andrade R, Camarinha L, Silva LD, Maia B, Ferreira E, Pires F, Andrade R, Camarinha L, César AF, Poço M, Ventura D, Loura R, Gomes P, Gomes C, Silva C, Melo E, Lindo J, Domingos J, Mendes Z, Poeta S, Carvalho T, Tomás C, Catarino H, Dixe MªA, Ramalho A, Rosado A, Mendes P, Paulo R, Garcia I, Petrica J, Rodrigues S, Meneses R, Afonso C, Faria L, Seixas A, Cordeiro M, Granjo P, Gomes JC, Souza NR, Furtado GE, Rocha SV, Silva P, Carvalho J, Morais MA, Santos S, Lebre P, Antunes A, Calha A, Xavier A, Cunha M, Pinto-Gouveia J, Alencar L, Cunha M, Madureira A, Cardoso I, Galhardo A, Daniel F, Rodrigues V, Luz L, Luz T, Ramos MR, Medeiros DC, Carmo BM, Seabra A, Padez C, Silva MC, Rodrigues A, Coelho P, Coelho A, Caminha M, Matheus F, Mendes E, Correia J, Kretzer M, Hernandez- Martinez FJ, Jimenez-Diaz JF, Rodriguez-De-Vera BC, Jimenez-Rodriguez C, Armas- Gonzalez Y, Rodrigues C, Pedroso R, Apolinário-Hagen J, Vehreschild V, Veloso M, Magalhães C, Cabral I, Ferraz M, Nave F, Costa E, Matos F, Pacheco J, Dias A, Pereira C, Duarte J, Cunha M, Silva D, Mónico LM, Alferes VR, Brêda MªSJ, Carvalho C, Parreira PM, Morais MªC, Ferreira P, Pimenta R, Boavida J, Pinto IC, Pires T, Silva C, Ribeiro M, Viega-Branco M, Pereira F, Pereira AMª, Almeida FM, Estevez GL, Ribeiro S, Kretzer MR, João PV, Nogueira P, Novais S, Pereira A, Carneiro L, Mota M, Cruz R, Santiago L, Fontes-Ribeiro C, Furtado G, Rocha SV, Coutinho AP, Neto JS, Vasconcelos LR, Souza NR, Dantas E, Dinis A, Carvalho S, Castilho P, Pinto-Gouveia J, Sarreira-Santos A, Figueiredo A, Medeiros-Garcia L, Seabra P, Rodrigues R, Morais MªC, Fernandes PO, Santiago C, Figueiredo MªH, Basto ML, Guimarães T, Coelho A, Graça A, Silva AM, Fonseca AR, Vale-Dias L, Minas B, Franco-Borges G, Simões C, Santos S, Serra A, Matos M, Jesus L, Tavares AS, Almeida A, Leitão C, Varandas E, Abreu R, Bellém F, Trindade IA, Ferreira C, Pinto-Gouveia J, Marta-Simões J, Amaral O, Miranda C, Guimarães P, Gonçalves R, Veiga N, Pereira C, Fleig TC, San-Martin EA, Goulart CL, Schneiders PB, Miranda NF, Carvalho LL, Silva AG, Topa J, Nogueira C, Neves S, Ventura R, Nazaré C, Brandão D, Freitas A, Ribeiro Ó, Paúl C, Mercê C, Branco M, Almeida P, Nascimento D, Pereira J, Catela D, Rafael H, Reis AC, Mendes A, Valente AR, Lousada M, Sousa D, Baltazar AL, Loureiro MªH, Oliveira A, Aparício J, Marques A, Marques A, Oliveira A, Neves J, Ayoub R, Sousa L, Marques-Vieira C, Severino S, José H, Cadorio I, Lousada M, Cunha M, Andrade D, Galhardo A, Couto M, Mendes F, Domingues C, Schukg S, Abrantes AM, Gonçalves AC, Sales T, Teixo R, Silva R, Estrela J, Laranjo M, Casalta-Lopes J, Rocha C, Simões PC, Sarmento-Ribeiro AB, Botelho MªF, Rosa MS, Fonseca V, Colaço D, Neves V, Jesus C, Hesse C, Rocha C, Osório N, Valado A, Caseiro A, Gabriel A, Svensson L, Mendes F, Siba WA, Pereira C, Tomaz J, Carvalho T, Pinto-Gouveia J, Cunha M, Duarte D, Lopes NV, Fonseca- Pinto R, Duarte D, Lopes NV, Fonseca-Pinto R, Martins AC, Brandão P, Martins L, Cardoso M, Morais N, Cruz J, Alves N, Faria P, Mateus A, Morouço P, Alves N, Ferreira N, Mateus A, Faria P, Morouço P, Malheiro I, Gaspar F, Barros L, Parreira P, Cardoso A, Mónico L, Carvalho C, Lopes A, Salgueiro-Oliveira A, Seixas A, Soares V, Dias T, Vardasca R, Gabriel J, Rodrigues S, Paredes H, Reis A, Marinho S, Filipe V, Lains J, Barroso J, Da Motta C, Carvalho CB, Pinto- Gouveia J, Peixoto E, Gomes AA, Costa V, Couto D, Marques DR, Leitão JA, Tavares J, Azevedo MH, Silva CF, Freitas J, Parreira P, Marôco J, Garcia-Gordillo MA, Collado-Mateo D,	

	Chen G, Iezzi A, Sala JA, Parraça JA, Gusi N, Sousa J, Marques M, Jardim J, Pereira A, Simões S, Cunha M, Sardo P, Guedes J, Lindo J, Machado P, Melo E, Carvalho CB, Benevides J, Sousa M, Cabral J, Da Motta C, Pereira AT, Xavier S, Azevedo J, Bento E, Marques C, Carvalho R, Marques M, Macedo A, Silva AM, Alves J, Gomes AA, Marques DR, Azevedo MªH, Silva C, Mendes A, Lee HD, Spolaôr N, Oliva JT, Chung WF, Fonseca-Pinto R, Bairros K, Silva CD, Souza CA, Schroeder SS, Araújo E, Monteiro H, Costa R, Dias SS, Torgal J, Henriques CG, Santos L, Caceiro EF, Ramalho SA, Oliveira R, Afreixo V, Santos J, Mota P, Cruz A, Pimentel F, Marques R, Dixe MªA, Querido A, Sousa P, Benevides J, Da Motta C, Sousa M, Caldeira SN, Carvalho CB, Querido A, Tomás C, Carvalho D, Gomes J, Cordeiro M, Costa JO, Valim FC, Ribeiro LC, Charepe Z, Querido A, Figueiredo MªH, Aquino PS, Ribeiro SG, Pinheiro AB, Lessa PA, Oliveira MF, Brito LS, Pinto ÍN, Furtado AS, Castro RB, Aquino CQ, Martins ES, Pinheiro AB, Aquino PS, Oliveira LL, Pinheiro PC, Sousa CR, Freitas VA, Silva TM, Lima AS, Aquino CQ, Andrade KV, Oliveira CA, Vidal EF, Ganho-Ávila A, Moura-Ramos M, Gonçalves Ó, Almeida J, Silva A, Brito I, Amado J, Rodrigo A, Santos S, Gomes F, Rosa MC, Marques SF, Luís S, Cavalheiro L, Ferreira P, Gonçalves R, Lopes RS, Cavalheiro L, Ferreira P, Gonçalves R, Fiorin BH, Santos MS, Oliveira ES, Moreira RL, Oliveira EA, Filho BL, Palmeira L, Garcia T, Pinto-Gouveia J, Cunha M, Cardoso S, Palmeira L, Cunha M, Pinto-Gouveia J, Marta-Simões J, Mendes AL, Trindade IA, Oliveira S, Ferreira C, Mendes AL, Marta-Simões J, Trindade IA, Ferreira C, Nave F, Campos M, Gaudêncio I, Martins F, Ferreira L, Lopes N, Fonseca-Pinto R, Rodrigues R, Azeredo Z, Vicente C, Silva J, Sousa P, Marques R, Mendes I, Rodrigues R, Azeredo Z, Vicente C, Vardasca R, Marques AR, Seixas A, Carvalho R, Gabriel J, Ferreira PP, Oliveira MT, Sousa AR, Maia AS, Oliveira ST, Costa PO, Silva MM, Arreguy-Sena C, Alvarenga-Martins N, Pinto PF, Oliveira DC, Parreira PD, Gomes AT, Braga LM, Araújo O, Lage I, Cabrita J, Teixeira L, Marques R, Dixe MªA, Querido A, Sousa P, Silva S, Cordeiro E, Pimentel J, Ferro-Lebres V, Souza JA, Tavares M, Dixe MªA, Sousa P, Passadouro R, Peralta T, Ferreira C, Lourenço G, Serrano J, Petrica J, Paulo R, Honório S, Mendes P, Simões A, Carvalho L, Pereira A, Silva S, Sousa P, Padilha JM, Figueiredo D, Valente C, Marques A, Ribas P, Sousa J, Brandão F, Sousa C, Martins M, Sousa P, Marques R, Mendes F, Fernandes R, Martins E, Magalhães C, Araújo P, Grande C, Mata MªA, Vieitez JG, Bianchini B, Nazario N, Filho JG, Kretzer M, Costa T, Almeida A, Baffour G, Almeida A, Costa T, Baffour G, Azeredo Z, Laranjeira C, Guerra M, Barbeiro AP, Ferreira R, Lopes S, Nunes L, Mendes A, Martins J, Schneider D, Kretzer M, Magajewski F, Soares C, Marques A, Batista M, Castuera RJ, Mesquita H, Faustino A, Santos J, Honório S, Vizzotto BP, Frigo L, Pivetta HF, Sardo D, Martins C, Abreu W, Figueiredo MªC, Batista M, Jimenez-Castuera R, Petrica J, Serrano J, Honório S, Paulo R, Mendes P, Sousa P, Marques R, Faustino A, Silveira P, Serrano J, Paulo R, Mendes P, Honório S, Oliveira C, Bastos F, Cruz I, Rodriguez CK, Kretzer MR, Nazário NO, Cruz P, Vaz DC, Ruben RB, Avelelas F, Silva S, Campos MªJ, Almeida M, Gonçalves L, Antunes L, Sardo P, Guedes J, Simões J, Machado P, Melo E, Cardoso S, Santos O, Nunes C, Loureiro I, Santos F, Alves G, Soar C, Marsi TO, Silva E, Pedrosa D, Leça A, Silva D, Galvão A, Gomes M, Fernandes P, Noné A, Combadão J, Ramalhete C, Figueiredo P, Caeiro P, Fontana KC, Lacerda JT, Machado PO, Borges R, Barbosa F, Sá D, Brunhoso G, Aparício G, Carvalho A, Garcia AP, Fernandes PO, Santos A, Veiga N, Brás C, Carvalho I, Batalha J, Glória M, Bexiga F, Coelho I, Amaral O, Pereira C, Pinho C, Paraíso N, Oliveira AI, Lima CF, Dias AP, Silva P, Espada M, Marques M, Pereira A, Pereira AMª, Veiga-Branco Mª, Pereira F, Ribeiro M, Lima V, Oliveira AI, Pinho C, Cruz G, Oliveira RF, Barreiros L, Moreira F, Camarneiro A, Loureiro MªH, Silva M, Duarte C, Jesus Â, Cruz A, Mota M, Novais S, Nogueira P, Pereira A, Carneiro L, João PV, Lima TM, Salgueiro-Oliveira A, Vaquinhas M, Parreira P, Melo R, Graveto J, Castilho A, Gomes JH, Medina MS, Blanco VG, Santos O, Lopes E, Virgolino A, Dinis A, Ambrósio S, Almeida I, Marques T, Heitor MªJ, Garcia-Gordillo MA, Collado-Mateo D, Olivares PR, Parraça JA, Sala JA, Castilho A, Graveto J, Parreira P, Oliveira A, Gomes JH, Melo R, Vaquinhas M, Cheio M, Cruz A, Pereira OR, Pinto S, Oliveira A, Manso MC, Sousa C, Vinha AF, Machado MªM, Vieira M, Fernandes B, Tomás T, Quirino D, Desouzart G, Matos R, Bordini M, Mouroço P, Matos AR, Serapioni M, Guimarães T, Fonseca V, Costa A, Ribeiro J, Lobato J, Martin IZ, Björklund A, Tavares AI, Ferreira P, Passadouro R, Morgado S, Tavares N, Valente J, Martins AC, Araújo P, Fernandes R, Mendes F, Magalhães C, Martins E, Mendes P, Paulo R, Faustino A, Mesquita H, Honório S, Batista M, Lacerda JT, Ortiga AB, Calvo MªC, Natal S, Pereira M, Ferreira M, Prata AR, Nelas P, Duarte J, Carneiro J, Oliveira AI, Pinho C, Couto C, Oliveira RF, Moreira F, Maia AS, Oliveira MT, Sousa AR, Ferreira PP, Souza GM, Almada LF, Conceição MA, Santiago EC, Rodrigues S, Domingues G, Ferreira I, Faria L, Seixas A, Costa AR, Jesus Â, Cardoso A, Meireles A, Colaço A, Cruz A, Vieira VL, Vincha KR, Cervato-Mancuso AMª, Faria M, Reis C, Cova MP, Ascenso RT, Almeida HA, Oliveira EG, Santana M, Pereira R, Oliveira EG, Almeida HA, Ascenso RT, Jesus R, Tapadas R, Tim-Tim C, Cezanne C, Lagoa M, Dias SS, Torgal J, Lopes J, Almeida H, Amado S, Carrão L, Cunha M, Saboga-Nunes L, Albuquerque C, Ribeiro O, Oliveira S, Morais MªC, Martins E, Mendes F, Fernandes R, Magalhães C, Araújo P, Pedro AR, Amaral O, Escoval A, Assunção V, Luís H, Luís L, Apolinário-Hagen J, Vehreschild V, Fotschl U, Lirk G, Martins AC, Andrade I,	

	Mendes F, Mendonça V, Antunes S, Andrade I, Osório N, Valado A, Caseiro A, Gabriel A, Martins AC, Mendes F, Silva PA, Mónico LM, Parreira PM, Carvalho C, Carvalho C, Parreira PM, Mónico LM, Ruivo J, Silva V, Sousa P, Padilha JM, Ferraz V, Aparício G, Duarte J, Vasconcelos C, Almeida A, Neves J, Correia T, Amorim H, Mendes R, Saboga- Nunes L, Cunha M, Albuquerque C, Pereira ES, Santos LS, Reis AS, Silva HR, Rombo J, Fernandes JC, Fernandes P, Ribeiro J, Mangas C, Freire A, Silva S, Francisco I, Oliveira A, Catarino H, Dixe MªA, Louro MªC, Lopes S, Dixe A, Dixe MªA, Menino E, Catarino H, Soares F, Oliveira AP, Gordo S, Kraus T, Tomás C, Queirós P, Rodrigues T, Sousa P, Frade JG, Lobão C, Moura CB, Dreyer LC, Meneghetti V, Cabral PP, Pinto F, Sousa P, Esteves MªR, Galvão S, Tytgat I, Andrade I, Osório N, Valado A, Caseiro A, Gabriel A, Martins AC, Mendes F, Casas-Novas M, Bernardo H, Andrade I, Sousa G, Sousa AP, Rocha C, Belo P, Osório N, Valado A, Caseiro A, Gabriel A, Martins AC, Mendes F, Martins F, Pulido-Fuentes M, Barroso I, Cabral G, Monteiro MJ, Rainho C, Prado A, Carvalho YM, Campos M, Moreira L, Ferreira J, Teixeira A, Rama L, Campos M, Moreira L, Ferreira J, Teixeira A, Rama L. Proceedings of the 3rd IPLeiria's International Health Congress : Leiria, Portugal. 6-7 May 2016. BMC Health Serv Res. 2016 Jul 6;16 Suppl 3(Suppl 3):200. doi: 10.1186/s12913-016-1423-5. PMID: 27409075; PMCID: PMC4943498.	
259	Chen HP, Huang BY, Yi TW, Deng YT, Liu J, Zhang J, Wang YQ, Zhang ZY, Jiang Y. Attitudes of Chinese Oncology Physicians Toward Death with Dignity. J Palliat Med. 2016 Aug;19(8):874-8. doi: 10.1089/jpm.2015.0344. Epub 2016 Mar 29. PMID: 27022774; PMCID: PMC4982948.	UNFOCUSED
260	Paulus DJ, Bakhshaie J, Garza M, Ochoa-Perez M, Mayorga NA, Bogiaizian D, Robles Z, Lu Q, Ditre J, Vowles K, Schmidt NB, Zvolensky MJ. Pain severity and emotion dysregulation among Latinos in a community health care setting: relations to mental health. Gen Hosp Psychiatry. 2016 Sep-Oct;42:41-8. doi: 10.1016/j.genhosppsych.2016.07.002. Epub 2016 Jul 9. PMID: 27638971; PMCID: PMC11846064.	UNFOCUSED
261	Gottlieb A, Menter A, Armstrong A, Ocampo C, Gu Y, Teixeira HD. Adalimumab Treatment in Women With Moderate-to-Severe Hidradenitis Suppurativa from the Placebo-Controlled Portion of a Phase 2, Randomized, Double-Blind Study. J Drugs Dermatol. 2016 Oct 1;15(10):1192-1196. PMID: 27741335.	UNFOCUSED
262	Park SA, Chung SH, Lee Y. Factors Associated with Suicide Risk in Advanced Cancer Patients: A Cross-Sectional Study. Asian Pac J Cancer Prev. 2016 Nov 1;17(11):4831-4836. doi: 10.22034/APJCP.2016.17.11.4831. PMID: 28030907; PMCID: PMC5454682.	INCLUDED
263	Fezai M, Slaymi C, Ben-Attia M, Lang F, Jemaà M. Purified Lesser weever fish venom (Trachinus vipera) induces eryptosis, apoptosis and cell cycle arrest. Sci Rep. 2016 Dec 20;6:39288. doi: 10.1038/srep39288. PMID: 27995979; PMCID: PMC5171788	UNFOCUSED
264	Schmidt KW. Sterbehilfe in (Spiel-)Filmen – Was wird (nicht) gezeigt? [Assisted suicide in the movies - what is (not) shown?]. Bundesgesundheitsblatt Gesundheitsforschung Gesundheitsschutz. 2017 Jan;60(1):99-107. German. doi: 10.1007/s00103-016-2474-9. PMID: 27896390.	UNFOCUSED
265	Fezai M, Slaymi C, Ben-Attia M, Kroemer G, Lang F, Jemaà M. Inhibition of Colon Carcinoma Cell Migration Following Treatment with Purified Venom from Lesser Weever Fish (Trachinus Vipera). Cell Physiol Biochem. 2017;41(6):2279-2288. doi: 10.1159/000475646. Epub 2017 Apr 26. PMID: 28456793.	UNFOCUSED
266	Tormey WP. Oxycodone in Palliative Care-Art and Empathy Still Have a Place. J Palliat Care. 2017 Jan;32(1):40-42. doi: 10.1177/0825859717705929. PMID: 28662625.	UNFOCUSED
267	Appart A, Lange AK, Sievert I, Bihain F, Tordeurs D. Le trouble de l'adaptation et le DSM-5 : une revue de la littérature [Adjustment disorder and DSM-A review]. Encephale. 2017 Feb;43(1):41-46. French. doi: 10.1016/j.encep.2015.06.007. Epub 2016 May 20. PMID: 27216596.	UNFOCUSED
268	Robinson S, Kissane DW, Brooker J, Hempton C, Burney S. The Relationship Between Poor Quality of Life and Desire to Hasten Death: A Multiple Mediation Model Examining the Contributions of Depression, Demoralization, Loss of Control, and Low Self-worth. J Pain Symptom Manage. 2017 Feb;53(2):243-249. doi: 10.1016/j.jpainsymman.2016.08.013. Epub 2016 Oct 12. PMID: 27744017.	LUMPING
269	Racine M, Sánchez-Rodríguez E, Gálan S, Tomé-Pires C, Solé E, Jensen MP, Nielson WR, Miró J, Moulin DE, Choinière M. Factors Associated with Suicidal Ideation in Patients with Chronic Non-Cancer Pain. Pain Med. 2017 Feb 1;18(2):283-293. doi: 10.1093/pm/pnw115. PMID: 28204732.	UNFOCUSED

270	Forstner AJ, Hecker J, Hofmann A, Maaser A, Reinbold CS, Mühleisen TW, Leber M, Strohmaier J, Degenhardt F, Treutlein J, Mattheisen M, Schumacher J, Streit F, Meier S, Herms S, Hoffmann P, Lacour A, Witt SH, Reif A, Müller-Myhsok B, Lucae S, Maier W, Schwarz M, Vedder H, Kammerer-Ciernioch J, Pfennig A, Bauer M, Hautzinger M, Moebus S, Schenk LM, Fischer SB, Sivalingam S, Czerski PM, Hauser J, Lissowska J, Szeszenia-Dabrowska N, Brennan P, McKay JD, Wright A, Mitchell PB, Fullerton JM, Schofield PR, Montgomery GW, Medland SE, Gordon SD, Martin NG, Krasnov V, Chuchalin A, Babadjanova G, Pantelejeva G, Abramova LI, Tiganov AS, Polonikov A, Khusnutdinova E, Alda M, Cruceanu C, Rouleau GA, Turecki G, Laprise C, Rivas F, Mayoral F, Kogevinas M, Grigoroiu-Serbanescu M, Becker T, Schulze TG, Rietschel M, Cichon S, Fier H, Nöthen MM. Identification of shared risk loci and pathways for bipolar disorder and schizophrenia. PLoS One. 2017 Feb 6;12(2):e0171595. doi: 10.1371/journal.pone.0171595. PMID: 28166306; PMCID: PMC5293228.	UNFOCUSED
271	Zhong BL, Li SH, Lv SY, Tian SL, Liu ZD, Li XB, Zhuang HQ, Tao R, Zhang W, Zhuo CJ. Suicidal ideation among Chinese cancer inpatients of general hospitals: prevalence and correlates. Oncotarget. 2017 Apr 11;8(15):25141-25150. doi: 10.18632/oncotarget.15350. PMID: 28212579; PMCID: PMC5421916.	LUMPING
272	Tokish JM, Kissenberth MJ, Tolan SJ, Salim TI, Tadlock J, Kellam T, Long CD, Crawford A, Lonergan KT, Hawkins RJ, Shanley E. Resilience correlates with outcomes after total shoulder arthroplasty. J Shoulder Elbow Surg. 2017 May;26(5):752-756. doi: 10.1016/j.jse.2016.12.070. Epub 2017 Feb 10. PMID: 28190668.	UNFOCUSED
273	Shim E-, Song YW, Park SH, Lee KM, Go DJ, Hahm BJ. Examining the Relationship Between Pain Catastrophizing and Suicide Risk in Patients with Rheumatic Disease: the Mediating Role of Depression, Perceived Social Support, and Perceived Burdensomeness. Int J Behav Med. 2017 Aug;24(4):501-512. doi: 10.1007/s12529-017-9648-1. PMID: 28299624.	UNFOCUSED
274	Bar Sela G, Bentur N, Rei Koren Z, Schultz M. [DO WE HAVE MEDICAL MEASURES THAT ATTEST TO THE EFFECT OF SPIRITUAL CARE IN TIME OF ILLNESS?]. Harefuah. 2017 Aug;156(8):502-506. Hebrew. PMID: 28853526.	UNFOCUSED
275	Kim J, Grobelna A. Nabilone for Chronic Pain Management: A Review of Clinical Effectiveness and Guidelines [Internet]. Ottawa (ON): Canadian Agency for Drugs and Technologies in Health; 2017 Aug 9. PMID: 29949325.	REVIEW
276	Aberle D, Wu SE, Oklu R, Erinjeri J, Deipolyi AR. Association Between Allergies and Psychiatric Disorders in Patients Undergoing Invasive Procedures. Psychosomatics. 2017 Sep-Oct;58(5):490-495. doi: 10.1016/j.psym.2017.03.015. Epub 2017 Mar 28. PMID: 28527521.	UNFOCUSED
277	Lin YH, Kao CC, Wu SF, Hung SL, Yang HY, Tung HY. Risk factors of post- traumatic stress symptoms in patients with cancer. J Clin Nurs. 2017 Oct;26(19-20):3137-3143. doi: 10.1111/jocn.13662. Epub 2017 Apr 17. PMID: 27875034.	UNFOCUSED
278	Braverman DW, Marcus BS, Wakim PG, Mercurio MR, Kopf GS. Health Care Professionals' Attitudes About Physician-Assisted Death: An Analysis of Their Justifications and the Roles of Terminology and Patient Competency. J Pain Symptom Manage. 2017 Oct;54(4):538-545.e3. doi: 10.1016/j.jpainsymman.2017.07.024. Epub 2017 Jul 15. PMID: 28716621; PMCID: PMC5632116.	UNFOCUSED
279	Chakraborty R, El-Jawahri AR, Litzow MR, Syrjala KL, Parnes AD, Hashmi SK. A systematic review of religious beliefs about major end-of-life issues in the five major world religions. Palliat Support Care. 2017 Oct;15(5):609-622. doi: 10.1017/S1478951516001061. PMID: 28901283; PMCID: PMC5865598.	REVIEW
280	Granek L, Nakash O, Ariad S, Chen W, Birenstock-Cohen S, Shapira S, Ben- David M. From will to live to will to die: oncologists, nurses, and social workers identification of suicidality in cancer patients. Support Care Cancer. 2017 Dec;25(12):3691-3702. doi: 10.1007/s00520-017-3795-4. Epub 2017 Jun 26. PMID: 28653106.	UNFOCUSED
281	Cheung G, Douwes G, Sundram F. Late-Life Suicide in Terminal Cancer: A Rational Act or Underdiagnosed Depression? J Pain Symptom Manage. 2017 Dec;54(6):835-842. doi: 10.1016/j.jpainsymman.2017.05.004. Epub 2017 Aug 12. PMID: 28807701.	UNFOCUSED

282	Spillane A, Larkin C, Corcoran P, Matvienko-Sikar K, Riordan F, Arensman E. Physical and psychosomatic health outcomes in people bereaved by suicide compared to people bereaved by other modes of death: a systematic review. BMC Public Health. 2017 Dec 12;17(1):939. doi: 10.1186/s12889-017-4930-3. PMID: 29228916; PMCID: PMC5725957.	REVIEW
283	Slater H, Campbell JM, Stinson JN, Burley MM, Briggs AM. End User and Implementer Experiences of mHealth Technologies for Noncommunicable Chronic Disease Management in Young Adults: Systematic Review. J Med Internet Res. 2017 Dec 12;19(12):e406. doi: 10.2196/jmir.8888. PMID: 29233804; PMCID: PMC5743925.	REVIEW
284	Balboni MJ, Sullivan A, Smith PT, Zaidi D, Mitchell C, Tulsky JA, Sulmasy DP, VanderWeele TJ, Balboni TA. The Views of Clergy Regarding Ethical Controversies in Care at the End of Life. J Pain Symptom Manage. 2018 Jan;55(1):65-74.e9. doi: 10.1016/j.jpainsymman.2017.05.009. Epub 2017 Aug 15. PMID: 28818632; PMCID: PMC5735011.	UNFOCUSED
285	Ho RTH, Fong TCT, Yip PSF. Perceived stress moderates the effects of a randomized trial of dance movement therapy on diurnal cortisol slopes in breast cancer patients. Psychoneuroendocrinology. 2018 Jan;87:119-126. doi: 10.1016/j.psyneuen.2017.10.012. Epub 2017 Oct 18. PMID: 29059542.	UNFOCUSED
286	Mori M, Fujimori M, Hamano J, Naito AS, Morita T. Which Physicians' Behaviors on Death Pronouncement Affect Family-Perceived Physician Compassion? A Randomized, Scripted, Video-Vignette Study. J Pain Symptom Manage. 2018 Feb;55(2):189-197.e4. doi: 10.1016/j.jpainsymman.2017.08.029. Epub 2017 Sep 6. PMID: 28887269.	UNFOCUSED
287	Adewumi AD, Hollingworth SA, Maravilla JC, Connor JP, Alati R. Prescribed Dose of Opioids and Overdose: A Systematic Review and Meta-Analysis of Unintentional Prescription Opioid Overdose. CNS Drugs. 2018 Feb;32(2):101-116. doi: 10.1007/s40263-018-0499-3. PMID: 29498021.	REVIEW
288	Roberts SE, John A, Kandalama U, Williams JG, Lyons RA, Lloyd K. Suicide following acute admissions for physical illnesses across England and Wales. Psychol Med. 2018 Mar;48(4):578-591. doi: 10.1017/S0033291717001787. Epub 2017 Jul 17. PMID: 28714426; PMCID: PMC5964467.	UNFOCUSED
289	LeRoy AS, Lu Q, Zvolensky MJ, Ramirez J, Fagundes CP. Anxiety sensitivity moderates the painful effects of feeling burdensome to others. Cogn Behav Ther. 2018 Mar;47(2):126-138. doi: 10.1080/16506073.2017.1357749. Epub 2017 Aug 9. PMID: 28791887; PMCID: PMC6049813.	UNFOCUSED
290	Kessoku T, Kusakabe A, Matsuura T, Honda Y, Yoshimi A, Goto A, Yoshida H, Sukegawa A, Hata C, Saito Y, Miyashita Y, Yashiro R, Komori T, Arai S, Nakajima A, Ichikawa Y. [Fentanyl Citrate Sublingual Tablets Were Effective in Relieving Symptoms of Akathisia - A Case Report]. Gan To Kagaku Ryoho. 2018 Mar;45(Suppl 1):89-91. Japanese. PMID: 29650885.	CASE
291	Alemayehu M, Deyessa N, Medihin G, Fekadu A. A descriptive analysis of depression and pain complaints among patients with cancer in a low income country. PLoS One. 2018 Mar 7;13(3):e0193713. doi: 10.1371/journal.pone.0193713. PMID: 29513716; PMCID: PMC5841758.	UNFOCUSED
292	Gamondi C, Pott M, Preston N, Payne S. Family Caregivers' Reflections on Experiences of Assisted Suicide in Switzerland: A Qualitative Interview Study. J Pain Symptom Manage. 2018 Apr;55(4):1085-1094. doi: 10.1016/j.jpainsymman.2017.12.482. Epub 2017 Dec 27. PMID: 29288877.	UNFOCUSED
293	Verkissen MN, Houttekier D, Cohen J, Schots R, Chambaere K, Deliens L. End- of-life decision-making across cancer types: results from a nationwide retrospective survey among treating physicians. Br J Cancer. 2018 May;118(10):1369-1376. doi: 10.1038/s41416-018-0070-5. Epub 2018 Mar 29. PMID: 29593337; PMCID: PMC5959875.	UNFOCUSED

294	Wray NR, Ripke S, Mattheisen M, Trzaskowski M, Byrne EM, Abdellaoui A, Adams MJ, Agerbo E, Air TM, Andlauer TMF, Bacanu SA, Bækvad-Hansen M, Beekman AFT, Bigdeli TB, Binder EB, Blackwood DRH, Bryois J, Buttenschøn HN, Bybjerg- Grauholm J, Cai N, Castelao E, Christensen JH, Clarke TK, Coleman JIR, Colodro- Conde L, Couvy-Duchesne B, Craddock N, Crawford GE, Crowley CA, Dashti HS, Davies G, Deary IJ, Degenhardt F, Derks EM, Direk N, Dolan CV, Dunn EC, Eley TC, Eriksson N, Escott-Price V, Kiadeh FHF, Finucane HK, Forstner AJ, Frank J, Gaspar HA, Gill M, Giusti-Rodríguez P, Goes FS, Gordon SD, Grove J, Hall LS, Hannon E, Hansen CS, Hansen TF, Herms S, Hickie IB, Hoffmann P, Homuth G, Horn C, Hottenga JJ, Hougaard DM, Hu M, Hyde CL, Ising M, Jansen R, Jin F, Jorgenson E, Knowles JA, Kohane IS, Kraft J, Kretzschmar WW, Krogh J, Kutalik Z, Lane JM, Li Y, Li Y, Lind PA, Liu X, Lu L, MacIntyre DJ, MacKinnon DF, Maier RM, Maier W, Marchini J, Mbarek H, McGrath P, McGuffin P, Medland SE, Mehta D, Middeldorp CM, Mihailov E, Milaneschi Y, Milani L, Mill J, Mondimore FM, Montgomery GW, Mostafavi S, Mullins N, Nauck M, Ng B, Nivard MG, Nyholt DR, O'Reilly PF, Oskarsson H, Owen MJ, Painter JN, Pedersen CB, Pedersen MG, Peterson RE, Pettersson E, Peyrot WJ, Pistis G, Posthuma D, Purcell SM, Quiroz JA, Qvist P, Rice JP, Riley BP, Rivera M, Saeed Mirza S, Saxena R, Schoevers R, Schulte EC, Shen L, Shi J, Shyn SI, Sigurdsson E, Sinnamon GBC, Smit JH, Smith DJ, Stefansson H, Steinberg S, Stockmeier CA, Streit F, Strohmaier J, Tansey KE, Teismann H, Teumer A, Thompson W, Thomson PA, Thorgeirsson TE, Tian C, Traylor M, Treutlein J, Trubetskoy V, Uitterlinden AG, Umbricht D, Van der Auwera S, van Hemert AM, Viktorin A, Visscher PM, Wang Y, Webb BT, Weinsheimer SM, Wellmann J, Willemsen G, Witt SH, Wu Y, Xi HS, Yang J, Zhang F; eQTLGen; 23andMe; Arolt V, Baune BT, Berger K, Boomsma DI, Cichon S, Dannlowski U, de Geus ECJ, DePaulo JR, Domenici E, Domschke K, Esko T, Grabe HJ, Hamilton SP, Hayward C, Heath AC, Hinds DA, Kendler KS, Kloiber S, Lewis G, Li QS, Lucae S, Madden PFA, Magnusson PK, Martin NG, McIntosh AM, Metspalu A, Mors O, Mortensen PB, Müller-Myhsok B, Nordentoft M, Nöthen MM, O'Donovan MC, Paciga SA, Pedersen NL, Penninx BWJH, Perlis RH, Porteous DJ, Potash JB, Preisig M, Rietschel M, Schaefer C, Schulze TG, Smoller JW, Stefansson K, Tiemeier H, Uher R, Völzke H, Weissman MM, Werge T, Winslow AR, Lewis CM, Levinson DF, Breen G, Børglum AD, Sullivan PF; Major Depressive Disorder Working Group of the Psychiatric Genomics Consortium. Genome-wide association analyses identify 44 risk variants and refine the genetic architecture of major depression. Nat Genet. 2018 May;50(5):668-681. doi: 10.1038/s41588-018-0090-3. Epub 2018 Apr 26. PMID: 29700475; PMCID: PMC5934326.	UNFOCUSED
295	Kann L, McManus T, Harris WA, Shanklin SL, Flint KH, Queen B, Lowry R, Chyen D, Whittle L, Thornton J, Lim C, Bradford D, Yamakawa Y, Leon M, Brener N, Ethier KA. Youth Risk Behavior Surveillance - United States, 2017. MMWR Surveill Summ. 2018 Jun 15;67(8):1-114. doi: 10.15585/mmwr.ss6708a1. PMID: 29902162; PMCID: PMC6002027.	UNFOCUSED
296	Kapoor AK, Bhatnagar S. Are We Missing Out on Something? Indian J Palliat Care. 2018 Jul-Sep;24(3):381-383. doi: 10.4103/IJPC.IJPC_199_17. PMID: 30111959; PMCID: PMC6069621.	UNFOCUSED
297	Jafri F, Sammut A. A rare case of suicidal ideation related to Adalimumab use. Open Access Rheumatol. 2018 Aug 17;10:113-115. doi: 10.2147/OARRR.S168559. PMID: 30147384; PMCID: PMC6103321.	CASE
298	Djuricic G, Milosevic Z, Radovic T, Dasic I, Alempijevic D, Sopta J. A posttraumatic pseudoaneurysm of the left radial artery as a result of a stab wound in an 8-year-old girl. Forensic Sci Med Pathol. 2018 Sep;14(3):406-409. doi: 10.1007/s12024-018-9975-9. Epub 2018 Apr 11. PMID: 29644530.	CASE
299	Gerhart J, Chen E, O'Mahony S, Burns J, Hoerger M. An Examination of State- Level Personality Variation and Physician Aid in Dying Legislation. J Pain Symptom Manage. 2018 Sep;56(3):385-389. doi: 10.1016/j.jpainsymman.2018.05.023. Epub 2018 Jun 6. PMID: 29885459.	UNFOCUSED
300	Aboumrad M, Shiner B, Riblet N, Mills PD, Watts BV. Factors contributing to cancer-related suicide: A study of root-cause analysis reports. Psychooncology. 2018 Sep;27(9):2237-2244. doi: 10.1002/pon.4815. Epub 2018 Jul 18. PMID: 30019361; PMCID: PMC8803475.	UNFOCUSED

301	Yun YH, Kim KN, Sim JA, Yoo SH, Kim M, Kim YA, Kang BD, Shim HJ, Song EK, Kang JH, Kwon JH, Lee JL, Nam EM, Maeng CH, Kang EJ, Do YR, Choi YS, Jung KH. Comparison of attitudes towards five end-of-life care interventions (active pain control, withdrawal of futile life-sustaining treatment, passive euthanasia, active euthanasia and physician-assisted suicide): a multicentred cross- sectional survey of Korean patients with cancer, their family caregivers, physicians and the general Korean population. BMJ Open. 2018 Sep 11;8(9):e020519. doi: 10.1136/bmjopen-2017-020519. Erratum in: BMJ Open. 2019 Aug 26;9(8):e020519corr1. doi: 10.1136/bmjopen-2017-020519corr1. PMID: 30206075; PMCID: PMC6144336.	UNFOCUSED
302	Akechi T. Psycho-oncology: History, Current Status, and Future Directions in Japan. JMA J. 2018 Sep 28;1(1):22-29. doi: 10.31662/jmaj.2018-0001. PMID: 33748519; PMCID: PMC7969909.	UNFOCUSED
303	Castañeda AM, Lee CS, Kim YC, Lee D, Moon JY. Addressing Opioid-Related Chemical Coping in Long-Term Opioid Therapy for Chronic Noncancer Pain: A Multicenter, Observational, Cross-Sectional Study. J Clin Med. 2018 Oct 14;7(10):354. doi: 10.3390/jcm7100354. PMID: 30322212; PMCID: PMC6210168.	UNFOCUSED
304	Jayatilleke N, Hayes RD, Chang CK, Stewart R. Acute general hospital admissions in people with serious mental illness. Psychol Med. 2018 Dec;48(16):2676-2683. doi: 10.1017/S0033291718000284. Epub 2018 Feb 28. PMID: 29486806; PMCID: PMC6236443.	UNFOCUSED
305	Heffner KL, France CR, Ashrafioun L, Quiñones M, Walsh P, Maloney MD, Giordano BD, Pigeon WR. Clinical Pain-related Outcomes and Inflammatory Cytokine Response to Pain Following Insomnia Improvement in Adults With Knee Osteoarthritis. Clin J Pain. 2018 Dec;34(12):1133-1140. doi: 10.1097/AJP.0000000000000644. PMID: 30134281; PMCID: PMC6219931.	UNFOCUSED
306	Mercadante S, Masedu F, Degan G, Marinangeli F, Aielli F; “Home Care Italy” group (HOCAI group). Physicians' Attitudes Toward Euthanasia and Assisted Suicide in Italy. J Pain Symptom Manage. 2018 Dec;56(6):e1-e3. doi: 10.1016/j.jpainsymman.2018.09.007. Epub 2018 Sep 18. PMID: 30236790.	UNFOCUSED
307	Miller D, Nevadunsky N. Palliative Care and Symptom Management for Women with Advanced Ovarian Cancer. Hematol Oncol Clin North Am. 2018 Dec;32(6):1087-1102. doi: 10.1016/j.hoc.2018.07.012. PMID: 30390762.	REVIEW
308	Balducci L. Geriatric Oncology, Spirituality, and Palliative Care. J Pain Symptom Manage. 2019 Jan;57(1):171-175. doi: 10.1016/j.jpainsymman.2018.05.009. Epub 2018 Jun 19. PMID: 29772281.	UNFOCUSED
309	Konishi Y, Nakata Y, Nemoto A, Ushijima M, Matsuura M. The preoperative risk factors of postoperative self-extubation in elderly patients. Int J Risk Saf Med. 2019;30(1):9-18. doi: 10.3233/JRS-180011. PMID: 30175984.	UNFOCUSED
310	Aigner KR, Selak E, Aigner K. Short-term intra-arterial infusion chemotherapy for head and neck cancer patients maintaining quality of life. J Cancer Res Clin Oncol. 2019 Jan;145(1):261-268. doi: 10.1007/s00432-018-2784-4. Epub 2018 Oct 31. PMID: 30382368; PMCID: PMC6325995.	UNFOCUSED
311	Henson KE, Brock R, Charnock J, Wickramasinghe B, Will O, Pitman A. Risk of Suicide After Cancer Diagnosis in England. JAMA Psychiatry. 2019 Jan 1;76(1):51-60. doi: 10.1001/jamapsychiatry.2018.3181. PMID: 30476945; PMCID: PMC6583458.	UNFOCUSED
312	Seemann L, Padala SA, Mohammed A, Belayneh N. Tumor-Induced Osteomalacia and the Importance of Plasma Fibroblast Growth Factor 23 as an Indicator: Diagnostic Delay Leads to a Suicide Attempt. J Investig Med High Impact Case Rep. 2019 Jan-Dec;7:2324709619895162. doi: 10.1177/2324709619895162. PMID: 31850815; PMCID: PMC6923526.	CASE
313	Vaegter HB, Støten M, Silseth SL, Erlangsen A, Handberg G, Sondergaard S, Stenager E. Cause-specific mortality of patients with severe chronic pain referred to a multidisciplinary pain clinic: a cohort register-linkage study. Scand J Pain. 2019 Jan 28;19(1):93-99. doi: 10.1515/sjpain-2018-0094. PMID: 30205653.	UNFOCUSED

314	Fettel J, Kühn B, Guillen NA, Sürün D, Peters M, Bauer R, Angioni C, Geisslinger G, Schnütgen F, Meyer Zu Heringdorf D, Werz O, Meybohm P, Zacharowski K, Steinhilber D, Roos J, Maier TJ. Sphingosine-1-phosphate (S1P) induces potent anti-inflammatory effects in vitro and in vivo by S1P receptor 4-mediated suppression of 5-lipoxygenase activity. FASEB J. 2019 Feb;33(2):1711-1726. doi: 10.1096/fj.201800221R. Epub 2018 Sep 6. PMID: 30188757.	UNFOCUSED
315	Evenblij K, Pasman HRW, van der Heide A, Hoekstra T, Onwuteaka-Philipsen BD. Factors associated with requesting and receiving euthanasia: a nationwide mortality follow-back study with a focus on patients with psychiatric disorders, dementia, or an accumulation of health problems related to old age. BMC Med. 2019 Feb 19;17(1):39. doi: 10.1186/s12916-019-1276-y. PMID: 30777057; PMCID: PMC6379969.	UNFOCUSED
316	Li M, Sun S, Dangelmajer S, Zhang Q, Wang J, Hu F, Dong F, Kahlert UD, Zhu M, Lei T. Exploiting tumor-intrinsic signals to induce mesenchymal stem cell- mediated suicide gene therapy to fight malignant glioma. Stem Cell Res Ther. 2019 Mar 12;10(1):88. doi: 10.1186/s13287-019-1194-0. PMID: 30867058; PMCID: PMC6417183.	UNFOCUSED
317	Choi NG, DiNitto DM, Marti CN, Conwell Y. Physical Health Problems as a Late-Life Suicide Precipitant: Examination of Coroner/Medical Examiner and Law Enforcement Reports. Gerontologist. 2019 Mar 14;59(2):356-367. doi: 10.1093/geront/gnx143. PMID: 28958040.	UNFOCUSED
318	Burghardt J, Klein E, Brähler E, Ernst M, Schneider A, Eckerle S, Neu MA, Wingerter A, Henninger N, Panova-Noeva M, Prochaska J, Wild P, Beutel M, Faber J. Prevalence of mental distress among adult survivors of childhood cancer in Germany-Compared to the general population. Cancer Med. 2019 Apr;8(4):1865-1874. doi: 10.1002/cam4.1936. Epub 2019 Mar 6. PMID: 30838816; PMCID: PMC6488141.	UNFOCUSED
319	Kraus C, Kadriu B, Lanzenberger R, Zarate CA Jr, Kasper S. Prognosis and improved outcomes in major depression: a review. Transl Psychiatry. 2019 Apr 3;9(1):127. doi: 10.1038/s41398-019-0460-3. PMID: 30944309; PMCID: PMC6447556.	REVIEW
320	Gwak SJ, Lee JS. Suicide Gene Therapy By Amphiphilic Copolymer Nanocarrier for Spinal Cord Tumor. Nanomaterials (Basel). 2019 Apr 8;9(4):573. doi: 10.3390/nano9040573. PMID: 30965667; PMCID: PMC6523721.	UNFOCUSED
321	McFarland DC, Walsh L, Napolitano S, Morita J, Jaiswal R. Suicide in Patients With Cancer: Identifying the Risk Factors. Oncology (Williston Park). 2019 Jun 19;33(6):221-6. PMID: 31219606.	REVIEW
322	Sinyor M, Williams M, Gulati S, Schaffer A. An Observational Study of Suicide Deaths by Self-Poisoning with Opioids in Toronto (1998-2015). Can J Psychiatry. 2019 Aug;64(8):577-583. doi: 10.1177/0706743719838777. Epub 2019 Mar 24. PMID: 30905165; PMCID: PMC6681511.	UNFOCUSED
323	Gallaway MS, Fink DS, Sampson L, Cohen GH, Tamburrino M, Liberzon I, Calabrese J, Galea S. Prevalence and covariates of problematic gambling among a US military cohort. Addict Behav. 2019 Aug;95:166-171. doi: 10.1016/j.addbeh.2019.03.013. Epub 2019 Mar 21. PMID: 30928661; PMCID: PMC6574081.	UNFOCUSED
324	Correction: <i>Comparison of attitudes towards five end-of-life care interventions (active pain control, withdrawal of futile life-sustaining treatment, passive euthanasia, active euthanasia and physician-assisted suicide): a multicentred cross-sectional survey of Korean patients with cancer, their family caregivers, physicians and the general Korean population</i>. BMJ Open. 2019 Aug 26;9(8):e020519corr1. doi: 10.1136/bmjopen-2017-020519corr1. Erratum for: BMJ Open. 2018 Sep 11;8(9):e020519. doi: 10.1136/bmjopen-2017-020519. PMID: 31455719; PMCID: PMC6719762.	DUPLICATE
325	Granek L, Nakash O, Ariad S, Shapira S, Ben-David M. Strategies and Barriers in Addressing Mental Health and Suicidality in Patients With Cancer. Oncol Nurs Forum. 2019 Sep 1;46(5):561-571. doi: 10.1188/19.ONF.561-571. PMID: 31424452.	UNFOCUSED
326	Bulotiene G, Pociute K. Interventions for Reducing Suicide Risk in Cancer Patients: A Literature Review. Eur J Psychol. 2019 Sep 27;15(3):637-649. doi: 10.5964/ejop.v15i3.1741. PMID: 33680150; PMCID: PMC7909181.	REVIEW

327	Burstein R, Henry NJ, Collison ML, Marczak LB, Sligar A, Watson S, Marquez N, Abbasalizad-Farhangi M, Abbasi M, Abd-Allah F, Abdoli A, Abdollahi M, Abdollahpour I, Abdulkader RS, Abrigo MRM, Acharya D, Adebayo OM, Adekanmbi V, Adham D, Afshari M, Aghaali M, Ahmadi K, Ahmadi M, Ahmadpour E, Ahmed R, Akal CG, Akinyemi JO, Alahdab F, Alam N, Alamene GM, Alene KA, Alijanzadeh M, Alinia C, Alipour V, Aljunid SM, Almalki MJ, Al-Mekhlafi HM, Altirkawi K, Alvis-Guzman N, Amegah AK, Amini S, Amit AML, Anbari Z, Androudi S, Anjomshoa M, Ansari F, Antonio CAT, Arabloo J, Arefi Z, Aremu O, Armoon B, Arora A, Artaman A, Asadi A, Asadi-Aliabadi M, Ashraf-Ganjouei A, Assadi R, Ataeinia B, Atre SR, Quintanilla BPA, Ayanore MA, Azari S, Babaee E, Babazadeh A, Badawi A, Bagheri S, Bagherzadeh M, Baheiraei N, Balouchi A, Barac A, Bassat Q, Baune BT, Bayati M, Bedi N, Beghi E, Behzadifar M, Behzadifar M, Belay YB, Bell B, Bell ML, Berbada DA, Bernstein RS, Bhattacharjee NV, Bhattarai S, Bhutta ZA, Bijani A, Bohlouli S, Breitborde NJK, Britton G, Browne AJ, Nagaraja SB, Busse R, Butt ZA, Car J, Cárdenas R, Castañeda-Orjuela CA, Cerin E, Chanie WF, Chatterjee P, Chu DT, Cooper C, Costa VM, Dalal K, Dandona L, Dandona R, Daoud F, Daryani A, Das Gupta R, Davis I, Davis Weaver N, Davitoiu DV, De Neve JW, Demeke FM, Demoz GT, Deribe K, Desai R, Deshpande A, Desyibelew HD, Dey S, Dharmaratne SD, Dhimal M, Diaz D, Doshmangir L, Duraes AR, Dwyer-Lindgren L, Earl L, Ebrahimi R, Ebrahimpour S, Effiong A, Eftekhari A, Ehsani-Chimeh E, El Sayed I, El Sayed Zaki M, El Tantawi M, El-Khatib Z, Emamian MH, Enany S, Eskandarieh S, Eyawo O, Ezalarab M, Faramarzi M, Fareed M, Faridnia R, Faro A, Fazaeli AA, Fazlzadeh M, Fentahun N, Fereshtehnejad SM, Fernandes JC, Filip I, Fischer F, Foigt NA, Foroutan M, Francis JM, Fukumoto T, Fullman N, Gallus S, Gebre DG, Gebrehiwot TT, Gebremeskel GG, Gessner BD, Geta B, Gething PW, Ghadimi R, Ghadiri K, Ghajarzadeh M, Ghashghaee A, Gill PS, Gill TK, Golding N, Gomes NGM, Gona PN, Gopalani SV, Gorini G, Goulart BNG, Graetz N, Greaves F, Green MS, Guo Y, Haj- Mirzaian A, Haj-Mirzaian A, Hall BJ, Hamidi S, Haririan H, Haro JM, Hasankhani M, Hasanpoor E, Hasanzadeh A, Hassankhani H, Hassen HY, Hegazy MI, Hendrie D, Heydarpour F, Hird TR, Hoang CL, Hollerich G, Rad EH, Hoseini-Ghahfarokhi M, Hossain N, Hosseini M, Hosseinzadeh M, Hostiuc M, Hostiuc S, Househ M, Hsairi M, Ilesanmi OS, Imani-Nasab MH, Iqbal U, Irvani SSN, Islam N, Islam SMS, Jürisson M, Balalami NJ, Jalali A, Javidnia J, Jayatilleke AU, Jenabi E, Ji JS, Jobanputra YB, Johnson K, Jonas JB, Shushtari ZJ, Jozwiak JJ, Kabir A, Kahsay A, Kalani H, Kalhor R, Karami M, Karki S, Kasaeian A, Kassebaum NJ, Keiyoro PN, Kemp GR, Khabiri R, Khader YS, Khafaie MA, Khan EA, Khan J, Khan MS, Khang YH, Khatab K, Khater A, Khater MM, Khatony A, Khazaei M, Khazaei S, Khazaei-Pool M, Khubchandani J, Kianipour N, Kim YJ, Kimokoti RW, Kinyoki DK, Kisa A, Kisa S, Kolola T, Kosen S, Koul PA, Koyanagi A, Kraemer MUG, Krishan K, Krohn KJ, Kugbey N, Kumar GA, Kumar M, Kumar P, Kuupiel D, Lacey B, Lad SD, Lami FH, Larsson AO, Lee PH, Leili M, Levine AJ, Li S, Lim LL, Listl S, Longbottom J, Lopez JCF, Lorkowski S, Magdeldin S, Abd El Razek HM, Abd El Razek MM, Majeed A, Maleki A, Malekzadeh R, Malta DC, Mamun AA, Manafi N, Manda AL, Mansourian M, Martins-Melo FR, Masaka A, Massenburg BB, Maulik PK, Mayala BK, Mazidi M, McKee M, Mehrotra R, Mehta KM, Meles GG, Mendoza W, Menezes RG, Meretoja A, Meretoja TJ, Mestrovic T, Miller TR, Miller-Petrie MK, Mills EJ, Milne GJ, Mini GK, Mir SM, Mirjalali H, Mirrakhimov EM, Mohamadi E, Mohammad DK, Darwesh AM, Mezerji NMG, Mohammed AS, Mohammed S, Mokdad AH, Molokhia M, Monasta L, Moodley Y, Moosazadeh M, Moradi G, Moradi M, Moradi Y, Moradi-Lakeh M, Moradinazar M, Moraga P, Morawska L, Mosapour A, Mousavi SM, Mueller UO, Muluneh AG, Mustafa G, Nabavizadeh B, Naderi M, Nagarajan AJ, Nahvijou A, Najafi F, Nangia V, Ndwandwe DE, Neamati N, Negoi I, Negoi RI, Ngunjiri JW, Thi Nguyen HL, Nguyen LH, Nguyen SH, Nielsen KR, Ningrum DNA, Nirayo YL, Nixon MR, Nnaji CA, Nojomi M, Noroozi M, Nosratnejad S, Noubiap JJ, Motlagh SN, Ofori-Asenso R, Ogbo FA, Oladimeji KE, Olagunju AT, Olfatifar M, Olum S, Olusanya BO, Oluwasanu MM, Onwujekwe OE, Oren E, Ortega- Altamirano DDV, Ortiz A, Osarenotor O, Osei FB, Osgood-Zimmerman AE, Otstavnov SS, Owolabi MO, P A M, Pagheh AS, Pakhale S, Panda-Jonas S, Pandey A, Park EK, Parsian H, Pashaei T, Patel SK, Pepito VCF, Pereira A, Perkins S, Pickering BV, Pilgrim T, Pirestani M, Piroozi B, Pirsaheb M, Plana-Ripoll O, Pourjafar H, Puri P, Qorbani M, Quintana H, Rabiee M, Rabiee N, Radfar A, Rafiei A, Rahim F, Rahimi Z, Rahimi-Movaghar V, Rahimzadeh S, Rajati F, Raju SB, Ramezankhani A, Ranabhat CL, Rasella D, Rashedi V, Rawal L, Reiner RC Jr, Renzaho AMN, Rezaei S, Rezapour A, Riahi SM, Ribeiro AI, Roever L, Roro EM, Roser M, Roshandel G, Roshani D, Rostami A, Rubagotti E, Rubino S, Sabour S, Sadat N, Sadeghi E, Saeedi R, Safari Y, Safari-Faramani R, Safdarian M, Sahebkar A, Salahshoor MR, Salam N, Salamati P, Salehi F, Zahabi SS, Salimi Y, Salimzadeh H, Salomon JA, Sambala EZ, Samy AM, Santric Milicevic MM, Jose BPS, Saraswathy SYI, Sarmiento- Suárez R, Sartorius B, Sathian B, Saxena S, Sbarra AN, Schaeffer LE, Schwebel DC, Sepanlou SG, Seyedmousavi S, Shaahmadi F, Shaikh MA, Shams-Beyranvand M, Shamshirian A, Shamsizadeh M, Sharafi K, Sharif M, Sharif-Alhoseini M, Sharifi H, Sharma J, Sharma R, Sheikh A, Shields C, Shigematsu M, Shiri R, Shiue I, Shuval K, Siddiqi TJ, Silva JP, Singh JA, Sinha DN, Sisay MM, Sisay S, Sliwa K, Smith DL, Somayaji R, Soofi M, Soriano JB, Sreeramareddy CT, Sudaryanto A, Sufiyan	UNFOCUSED

	MB, Sykes BL, Sylaja PN, Tabarés-Seisdedos R, Tabb KM, Tabuchi T, Taveira N, Temsah MH, Terkawi AS, Tessema ZT, Thankappan KR, Thirunavukkarasu S, To QG, Tovani-Palone MR, Tran BX, Tran KB, Ullah I, Usman MS, Uthman OA, Vahedian-Azimi A, Valdez PR, van Boven JFM, Vasankari TJ, Vasseghian Y, Veisani Y, Venketasubramanian N, Violante FS, Vladimirov SK, Vlassov V, Vos T, Vu GT, Vujcic IS, Waheed Y, Wakefield J, Wang H, Wang Y, Wang YP, Ward JL, Weintraub RG, Weldegwergs KG, Weldesamuel GT, Westerman R, Wiysonge CS, Wondafrash DZ, Woyczynski L, Wu AM, Xu G, Yadegar A, Yamada T, Yazdi-Feyzabadi V, Yilgwan CS, Yip P, Yonemoto N, Lebni JY, Younis MZ, Yousefifard M, Yousof HSA, Yu C, Yusefzadeh H, Zabeh E, Moghadam TZ, Bin Zaman S, Zamani M, Zandian H, Zangeneh A, Zerfu TA, Zhang Y, Ziapour A, Zodpey S, Murray CJL, Hay SI. Mapping 123 million neonatal, infant and child deaths between 2000 and 2017. Nature. 2019 Oct;574(7778):353-358. doi: 10.1038/s41586-019-1545-0. Epub 2019 Oct 16. PMID: 31619795; PMCID: PMC6800389.	
328	Hou L, Rao DA, Yuki K, Cooley J, Henderson LA, Jonsson AH, Kaiserman D, Gorman MP, Nigrovic PA, Bird PI, Becher B, Remold-O'Donnell E. SerpinB1 controls encephalitogenic T helper cells in neuroinflammation. Proc Natl Acad Sci U S A. 2019 Oct 8;116(41):20635-20643. doi: 10.1073/pnas.1905762116. Epub 2019 Sep 23. PMID: 31548399; PMCID: PMC6789640.	UNFOCUSED
329	Patel P, Konala VM, Adapa S, Gayam V, Sahasranam P, Bose S, Golez CD, Naramala S. Case Report of Lethal Toxin Lurking in an Edible Plant. J Popul Ther Clin Pharmacol. 2019 Oct 22;26(3):e14-e18. doi: 10.15586/jptcp.v26i3.633. PMID: 31904202.	CASE
330	Dragioti E, Solmi M, Favaro A, Fusar-Poli P, Dazzan P, Thompson T, Stubbs B, Firth J, Fornaro M, Tsartsalis D, Carvalho AF, Vieta E, McGuire P, Young AH, Shin JI, Correll CU, Evangelou E. Association of Antidepressant Use With Adverse Health Outcomes: A Systematic Umbrella Review. JAMA Psychiatry. 2019 Dec 1;76(12):1241-1255. doi: 10.1001/jamapsychiatry.2019.2859. Erratum in: JAMA Psychiatry. 2021 May 1;78(5):569. doi: 10.1001/jamapsychiatry.2021.0314. PMID: 31577342; PMCID: PMC6777224.	REVIEW
331	Johnson CC, Phillips KM, Miller SN. Suicidal Ideation among Veterans Living with Cancer Referred to Mental Health. Clin Gerontol. 2020 Jan-Feb;43(1):24-36. doi: 10.1080/07317115.2019.1686719. Epub 2019 Nov 4. PMID: 31680645.	INCLUDED
332	Yang LK, Lu L, Feng B, Wang XS, Yue J, Li XB, Zhuo M, Liu SB. FMRP acts as a key messenger for visceral pain modulation. Mol Pain. 2020 Jan- Dec;16:1744806920972241. doi: 10.1177/1744806920972241. PMID: 33243040; PMCID: PMC7786421.	UNFOCUSED
333	Jahn-Kuch D, Domke A, Bitsche S, Stöger H, Avian A, Jeitler K, Posch N, Siebenhofer A. End-of-life decision making by Austrian physicians - a cross- sectional study. BMC Palliat Care. 2020 Jan 4;19(1):4. doi: 10.1186/s12904-019-0509-3. PMID: 31901225; PMCID: PMC6942327.	UNFOCUSED
334	Rodríguez-Mayoral O, Pérez-Esparza R, Domínguez-Ocadio G, Allende-Pérez S. Ketamine as augmentation for the treatment of major depression and suicidal risk in advanced cancer: Case report. Palliat Support Care. 2020 Feb;18(1):110-112. doi: 10.1017/S1478951519000580. PMID: 31397251.	CASE
335	Lantheaume S, Montagne M, Shankland R. Intervention centrée sur les ressources pour réduire les troubles anxieux et dépressifs chez les patients atteints de cancer : une étude pilote [Intervention focused on resources to reduce anxiety and depression disorders in cancer patients: A pilot study]. Encephale. 2020 Feb;46(1):13-22. French. doi: 10.1016/j.encep.2019.07.005. Epub 2019 Oct 11. PMID: 31610923.	UNFOCUSED
336	Shibata T, Yoshimatsu H, Yano T, Karakawa R, Ishikawa S, Takeda A. Spinal infarction caused by hypovolemic shock following massive bleeding from stab wounds to the neck. Trauma Case Rep. 2019 Dec 18;25:100269. doi: 10.1016/j.tcr.2019.100269. Erratum in: Trauma Case Rep. 2023 Feb 17;45:100793. doi: 10.1016/j.tcr.2023.100793. Erratum in: Trauma Case Rep. 2023 Mar 01;45:100814. doi: 10.1016/j.tcr.2023.100814. PMID: 31890834; PMCID: PMC6928351.	CASE
337	Amin F, Khan MS, Bano B. Mammalian cystatin and protagonists in brain diseases. J Biomol Struct Dyn. 2020 Apr;38(7):2171-2196. doi: 10.1080/07391102.2019.1620636. Epub 2019 Jun 5. PMID: 31107181.	UNFOCUSED

338	Tang L, Zhang Y, Pang Y. Patient-reported outcomes from the distress assessment and response tool program in Chinese cancer inpatients. Psychooncology. 2020 May;29(5):869-877. doi: 10.1002/pon.5358. Epub 2020 Feb 24. PMID: 32040238.	UNFOCUSED
339	Filetti M, D'Amuri S, Giusti R. La salute come diritto umano, il diritto alla vita e la libertà di scegliere [Health as a human right, the right to life and the freedom to choose.]. Recenti Prog Med. 2020 May;111(5):285-286. Italian. doi: 10.1701/3366.33406. PMID: 32448875.	UNFOCUSED
340	Kessler RC, Bauer MS, Bishop TM, Demler OV, Dobscha SK, Gildea SM, Goulet JL, Karras E, Kreyenbuhl J, Landes SJ, Liu H, Luedtke AR, Mair P, McAuliffe WHB, Nock M, Petukhova M, Pigeon WR, Sampson NA, Smoller JW, Weinstock LM, Bossarte RM. Using Administrative Data to Predict Suicide After Psychiatric Hospitalization in the Veterans Health Administration System. Front Psychiatry. 2020 May 6;11:390. doi: 10.3389/fpsyt.2020.00390. PMID: 32435212; PMCID: PMC7219514.	UNFOCUSED
341	Tran K, McCormack S. Ketamine for Chronic Non-Cancer Pain: A Review of Clinical Effectiveness, Cost-Effectiveness, and Guidelines [Internet]. Ottawa (ON): Canadian Agency for Drugs and Technologies in Health; 2020 May 28. PMID: 33231962.	REVIEW
342	Lee Y, Hung CF, Chien CY, Lin PY, Lin MC, Wang CC, Lu HI, Chen YC, Chong MY, Wang LJ. Comparison of prevalence and associated factors of depressive disorder between patients with head and neck cancer and those with lung cancer at a tertiary hospital in Taiwan: a cross-sectional study. BMJ Open. 2020 Jun 29;10(6):e037918. doi: 10.1136/bmjopen-2020-037918. PMID: 32601116; PMCID: PMC7328812.	UNFOCUSED
343	Orr MF, Rogers AH, Shepherd JM, Buckner JD, Ditre JW, Bakhshaie J, Zvolensky MJ. Is there a relationship between cannabis use problems, emotion dysregulation, and mental health problems among adults with chronic pain? Psychol Health Med. 2020 Jul;25(6):742-755. doi: 10.1080/13548506.2019.1653485. Epub 2019 Aug 13. PMID: 31407604.	UNFOCUSED
344	Andersen BL, Valentine TR, Lo SB, Carbone DP, Presley CJ, Shields PG. Newly diagnosed patients with advanced non-small cell lung cancer: A clinical description of those with moderate to severe depressive symptoms. Lung Cancer. 2020 Jul;145:195-204. doi: 10.1016/j.lungcan.2019.11.015. Epub 2019 Nov 21. PMID: 31806360; PMCID: PMC7239743.	UNFOCUSED
345	Gupta MA, Vujcic B, Sheridan AD, Gupta AK. Reduced risk of suicidal behaviours associated with the treatment of hidradenitis suppurativa with tumour necrosis factor alpha antagonists: results from the US FDA Adverse Events Reporting System pharmacovigilance database. J Eur Acad Dermatol Venereol. 2020 Jul;34(7):1564-1568. doi: 10.1111/jdv.16224. Epub 2020 Feb 19. PMID: 31981260.	UNFOCUSED
346	Isenberg-Grzeda E, Bean S, Cohen C, Selby D. Suicide Attempt After Determination of Ineligibility for Assisted Death: A Case Series. J Pain Symptom Manage. 2020 Jul;60(1):158-163. doi: 10.1016/j.jpainsymman.2020.02.016. Epub 2020 Feb 24. PMID: 32105792.	CASE
347	Akbar N J M, Shekhawat RS, Gorchiya A, Meshram VP, Bharti JN. Forensic implications of pituitary macroadenoma: An autopsy case report. J Forensic Leg Med. 2020 Jul;73:101991. doi: 10.1016/j.jflm.2020.101991. Epub 2020 May 30. PMID: 32658749.	CASE
348	Chowdhury FH, Rumi JUM, Zainab F, Hakim M. Ganglioneuroma of Glossopharyngeal Nerve in a Patient with Glossopharyngeal Neuralgia: A Case Report. NMC Case Rep J. 2020 Jun 24;7(3):117-120. doi: 10.2176/nmccrj.cr.2019-0094. PMID: 32695559; PMCID: PMC7363645.	CASE
349	Phan K, Huo YR, Smith SD. Hidradenitis suppurativa and psychiatric comorbidities, suicides and substance abuse: systematic review and meta- analysis. Ann Transl Med. 2020 Jul;8(13):821. doi: 10.21037/atm-20-1028. PMID: 32793666; PMCID: PMC7396254.	REVIEW
350	Zhang Y, Li W, Zhang Z, Sun H, Garg S, Yang Y, Wang H. Suicidal Ideation in Newly-Diagnosed Chinese Cancer Patients. Front Psychiatry. 2020 Jul 23;11:708. doi: 10.3389/fpsyt.2020.00708. PMID: 32793000; PMCID: PMC7390886.	INCLUDED

351	Rouzer CA, Marnett LJ. Structural and Chemical Biology of the Interaction of Cyclooxygenase with Substrates and Non-Steroidal Anti-Inflammatory Drugs. Chem Rev. 2020 Aug 12;120(15):7592-7641. doi: 10.1021/acs.chemrev.0c00215. Epub 2020 Jul 1. PMID: 32609495; PMCID: PMC8253488.	UNFOCUSED
352	Bommier C, Charlier P, Hervé C. Chapter 7. What symbolic answers to death in the medical world? J Int Bioethique Ethique Sci. 2020 Sep 14;Vol. 31(1):85-96. English. doi: 10.3917/jibes.311.0085. PMID: 33089677.	UNFOCUSED
353	Haagsma JA, James SL, Castle CD, Dingels ZV, Fox JT, Hamilton EB, Liu Z, Lucchesi LR, Roberts NLS, Sylte DO, Adebayo OM, Ahmadi A, Ahmed MB, Aichour MTE, Alahdab F, Alghnam SA, Aljunid SM, Al-Raddadi RM, Alsharif U, Altirkawi K, Anjomshoa M, Antonio CAT, Appiah SCY, Aremu O, Arora A, Asayesh H, Assadi R, Awasthi A, Ayala Quintanilla BP, Balalla S, Banstola A, Barker-Collo SL, Bärnighausen TW, Bazargan-Hejazi S, Bedi N, Behzadifar M, Behzadifar M, Benjet C, Bennett DA, Bensenor IM, Bhaumik S, Bhutta ZA, Bijani A, Borges G, Borschmann R, Bose D, Boufous S, Brazinova A, Campuzano Rincon JC, Cárdenas R, Carrero JJ, Carvalho F, Castañeda-Orjuela CA, Catalá-López F, Choi JJ, Christopher DJ, Crowe CS, Dalal K, Daryani A, Davitoiu DV, Degenhardt L, De Leo D, De Neve JW, Deribe K, Dessie GA, deVeber GA, Dharmaratne SD, Doan LP, Dolan KA, Driscoll TR, Dubey M, El-Khatib Z, Ellingsen CL, El Sayed Zaki M, Endries AY, Eskandarieh S, Faro A, Fereshtehnejad SM, Fernandes E, Filip I, Fischer F, Franklin RC, Fukumoto T, Gezae KE, Gill TK, Goulart AC, Grada A, Guo Y, Gupta R, Haghparast Bidgoli H, Haj-Mirzaian A, Haj-Mirzaian A, Hamadeh RR, Hamidi S, Haro JM, Hassankhani H, Hassen HY, Havmoeller R, Hendrie D, Henok A, Híjar M, Hole MK, Homaie Rad E, Hossain N, Hostiuc S, Hu G, Igumbor EU, Ilesanmi OS, Irvani SSN, Islam SMS, Ivers RQ, Jacobsen KH, Jahanmehr N, Jakovljevic M, Jayatilleke AU, Jha RP, Jonas JB, Jorjoran Shushtari Z, Jozwiak JJ, Jürisson M, Kabir A, Kalani R, Kasaeian A, Kelbore AG, Kengne AP, Khader YS, Khafaie MA, Khalid N, Khan EA, Khoja AT, Kiadaliri AA, Kim YE, Kim D, Kisa A, Koyanagi A, Kuate Defo B, Kucuk Bicer B, Kumar M, Lalloo R, Lam H, Lami FH, Lansingh VC, Leasher JL, Li S, Linn S, Lunevicius R, Machado FR, Magdy Abd El Razek H, Magdy Abd El Razek M, Mahotra NB, Majdan M, Majeed A, Malekzadeh R, Malik MA, Malta DC, Manda AL, Mansournia MA, Massenburg BB, Maulik PK, Meheretu HAA, Mehndiratta MM, Melese A, Mendoza W, Mengesha MM, Meretoja TJ, Meretoja A, Mestrovic T, Miazgowski T, Miller TR, Mini GK, Mirrakhimov EM, Moazen B, Mohammad Gholi Mezerji N, Mohammadibakhsh R, Mohammed S, Molokhia M, Monasta L, Mondello S, Montero-Zamora PA, Moodley Y, Moosazadeh M, Moradi G, Moradi-Lakeh M, Morawska L, Velásquez IM, Morrison SD, Moschos MM, Mousavi SM, Murthy S, Musa KI, Naik G, Najafi F, Nangia V, Nascimento BR, Ndwandwe DE, Negoi I, Nguyen TH, Nguyen SH, Nguyen LH, Nguyen HLT, Ningrum DNA, Nirayo YL, Ofori-Asenso R, Ogbo FA, Oh IH, Oladimeji O, Olagunju AT, Olagunju TO, Olivares PR, Orpana HM, Otstavnov SS, P A M, Pakhale S, Park EK, Patton GC, Pesudovs K, Phillips MR, Polinder S, Prakash S, Radfar A, Rafay A, Rafiei A, Rahimi S, Rahimi-Movaghar V, Rahman MA, Rai RK, Ramezanzadeh K, Rawaf S, Rawaf DL, Renzaho AMN, Resnikoff S, Rezaeian S, Roever L, Ronfani L, Roshandel G, Sabde YD, Saddik B, Salamati P, Salimi Y, Salz I, Samy AM, Sanabria J, Sanchez Riera L, Santric Milicevic MM, Satpathy M, Sawhney M, Sawyer SM, Saxena S, Saylan M, Schneider IJC, Schwebel DC, Seedat S, Sepanlou SG, Shaikh MA, Shams-Beyranvand M, Shamsizadeh M, Sharif-Alhoseini M, Sheikh A, Shen J, Shigematsu M, Shiri R, Shiue I, Silva JP, Singh JA, Sinha DN, Soares Filho AM, Soriano JB, Soshnikov S, Soyiri IN, Starodubov VI, Stein DJ, Stokes MA, Sufiyan MB, Sunshine JE, Sykes BL, Tabarés-Seisdedos R, Tabb KM, Tehrani-Banihashemi A, Tessema GA, Thakur JS, Tran KB, Tran BX, Tudor Car L, Uthman OA, Uzochukwu BSC, Valdez PR, Varavikova E, Vasconcelos AMN, Venketasubramanian N, Violante FS, Vlassov V, Waheed Y, Wang YP, Wijeratne T, Winkler AS, Yadav P, Yano Y, Yenesew MA, Yip P, Yisma E, Yonemoto N, Younis MZ, Yu C, Zafar S, Zaidi Z, Zaman SB, Zamani M, Zhao Y, Zodpey S, Hay SI, Lopez AD, Mokdad AH, Vos T. Burden of injury along the development spectrum: associations between the Socio-demographic Index and disability-adjusted life year estimates from the Global Burden of Disease Study 2017. Inj Prev. 2020 Oct;26(Supp 1):i12-i26. doi: 10.1136/injuryprev-2019-043296. Epub 2020 Jan 8. Erratum in: Inj Prev. 2020 Oct;26(Supp 1):i164. doi: 10.1136/injuryprev-2019-043296corr1. PMID: 31915273; PMCID: PMC7571356.	UNFOCUSED

354	James SL, Castle CD, Dingels ZV, Fox JT, Hamilton EB, Liu Z, S Roberts NL, Sylte DO, Henry NJ, LeGrand KE, Abdelalim A, Abdoli A, Abdollahpour I, Abdulkader RS, Abedi A, Abosetugn AE, Abushouk AI, Adebayo OM, Agudelo-Botero M, Ahmad T, Ahmed R, Ahmed MB, Eddine Aichour MT, Alahdab F, Alamene GM, Alanezi FM, Alebel A, Alema NM, Alghnam SA, Al-Hajj S, Ali BA, Ali S, Alikhani M, Alinia C, Alipour V, Aljunid SM, Almasi-Hashiani A, Almasri NA, Altirkawi K, Abdeldayem Amer YS, Amini S, Loreche Amit AM, Andrei CL, Ansari-Moghaddam A, T Antonio CA, Yaw Appiah SC, Arabloo J, Arab-Zozani M, Arefi Z, Aremu O, Ariani F, Arora A, Asaad M, Asghari B, Awoke N, Ayala Quintanilla BP, Ayano G, Ayanore MA, Azari S, Azarian G, Badawi A, Badiye AD, Bagli E, Baig AA, Bairwa M, Bakhtiari A, Balachandran A, Banach M, Banerjee SK, Banik PC, Banstola A, Barker-Collo SL, Bärnighausen TW, Barrero LH, Barzegar A, Bayati M, Baye BA, Bedi N, Behzadifar M, Bekuma TT, Belete H, Benjet C, Bennett DA, Bensenor IM, Berhe K, Bhardwaj P, Bhat AG, Bhattacharyya K, Bibi S, Bijani A, Bin Sayeed MS, Borges G, Borzì AM, Boufous S, Brazinova A, Briko NI, Budhathoki SS, Car J, Cárdenas R, Carvalho F, Castaldelli-Maia JM, Castañeda-Orjuela CA, Castelpietra G, Catalá-López F, Cerin E, Chandan JS, Chanie WF, Chattu SK, Chattu VK, Chatziralli I, Chaudhary N, Cho DY, Kabir Chowdhury MA, Chu DT, Colquhoun SM, Constantin MM, Costa VM, Damiani G, Daryani A, Dávila-Cervantes CA, Demeke FM, Demis AB, Demoz GT, Demsie DG, Derakhshani A, Deribe K, Desai R, Nasab MD, da Silva DD, Dibaji Forooshani ZS, Doyle KE, Driscoll TR, Dubljanin E, Adema BD, Eagan AW, Eftekhari A, Ehsani- Chimeh E, Sayed Zaki ME, Elemineh DA, El-Jaafary SI, El-Khatib Z, Ellingsen CL, Emamian MH, Endalew DA, Eskandarieh S, Faris PS, Faro A, Farzadfar F, Fatahi Y, Fekadu W, Ferede TY, Fereshtehnejad SM, Fernandes E, Ferrara P, Feyissa GT, Filip I, Fischer F, Folayan MO, Foroutan M, Francis JM, Franklin RC, Fukumoto T, Geberemariyam BS, Gebre AK, Gebremedhin KB, Gebremeskel GG, Gebremichael B, Gedefaw GA, Geta B, Ghafourifard M, Ghamari F, Ghashghaee A, Gholamian A, Gill TK, Goulart AC, Grada A, Grivna M, Mohialdeen Gubari MI, Guimarães RA, Guo Y, Gupta G, Haagsma JA, Hafezi-Nejad N, Bidgoli HH, Hall BJ, Hamadeh RR, Hamidi S, Haro JM, Hasan MM, Hasanzadeh A, Hassanipour S, Hassankhani H, Hassen HY, Havmoeller R, Hayat K, Hendrie D, Heydarpour F, Híjar M, Ho HC, Hoang CL, Hole MK, Holla R, Hossain N, Hosseinzadeh M, Hostiuc S, Hu G, Ibitoye SE, Ilesanmi OS, Ilic I, Ilic MD, Inbaraj LR, Indriasih E, Naghibi Irvani SS, Shariful Islam SM, Islam MM, Ivers RQ, Jacobsen KH, Jahani MA, Jahanmehr N, Jakovljevic M, Jalilian F, Jayaraman S, Jayatilleke AU, Jha RP, John-Akinola YO, Jonas JB, Joseph N, Joukar F, Jozwiak JJ, Jungari SB, Jürisson M, Kabir A, Kadel R, Kahsay A, Kalankesh LR, Kalhor R, Kamil TA, Kanchan T, Kapoor N, Karami M, Kasaeian A, Kassaye HG, Kavetskyy T, Kebede HK, Keiyoro PN, Kelbore AG, Kelkay B, Khader YS, Khafaie MA, Khalid N, Khalil IA, Khalilov R, Khammarnia M, Khan EA, Khan M, Khanna T, Khazaie H, Shadmani FK, Khundkar R, Kiirithio DN, Kim YE, Kim D, Kim YJ, Kisa A, Kisa S, Komaki H, M Kondlahalli SK, Korshunov VA, Koyanagi A, G Kraemer MU, Krishan K, Bicer BK, Kugbey N, Kumar V, Kumar N, Kumar GA, Kumar M, Kumaresh G, Kurmi OP, Kuti O, Vecchia C, Lami FH, Lamichhane P, Lang JJ, Lansingh VC, Laryea DO, Lasrado S, Latifi A, Lauriola P, Leasher JL, Huey Lee SW, Lenjebo TL, Levi M, Li S, Linn S, Liu X, Lopez AD, Lotufo PA, Lunevicius R, Lyons RA, Madadin M, El Razek MMA, Mahotra NB, Majdan M, Majeed A, Malagon-Rojas JN, Maled V, Malekzadeh R, Malta DC, Manafi N, Manafi A, Manda AL, Manjunatha N, Mansour-Ghanaei F, Mansouri B, Mansournia MA, Maravilla JC, March LM, Mason- Jones AJ, Masoumi SZ, Massenburg BB, Maulik PK, Meles GG, Melese A, Melketsedik ZA, N Memiah PT, Mendoza W, Menezes RG, Mengesha MB, Mengesha MM, Meretoja TJ, Meretoja A, Merie HE, Mestrovic T, Miazgowski B, Miazgowski T, Miller TR, Mini GK, Mirica A, Mirrakhimov EM, Mirzaei-Alavijeh M, Mithra P, Moazen B, Moghadaszadeh M, Mohamadi E, Mohammad Y, Mohammad KA, Darwesh AM, Gholi Mezerji NM, Mohammadian-Hafshejani A, Mohammadoo-Khorasani M, Mohammadpourhodki R, Mohammed S, Mohammed JA, Mohebi F, Molokhia M, Monasta L, Moodley Y, Moosazadeh M, Moradi M, Moradi G, Moradi-Lakeh M, Moradpour F, Morawska L, Velásquez IM, Morisaki N, Morrison SD, Mossie TB, Muluneh AG, Murthy S, Musa KI, Mustafa G, Nabhan AF, Nagarajan AJ, Naik G, Naimzada MD, Najafi F, Nangia V, Nascimento BR, Naserbakht M, Nayak V, Ndwandwe DE, Negoi I, Ngunjiri JW, Nguyen CT, Thi Nguyen HL, Nikbakhsh R, Anggraini Ningrum DN, Nnaji CA, Nyasulu PS, Ogbo FA, Oghenetega OB, Oh IH, Okunga EW, Olagunju AT, Olagunju TO, Bali AO, Onwujekwe OE, Asante KO, Orpana HM, Ota E, Otstavnov N, Otstavnov SS, A MP, Padubidri JR, Pakhale S, Pakshir K, Panda-Jonas S, Park EK, Patel SK, Pathak A, Pati S, Patton GC, Paulos K, Peden AE, Filipino Pepito VC, Pereira J, Pham HQ, Phillips MR, Pinheiro M, Polibin RV, Polinder S, Poustchi H, Prakash S, Angga Pribadi DR, Puri P, Syed ZQ, Rabiee M, Rabiee N, Radfar A, Rafay A, Rafiee A, Rafiei A, Rahim F, Rahimi S, Rahimi-Movaghar V, Rahman MA, Rajabpour-Sanati A, Rajati F, Rakovac I, Ranganathan K, Rao SJ, Rashedi V, Rastogi P, Rathi P, Rawaf S, Rawal L, Rawassizadeh R, Renjith V, N Renzaho AM, Resnikoff S, Rezapour A, Ribeiro AI, Rickard J, Rios González CM, Ronfani L, Roshandel G, Saad AM, Sabde YD, Sabour S, Saddik B, Safari S, Safari-Faramani R, Safarpour H, Safdarian M, Sajadi SM, Salamati P, Salehi F, Zahabi SS, Rashad Salem MR, Salem H, Salman O, Salz I, Samy AM, Sanabria J, Riera	UNFOCUSED

	LS, Santric Milicevic MM, Sarker AR, Sarveazad A, Sathian B, Sawhney M, Sawyer SM, Saxena S, Sayyah M, Schwebel DC, Seedat S, Senthilkumaran S, Sepanlou SG, Seyedmousavi S, Sha F, Shaahmadi F, Shahabi S, Shaikh MA, Shams-Beyranvand M, Shamsizadeh M, Sharif-Alhoseini M, Sharifi H, Sheikh A, Shigematsu M, Shin JI, Shiri R, Siabani S, Sigfusdottir ID, Singh PK, Singh JA, Sinha DN, Smarandache CG, R Smith EU, Soheili A, Soleymani B, Soltanian AR, Soriano JB, Sorrie MB, Soyiri IN, Stein DJ, Stokes MA, Sufiyan MB, Rasul Suleria HA, Sykes BL, Tabarés-Seisdedos R, Tabb KM, Taddele BW, Tadesse DB, Tamiru AT, Tarigan IU, Tefera YM, Tehrani-Banihashemi A, Tekle MG, Tekulu GH, Tesema AK, Tesfay BE, Thapar R, Tilahune AB, Tlaye KG, Tohidinik HR, Topor- Madry R, Tran BX, Tran KB, Tripathy JP, Tsai AC, Car LT, Ullah S, Ullah I, Umar M, Unnikrishnan B, Upadhyay E, Uthman OA, Valdez PR, Vasankari TJ, Venketasubramanian N, Violante FS, Vlassov V, Waheed Y, Weldesamuel GT, Werdecker A, Wiangkham T, Wolde HF, Woldeyes DH, Wondafrash DZ, Wondmeneh TG, Wondmieneh AB, Wu AM, Yadav R, Yadollahpour A, Yano Y, Yaya S, Yazdi-Feyzabadi V, Yip P, Yisma E, Yonemoto N, Yoon SJ, Youm Y, Younis MZ, Yousefi Z, Yu Y, Yu C, Yusefzadeh H, Moghadam TZ, Zaidi Z, Zaman SB, Zamani M, Zamanian M, Zandian H, Zarei A, Zare F, Zhang ZJ, Zhang Y, Zodpey S, Dandona L, Dandona R, Degenhardt L, Dharmaratne SD, Hay SI, Mokdad AH, Reiner RC Jr, Sartorius B, Vos T. Global injury morbidity and mortality from 1990 to results from the Global Burden of Disease Study 2017. Inj Prev. 2020 Oct;26(Supp 1):i96-i114. doi: 10.1136/injuryprev-2019-043494. Epub 2020 Apr 24. Erratum in: Inj Prev. 2020 Oct;26(Supp 1):i165. doi: 10.1136/injuryprev-2019-043494corr1. PMID: 32332142; PMCID: PMC7571366.	
355	Tripp DA, Mihajlovic V, Fretz K, Fervaha G, Izard J, Corby R, Siemens DR. Quality of life, depression, and psychosocial mechanisms of suicide risk in prostate cancer. Can Urol Assoc J. 2020 Oct;14(10):E487-E492. doi: 10.5489/cuaj.6310. PMID: 32432532; PMCID: PMC7716829.	UNFOCUSED
356	McLeod SL, Thompson C, Borgundvaag B, Thabane L, Ovens H, Scott S, Ahmed T, Grewal K, McCarron J, Filsinger B, Mittmann N, Worster A, Agoritsas T, Bullard M, Guyatt G. Consistency of triage scores by presenting complaint pre- and post- implementation of a real-time electronic triage decision support tool. J Am Coll Emerg Physicians Open. 2020 Apr 21;1(5):747-756. doi: 10.1002/emp2.12062. PMID: 33145515; PMCID: PMC7593433.	UNFOCUSED
357	Jidveian Popescu M, Stoicea MC, Marinescu I, Cismaşiu RS, Stovicek PO, Tudose C, Ciobanu AM. Depression and anxiety in recurrent giant cell tumor of bone. Rom J Morphol Embryol. 2020 Oct-Dec;61(4):1057-1065. doi: 10.47162/RJME.61.4.08. PMID: 34171055; PMCID: PMC8343577.	UNFOCUSED
358	Jani M, Birlie Yimer B, Sheppard T, Lunt M, Dixon WG. Time trends and prescribing patterns of opioid drugs in UK primary care patients with non-cancer pain: A retrospective cohort study. PLoS Med. 2020 Oct 15;17(10):e1003270. doi: 10.1371/journal.pmed.1003270. PMID: 33057368; PMCID: PMC7561110.	UNFOCUSED
359	Boros F, Vécsei L. Progress in the development of kynurenine and quinoline-3-carboxamide-derived drugs. Expert Opin Investig Drugs. 2020 Nov;29(11):1223-1247. doi: 10.1080/13543784.2020.1813716. Epub 2020 Sep 2. PMID: 32819186.	UNFOCUSED
360	Xu Q, Jia S, Fukasawa M, Lin L, Na J, Mu Z, Li B, Li N, Zhao T, Ju Z, He M, Yu L, Kawakami N, Li Y, Jiang C. A cross-sectional study on associations of physical symptoms, health self-efficacy, and suicidal ideation among Chinese hospitalized cancer patients. BMC Psychiatry. 2020 Nov 19;20(1):544. doi: 10.1186/s12888-020-02945-x. PMID: 33213416; PMCID: PMC7678141.	LUMPING
361	Wolfe F, Ablin J, Baker JF, Diab R, Guymer EK, Littlejohn GO, Michaud K, Rasker JJ, Walitt B, Häuser W. All-cause and cause-specific mortality in persons with fibromyalgia and widespread pain: An observational study in 35,248 persons with rheumatoid arthritis, non-inflammatory rheumatic disorders and clinical fibromyalgia. Semin Arthritis Rheum. 2020 Dec;50(6):1457-1464. doi: 10.1016/j.semarthrit.2020.02.005. Epub 2020 Feb 16. PMID: 32173059.	UNFOCUSED
362	Akechi T, Okuyama T, Uchida M, Kubota Y, Hasegawa T, Suzuki N, Komatsu H, Kusumoto S, Iida S. Factors associated with suicidal ideation in patients with multiple myeloma. Jpn J Clin Oncol. 2020 Dec 16;50(12):1475-1478. doi: 10.1093/jjco/hyaa143. PMID: 32779718.	LUMPING

363	Carreira H, Williams R, Funston G, Stanway S, Bhaskaran K. Associations between breast cancer survivorship and adverse mental health outcomes: A matched population-based cohort study in the United Kingdom. PLoS Med. 2021 Jan 7;18(1):e1003504. doi: 10.1371/journal.pmed.1003504. PMID: 33411711; PMCID: PMC7822529.	UNFOCUSED
364	Bhat TS, Herbosa CM, Rosenberg AR, Sogade O, Jeffe DB, Mehta-Shah N, Semenov YR, Musiek AC. Current measures are not sufficient: an interview-based qualitative assessment of quality of life in cutaneous T-cell lymphoma. Br J Dermatol. 2021 Feb;184(2):310-318. doi: 10.1111/bjd.19298. Epub 2020 Aug 2. PMID: 32510571; PMCID: PMC7722174.	UNFOCUSED
365	Goldman-Mellor S, Hall C, Cerdá M, Bhat H. Firearm suicide mortality among emergency department patients with physical health problems. Ann Epidemiol. 2021 Feb;54:38-44.e3. doi: 10.1016/j.annepidem.2020.09.007. Epub 2020 Sep 18. PMID: 32950655; PMCID: PMC8898069	UNFOCUSED
366	Katzman JG, Katzman JW. COVID-19 Has Provided 20/20 Vision Illuminating Our Nation's Health Crises. Pain Med. 2021 Feb 4;22(1):6-9. doi: 10.1093/pm/pnaa357. PMID: 32986827; PMCID: PMC7543634.	UNFOCUSED
367	Shahar G. Interdisciplinarity and Integration: An Introduction to the Special Issue on Psychopathology in Medical Settings. J Clin Psychol Med Settings. 2021 Mar;28(1):1-5. doi: 10.1007/s10880-020-09752-2. Epub 2020 Nov 20. PMID: 33219478; PMCID: PMC7678582.	UNFOCUSED
368	You DS, Mardian AS, Darnall BD, Chen CA, De Bruyne K, Flood PD, Kao MC, Karnik AD, McNeely J, Porter JG, Schwartz RP, Stieg RL, Mackey SC. A Brief Screening Tool for Opioid Use Disorder: EMPOWER Study Expert Consensus Protocol. Front Med (Lausanne). 2021 Mar 31;8:591201. doi: 10.3389/fmed.2021.591201. PMID: 33869240; PMCID: PMC8044786.	UNFOCUSED
369	Aziato L, Pwavra JBP, Paarima Y, Konlan KD. The Nurse or Midwife at the Crossroads of Caring for Patients With Suicidal and Rigid Religious Ideations in Africa. Front Psychol. 2021 Apr 27;12:549766. doi: 10.3389/fpsyg.2021.549766. PMID: 33986704; PMCID: PMC8110727.	UNFOCUSED
370	Tulandi T. Endometriosis and Pelvic Pain Awareness: Infertility, Suicidal Ideation, and Cancer. J Obstet Gynaecol Can. 2021 May;43(5):543-544. doi: 10.1016/j.jogc.2021.03.001. Epub 2021 Mar 4. PMID: 33676039.	UNFOCUSED
371	Ezard N, Clifford B, Dunlop A, Bruno R, Carr A, Liu Z, Siefried KJ, Lintzeris N. Safety and tolerability of oral lisdexamfetamine in adults with methamphetamine dependence: a phase-2 dose-escalation study. BMJ Open. 2021 May 18;11(5):e044696. doi: 10.1136/bmjopen-2020-044696. PMID: 34006547; PMCID: PMC8137170.	UNFOCUSED
372	Nnomadim OP, Bustamante Helfrich B. Complications of Neurofibromatosis 1 (NF1) in an Adult With Multiple Comorbidities. Cureus. 2021 Jul 20;13(7):e16512. doi: 10.7759/cureus.16512. PMID: 34430126; PMCID: PMC8375605.	UNFOCUSED
373	Lyon ME, Cheng YI, Needle J, Friebert S, Baker JN, Jiang J, Wang J. The intersectionality of gender and poverty on symptom suffering among adolescents with cancer. Pediatr Blood Cancer. 2021 Aug;68(8):e29144. doi: 10.1002/pbc.29144. Epub 2021 Jun 1. Erratum in: Pediatr Blood Cancer. 2021 Nov;68(11):e29348. doi: 10.1002/pbc.29348. PMID: 34061435; PMCID: PMC8406702	UNFOCUSED
374	Kułak-Bejda A, Bejda G, Waszkiewicz N. Mental Disorders, Cognitive Impairment and the Risk of Suicide in Older Adults. Front Psychiatry. 2021 Aug 25;12:695286. doi: 10.3389/fpsyt.2021.695286. PMID: 34512415; PMCID: PMC8423910.	UNFOCUSED
375	Choi Y, Park EC. Suicide after cancer diagnosis in South Korea: a population-based cohort study. BMJ Open. 2021 Sep 2;11(9):e049358. doi: 10.1136/bmjopen-2021-049358. PMID: 34475169; PMCID: PMC8413965.	UNFOCUSED
376	Han X, Hu X, Zhao J, Ma J, Jemal A, Yabroff KR. Trends of Cancer-Related Suicide in the United States: 1999-2018. J Natl Cancer Inst. 2021 Sep 4;113(9):1258-1262. doi: 10.1093/jnci/djaa183. PMID: 33464295.	UNFOCUSED
377	Singh J, Mehta V. When intra-operative exploration is the only option, severe medically refractory trigeminal neuralgia. J Community Hosp Intern Med Perspect. 2021 Sep 20;11(5):686-688. doi: 10.1080/20009666.2021.1944570. PMID: 34567465; PMCID: PMC8462915.	UNFOCUSED

378	Julião M, Chochinov HM, Samorinha C, da Silva Soares D, Antunes B. Prevalence and Factors Associated With Will-to-Live in Patients With Advanced Disease: Results From a Portuguese Retrospective Study. J Pain Symptom Manage. 2021 Oct;62(4):820-827. doi: 10.1016/j.jpainsymman.2021.02.018. Epub 2021 Feb 22. PMID: 33631327.	UNFOCUSED
379	Bouquet E, Pain S, Eiden C, Jouanjus E, Richard N, Fauconneau B, Pérault- Pochat MC; French Addictovigilance Network. Adverse events of recreational cannabis use reported to the French addictovigilance network (2012-2017). Br J Clin Pharmacol. 2021 Oct;87(10):3925-3937. doi: 10.1111/bcp.14812. Epub 2021 Apr 19. PMID: 34282851.	UNFOCUSED
380	Sullivan MD, Ballantyne JC. The Right to Pain Relief: Its Origins in End- of-life Care and Extension to Chronic Pain Care. Clin J Pain. 2021 Oct 26;38(1):58-63. doi: 10.1097/AJP.0000000000001000. PMID: 34699404.	UNFOCUSED
381	GBD 2019 Adolescent Mortality Collaborators. Global, regional, and national mortality among young people aged 10-24 years, 1950-a systematic analysis for the Global Burden of Disease Study 2019. Lancet. 2021 Oct 30;398(10311):1593-1618. doi: 10.1016/S0140-6736(21)01546-4. Epub 2021 Oct 28. Erratum in: Lancet. 2022 Feb 26;399(10327):802. doi: 10.1016/S0140-6736(22)00320-8. PMID: 34755628; PMCID: PMC8576274.	UNFOCUSED
382	Nugent SM, Morasco BJ, Handley R, Clayburgh D, Hooker ER, Ganzini L, Knight SJ, Chen JI, Sullivan DR, Slatore CG. Risk of Suicidal Self-directed Violence Among US Veteran Survivors of Head and Neck Cancer. JAMA Otolaryngol Head Neck Surg. 2021 Nov 1;147(11):981-989. doi: 10.1001/jamaoto.2021.2625. Erratum in: JAMA Otolaryngol Head Neck Surg. 2022 Feb 1;148(2):200. doi: 10.1001/jamaoto.2021.3852. PMID: 34617963; PMCID: PMC8498929.	INCLUDED
383	Roy R, Sommer JL, Bolton JM, El-Gabalawy R. Understanding correlates of suicidality among those with usual pain and discomfort: A Canadian nationally representative study. J Psychosom Res. 2021 Dec;151:110651. doi: 10.1016/j.jpsychores.2021.110651. Epub 2021 Oct 14. PMID: 34673350.	UNFOCUSED
384	Men YV, Lam TC, Yeung CY, Yip PSF. Understanding the impact of clinical characteristics and healthcare utilizations on suicide among cancer sufferers: a case-control study in Hong Kong. Lancet Reg Health West Pac. 2021 Oct 21;17:100298. doi: 10.1016/j.lanwpc.2021.100298. PMID: 34734204; PMCID: PMC8551816.	UNFOCUSED
385	Okereke OI, Vyas CM, Mischoulon D, Chang G, Cook NR, Weinberg A, Bubes V, Copeland T, Friedenberg G, Lee IM, Buring JE, Reynolds CF 3rd, Manson JE. Effect of Long-term Supplementation With Marine Omega-3 Fatty Acids vs Placebo on Risk of Depression or Clinically Relevant Depressive Symptoms and on Change in Mood Scores: A Randomized Clinical Trial. JAMA. 2021 Dec 21;326(23):2385-2394. doi: 10.1001/jama.2021.21187. PMID: 34932079; PMCID: PMC8693224.	UNFOCUSED
386	Kim TE, Lee RG, Park SY, Oh IH. Measuring Trends in the Socioeconomic Burden of Disease in Korea, 2007-2015. J Prev Med Public Health. 2022 Jan;55(1):19-27. doi: 10.3961/jpmph.21.594. Epub 2022 Jan 31. PMID: 35135045; PMCID: PMC8841198.	UNFOCUSED
387	Stukalin I, Olaiya OR, Naik V, Wiebe E, Kekewich M, Kelly M, Wilding L, Halko R, Oczkowski S. Medications and dosages used in medical assistance in dying: a cross-sectional study. CMAJ Open. 2022 Jan 18;10(1):E19-E26. doi: 10.9778/cmajo.20200268. PMID: 35042691; PMCID: PMC8920593.	UNFOCUSED
388	Hall AL, Sweet J, Tweel M, MacLean MB. Comparing negative health indicators in male and female veterans with the Canadian general population. BMJ Mil Health. 2022 Feb;168(1):82-87. doi: 10.1136/bmjmilitary-2020-001526. Epub 2020 Aug 30. PMID: 32868291; PMCID: PMC8788043.	UNFOCUSED
389	Senf B, Maiwurm P, Fettel J. Attitudes and opinions towards suicidality in professionals working with oncology patients: results from an online survey. Support Care Cancer. 2022 Feb;30(2):1775-1786. doi: 10.1007/s00520-021-06590-2. Epub 2021 Oct 1. PMID: 34599381; PMCID: PMC8727409.	UNFOCUSED

390	Mullins N, Kang J, Campos AI, Coleman JRI, Edwards AC, Galfalvy H, Levey DF, Lori A, Shabalin A, Starnawska A, Su MH, Watson HJ, Adams M, Awasthi S, Gandal M, Hafferty JD, Hishimoto A, Kim M, Okazaki S, Otsuka I, Ripke S, Ware EB, Bergen AW, Berrettini WH, Bohus M, Brandt H, Chang X, Chen WJ, Chen HC, Crawford S, Crow S, DiBlasi E, Duriez P, Fernández-Aranda F, Fichter MM, Gallinger S, Glatt SJ, Gorwood P, Guo Y, Hakonarson H, Halmi KA, Hwu HG, Jain S, Jamain S, Jiménez-Murcia S, Johnson C, Kaplan AS, Kaye WH, Keel PK, Kennedy JL, Klump KL, Li D, Liao SC, Lieb K, Lilenfeld L, Liu CM, Magistretti PJ, Marshall CR, Mitchell JE, Monson ET, Myers RM, Pinto D, Powers A, Ramoz N, Roepke S, Rozanov V, Scherer SW, Schmahl C, Sokolowski M, Strober M, Thornton LM, Treasure J, Tsuang MT, Witt SH, Woodside DB, Yilmaz Z, Zillich L, Adolfsson R, Agartz I, Air TM, Alda M, Alfredsson L, Andreassen OA, Anjorin A, Appadurai V, Soler Artigas M, Van der Auwera S, Azevedo MH, Bass N, Bau CHD, Baune BT, Bellivier F, Berger K, Biernacka JM, Bigdeli TB, Binder EB, Boehnke M, Boks MP, Bosch R, Braff DL, Bryant R, Budde M, Byrne EM, Cahn W, Casas M, Castelao E, Cervilla JA, Chaumette B, Cichon S, Corvin A, Craddock N, Craig D, Degenhardt F, Djurovic S, Edenberg HJ, Fanous AH, Foo JC, Forstner AJ, Frye M, Fullerton JM, Gatt JM, Gejman PV, Giegling I, Grabe HJ, Green MJ, Grevet EH, Grigoroiu-Serbanescu M, Gutierrez B, Guzman-Parra J, Hamilton SP, Hamshere ML, Hartmann A, Hauser J, Heilmann-Heimbach S, Hoffmann P, Ising M, Jones I, Jones LA, Jonsson L, Kahn RS, Kelsoe JR, Kendler KS, Kloiber S, Koenen KC, Kogevinas M, Konte B, Krebs MO, Landén M, Lawrence J, Leboyer M, Lee PH, Levinson DF, Liao C, Lissowska J, Lucae S, Mayoral F, McElroy SL, McGrath P, McGuffin P, McQuillin A, Medland SE, Mehta D, Melle I, Milaneschi Y, Mitchell PB, Molina E, Morken G, Mortensen PB, Müller- Myhsok B, Nievergelt C, Nimgaonkar V, Nöthen MM, O'Donovan MC, Ophoff RA, Owen MJ, Pato C, Pato MT, Penninx BWJH, Pimm J, Pistis G, Potash JB, Power RA, Preisig M, Quested D, Ramos-Quiroga JA, Reif A, Ribasés M, Richarte V, Rietschel M, Rivera M, Roberts A, Roberts G, Rouleau GA, Rovaris DL, Rujescu D, Sánchez- Mora C, Sanders AR, Schofield PR, Schulze TG, Scott LJ, Serretti A, Shi J, Shyn SI, Sirignano L, Sklar P, Smeland OB, Smoller JW, Sonuga-Barke EJS, Spalletta G, Strauss JS, Świątkowska B, Trzaskowski M, Turecki G, Vilar-Ribó L, Vincent JB, Völzke H, Walters JTR, Shannon Weickert C, Weickert TW, Weissman MM, Williams LM, Wray NR, Zai CC, Ashley-Koch AE, Beckham JC, Hauser ER, Hauser MA, Kimbrel NA, Lindquist JH, McMahon B, Oslin DW, Qin X; Major Depressive Disorder Working Group of the Psychiatric Genomics Consortium; Bipolar Disorder Working Group of the Psychiatric Genomics Consortium; Eating Disorders Working Group of the Psychiatric Genomics Consortium; German Borderline Genomics Consortium; MVP Suicide Exemplar Workgroup; VA Million Veteran Program; Agerbo E, Børglum AD, Breen G, Erlangsen A, Esko T, Gelernter J, Hougaard DM, Kessler RC, Kranzler HR, Li QS, Martin NG, McIntosh AM, Mors O, Nordentoft M, Olsen CM, Porteous D, Ursano RJ, Wasserman D, Werge T, Whiteman DC, Bulik CM, Coon H, Demontis D, Docherty AR, Kuo PH, Lewis CM, Mann JJ, Rentería ME, Smith DJ, Stahl EA, Stein MB, Streit F, Willour V, Ruderfer DM. Dissecting the Shared Genetic Architecture of Suicide Attempt, Psychiatric Disorders, and Known Risk Factors. Biol Psychiatry. 2022 Feb 1;91(3):313-327. doi: 10.1016/j.biopsych.2021.05.029. Epub 2021 Sep 9. PMID: 34861974; PMCID: PMC8851871.	UNFOCUSED
391	Kremeike K, Dojan T, Boström K, Voltz R. Umgang mit Todeswünschen in der Palliativversorgung [Dealing with Desire to Die in Palliative Care]. Ther Umsch. 2022 Feb;79(1):61-66. German. doi: 10.1024/0040-5930/a001329. PMID: 35100826	UNFOCUSED
392	Humphreys K, Shover CL, Andrews CM, Bohnert ASB, Brandeau ML, Caulkins JP, Chen JH, Cuéllar MF, Hurd YL, Juurlink DN, Koh HK, Krebs EE, Lembke A, Mackey SC, Larrimore Ouellette L, Suffoletto B, Timko C. Responding to the opioid crisis in North America and beyond: recommendations of the Stanford-Lancet Commission. Lancet. 2022 Feb 5;399(10324):555-604. doi: 10.1016/S0140-6736(21)02252-2. Epub 2022 Feb 2. PMID: 35122753; PMCID: PMC9261968.	UNFOCUSED
393	Chen H, He Q, Zeng Y, Wang L, Yu H, Yin W, Jiang Y, Liu L. Feeling like the sky is falling down: Experiences of parents of adolescents diagnosed with cancer in one-child families in China - A qualitative study. J Clin Nurs. 2022 Mar;31(5-6):733-743. doi: 10.1111/jocn.15913. Epub 2021 Jul 13. PMID: 34258803.	LUMPING
394	Chen W, Chen Y, Xiao H. Existential Distress in Cancer Patients: A Concept Analysis. Cancer Nurs. 2022 Mar-Apr 01;45(2):E471-E486. doi: 10.1097/NCC.0000000000000925. PMID: 35174696.	UNFOCUSED
395	Wahab S, Chua TY, Razali R, Mat Saher Z, Zamzam IH, Bujang MA. Suicidal Behavior Among Elderly Inpatients: its Relation to Functional Disability and Pain. Psychol Res Behav Manag. 2022 Mar 24;15:737-750. doi: 10.2147/PRBM.S341768. PMID: 35356540; PMCID: PMC8959869.	UNFOCUSED

396	Parke SC, Langelier DM, Cheng JT, Kline-Quiroz C, Stubblefield MD. State of Rehabilitation Research in the Head and Neck Cancer Population: Functional Impact vs. Impairment-Focused Outcomes. Curr Oncol Rep. 2022 Apr;24(4):517-532. doi: 10.1007/s11912-022-01227-x. Epub 2022 Feb 19. PMID: 35182293.	UNFOCUSED
397	Coon H, Shabalin A, Bakian AV, DiBlasi E, Monson ET, Kirby A, Chen D, Fraser A, Yu Z, Staley M, Callor WB, Christensen ED, Crowell SE, Gray D, Crockett DK, Li QS, Keeshin B, Docherty AR. Extended familial risk of suicide death is associated with younger age at death and elevated polygenic risk of suicide. Am J Med Genet B Neuropsychiatr Genet. 2022 Apr;189(3-4):60-73. doi: 10.1002/ajmg.b.32890. Epub 2022 Feb 24. PMID: 35212135; PMCID: PMC9149029.	UNFOCUSED
398	Pakniyat-Jahromi S, Amazan R, Garrels E, Zamiri A, Galo AR, Gunturu S. Treatment Modalities for Chronic Pain in Elderly Patients With Depression: A Systematic Review. Prim Care Companion CNS Disord. 2022 May 24;24(3):21r03097. doi: 10.4088/PCC.21r03097. PMID: 35621831.	UNFOCUSED
399	Zhang L, Cai H, Bai W, Zou SY, Feng KX, Li YC, Liu HZ, Du X, Zeng ZT, Lu CM, Zhang L, Mi WF, Ding YH, Yang JJ, Jackson T, Cheung T, Su Z, An FR, Xiang YT. Prevalence of suicidality in clinically stable patients with major depressive disorder during the COVID-19 pandemic. J Affect Disord. 2022 Jun 15;307:142-148. doi: 10.1016/j.jad.2022.03.042. Epub 2022 Mar 22. PMID: 35337925; PMCID: PMC8938301.	UNFOCUSED
400	Fernández-Ginés FD, Cortiñas-Sáenz M, Selva-Sevilla C, Gerónimo-Pardo M. Sevoflurane topical analgesia for intractable pain with suicidal ideation. BMJ Support Palliat Care. 2022 Jul;12(e2):e192-e193. doi: 10.1136/bmjspcare-2019-002023. Epub 2020 Jun 29. PMID: 32601149.	CASE
401	Molla A, Aderaw M, Mulat H, Fanta B, Nenko G, Adane A. Suicidal ideation, attempt and associated factors among people living with cancer in Ethiopia: a cross-sectional study. Ann Gen Psychiatry. 2022 Jul 26;21(1):28. doi: 10.1186/s12991-022-00407-0. PMID: 35883094; PMCID: PMC9316727.	UNFOCUSED
402	Corwell BN, Motov SM, Davis NL, Kim HK. Novel uses of ketamine in the emergency department. Expert Opin Drug Saf. 2022 Aug;21(8):1009-1025. doi: 10.1080/14740338.2022.2100883. Epub 2022 Jul 21. PMID: 35822534.	UNFOCUSED
403	Vasavda C, Xu R, Liew J, Kothari R, Dhindsa RS, Semenza ER, Paul BD, Green DP, Sabbagh MF, Shin JY, Yang W, Snowman AM, Albacarys LK, Moghekar A, Pardo- Villamizar CA, Luciano M, Huang J, Bettegowda C, Kwatra SG, Dong X, Lim M, Snyder SH. Identification of the NRF2 transcriptional network as a therapeutic target for trigeminal neuropathic pain. Sci Adv. 2022 Aug 5;8(31):eabo5633. doi: 10.1126/sciadv.abo5633. Epub 2022 Aug 3. PMID: 35921423; PMCID: PMC9348805.	IN VITRO
404	Yamada K, Fujii T, Kubota Y, Ikeda T, Hanazato M, Kondo N, Matsudaira K, Kondo K. Prevalence and municipal variation in chronic musculoskeletal pain among independent older people: data from the Japan Gerontological Evaluation Study (JAGES). BMC Musculoskelet Disord. 2022 Aug 5;23(1):755. doi: 10.1186/s12891-022-05694-y. PMID: 35932026; PMCID: PMC9356514.	UNFOCUSED
405	Huang S, Lewis MO, Bao Y, Adekkanattu P, Adkins LE, Banerjee S, Bian J, Gellad WF, Goodin AJ, Luo Y, Fairless JA, Walunas TL, Wilson DL, Wu Y, Yin P, Oslin DW, Pathak J, Lo-Ciganic WH. Predictive Modeling for Suicide-Related Outcomes and Risk Factors among Patients with Pain Conditions: A Systematic Review. J Clin Med. 2022 Aug 17;11(16):4813. doi: 10.3390/jcm11164813. PMID: 36013053; PMCID: PMC9409905.	REVIEW
406	Anderson D, Razzak AN, McDonald M, Cao D, Hasoon J, Viswanath O, Kaye AD, Urits I. Mental Health in Urologic Oncology. Health Psychol Res. 2022 Aug 20;10(3):37518. doi: 10.52965/001c.37518. PMID: 35999977; PMCID: PMC9392841.	UNFOCUSED
407	Bahnsen MK, Graugaard C, Andersson M, Andresen JB, Frisch M. Physical and Mental Health Problems and Their Associations With Inter-Personal Sexual Inactivity and Sexual Dysfunctions in Denmark: Baseline Assessment in a National Cohort Study. J Sex Med. 2022 Oct;19(10):1562-1579. doi: 10.1016/j.jsxm.2022.07.004. Epub 2022 Aug 13. PMID: 35970709.	UNFOCUSED
408	Raghubar KP, Chambers T, Hill RM, Taylor O, Hockenberry M, Hooke MC, Mitby P, Moore IK, Brown AL, Scheurer ME. Longitudinal investigation of suicidal ideation and associated factors during pediatric acute lymphoblastic leukemia chemotherapy. Psychooncology. 2022 Oct;31(10):1782-1789. doi: 10.1002/pon.6014. Epub 2022 Aug 27. PMID: 35986586; PMCID: PMC10754031.	LUMPING

409	Sáenz-Aldea M, Zarrabeitia MT, García Blanco A, Santurtún A. Scrutinizing the Profile and Risk Factors of Suicide: A Perspective from a Case-Control Study Focused on a Northern Region of Spain. Int J Environ Res Public Health. 2022 Nov 29;19(23):15867. doi: 10.3390/ijerph192315867. PMID: 36497948; PMCID: PMC9741352.	CASE
410	Faley B, Brooks A, Vartan CM, DiScala SL. Impact of Clinical Pharmacist Practitioner-Driven High Opioid Dose Reevaluation in Veterans with Chronic Non- Cancer Pain. J Pain Palliat Care Pharmacother. 2022 Dec;36(4):249-259. doi: 10.1080/15360288.2022.2113594. Epub 2022 Aug 31. PMID: 36044721.	UNFOCUSED
411	Liu T, Liu W, Leung A, Jia S, Lee P, Liu L, Mutsaers A, Miller S, Honarmand K, Malik S, Qu M, Ball I. Medical Assistance in Dying in Oncology Patients: A Canadian Academic Hospital's Experience. Curr Oncol. 2022 Dec 1;29(12):9407-9415. doi: 10.3390/curroncol29120739. PMID: 36547153; PMCID: PMC9777282.	UNFOCUSED
412	Lozupone M, Donghia R, Sardone R, Mollica A, Berardino G, Lampignano L, Griseta C, Zupo R, Castellana F, Bortone I, Dibello V, Resta E, Stallone R, Seripa D, Daniele A, Solfrizzi V, Altamura M, Bellomo A, Panza F. Apolipoprotein E genotype, inflammatory biomarkers, and non-psychiatric multimorbidity contribute to the suicidal ideation phenotype in older age. The Salus in Apulia Study. J Affect Disord. 2022 Dec 15;319:202-212. doi: 10.1016/j.jad.2022.09.046. Epub 2022 Sep 23. PMID: 36155237.	UNFOCUSED
413	Rakovec M, Zhu W, Khalafallah AM, Salvatori R, Hamrahian AH, Gallia GL, Ishii M, London NR Jr, Ramanathan M Jr, Rowan NR, Mukherjee D. Patient reported outcomes and treatment satisfaction in patients with cushing syndrome. Endocrine. 2023 Jan;79(1):161-170. doi: 10.1007/s12020-022-03214-5. Epub 2022 Oct 13. PMID: 36227510.	UNFOCUSED
414	Onyeka TC, Onu JU, Agom DA. Psychosocial aspects of adult cancer patients: A scoping review of sub-Saharan Africa. Psychooncology. 2023 Jan;32(1):86-106. doi: 10.1002/pon.6052. Epub 2022 Oct 21. PMID: 36250212.	REVIEW
415	Jacobs J, Jacobs P. Judaism. Cancer Treat Res. 2023;187:237-259. doi: 10.1007/978-3-031-29923-0_17. PMID: 37851231.	UNFOCUSED
416	Quah ELY, Chua KZY, Lua JK, Wan DWJ, Chong CS, Lim YX, Krishna L. A Systematic Review of Stakeholder Perspectives of Dignity and Assisted Dying. J Pain Symptom Manage. 2023 Feb;65(2):e123-e136. doi: 10.1016/j.jpainsymman.2022.10.004. Epub 2022 Oct 14. PMID: 36244639.	REVIEW
417	Åkeflo L, Elmerstig E, Bergmark K, Dunberger G. Barriers to and strategies for dealing with vaginal dilator therapy - Female pelvic cancer survivors' experiences: A qualitative study. Eur J Oncol Nurs. 2023 Feb;62:102252. doi: 10.1016/j.ejon.2022.102252. Epub 2022 Dec 10. PMID: 36603495.	UNFOCUSED
418	Murri MB, Caruso R, Christensen AP, Folesani F, Nanni MG, Grassi L. The facets of psychopathology in patients with cancer: Cross-sectional and longitudinal network analyses. J Psychosom Res. 2023 Feb;165:111139. doi: 10.1016/j.jpsychores.2022.111139. Epub 2022 Dec 27. PMID: 36610333.	UNFOCUSED
419	Campos AI, Garcia-Marin LM, Christensen H, Batterham PJ, van Velzen LS, Schmaal L; International Suicide Genetics Consortium; Rabinowitz JA, Jahanshad N, Martin NG, Cuellar-Partida G, Ruderfer D, Mullins N, Rentería ME. Genomics- driven screening for causal determinants of suicide attempt. Aust N Z J Psychiatry. 2023 Mar;57(3):423-431. doi: 10.1177/00048674221091499. Epub 2022 Apr 11. PMID: 35403454.	IN VITRO
420	Straus LD, An X, Ji Y, McLean SA, Neylan TC; AURORA Study Group; Cakmak AS, Richards A, Clifford GD, Liu M, Zeng D, House SL, Beaudoin FL, Stevens JS, Linnstaedt SD, Germine LT, Bollen KA, Rauch SL, Haran JP, Storrow AB, Lewandowski C, Musey PI, Hendry PL, Sheikh S, Jones CW, Punches BE, Kurz MC, Swor RA, Hudak LA, Seamon MJ, Datner EM, Chang AM, Pearson C, Peak DA, Merchant RC, Domeier RM, Rathlev NK, O'Neil BJ, Sergot P, Sanchez LD, Bruce SE, Miller MW, Pietrzak RH, Joormann J, Barch DM, Pizzagalli DA, Sheridan JF, Harte SE, Elliott JM, Kessler RC, Ressler KJ, Koenen KC. Utility of Wrist-Wearable Data for Assessing Pain, Sleep, and Anxiety Outcomes After Traumatic Stress Exposure. JAMA Psychiatry. 2023 Mar 1;80(3):220-229. doi: 10.1001/jamapsychiatry.2022.4533. PMID: 36630119; PMCID: PMC9857758.	UNFOCUSED

421	Lester EG, Wang KE, Blakeley JO, Vranceanu AM. Occurrence and Severity of Suicidal Ideation in Adults With Neurofibromatosis Participating in a Mind-Body RCT. Cogn Behav Neurol. 2023 Mar 1;36(1):19-27. doi: 10.1097/WNN.0000000000000332. PMID: 36651958.	UNFOCUSED
422	Kessler RC, Bauer MS, Bishop TM, Bossarte RM, Castro VM, Demler OV, Gildea SM, Goulet JL, King AJ, Kennedy CJ, Landes SJ, Liu H, Luedtke A, Mair P, Marx BP, Nock MK, Petukhova MV, Pigeon WR, Sampson NA, Smoller JW, Miller A, Haas G, Benware J, Bradley J, Owen RR, House S, Urosevic S, Weinstock LM. Evaluation of a Model to Target High-risk Psychiatric Inpatients for an Intensive Postdischarge Suicide Prevention Intervention. JAMA Psychiatry. 2023 Mar 1;80(3):230-240. doi: 10.1001/jamapsychiatry.2022.4634. PMID: 36652267; PMCID: PMC9857842.	UNFOCUSED
423	Vissers S, Dierickx S, Deliens L, Mortier F, Cohen J, Chambaere K. Characteristics and outcomes of peer consultations for assisted dying request assessments: Cross-sectional survey study among attending physicians. Front Public Health. 2023 Mar 29;11:1100353. doi: 10.3389/fpubh.2023.1100353. PMID: 37064672; PMCID: PMC10090406.	UNFOCUSED
424	Oveisi N, Khan Z, Brotto LA. A qualitative study of sexual health and function of females with pelvic cancer. Sex Med. 2023 Mar 1;11(2):qfac002. doi: 10.1093/sexmed/qfac002. PMID: 36910701; PMCID: PMC9978583.	UNFOCUSED
425	James KE, Agarwal S, Armenion KL, Clapp C, Barnes A, Ye GY, Zisook S, Davidson JE. A deductive thematic analysis of nurses with job-related problems who completed suicide during the early COVID-19 pandemic: A preliminary report. Worldviews Evid Based Nurs. 2023 Apr;20(2):96-106. doi: 10.1111/wvn.12640. Epub 2023 Mar 29. PMID: 36991524.	UNFOCUSED
426	Zhou JX, Goh C, Chiam M, Krishna LKR. Painting and Poetry From a Bereaved Family and the Caring Physician. J Pain Symptom Manage. 2023 May;65(5):e503-e506. doi: 10.1016/j.jpainsymman.2022.03.008. Epub 2022 Mar 24. PMID: 35339612.	UNFOCUSED
427	Mofatteh M, Mashayekhi MS, Arfaie S, Chen Y, Malhotra AK, Alvi MA, Sader N, Antonick V, Fatehi Hassanabad M, Mansouri A, Das S, Liao X, McIntyre RS, Del Maestro R, Turecki G, Cohen-Gadol AA, Zadeh G, Ashkan K. Suicidal ideation and attempts in brain tumor patients and survivors: A systematic review. Neurooncol Adv. 2023 May 12;5(1):vdad058. doi: 10.1093/noajnl/vdad058. PMID: 37313501; PMCID: PMC10259251.	REVIEW
428	Gisev N, Pearson SA, Dobbins T, Buizen L, Murphy T, Wilson A, Blyth F, Dunlop A, Larney S, Currow DC, Mattick RP, Degenhardt L. Cohort profile: POPPY II - a population-based cohort examining the patterns and outcomes of prescription opioid use in New South Wales, Australia. BMJ Open. 2023 May 17;13(5):e068310. doi: 10.1136/bmjopen-2022-068310. PMID: 37197812; PMCID: PMC10193079.	UNFOCUSED
429	de A Simoes Moreira D, Gauer LE, Teixeira G, Fonseca da Silva AC, Cavalcanti S, Quevedo J. Efficacy and adverse effects of ketamine versus electroconvulsive therapy for major depressive disorder: A systematic review and meta-analysis. J Affect Disord. 2023 Jun 1;330:227-238. doi: 10.1016/j.jad.2023.02.152. Epub 2023 Mar 11. PMID: 36907464; PMCID: PMC10497186.	UNFOCUSED
430	Amjad MA, Siddiqui AM, Bashir K, Ghafoor AU, Durrani RS. Prevalence of chronic pain in Pakistan - a national survey. J Pak Med Assoc. 2023 Jun;73(6):1217-1220. doi: 10.47391/JPMA.6671. PMID: 37427618.	UNFOCUSED
431	Biazus TB, Beraldi GH, Tokeshi L, Rotenberg LS, Dragioti E, Carvalho AF, Solmi M, Lafer B. All-cause and cause-specific mortality among people with bipolar disorder: a large-scale systematic review and meta-analysis. Mol Psychiatry. 2023 Jun;28(6):2508-2524. doi: 10.1038/s41380-023-02109-9. Epub 2023 Jul 25. PMID: 37491460; PMCID: PMC10611575.	UNFOCUSED
432	Xing Y, Zhao W, Duan C, Zheng J, Zhao X, Yang J, Sun N, Chen J. Developing a visual model for predicting depression in patients with lung cancer. J Clin Nurs. 2023 Aug;32(15-16):4614-4625. doi: 10.1111/jocn.16487. Epub 2022 Aug 10. PMID: 35949178.	UNFOCUSED
433	Decazes E, Rigal O, Clatot F. Effect of a single dose of intravenous ketamine on the wish to hasten death in palliative care: A case report in advanced cancer. Palliat Support Care. 2023 Aug;21(4):765-767. doi: 10.1017/S1478951523000317. PMID: 36960611.	UNFOCUSED

434	Cheatle MD, Giordano NA, Themelis K, Tang NKY. Suicidal thoughts and behaviors in patients with chronic pain, with and without co-occurring opioid use disorder. Pain Med. 2023 Aug 1;24(8):941-948. doi: 10.1093/pm/pnad043. PMID: 37014415; PMCID: PMC10391589.	UNFOCUSED
435	Gerson SM, Gamondi C, Wiebe E, Deliens L. Should Palliative Care Teams be Involved in Medical Assisted Dying? J Pain Symptom Manage. 2023 Aug;66(2):e233-e237. doi: 10.1016/j.jpainsymman.2023.04.004. Epub 2023 Apr 16. PMID: 37072103.	UNFOCUSED
436	GBD 2019 Australia Collaborators. The burden and trend of diseases and their risk factors in Australia, 1990-a systematic analysis for the Global Burden of Disease Study 2019. Lancet Public Health. 2023 Aug;8(8):e585-e599. doi: 10.1016/S2468-2667(23)00123-8. Erratum in: Lancet Public Health. 2023 Sep;8(9):e669. doi: 10.1016/S2468-2667(23)00184-6. Erratum in: Lancet Public Health. 2023 Dec;8(12):e914. doi: 10.1016/S2468-2667(23)00251-7. PMID: 37516475; PMCID: PMC10400798.	UNFOCUSED
437	Way BM, Griffin KR, Kraus SW, Tsai J, Pietrzak RH. Erectile Dysfunction in a U.S. National Sample of Male Military Veterans. Mil Med. 2023 Aug 29;188(9-10):2837-2843. doi: 10.1093/milmed/usac187. PMID: 35792506.	UNFOCUSED
438	Solmi M, De Toffol M, Kim JY, Choi MJ, Stubbs B, Thompson T, Firth J, Miola A, Croatto G, Baggio F, Michelon S, Ballan L, Gerdle B, Monaco F, Simonato P, Scocco P, Ricca V, Castellini G, Fornaro M, Murru A, Vieta E, Fusar-Poli P, Barbui C, Ioannidis JPA, Carvalho AF, Radua J, Correll CU, Cortese S, Murray RM, Castle D, Shin JI, Dragioti E. Balancing risks and benefits of cannabis use: umbrella review of meta-analyses of randomised controlled trials and observational studies. BMJ. 2023 Aug 30;382:e072348. doi: 10.1136/bmj-2022-072348. PMID: 37648266; PMCID: PMC10466434.	UNFOCUSED
439	Jackson N, Turner M, Paterson C. What are the holistic care impacts among individuals living through the COVID-19 pandemic in residential or community care settings? An integrative systematic review. Int J Older People Nurs. 2023 Sep;18(5):e12557. doi: 10.1111/opn.12557. Epub 2023 Jun 26. PMID: 37365716.	UNFOCUSED
440	Henry M, Alias A, Bisson-Gervais V, Liu JY, Dargis L, Gauthier L, Tapp D, Greenfield B, Mishara B. Medical assistance in dying in Canada: A scoping review on the concept of suffering. Psychooncology. 2023 Sep;32(9):1339-1347. doi: 10.1002/pon.6196. Epub 2023 Jul 26. PMID: 37496186.	UNFOCUSED
441	Shen F, Zhou X, Guo F, Fan K, Zhou Y, Xia J, Xu Z, Liu Z. Increased risk of postpartum depression in women with lactational mastitis: a cross-sectional study. Front Psychiatry. 2023 Sep 1;14:1229678. doi: 10.3389/fpsyt.2023.1229678. PMID: 37727256; PMCID: PMC10506305.	UNFOCUSED
442	Malgaroli M, Szuhany KL, Riley G, Miron CD, Park JH, Rosenthal J, Chachoua A, Meyers M, Simon NM. Heterogeneity of posttraumatic stress, depression, and fear of cancer recurrence in breast cancer survivors: a latent class analysis. J Cancer Surviv. 2023 Oct;17(5):1510-1521. doi: 10.1007/s11764-022-01195-y. Epub 2022 Feb 28. PMID: 35224684; PMCID: PMC10037701.	UNFOCUSED
443	Wu HM, Chiang CY, Chen WY, Chen CJ, Tseng CC, Chang YC, Cheng WM, Kuan YH. Cyclizine-induced proinflammatory responses through Akt-NFκB pathway in macrophages. Environ Toxicol. 2023 Dec;38(12):2819-2825. doi: 10.1002/tox.23913. Epub 2023 Aug 8. PMID: 37551787.	UNFOCUSED
444	Spencer CN, Khalil M, Herbert M, Aravkin AY, Arrieta A, Baeza MJ, Bustreo F, Cagney J, Calderon-Anyosa RJC, Carr S, Chandan JK, Coll CVN, de Andrade FMD, de Andrade GN, Debure AN, Flor LS, Hammond B, Hay SI, Knaul FN, Lim RQH, McLaughlin SA, Minhas S, Mohr JK, Mullany EC, Murray CJL, O'Connell EM, Patwardhan V, Reinach S, Scott D, Sorenson RJD, Stein C, Stöckl H, Twalibu A, Vasconcelos N, Zheng P, Metheny N, Chandan JS, Gakidou E. Health effects associated with exposure to intimate partner violence against women and childhood sexual abuse: a burden of proof study. Nat Med. 2023 Dec;29(12):3243-3258. doi: 10.1038/s41591-023-02629-5. Epub 2023 Dec 11. PMID: 38081957; PMCID: PMC10719101.	UNFOCUSED
445	Liaw V, McCreary M, Friedman DI. Quality of Life in Patients With Confirmed and Suspected Spinal CSF Leaks. Neurology. 2023 Dec 4;101(23):e2411-e2422. doi: 10.1212/WNL.0000000000207763. Erratum in: Neurology. 2024 Jul 9;103(1):e209596. doi: 10.1212/WNL.0000000000209596. PMID: 37816637; PMCID: PMC10752647.	UNFOCUSED

446	Chen J, Ping Z, Hu D, Wang J, Liu Y. Risk factors associated with suicidal ideation among cancer patients: a systematic review and meta-analysis. Front Psychol. 2024 Jan 8;14:1287290. doi: 10.3389/fpsyg.2023.1287290. PMID: 38259550; PMCID: PMC10800839.	REVIEW
447	Minian N, Gayapersad A, Coroiu A, Dragonetti R, Zawertailo L, Zaheer J, O'Neill B, Lange S, Thomson N, Crawford A, Kennedy SH, Selby P. Prototyping the implementation of a suicide prevention protocol in primary care settings using PDSA cycles: a mixed method study. Front Psychiatry. 2024 Jan 25;15:1286078. doi: 10.3389/fpsyt.2024.1286078. PMID: 38333892; PMCID: PMC10850298.	UNFOCUSED
448	Revuelta-Gutiérrez R, Contreras-Vázquez OR, Piñón-Jiménez F, Martínez-Anda JJ. Trigeminal neuralgia secondary to epidermoid cyst and neurovascular conflict: An illustrative case with literature review. Surg Neurol Int. 2024 Feb 9;15:36. doi: 10.25259/SNI_925_2023. PMID: 38468668; PMCID: PMC10927216.	UNFOCUSED
449	Mani RK, Bhatnagar S, Butola S, Gursahani R, Mehta D, Simha S, Divatia JV, Kumar A, Iyer SK, Deodhar J, Bhat RS, Salins N, Thota RS, Mathur R, Iyer RK, Gupta S, Kulkarni P, Murugan S, Nasa P, Myatra SN. Indian Society of Critical Care Medicine and Indian Association of Palliative Care Expert Consensus and Position Statements for End-of-life and Palliative Care in the Intensive Care Unit. Indian J Crit Care Med. 2024 Mar;28(3):200-250. doi: 10.5005/jp- journals-10071-24661. PMID: 38477011; PMCID: PMC10926026.	UNFOCUSED
450	Agrawal M, Richards W, Beaussant Y, Shnayder S, Ameli R, Roddy K, Stevens N, Richards B, Schor N, Honstein H, Jenkins B, Bates M, Thambi P. Psilocybin- assisted group therapy in patients with cancer diagnosed with a major depressive disorder. Cancer. 2024 Apr 1;130(7):1137-1146. doi: 10.1002/cncr.35010. Epub 2023 Dec 18. PMID: 38105655.	UNFOCUSED
451	Ramaiyer MS, Saad E, Kurt I, Borahay MA. Genetic Mechanisms Driving Uterine Leiomyoma Pathobiology, Epidemiology, and Treatment. Genes (Basel). 2024 Apr 27;15(5):558. doi: 10.3390/genes15050558. PMID: 38790186; PMCID: PMC11121260.	UNFOCUSED
452	Lebwohl MG, Koo JY, Armstrong AW, Strober BE, Martin GM, Rawnsley NN, Goehring EL Jr, Jacobson AA. Brodalumab: 5-Year US Pharmacovigilance Report. Dermatol Ther (Heidelb). 2024 May;14(5):1349-1357. doi: 10.1007/s13555-024-01162-8. Epub 2024 May 9. PMID: 38724839; PMCID: PMC11116300.	UNFOCUSED
453	GBD 2021 Diseases and Injuries Collaborators. Global incidence, prevalence, years lived with disability (YLDs), disability-adjusted life-years (DALYs), and healthy life expectancy (HALE) for 371 diseases and injuries in 204 countries and territories and 811 subnational locations, 1990-a systematic analysis for the Global Burden of Disease Study 2021. Lancet. 2024 May 18;403(10440):2133-2161. doi: 10.1016/S0140-6736(24)00757-8. Epub 2024 Apr 17. PMID: 38642570; PMCID: PMC11122111.	UNFOCUSED
454	GBD 2021 Risk Factors Collaborators. Global burden and strength of evidence for 88 risk factors in 204 countries and 811 subnational locations, 1990-a systematic analysis for the Global Burden of Disease Study 2021. Lancet. 2024 May 18;403(10440):2162-2203. doi: 10.1016/S0140-6736(24)00933-4. Erratum in: Lancet. 2024 Jul 20;404(10449):244. doi: 10.1016/S0140-6736(24)01458-2. PMID: 38762324; PMCID: PMC11120204.	UNFOCUSED
455	Coon H, Shabalin A, DiBlasi E, Monson ET, Han S, Kaufman EA, Chen D, Kious B, Molina N, Yu Z, Staley M, Crockett DK, Colbert SM, Mullins N, Bakian AV, Docherty AR, Keeshin B. Absence of nonfatal suicidal behavior preceding suicide death reveals differences in clinical risks. medRxiv [Preprint]. 2024 Jun 5:2024.06.05.24308493. doi: 10.1101/2024.06.05.24308493. Update in: Psychiatry Res. 2025 May;347:116391. doi: 10.1016/j.psychres.2025.116391. PMID: 38883733; PMCID: PMC11177925.	UNFOCUSED
456	Gao X, Li Z, Chai J, Li S, Pan X, Liu J, Li L, Qin S, Kang Y, Zhu Y. Electroencephalographic insights into the pathophysiological mechanisms of emergence delirium in children and corresponding clinical treatment strategies. Front Pharmacol. 2024 Jun 19;15:1349105. doi: 10.3389/fphar.2024.1349105. PMID: 38962301; PMCID: PMC11219819.	UNFOCUSED
457	Mitani H, Kondo N, Amemiya A, Tabuchi T. Promotive and protective effects of community-related positive childhood experiences on adult health outcomes in the context of adverse childhood experiences: a nationwide cross-sectional survey in Japan. BMJ Open. 2024 Jun 25;14(6):e082134. doi: 10.1136/bmjopen-2023-082134. PMID: 38925696; PMCID: PMC11202639.	UNFOCUSED

458	Kim S, Perry LM, Mossman B, Dunn A, Hoerger M. Financial burden and physical and emotional quality of life in COPD, heart failure, and kidney failure. PLoS One. 2024 Jul 5;19(7):e0306620. doi: 10.1371/journal.pone.0306620. PMID: 38968278; PMCID: PMC11226075.	UNFOCUSED
459	Nierstedt R, Yershova K, Serafin J, Barnett KM. Patients with Suicidal Ideation for Outpatient Surgery at Freestanding Ambulatory Surgery Centers: How Do We Decide to Proceed or to Postpone? J Clin Anesth. 2024 Aug;95:111424. doi: 10.1016/j.jclinane.2024.111424. Epub 2024 Mar 19. PMID: 38507863.	UNFOCUSED
460	Rodríguez-Prat A, Pergolizzi D, Crespo I, Julià-Torras J, Balaguer A, Kremeike K, Voltz R, Monforte-Royo C. The Wish to Hasten Death in Patients With Life-Limiting Conditions. A Systematic Overview. J Pain Symptom Manage. 2024 Aug;68(2):e91-e115. doi: 10.1016/j.jpainsymman.2024.04.023. Epub 2024 May 3. PMID: 38703862.	UNFOCUSED
461	Tuan NV, Ha LTT, Hung PQ, Thien LC, San BV, Yen NH, Ha TTT, Long NT, Thang TT, Trang DT, Thanh NH. Suicidal Ideation was Associated with Quality of Life Impairment of Patients with Lung Cancer: A Cross-Sectional Study in Vietnam. Asian Pac J Cancer Prev. 2024 Aug 27;25(8):2877-2883. doi: 10.31557/APJCP.2024.25.8.2877. PMID: 39205586; PMCID: PMC11495427.	INCLUDED
462	Zhu S, Jin Q, Zhang S, Song Z, Zhang S, Zhao Z. Integrating Network Pharmacology and Experimental Verification to Explore the Pharmacological Mechanisms of Radix Paeoniae Rubra Against Glioma. Appl Biochem Biotechnol. 2024 Sep;196(9):6424-6441. doi: 10.1007/s12010-024-04887-6. Epub 2024 Feb 21. PMID: 38381309.	UNFOCUSED
463	Tutelman PR, Noel M, Bernier E, Schulte FSM, Kopala-Sibley DC. Adverse Childhood Experiences Moderate the Relationship Between Pain and Later Suicidality Severity Among Youth: A Longitudinal High-Risk Cohort Study. J Pain. 2024 Sep;25(9):104554. doi: 10.1016/j.jpain.2024.104554. Epub 2024 May 6. PMID: 38719156.	UNFOCUSED
464	Mäkitie AA, Alabi RO, Pulkki-Råback L, Almangush A, Beitler JJ, Saba NF, Strojan P, Takes R, Guntinas-Lichius O, Ferlito A. Psychological Factors Related to Treatment Outcomes in Head and Neck Cancer. Adv Ther. 2024 Sep;41(9):3489-3519. doi: 10.1007/s12325-024-02945-3. Epub 2024 Aug 7. PMID: 39110309; PMCID: PMC11349815.	UNFOCUSED
465	Gili R, Gianluca S, Paolo A, Federica S, Paola LC, Simone C, Matteo S, Almalina B, Filippo M, Lucia DM, Vecchio S. The role of prehabilitation in HNSCC patients treated with chemoradiotherapy. Support Care Cancer. 2024 Sep 5;32(10):638. doi: 10.1007/s00520-024-08834-3. PMID: 39235658; PMCID: PMC11377665.	UNFOCUSED
466	Espuig A, Pons-Vinent M, Carbajo E, Lacomba-Trejo L. The Impact of Pain, Threat Perception and Emotional Distress on Suicide Risk in Individuals with Colorectal Cancer. Nurs Rep. 2024 Sep 25;14(4):2629-2639. doi: 10.3390/nursrep14040194. PMID: 39449431; PMCID: PMC11503388.	INCLUDED
467	Khosravi M, Kasaeiyan R. A current challenge in classification and treatment of DSM-5-TR prolonged grief disorder. Psychol Trauma. 2024 Nov;16(8):1239-1241. doi: 10.1037/tra0001510. Epub 2023 Apr 27. PMID: 37104775.	UNFOCUSED
468	Nwankwo A, Koyyalagunta D, Huh B, D'Souza RS, Javed S. A comprehensive review of the typical and atypical side effects of gabapentin. Pain Pract. 2024 Nov;24(8):1051-1058. doi: 10.1111/papr.13400. Epub 2024 Jul 1. PMID: 38949515.	UNFOCUSED
469	Raja SM, Guptill JT, Mack M, Peterson M, Byard S, Twieg R, Jordan L, Rich N, Castledine R, Bourne S, Wilmshurst M, Oxendine S, Avula SGC, Zuleta H, Quigley P, Lawson S, McQuaker SJ, Ahmadkhaniha R, Appelbaum LG, Kowalski K, Barksdale CT, Gufford BT, Awan A, Sancho AR, Moore MC, Berrada K, Cogan GB, DeLaRosa J, Radcliffe J, Pao M, Kennedy M, Lawrence Q, Goldfeder L, Amanfo L, Zanos P, Gilbert JR, Morris PJ, Moaddel R, Gould TD, Zarate CA Jr, Thomas CJ. A Phase 1 Assessment of the Safety, Tolerability, Pharmacokinetics and Pharmacodynamics of (2R,6R)-Hydroxynorketamine in Healthy Volunteers. Clin Pharmacol Ther. 2024 Nov;116(5):1314-1324. doi: 10.1002/cpt.3391. Epub 2024 Jul 25. PMID: 39054770; PMCID: PMC11479831.	UNFOCUSED

470	Park SY. Sex-Specific Factors Associated with Suicidal Ideation among Patients with Cancer in Korea: A Population-Based Study. Asian Pac J Cancer Prev. 2024 Nov 1;25(11):3987-3996. doi: 10.31557/APJCP.2024.25.11.3987. PMID: 39611923; PMCID: PMC11996106.	UNFOCUSED
471	Dasanu CA, Alvarez-Argote J, Dasanu RG, Soliman A, Codreanu I. Erlotinib- induced Perioral Lesions Resembling Scleroderma. Acta Dermatovenerol Croat. 2024 Nov;32(2):118-119. PMID: 39803739.	UNFOCUSED
472	GBD 2019 Injuries Collaborators. Global, regional, and national burden of injuries, and burden attributable to injuries risk factors, 1990 to 2019: results from the Global Burden of Disease study 2019. Public Health. 2024 Dec;237:212-231. doi: 10.1016/j.puhe.2024.06.011. Epub 2024 Oct 24. PMID: 39454232.	UNFOCUSED
473	DiBlasi E, Kaufman EA, Webster S, Hagn EE, Shabalin AA, Chen D, Han S, Jawish R, Monson ET, Staley MJ, Keeshin BR, Docherty AR, Bakian AV, Okifuji A, Coon H. Phenome-wide diagnostic comparison among suicide deaths and living individuals with chronic pain diagnoses. BMC Med. 2024 Dec 2;22(1):568. doi: 10.1186/s12916-024-03794-1. PMID: 39617899; PMCID: PMC11610288.	UNFOCUSED
474	GBD 2021 US Burden of Disease Collaborators. The burden of diseases, injuries, and risk factors by state in the USA, 1990-a systematic analysis for the Global Burden of Disease Study 2021. Lancet. 2024 Dec 7;404(10469):2314-2340. doi: 10.1016/S0140-6736(24)01446-6. Erratum in: Lancet. 2025 Jan 25;405(10475):302. doi: 10.1016/S0140-6736(25)00105-9. PMID: 39645376; PMCID: PMC11694014.	UNFOCUSED
475	GBD 2021 US Burden of Disease and Forecasting Collaborators. Burden of disease scenarios by state in the USA, 2022-a forecasting analysis for the Global Burden of Disease Study 2021. Lancet. 2024 Dec 7;404(10469):2341-2370. doi: 10.1016/S0140-6736(24)02246-3. Erratum in: Lancet. 2025 May 3;405(10489):1579. doi: 10.1016/S0140-6736(25)00828-1. PMID: 39645377; PMCID: PMC11715278.	UNFOCUSED
476	Harsanyi H, Yang L, Lau J, Cheung W, Cuthbert C. The contribution of nonmedical opioid use to healthcare encounters for opioid overdose and use disorders among long-term users with metastatic cancer. Support Care Cancer. 2024 Dec 13;33(1):27. doi: 10.1007/s00520-024-09082-1. Erratum in: Support Care Cancer. 2025 Jan 20;33(2):114. doi: 10.1007/s00520-025-09161-x. PMID: 39672945.	UNFOCUSED
477	Dydyk AM, Sizemore DC, Trachsel LA, Conermann T, Porter BR. West Virginia Opioid Prescribing for Chronic Pain While Avoiding Drug Diversion. 2023 Jun 12. In: StatPearls [Internet]. Treasure Island (FL): StatPearls Publishing; 2025 Jan–. PMID: 33090753.	UNFOCUSED
478	Dydyk AM, Sizemore DC, Trachsel LA, Conermann T, Porter BR. Vermont Controlled Substance Abuse, Diversion, Storage, Disposal, Monitoring, and Legal Issues. 2022 Nov 26. In: StatPearls [Internet]. Treasure Island (FL): StatPearls Publishing; 2025 Jan–. PMID: 33232093.	UNFOCUSED
479	Dydyk AM, Sizemore DC, Ravert DM, Porter BR. Understanding Delaware Prescribing and Distribution of Controlled Substances. 2023 Apr 10. In: StatPearls [Internet]. Treasure Island (FL): StatPearls Publishing; 2025 Jan–. PMID: 33232097.	UNFOCUSED
480	Dydyk AM, Sizemore DC, Haddad LM, Lindsay L, Porter BR. NP Safe Prescribing of Controlled Substances While Avoiding Drug Diversion. 2023 Jan 29. In: StatPearls [Internet]. Treasure Island (FL): StatPearls Publishing; 2025 Jan–. PMID: 33232099.	UNFOCUSED
481	Dydyk AM, Sizemore DC, Fariba KA, Sanghavi DK, Porter BR. Florida Controlled Substance Prescribing. 2022 Oct 26. In: StatPearls [Internet]. Treasure Island (FL): StatPearls Publishing; 2025 Jan–. PMID: 33428370.	UNFOCUSED
482	Dydyk AM, Sizemore DC, Smock W, Dulebohn SC, Porter BR. Kentucky KASPER and Controlled Substance Prescribing. 2023 Jun 12. In: StatPearls [Internet]. Treasure Island (FL): StatPearls Publishing; 2025 Jan–. PMID: 33620803.	UNFOCUSED
483	Dydyk AM, Sizemore DC, Trachsel LA, Dulebohn SC, Porter BR. Tennessee Controlled Substance Prescribing for Acute and Chronic Pain. 2023 May 27. In: StatPearls [Internet]. Treasure Island (FL): StatPearls Publishing; 2025 Jan–. PMID: 33620833.	UNFOCUSED
484	Dydyk AM, Sizemore DC, Patel BC, Ronquillo Y, Porter BR. Utah Controlled Substance Prescribing. 2023 Apr 10. In: StatPearls [Internet]. Treasure Island (FL): StatPearls Publishing; 2025 Jan–. PMID: 33620855.	UNFOCUSED

485	Lebwohl MG, Koo JY, Armstrong AW, Strober BE, Yoon SH, Rawnsley NN, Goehring EL Jr, Mangin GD, Jacobson AA. Brodalumab: Six-Year US Pharmacovigilance Report. Dermatol Ther (Heidelb). 2025 Jan;15(1):213-222. doi: 10.1007/s13555-024-01304-y. Epub 2024 Nov 26. PMID: 39589679; PMCID: PMC11785849.	UNFOCUSED
486	Lee JH, Lee H, Son Y, Kim HJ, Park J, Lee H, Fond G, Boyer L, Smith L, Rahmati M, Pizzol D, Kang J, Yon DK, Oh H. Racial Discrimination and Multiple Health Outcomes: An Umbrella Review of Systematic Reviews and Meta-Analyses. Med Princ Pract. 2025;34(2):138-151. doi: 10.1159/000542988. Epub 2024 Dec 5. PMID: 39637838; PMCID: PMC11936452.	UNFOCUSED
487	Simon NB, Mas D Alessandro NM, Lebak K, Serafin J, Barnett KM. Special Populations in Ambulatory Surgery: Oncologic, Lactating, Transgender and Gender Diverse, and Suicidal Ideation. Int Anesthesiol Clin. 2025 Jan 1;63(1):32-44. doi: 10.1097/AIA.0000000000000459. Epub 2024 Nov 15. PMID: 39651666; PMCID: PMC12510472.	UNFOCUSED
488	Mwobobia J, White MC, Osazuwa-Peters OL, Adjei Boakye E, Abouelella DK, Barnes JM, Viet CT, Ramos K, Corbett C, Osazuwa-Peters N. Depression, non- medical pain prescriptions, and suicidal behavior in cancer survivors. J Cancer Surviv. 2025 Jan 16:10.1007/s11764-024-01740-x. doi: 10.1007/s11764-024-01740-x. Epub ahead of print. PMID: 39821751; PMCID: PMC12217687.	UNFOCUSED
489	Brewer C, Hopwood MC, Winyard G. Assisted deaths in Switzerland for UK residents: diagnoses and their implications for palliative medicine and assisted dying legislation. BMJ Support Palliat Care. 2025 Feb 26;15(2):259-261. doi: 10.1136/spcare-2023-004719. PMID: 38395598.	UNFOCUSED
490	Huang YT, Jenkins DA, Yimer BB, Jani M. Factors associated with long-term opioid use among patients with axial spondyloarthritis or psoriatic arthritis who initiate opioids. Rheumatology (Oxford). 2025 Apr 1;64(4):1844-1852. doi: 10.1093/rheumatology/keae444. PMID: 39150473; PMCID: PMC11962957.	UNFOCUSED
491	Eskreis-Winkler L, Troncoso Peres LT, Fishbach A. The bigger the problem the littler: When the scope of a problem makes it seem less dangerous. J Pers Soc Psychol. 2025 Apr;128(4):790-806. doi: 10.1037/pspa0000409. Epub 2024 Oct 24. PMID: 39446641.	UNFOCUSED
492	Blaney C, Sommer JL, Bilevicius E, Mota N, El-Gabalawy R. System-based pain groups are uniquely associated with sociodemographic and psychiatric correlates among those with posttraumatic stress disorder (PTSD). J Psychiatr Res. 2025 Apr;184:241-248. doi: 10.1016/j.jpsychires.2025.02.058. Epub 2025 Feb 28. PMID: 40056644.	UNFOCUSED
493	Goldstein KM, Pace R, Dancu C, Raman SR, Bridges-Curry Z, Klimek-Johnson P, Jeevanathan A, Gallion AH, Der T, Tabriz AA, Sprague S, Rushton S, Hammer AJ, Sims CA, Coleman JN, Martino J, Cantrell S, Gordon AM, Jacobs M, Alexopoulos AS, Chen D, Gierisch JM. An Evidence Map of the Women Veterans' Health Literature, 2016 to A Systematic Review. JAMA Netw Open. 2025 Apr 1;8(4):e256372. doi: 10.1001/jamanetworkopen.2025.6372. Erratum in: JAMA Netw Open. 2025 May 1;8(5):e2518943. doi: 10.1001/jamanetworkopen.2025.18943. PMID: 40261651; PMCID: PMC12015682.	UNFOCUSED
494	Zhang L, Lin X, Hang L. Non-linear relationship between serum α-Klotho and suicide attempt: evidence from a large never-smoking population. BMC Psychiatry. 2025 Apr 16;25(1):382. doi: 10.1186/s12888-025-06784-6. PMID: 40241033; PMCID: PMC12004823.	UNFOCUSED
495	Lazar DE, Hanganu B, Postolica R, Buhas CL, Paparau C, Ioan BG. Suicide Risk in Digestive Cancer Patients: A Systematic Review of Sociodemographic, Psychological, and Clinical Predictors. Cancers (Basel). 2025 Apr 24;17(9):1427. doi: 10.3390/cancers17091427. PMID: 40361354; PMCID: PMC12070826.	REVIEW
496	Streitberger K, Harnik MA, Saliba A, Bischoff N, Blättler LT, Schwegler K, Baumgartner C, Sutter N, Wertli MM. Two-Phase Inpatient Withdrawal Programme for Long-Term Opioid Use in Non-Cancer Pain. Eur J Pain. 2025 May;29(5):e70010. doi: 10.1002/ejp.70010. PMID: 40116133; PMCID: PMC11926771.	UNFOCUSED
497	Kurisu K, Fujimori M, Harashima S, Okamura M, Yoshiuchi K, Uchitomi Y. Exploratory Analysis of Nationwide Japanese Patient Safety Reports on Suicide and Suicide Attempts Among Inpatients With Cancer Using Large Language Models. Psychooncology. 2025 May;34(5):e70150. doi: 10.1002/pon.70150. PMID: 40320591; PMCID: PMC12050354.	UNFOCUSED

498	Killikelly C, Smith KV, Zhou N, Prigerson HG, O'Connor MF, Kokou-Kpolou CK, Boelen PA, Maercker A. Prolonged grief disorder. Lancet. 2025 May 3;405(10489):1621-1632. doi: 10.1016/S0140-6736(25)00354-X. Epub 2025 Apr 17. PMID: 40254022.	UNFOCUSED
499	Rolová G, Engeland A, Pedersen L, Odsbu I, Hamina A, Skurtveit S. Mortality among patients with long-term prescription opioid use in Norway: a nationwide registry-based cohort study. Pain. 2025 May 13. doi: 10.1097/j.pain.0000000000003653. Epub ahead of print. PMID: 40372278.	UNFOCUSED
500	Osaghae I, Chido-Amajuoyi OG, Talluri R, Shete S. Prevalence, reasons for use, perceived benefits, and awareness of health risks of cannabis use among cancer survivors - implications for policy and interventions. J Cancer Surviv. 2025 Jun;19(3):1010-1018. doi: 10.1007/s11764-023-01526-7. Epub 2023 Dec 29. PMID: 38158514; PMCID: PMC11817783.	UNFOCUSED
501	Mercadante S. Assisted Suicide in Italy: There Is Great Chaos Under Heaven. J Palliat Med. 2025 Jun 16. doi: 10.1089/jpm.2025.0128. Epub ahead of print. PMID: 40522641.	UNFOCUSED
502	Tang C, Liu Y, Duan L, Wang T, Hu J, Jin Y, Zhang K. Linking psychological pain and suicidal ideation: The chain mediating effects of time perspective and meaning in life in young adults with cancer. J Health Psychol. 2025 Jun 19:13591053251344503. doi: 10.1177/13591053251344503. Epub ahead of print. PMID: 40534340.	UNFOCUSED
503	Stretanski MF, Kopitnik NL, Matha A, Conermann T. Chronic Pain. 2025 Jun 23. In: StatPearls [Internet]. Treasure Island (FL): StatPearls Publishing; 2025 Jan–. PMID: 31971706.	UNFOCUSED
504	Hanna JR, McCloy K, Anderson J, McKeever A, Semple CJ. Suicide and Head and Neck Cancer: A Systematic Review With Meta-Analysis and Narrative Synthesis. Psychooncology. 2025 Jul;34(7):e70233. doi: 10.1002/pon.70233. PMID: 40709719; PMCID: PMC12291453.	REVIEW
505	Barbosa ERF, da Veiga DR, Ayala MG, Filho IDAOS, Gomez AS, de Alencar EBA, Pérez AS, Okereke CE, Vinas DC, Siquieroli RS, de Sousa Francelino H, Posso AN, Meneghetti Inácio Silva I, da Cunha PHM, Labra VB, Gomez-Carrillo D, Lanaia S, de Almeida LKR, Lomashvili EM, Sanchez LSS, Muñoz SC, Fuentes P, Mello AGT, Emiliozzi N, Terseg A, Peralta RAR, Nishizawa T, Chavez EBV, Bueno C. Sleep Impairment and Chronic Pain in the Military: A Scoping Review. J Sleep Res. 2025 Jul 14:e70100. doi: 10.1111/jsr.70100. Epub ahead of print. PMID: 40653977.	UNFOCUSED
506	Khanani MI, Khan MR, Farooqi MF, Fazal J, Aabideen Z, Alkuwaiti NS. Digital Media Use and Screen Time Exposure Among Youths: A Lifestyle-Based Public Health Concern. Cureus. 2025 Jul 20;17(7):e88373. doi: 10.7759/cureus.88373. PMID: 40837898; PMCID: PMC12364383.	UNFOCUSED
507	Zhou J, Zhou F, Tang Y, Ma J. Navigating mortality: exploring the dynamic changes related to cultural worldviews and self-esteem in cancer patients. BMC Psychol. 2025 Jul 22;13(1):817. doi: 10.1186/s40359-025-03037-9. PMID: 40696463; PMCID: PMC12285192.	UNFOCUSED
508	Wright B, Evanson A, Casey C, Law KC, Rogers AH, Comtois KA. Exploring the Impact of the Caring Contacts Intervention on the Stress and Distress of Veterans and Service Members: Protocol for a Randomized Controlled Trial. JMIR Res Protoc. 2025 Aug 13;14:e72140. doi: 10.2196/72140. PMID: 40801426; PMCID: PMC12391844.	UNFOCUSED
509	Lee Y, Giordano NA, Compton PA, Tang NKY, Polomano RC, Cheatle MD. Correlates of mental defeat in individuals with chronic non-cancer pain on long- term opioid therapy. Pain Med. 2025 Sep 1;26(9):576-582. doi: 10.1093/pm/pnaf055. PMID: 40341399; PMCID: PMC12405750.	UNFOCUSED
510	Shim EJ, Noh HL, Hahm BJ, Lee YJ, Lee JW, Youn HJ, Park SJ. Validation of the MASC-20 and Development of a Short Form to Assess Suicide Risk in Cancer Patients. J Pain Symptom Manage. 2025 Sep;70(3):302-312.e3. doi: 10.1016/j.jpainsymman.2025.06.001. Epub 2025 Jun 6. PMID: 40484358.	UNFOCUSED
511	Abdi N, Mohebbi Z, Ghanbarzadeh S, Keshtkaran Z, Zaj P. Spiritual Distress in Women With Breast Cancer: A Concept Analysis. Scand J Caring Sci. 2025 Sep;39(3):e70046. doi: 10.1111/scs.70046. PMID: 40589282.	UNFOCUSED

512	Buchok M, Chochinov HM, Kowall S, Bolton SL, El-Gabalawy R, Hensel JM, Bolton JM. Self-Reported Dignity among People Admitted to Psychiatric Wards and Its Association with Suicidal Behaviour: Perte de dignité auto-évaluée chez les personnes admises dans des services psychiatriques et son association avec les comportements suicidaires. Can J Psychiatry. 2025 Sep;70(9):681-689. doi: 10.1177/07067437251355644. Epub 2025 Jul 7. PMID: 40619944; PMCID: PMC12234506.	UNFOCUSED
513	Loftus EV Jr, D'Haens G, Louis E, Regueiro M, Jairath V, Magro F, Nakase H, Dubcenco E, Lacerda AP, Ford S, Feng T, Duncan B, Fish I, Cunneen C, Anyanwu SI, Aponte F, Griffith J, Blumenstein I. Efficacy and safety of upadacitinib maintenance therapy in patients with moderately to severely active Crohn's disease: 2-year results from the U-ENDURE Long-Term Extension study. J Crohns Colitis. 2025 Sep 7;19(8):jjaf138. doi: 10.1093/ecco-jcc/jjaf138. PMID: 40704669; PMCID: PMC12459986.	UNFOCUSED
514	Huang S, Jiao T, Guo SJ, Star JA, Bian J, Wilson DL, Goodin AJ. Suicide risk after abrupt discontinuation of long-term opioid therapy: an observational cohort study. Pain Med. 2025 Oct 1;26(10):631-642. doi: 10.1093/pm/pnaf044. PMID: 40209076.	UNFOCUSED
515	Weeger-Elsner S, Elsner F. Wahrung der Patientenautonomie am Lebensende: Sterbebegleitung zwischen Lebensschutz und Recht auf Selbstbestimmung [Consideration of patient autonomy at the end of life : End of life care between the protection of life and the right to self-determination]. Schmerz. 2025 Oct;39(5):369-377. German. doi: 10.1007/s00482-025-00894-6. Epub 2025 Jul 14. PMID: 40658150.	UNFOCUSED
516	Weinstock LB, Afrin LB, Reiersen AM, Brook J, Blitshteyn S, Ehrlich G, Schofield JR, Kinsella L, Kaufman D, Dempsey T, Molderings GJ. Prevalence and treatment response of neuropsychiatric disorders in mast cell activation syndrome. Brain Behav Immun Health. 2025 Jun 30;48:101048. doi: 10.1016/j.bbih.2025.101048. PMID: 40686928; PMCID: PMC12270938.	UNFOCUSED
517	Lebwohl MG, Koo JY, Armstrong AW, Strober BE, Yoon SH, Rawnsley NN, Goehring EL Jr, Jacobson AA. Brodalumab: Seven-Year US Pharmacovigilance Report. Dermatol Ther (Heidelb). 2025 Oct;15(10):3025-3035. doi: 10.1007/s13555-025-01497-w. Epub 2025 Jul 29. PMID: 40730908; PMCID: PMC12454691.	UNFOCUSED
518	Ruggiero R, Longo M, Mascolo A, Di Nuzzo M, Laino LV, Caruso P, D'Amato R, Rafaniello C, Maiorino MI, Esposito K, Capuano A. Real-world safety comparison of liraglutide and semaglutide in weight management: Insights from European pharmacovigilance data. Eur J Pharmacol. 2025 Oct 5;1004:178004. doi: 10.1016/j.ejphar.2025.178004. Epub 2025 Jul 25. PMID: 40716637.	UNFOCUSED

Table S2. Results from the PsycInfo database search with final decision label. (pain* AND [suicid* OR "self-harm" OR "self-injurious behavior" OR "self-inflicted injury" or "self-killing"] AND [cancer* OR oncolog* OR tumor* OR neoplasm* OR metasta*]. → 314 results (Oct 1st, 2025) → 0 included.

	**References**	**Label**
1	Zhou J, Zhou F, Tang Y, Ma J. Navigating mortality: Exploring the dynamic changes related to cultural worldviews and self-esteem in cancer patients. BMC Psychology. 2025;13(1):817. doi:https://doi.org/10.1186/s40359-025-03037-9	DUPLICATED
2	1. Barbosa ERF, da Veiga DR, Ayala MG, et al. Sleep impairment and chronic pain in the military: A scoping review. J Sleep Res. 2025:15. doi:https://doi.org/10.1111/jsr.70100	DUPLICATED
3	1. Eskreis-Winkler L, Troncoso Peres LT, Fishbach A. The bigger the problem the littler: When the scope of a problem makes it seem less dangerous. J Pers Soc Psychol. 2025;128(4):790. doi:https://doi.org/10.1037/pspa0000409	DUPLICATED
4	1. APA handbook of pediatric psychology, developmental-behavioral pediatrics, and developmental science: Pediatric psychology and developmental-behavioral pediatrics: Clinical applications of developmental science. American Psychological Association; 2025	LUMPING
5	1. Santo TJ, Jr. Examining childhood trauma and comorbid mental disorders among people with opioid use disorder. Order No. AAI31863970 ed. University of New South Wales (Australia); 2025.	REVIEW

	https://www.proquest.com/dissertations-theses/examining-childhood-trauma-comorbid-mental/docview/3188430731/se-2	
6	1. Khosravi M, Kasaeiyan R. A current challenge in classification and treatment of DSM-5-TR prolonged grief disorder. Psychological Trauma: Theory, Research, Practice, and Policy. 2024;16(8):1239-1241. doi:https://doi.org/10.1037/tra0001510	DUPLICATED
7	Nwankwo A, Koyyalagunta D, Huh B, D'Souza RS, Javed S. A comprehensive review of the typical and atypical side effects of gabapentin. Pain Practice. 2024;24(8):1051-1058. doi:https://doi.org/10.1111/papr.13400	DUPLICATED
8	Cheatle MD. Pain, substance use disorder and suicide: On the edge. Current Addiction Reports. 2024;11(5):809-817. doi:https://doi.org/10.1007/s40429-024-00585-9	REVIEW
9	Interventions in health care interaction. Palgrave Macmillan/Springer Nature; 2024	UNFOCUSED
10	Bennett S, Robb KA, Andoh-Arthur J, et al. Establishing research priorities for investigating male suicide risk and recovery: A modified Delphi study with lived-experience experts. Psychology of Men & Masculinities. 2024;25(1):85-98. doi:https://doi.org/10.1037/men0000448	REVIEW
11	Liaw V, McCreary M, Friedman DI. Quality of life in patients with confirmed and suspected spinal CSF leaks. Neurology. 2023;101(23). doi:https://doi.org/10.1212/WNL.0000000000207763	DUPLICATED
12	Henry M, Alias A, Bisson-Gervais V, et al. Medical assistance in dying in Canada: A scoping review on the concept of suffering. Psychooncology. 2023;32(9):1339-1347. doi:https://doi.org/10.1002/pon.6196	DUPLICATED
13	Way BM, Griffin KR, Kraus SW, Tsai J, Pietrzak RH. Erectile dysfunction in a U.S. national sample of male military veterans. Mil Med. 2023;188(9-10):2837-2843. doi:https://doi.org/10.1093/milmed/usac187	DUPLICATED
14	Lewis BR, Garland EL, Byrne K, et al. HOPE: A pilot study of psilocybin enhanced group psychotherapy in patients with cancer. J Pain Symptom Manage. 2023;66(3):258-269. doi:https://doi.org/10.1016/j.jpainsymman.2023.06.006	UNFOCUSED
15	Godena EJ, Freeburn JL, Silverberg ND, Perez DL. A case of functional cognitive disorder: Psychotherapy and speech and language therapy insights. Harv Rev Psychiatry. 2023;31(5):248. doi:https://doi.org/10.1097/HRP.0000000000000379	CASE
16	Cheatle MD, Giordano NA, Themelis K, Tang NKY. Suicidal thoughts and behaviors in patients with chronic pain, with and without co-occurring opioid use disorder. Pain Medicine. 2023;24(8):941-948. doi:https://doi.org/10.1093/pm/pnad043	DUPLICATED
17	Bennett S, Robb KA, Zortea TC, Dickson A, Richardson C, O'Connor RC. Male suicide risk and recovery factors: A systematic review and qualitative metasynthesis of two decades of research. Psychol Bull. 2023;149(7-8):371. doi:https://doi.org/10.1037/bul0000397	REVIEW
18	Zhou JX, Goh C, Chiam M, Krishna LKR. Painting and poetry from a bereaved family and the caring physician. J Pain Symptom Manage. 2023;65(5). doi:https://doi.org/10.1016/j.jpainsymman.2022.03.008	DUPLICATED
19	James KE, Agarwal S, Armenion KL, et al. A deductive thematic analysis of nurses with job-related problems who completed suicide during the early COVID-19 pandemic: A preliminary report. Worldviews on Evidence-Based Nursing. 2023;20(2):96-106. doi:https://doi.org/10.1111/wvn.12640	DUPLICATED
20	Du L, Shi H, Qian Y, et al. Development and validation of a model for predicting the risk of suicide in patients with cancer. Arch Suicide Res. 2023;27(2):644-659. doi:https://doi.org/10.1080/13811118.2022.2035289	UNFOCUSED
21	Lester EG, Wang KE, Blakeley JO, Vranceanu A. Occurrence and severity of suicidal ideation in adults with neurofibromatosis participating in a mind–body RCT. Cognitive and Behavioral Neurology. 2023;36(1):19-27. doi:https://doi.org/10.1097/WNN.0000000000000332	DUPLICATED
22	Campos AI, Garcia-Marin L, Christensen H, et al. Genomics-driven screening for causal determinants of suicide attempt. Aust N Z J Psychiatry. 2023;57(3):423-431. doi:https://doi.org/10.1177/00048674221091499	DUPLICATED
23	Shnayder S, Ameli R, Sinaii N, Berger A, Agrawal M. Psilocybin-assisted therapy improves psycho-social-spiritual well-being in cancer patients. J Affect Disord. 2023;323:592-597. doi:https://doi.org/10.1016/j.jad.2022.11.046	UNFOCUSED
24	Murri MB, Caruso R, Christensen AP, Folesani F, Nanni MG, Grassi L. The facets of psychopathology in patients with cancer: Cross-sectional and longitudinal network analyses. J Psychosom Res. 2023;165:111139. doi:https://doi.org/10.1016/j.jpsychores.2022.111139	DUPLICATED
25	Quah ELY, Chua KZY, Lua JK, et al. A systematic review of stakeholder perspectives of dignity and assisted dying. J Pain Symptom Manage. 2023;65(2):e123-e136. doi:https://doi.org/10.1016/j.jpainsymman.2022.10.004	DUPLICATED
26	Åkeflo L, Elmerstig E, Bergmark K, Dunberger G. Barriers to and strategies for dealing with vaginal dilator therapy—Female pelvic cancer survivors' experiences: A qualitative study. European Journal of Oncology Nursing. 2023;62:1-8. doi:https://doi.org/10.1016/j.ejon.2022.102252	DUPLICATED
27	Rowland JH. When the loss is not just personal, but is of one's self. In: Loscalzo M, Forstein M, Klein LA, eds. Loss and grief: Personal stories of doctors and other healthcare professionalsOxford University Press; 2023:61. https://www.proquest.com/books/when-loss-is-not-just-personal-one-s-self/docview/2766115387/se-2	UNFOCUSED

28	Handbook of clinical child psychology: Integrating theory and research into practice. Springer Nature Switzerland AG; 2023	LUMPING
29	Handbook of psychiatry in palliative medicine: Psychosocial care of the terminally ill. 3rd ed. ed. Oxford University Press; 2023	LUMPING
30	Onyeka TC, Onu JU, Agom DA. Psychosocial aspects of adult cancer patients: A scoping review of sub-Saharan Africa. Psychooncology. 2023;32(1):86. doi:https://doi.org/10.1002/pon.6052	DUPLICATED
31	Lozupone M, Donghia R, Sardone R, et al. Apolipoprotein E genotype, inflammatory biomarkers, and non-psychiatric multimorbidity contribute to the suicidal ideation phenotype in older age. The Salus in Apulia Study. J Affect Disord. 2022;319:202-212. doi:https://doi.org/10.1016/j.jad.2022.09.046	DUPLICATED
32	Kazlauskiene J, Navickas A, Lesinskiene S, Bulotiene G. Risk factors for suicide in cancer patients and preventive measures: A literature review. Subtitle: Cancer patient's suicides and prevention. Archives of Psychiatry and Psychotherapy. 2022;24(4):68-77. doi:https://doi.org/10.12740/APP/152776	REVIEW
33	Raghubar KP, Chambers T, Hill RM, et al. Longitudinal investigation of suicidal ideation and associated factors during pediatric acute lymphoblastic leukemia chemotherapy. Psychooncology. 2022;31(10):1782. doi:https://doi.org/10.1002/pon.6014	DUPLICATED
34	Bahnsen MK, Graugaard C, Andersson M, Andresen JB, Frisch M. Physical and mental health problems and their associations with inter-personal sexual inactivity and sexual dysfunctions in Denmark: Baseline assessment in a national cohort study. Journal of Sexual Medicine. 2022;19(10):1562-1579. doi:https://doi.org/10.1016/j.jsxm.2022.07.004	DUPLICATED
35	Onwumere J, Stubbs B, Stirling M, et al. Pain management in people with severe mental illness: An agenda for progress. Pain. 2022;163(9):1653-1660. doi:https://doi.org/10.1097/j.pain.0000000000002633	UNFOCUSED
36	Xing Y, Zhao W, Duan C, et al. Developing a visual model for predicting depression in patients with lung cancer. J Clin Nurs. 2022. doi:https://doi.org/10.1111/jocn.16487	DUPLICATED
37	Molla A, Aderaw M, Mulat H, Fanta B, Nenko G, Adane A. Suicidal ideation, attempt and associated factors among people living with cancer in Ethiopia: A cross-sectional study. Annals of General Psychiatry. 2022;21:28. doi:https://doi.org/10.1186/s12991-022-00407-0	DUPLICATED
38	Padron A, McCrae CS, Robinson ME, et al. Impacts of cognitive behavioral therapy for insomnia and pain on sleep in women with gynecologic malignancies: A randomized controlled trial. Behavioral Sleep Medicine. 2022;20(4):460-476. doi:https://doi.org/10.1080/15402002.2021.1932500	UNFOCUSED
39	Zhang L, Cai H, Bai W, et al. Prevalence of suicidality in clinically stable patients with major depressive disorder during the COVID-19 pandemic. J Affect Disord. 2022;307:142-148. doi:https://doi.org/10.1016/j.jad.2022.03.042	DUPLICATED
40	Coon H, Shabalin A, Bakian AV, et al. Extended familial risk of suicide death is associated with younger age at death and elevated polygenic risk of suicide. American Journal of Medical Genetics Part B: Neuropsychiatric Genetics. 2022;189(3-4):60. doi:https://doi.org/10.1002/ajmg.b.32890	DUPLICATED
41	Wahab S, Chua TY, Razali R, Saher ZM, Zamzam IH, Bujang MA. Suicidal behavior among elderly inpatients: Its relation to functional disability and pain. Psychology Research and Behavior Management. 2022;15:28. doi:https://doi.org/10.2147/PRBM.S341768	DUPLICATED
42	Chen H, He Q, Zeng Y, et al. Feeling like the sky is falling down: Experiences of parents of adolescents diagnosed with cancer in one-child families in China—A qualitative study. J Clin Nurs. 2022;31(5-6):733. doi:https://doi.org/10.1111/jocn.15913	DUPLICATED
43	Chen W, Chen Y, Xiao H. Existential distress in cancer patients: A concept analysis. Cancer Nurs. 2022;45(2):E471-E486. doi:https://doi.org/10.1097/NCC.0000000000000925	DUPLICATED
44	Sullivan MD, Ballantyne JC. The right to pain relief: Its origins in end-of-life care and extension to chronic pain care. Clin J Pain. 2022;38(1):58-63. doi:https://doi.org/10.1097/AJP.0000000000001000	DUPLICATED
45	Sylvia JM. Self-administered psychotherapy for chronic non-cancer pain and depression. Order No. AAI28491779 ed. Salve Regina University; 2022. https://www.proquest.com/dissertations-theses/self-administered-psychotherapy-chronic-non/docview/2596639996/se-2	UNFOCUSED
46	Julião M, Chochinov HM, Samorinha C, da Silva Soares D, Antunes B. Prevalence and factors associated with will-to-live in patients with advanced disease: Results from a Portuguese retrospective study. J Pain Symptom Manage. 2021;62(4):820-827. doi:https://doi.org/10.1016/j.jpainsymman.2021.02.018	DUPLICATED
47	Kułak-Bejda A, Bejda G, Waszkiewicz N. Mental disorders, cognitive impairment and the risk of suicide in older adults. Frontiers in Psychiatry. 2021;12:7. doi:https://doi.org/10.3389/fpsyt.2021.695286	DUPLICATED
48	Aziato L, Pwavra JBP, Paarima Y, Konlan KD. The nurse or midwife at the crossroads of caring for patients with suicidal and rigid religious ideations in Africa. Frontiers in Psychology. 2021;12:7. doi:https://doi.org/10.3389/fpsyg.2021.549766	DUPLICATED

49	Shahar G. Interdisciplinarity and integration: An introduction to the special issue on psychopathology in medical settings. Journal of Clinical Psychology in Medical Settings. 2021;28(1):1. doi:https://doi.org/10.1007/s10880-020-09752-2	DUPLICATED
50	Forman EM, Arch JJ, Bricker JB, et al. Mindfulness and acceptance-based treatments. In: Barkham M, Lutz W, Castonguay LG, eds. Bergin and Garfield's handbook of psychotherapy and behavior change: 50th anniversary edition (7th ed.)7th ed. ed. John Wiley & Sons, Inc; 2021:507. https://www.proquest.com/books/mindfulness-acceptance-based-treatments/docview/2687769293/se-2	REVIEW
51	Stenager E, Stenager E, Erlangsen A. Somatic diseases and suicidal behaviour. In: Wasserman D, ed. Oxford textbook of suicidology and suicide prevention (2nd ed.)2nd ed. ed. Oxford University Press; 2021:321. https://www.proquest.com/books/somatic-diseases-suicidal-behaviour/docview/2661136326/se-2	REVIEW
52	Johnson J. Mindful writing for transformation. In: Monk L, Maisel E, eds. Transformational journaling for coaches, therapists, and clients: A complete guide to the benefits of personal writingRoutledge/Taylor & Francis Group; 2021:139	REVIEW
53	Khechine W, Ezzaairi F, Sahli J, et al. Burn-out of the medical oncology health care professionals and associated factors in Tunisia. Psycho-Oncologie. 2020;14(4):207-216. doi:https://doi.org/10.3166/pson-2020-0134	UNFOCUSED
54	Xu Q, Jia S, Fukasawa M, et al. A cross-sectional study on associations of physical symptoms, health self-efficacy, and suicidal ideation among Chinese hospitalized cancer patients. BMC Psychiatry. 2020;20:10. doi:https://doi.org/10.1186/s12888-020-02945-x	DUPLICATED
55	Zhang Y, Li W, Zhang Z, et al. Suicidal ideation in newly-diagnosed Chinese cancer patients. Frontiers in Psychiatry. 2020;11:8. doi:https://doi.org/10.3389/fpsyt.2020.00708	DUPLICATED
56	Isenberg-Grzeda E, Bean S, Cohen C, Selby D. Suicide Attempt After Determination of Ineligibility for Assisted Death: A Case Series. J Pain Symptom Manage. 2020 Jul;60(1):158-163. doi: 10.1016/j.jpainsymman.2020.02.016. Epub 2020 Feb 24. PMID: 32105792.	DUPLICATED
57	Hassamal S, Razavi M, Clark K, Dale W, Loscalzo M. Pain distress among patients prior to initiating cancer treatment. Psychooncology. 2020;29(5):938-941. doi:https://doi.org/10.1002/pon.5365	UNFOCUSED
58	Tang L, Zhang Y, Pang Y. Patient-reported outcomes from the distress assessment and response tool program in Chinese cancer inpatients. Psychooncology. 2020 May;29(5):869-877. doi: 10.1002/pon.5358. Epub 2020 Feb 24. PMID: 32040238.	DUPLICATED
59	Boujan N, Géraud C. Neuropsychiatric symptoms, skin disease, and weight loss: Necrolytic migratory erythema and a glucagonoma. The Lancet. 2020;395(10228):985. doi:https://doi.org/10.1016/S0140-6736(20)30324-X	UNFOCUSED
60	Lantheaume S, Montagne M, Shankland R. Intervention centrée sur les ressources pour réduire les troubles anxieux et dépressifs chez les patients atteints de cancer : une étude pilote [Intervention focused on resources to reduce anxiety and depression disorders in cancer patients: A pilot study]. Encephale. 2020 Feb;46(1):13-22. French. doi: 10.1016/j.encep.2019.07.005. Epub 2019 Oct 11. PMID: 31610923.	DUPLICATED
61	Locher C, Gaab J, Blease C, Inderbinen M, Kost L, Koechlin H. Placebos are part of the solution, not the problem. An exemplification of the case of antidepressants in pediatric chronic pain conditions. Frontiers in Psychiatry. 2020;10:998. doi:https://doi.org/10.3389/fpsyt.2019.00998	CASE
62	Incayawar M. The need to fathom overlapping pain and psychiatric syndromes. In: Incayawar M, Maldonado-Bouchard S, Clark MR, eds. Overlapping pain and psychiatric syndromes: Global perspectivesOxford University Press; 2020:3	REVIEW
63	Clinical handbook of psychological consultation in pediatric medical settings. Springer Nature Switzerland AG; 2020	UNFOCUSED
64	Steinhoff A. Late-effect symptoms, tobacco and alcohol use, and demoralization in head and neck cancer survivors. Order No. AAI27994324 ed. University of Kansas; 2020. https://www.proquest.com/dissertations-theses/late-effect-symptoms-tobacco-alcohol-use/docview/2430694966/se-2	UNFOCUSED
65	Johnson CC, Phillips KM, Miller SN. Suicidal Ideation among Veterans Living with Cancer Referred to Mental Health. Clin Gerontol. 2020 Jan-Feb;43(1):24-36. doi: 10.1080/07317115.2019.1686719. Epub 2019 Nov 4. PMID: 31680645.	DUPLICATED
66	Granek L, Nakash O, Ariad S, Shapira S, Ben-David M. Strategies and Barriers in Addressing Mental Health and Suicidality in Patients With Cancer. Oncol Nurs Forum. 2019 Sep 1;46(5):561-571. doi: 10.1188/19.ONF.561-571. PMID: 31424452.	DUPLICATED
67	Koch ED, Kapanadze S, Eerdekens M, et al. Cebranopadol, a novel first-in-class analgesic drug candidate: First experience with cancer-related pain for up to 26 weeks. J Pain Symptom Manage. 2019;58(3):390-399. doi:https://doi.org/10.1016/j.jpainsymman.2019.05.012	UNFOCUSED
68	Sinyor M, Williams M, Gulati S, Schaffer A. An observational study of suicide deaths by self-poisoning with opioids in Toronto (1998-2015). The Canadian Journal of Psychiatry / La Revue canadienne de psychiatrie. 2019;64(8):577-	DUPLICATED

	583. https://www.proquest.com/scholarly-journals/observational-study-suicide-deaths-self-poisoning/docview/2488231953/se-2	
69	Gallaway MS, Fink DS, Sampson L, Cohen GH, Tamburrino M, Liberzon I, Calabrese J, Galea S. Prevalence and covariates of problematic gambling among a US military cohort. Addict Behav. 2019 Aug;95:166-171. doi: 10.1016/j.addbeh.2019.03.013. Epub 2019 Mar 21. PMID: 30928661; PMCID: PMC6574081.	DUPLICATED
70	Martinovic J. A brief history of death and American psychiatry. Harv Rev Psychiatry. 2019;27(4):260. doi:https://doi.org/10.1097/HRP.0000000000000216	REVIEW
71	Choi NG, DiNitto DM, Marti CN, Conwell Y. Physical Health Problems as a Late-Life Suicide Precipitant: Examination of Coroner/Medical Examiner and Law Enforcement Reports. Gerontologist. 2019 Mar 14;59(2):356-367. doi: 10.1093/geront/gnx143. PMID: 28958040.	DUPLICATED
72	Eerdekens M, Kapanadze S, Koch ED, et al. Cancer-related chronic pain: Investigation of the novel analgesic drug candidate cebranopadol in a randomized, double-blind, noninferiority trial. European Journal of Pain. 2019;23(3):577-588. doi:https://doi.org/10.1002/ejp.1331	UNFOCUSED
73	The Cambridge handbook of psychology, health and medicine. 3rd ed. ed. Cambridge University Press; 2019. https://www.proquest.com/books/cambridge-handbook-psychology-health-medicine/docview/2299204776/se-2	UNFOCUSED
74	Kara T, Topkarci Z, Gördü Z, Akaltun İ. Gardner-Diamond syndrome in an adolescent with suicidal ideation: A case report. Journal of Psychiatric Practice. 2019;25(1):54. doi:https://doi.org/10.1097/PRA.0000000000000351	CASE
75	Balducci L. Geriatric Oncology, Spirituality, and Palliative Care. J Pain Symptom Manage. 2019 Jan;57(1):171-175. doi: 10.1016/j.jpainsymman.2018.05.009. Epub 2018 Jun 19. PMID: 29772281.	DUPLICATED
76	Henson KE, Brock R, Charnock J, Wickramasinghe B, Will O, Pitman A. Risk of suicide after cancer diagnosis in England. JAMA Psychiatry. 2019;76(1):51-60. doi:https://doi.org/10.1001/jamapsychiatry.2018.3181	UNFOCUSED
77	Jayatilleke N, Hayes RD, Chang C, Stewart R. Acute general hospital admissions in people with serious mental illness. Psychol Med. 2018;48(16):2676-2683. doi:https://doi.org/10.1017/S0033291718000284	DUPLICATED
78	Mercadante S, Masedu F, Degan G, Marinangeli F, Aielli F. Physicians' attitudes toward euthanasia and assisted suicide in Italy. J Pain Symptom Manage. 2018;56(6):e1-e3. doi:https://doi.org/10.1016/j.jpainsymman.2018.09.007	DUPLICATED
79	Aboumrad M, Shiner B, Riblet N, Mills PD, Watts BV. Factors contributing to cancer-related suicide: A study of root-cause analysis reports. Psychooncology. 2018;27(9):2237-2244. doi:https://doi.org/10.1002/pon.4815	DUPLICATED
80	Adewumi AD, Hollingworth SA, Maravilla JC, Connor JP, Alati R. Prescribed dose of opioids and overdose: A systematic review and meta-analysis of unintentional prescription opioid overdose. CNS Drugs. 2018;32(2):101-116. doi:https://doi.org/10.1007/s40263-018-0499-3	DUPLICATED
81	Ong AD, Standiford T, Deshpande S. Hope and stress resilience. In: Gallagher MW, Lopez SJ, eds. The Oxford handbook of hopeOxford University Press; 2018:255-284, 373 Pages. https://www.proquest.com/books/hope-stress-resilience/docview/2019663473/se-2	REVIEW
82	Balboni MJ, Sullivan A, Smith PT, et al. The views of clergy regarding ethical controversies in care at the end of life. J Pain Symptom Manage. 2018;55(1):65-74. doi:https://doi.org/10.1016/j.jpainsymman.2017.05.009	DUPLICATED
83	Bernatchez MS, Savard J, Ivers H. Disruptions in sleep–wake cycles in community-dwelling cancer patients receiving palliative care and their correlates. Chronobiol Int. 2018;35(1):49-62. doi:https://doi.org/10.1080/07420528.2017.1381615	UNFOCUSED
84	Slater H, Campbell JM, Stinson JN, Burley MM, Briggs AM. End user and implementer experiences of mHealth technologies for noncommunicable chronic disease management in young adults: Systematic review. Journal of Medical Internet Research. 2017;19(12):26. doi:https://doi.org/10.2196/jmir.8888	DUPLICATED
85	Cheung G, Douwes G, Sundram F. Late-life suicide in terminal cancer: A rational act or underdiagnosed depression? J Pain Symptom Manage. 2017;54(6):835-842. doi:https://doi.org/10.1016/j.jpainsymman.2017.05.004	DUPLICATED
86	Baldwin M, Boilini H, Lamvu G. Chronic pain and suicide: Is there a role for Ketamine? Mil Med. 2017;182(11-12):1746. doi:https://doi.org/10.7205/MILMED-D-17-00034	EDITORIAL
87	Braverman DW, Marcus BS, Wakim PG, Mercurio MR, Kopf GS. Health care professionals' attitudes about physician-assisted death: An analysis of their justifications and the roles of terminology and patient competency. J Pain Symptom Manage. 2017;54(4):538-545. doi:https://doi.org/10.1016/j.jpainsymman.2017.07.024	DUPLICATED
88	Lin Y, Kao C, Wu S, Hung S, Yang H, Tung H. Risk factors of post-traumatic stress symptoms in patients with cancer. J Clin Nurs. 2017;26(19-20):3137-3143. doi:https://doi.org/10.1111/jocn.13662	DUPLICATED
89	Aberle D, Wu SE, Oklu R, Erinjeri J, Deipolyi AR. Association between allergies and psychiatric disorders in patients undergoing invasive procedures. Psychosomatics: Journal of Consultation and Liaison Psychiatry. 2017;58(5):490-495. doi:https://doi.org/10.1016/j.psym.2017.03.015	DUPLICATED
90	Shim E, Song YW, Park S, Lee K, Go DJ, Hahm B. Examining the relationship between pain catastrophizing and suicide risk in patients with rheumatic disease: The mediating role of depression, perceived social support, and perceived burdensomeness. Int J Behav Med. 2017;24(4):501-512. doi:https://doi.org/10.1007/s12529-017-9648-1	DUPLICATED

91	Johnson A. The many faces of multiple chemical sensitivity. Ecopsychology. 2017;9(2):60. doi:https://doi.org/10.1089/eco.2016.0040	UNFOCUSED
92	Ruan X, Wu H, Wang D. Suicidal behaviour following traumatic brain injury. Brain Injury. 2017;31(5):717. doi:https://doi.org/10.1080/02699052.2017.1282043	EDITORIAL
93	Racine M, Sánchez-Rodríguez E, Gálan S, et al. Factors associated with suicidal ideation in patients with chronic non-cancer pain. Pain Medicine. 2017;18(2):283-293. https://www.proquest.com/scholarly-journals/factors-associated-with-suicidal-ideation/docview/2008438839/se-2	DUPLICATED
94	Riquin É, Hubault P, Baize N, Lhuillier J, Duverger P. Particularités de l'accompagnement en soins palliatifs d'un patient atteint de schizophrénie en secteur psychiatrique. Annales Médico-Psychologiques. 2017;175(2):140-145. doi:https://doi.org/10.1016/j.amp.2016.06.003	UNFOCUSED
95	Practical psychology in medical rehabilitation. Springer International Publishing/Springer Nature; 2017	UNFOCUSED
96	Chresfield R, Rae. Adjustment and acceptance of chronic pain: The lived experience of adults 30 to 39 years of age. Order No. AAI10127808 ed. State University of New York at Buffalo; 2017. https://www.proquest.com/dissertations-theses/adjustment-acceptance-chronic-pain-lived/docview/1886292331/se-2	UNFOCUSED
97	Emanuel EJ, Onwuteaka-Philipsen B, Urwin JW, Cohen J. Attitudes and practices of euthanasia and physician-assisted suicide in the United States, Canada, and Europe. JAMA: Journal of the American Medical Association. 2016;316(1):79-90. doi:https://doi.org/10.1001/jama.2016.8499	DUPLICATED
98	Elhoseiny A, Elleithy W, Tantawy A, Mohamed KAE. Assessment of suicidal risk factors in cancer patients attending oncology unit in Suez Canal University Hospital. Arab Journal of Psychiatry. 2016;27(1):41-52. doi:https://doi.org/10.12816/0023155	UNFOCUSED
99	Aly Z, Rosen N, Evans RW. Migraine and the risk of suicide. Headache: The Journal of Head and Face Pain. 2016;56(4):753-761. doi:https://doi.org/10.1111/head.12815	EDITORIAL
100	Spangenberg L, Zenger M, Garcia-Torres F, et al. Dimensionality, stability, and validity of the Beck Hopelessness Scale in cancer patients receiving curative and palliative treatment. J Pain Symptom Manage. 2016;51(3):615-622. doi:https://doi.org/10.1016/j.jpainsymman.2015.11.008	DUPLICATED
101	Lichtenthal WG, Corner GW, Sweeney C, Roberts KE. Grieving the traumatic death of a child. In: Black BP, Wright PM, Limbo R, eds. Perinatal and pediatric bereavement in nursing and other health professionsSpringer Publishing Company; 2016:249. https://www.proquest.com/books/grieving-traumatic-death-child/docview/1773463529/se-2	UNFOCUSED
102	Mindfulness-based cognitive therapy: Innovative applications. Springer International Publishing/Springer Nature; 2016	UNFOCUSED
103	Advanced practice palliative nursing. Oxford University Press; 2016	UNFOCUSED
104	McCarthy M. Will Public Health England lead research? The Lancet. 2015;386(10010):2232. doi:https://doi.org/10.1016/S0140-6736(15)00196-8	EDITORIAL
105	Campbell G, Bruno R, Darke S, Degenhardt L. Associations of borderline personality with pain, problems with medications and suicidality in a community sample of chronic non-cancer pain patients prescribed opioids for pain. Gen Hosp Psychiatry. 2015;37(5):434-440. doi:https://doi.org/10.1016/j.genhosppsych.2015.05.004	DUPLICATED
106	Campbell G, Darke S, Bruno R, Degenhardt L. The prevalence and correlates of chronic pain and suicidality in a nationally representative sample. Aust N Z J Psychiatry. 2015;49(9):803-811. doi:https://doi.org/10.1177/0004867415569795	DUPLICATED
107	Quill TE. Voluntary Stopping of Eating and Drinking (VSED), Physician-Assisted Death (PAD), or neither in the last stage of life? Both should be available as a last resort. Annals of Family Medicine. 2015;13(5):408-409. doi:https://doi.org/10.1370/afm.1850	CASE
108	Smolensky MH, Portaluppi F, Manfredini R, et al. Diurnal and twenty-four hour patterning of human diseases: Acute and chronic common and uncommon medical conditions. Sleep Medicine Reviews. 2015;21:12-22. doi:https://doi.org/10.1016/j.smrv.2014.06.005	DUPLICATED
109	Barton-Burke M. Oncology nursing as ethical practice. Oncol Nurs Forum. 2015;42(3):214. https://www.proquest.com/scholarly-journals/oncology-nursing-as-ethical-practice/docview/1709216410/se-2	UNFOCUSED
110	Smith KA, Harvath TA, Goy ER, Ganzini L. Predictors of pursuit of physician-assisted death. J Pain Symptom Manage. 2015;49(3):555-561. doi:https://doi.org/10.1016/j.jpainsymman.2014.06.010	DUPLICATED
111	Psychiatric care of the medical patient. 3rd ed. ed. Oxford University Press; 2015	UNFOCUSED
112	Handbook of oncology social work: Psychosocial care for people with cancer. Oxford University Press; 2015. https://www.proquest.com/books/handbook-oncology-social-work-psychosocial-care/docview/1746992339/se-2	UNFOCUSED
113	Bowles DJ. Gerontology nursing case studies: 100+ narratives for learning. 2nd ed. ed. Springer Publishing Company; 2015. https://www.proquest.com/books/gerontology-nursing-case-studies-100-narratives/docview/1735928978/se-2	UNFOCUSED

114	Psycho-oncology. 3rd ed. ed. Oxford University Press; 2015. https://www.proquest.com/books/psycho-oncology/docview/1690660445/se-2	UNFOCUSED
115	Parpa E, Tsilika E, Gennimata V, Mystakidou K. Elderly cancer patients' psychopathology: A systematic review Aging and mental health. Arch Gerontol Geriatr. 2015;60(1):9-15. doi:https://doi.org/10.1016/j.archger.2014.09.008	DUPLICATED
116	Omerov P, Steineck G, Dyregrov K, Runeson B, Nyberg U. The ethics of doing nothing. Suicide-bereavement and research: Ethical and methodological considerations. Psychol Med. 2014;44(16):3409-3420. doi:https://doi.org/10.1017/S0033291713001670	DUPLICATED
117	Ekholm O, Kurita GP, Højsted J, Juel K, Sjøgren P. Chronic pain, opioid prescriptions, and mortality in Denmark: A population-based cohort study. Pain. 2014;155(12):2486-2490. doi:https://doi.org/10.1016/j.pain.2014.07.006	DUPLICATED
118	Breivik H, Stubhaug A. Burden of disease is often aggravated by opioid treatment of chronic pain patients: Etiology and prevention. Pain. 2014;155(12):2441. doi:https://doi.org/10.1016/j.pain.2014.09.011	EDITORIAL
119	Castaneto MS, Gorelick DA, Desrosiers NA, Hartman RL, Pirard S, Huestis MA. Synthetic cannabinoids: Epidemiology, pharmacodynamics, and clinical implications. Drug Alcohol Depend. 2014;144:12-41. doi:https://doi.org/10.1016/j.drugalcdep.2014.08.005	DUPLICATED
120	Recklitis CJ, Zhou ES, Zwemer EK, Hu JC, Kantoff PW. Suicidal ideation in prostate cancer survivors: Understanding the role of physical and psychological health outcomes. Cancer. 2014;120(21):3393-3400. doi:https://doi.org/10.1002/cncr.28880	DUPLICATED
121	Sharpe M, Walker J, Hansen CH, et al. Integrated collaborative care for comorbid major depression in patients with cancer (SMaRT Oncology-2): A multicentre randomised controlled effectiveness trial. The Lancet. 2014;384(9948):1099-1108. doi:https://doi.org/10.1016/S0140-6736(14)61231-9	DUPLICATED
122	Margari F, Lorusso M, Matera E, et al. Aggression, impulsivity, and suicide risk in benign chronic pain patients—A cross-sectional study. Neuropsychiatric Disease and Treatment. 2014;10:8. https://www.proquest.com/scholarly-journals/aggression-impulsivity-suicide-risk-benign/docview/1641024966/se-2	UNFOCUSED
123	Abbott CH, Prigerson HG, Maciejewski PK. The influence of patients' quality of life at the end of life on bereaved caregivers' suicidal ideation. J Pain Symptom Manage. 2014;48(3):459-464. doi:https://doi.org/10.1016/j.jpainsymman.2013.09.011	DUPLICATED
124	Webster LR. Pain and suicide: The other side of the opioid story. Pain Medicine. 2014;15(3):345. doi:https://doi.org/10.1111/pme.12398	UNFOCUSED
125	Jaiswal R, Alici Y, Breitbart W. A comprehensive review of palliative care in patients with cancer. International Review of Psychiatry. 2014;26(1):87-101. doi:https://doi.org/10.3109/09540261.2013.868788	DUPLICATED
126	Tillman J, Carter A. The trauma of patient suicide. In: Deutsch RA, ed. Traumatic ruptures: Abandonment and betrayal in the analytic relationshipRoutledge/Taylor & Francis Group; 2014:66. https://www.proquest.com/books/trauma-patient-suicide/docview/1661994101/se-2	UNFOCUSED
127	The Oxford handbook of depression and comorbidity. Oxford University Press; 2014	UNFOCUSED
128	DSM-5™ clinical cases. American Psychiatric Publishing, Inc; 2014. https://www.proquest.com/books/dsm-5™-clinical-cases/docview/1554230254/se-2	CASE
129	Breitbart WS, Alici Y. Psychosocial palliative care. Oxford University Press; 2014	UNFOCUSED
130	Covington C. Shrinking the news: Headline stories on the couch. Karnac Books; 2014. https://www.proquest.com/books/shrinking-news-headline-stories-on-couch/docview/1528881890/se-2	UNFOCUSED
131	Translating psychological research into practice. Springer Publishing Company; 2014. https://www.proquest.com/books/translating-psychological-research-into-practice/docview/1518034488/se-2	UNFOCUSED
132	No ai. Abstracts of the IPOS 15th World Congress of Psycho-Oncology, 4-8 November 2013, Rotterdam, The Netherlands. Psychooncology. 2013;22:1. doi:https://doi.org/10.1111/j.1099-1611.2013.3393	UNFOCUSED
133	MacGregor C. Remembering Lithuania: Fathers, groups and the 'patrix'. Group Analysis. 2013;46(3):18. doi:https://doi.org/10.1177/0533316413498843h	EDITORIAL
134	Ilgen MA, Kleinberg F, Ignacio RV, et al. Noncancer pain conditions and risk of suicide. JAMA Psychiatry. 2013;70(7):692-697. doi:https://doi.org/10.1001/jamapsychiatry.2013.908	UNFOCUSED
135	No ai. Physician-assisted suicide. N Engl J Med. 2013;368(15):1450. https://www.proquest.com/scholarly-journals/physician-assisted-suicide/docview/1366315925/se-2	EDITORIAL
136	Ventegodt S, Merrick J. Advice about medicine. In: Merrick J, ed. Alternative medicine yearbook, 2011Nova Biomedical Books; 2013:533. https://www.proquest.com/books/advice-about-medicine/docview/1531962352/se-2	UNFOCUSED
137	Cheatle MD. Pain and addiction. In: Miller PM, Ball SA, Bates ME, et al, eds. Comprehensive addictive behaviors and disorders, Vol. 3: Interventions for addictionElsevier Academic Press; 2013:503	UNFOCUSED

138	University and college students: Health and development issues for the leaders of tomorrow. Nova Biomedical Books; 2013. https://www.proquest.com/books/university-college-students-health-development/docview/1562145354/se-2	UNFOCUSED
139	Final acts: The end of life, hospice and palliative care. Baywood Publishing Co; 2013. https://www.proquest.com/books/final-acts-end-life-hospice-palliative-care/docview/1554230626/se-2	UNFOCUSED
140	Chiu PH. Where there is hope, there is life: Chinese and Western perspectives on living through terminal illness in China. Order No. AAI3528741 ed. ; 2013. https://www.proquest.com/dissertations-theses/where-there-is-hope-life-chinese-western/docview/1417863451/se-2	UNFOCUSED
141	Johnstone M. Organization position statements and the stance of "studied neutrality" on euthanasia in palliative care. J Pain Symptom Manage. 2012;44(6):896. doi:https://doi.org/10.1016/j.jpainsymman.2011.12.276	UNFOCUSED
142	Langford DJ, Lee K, Miaskowski C. Sleep disturbance interventions in oncology patients and family caregivers: A comprehensive review and meta-analysis. Sleep Medicine Reviews. 2012;16(5):397-414. doi:https://doi.org/10.1016/j.smrv.2011.07.002	REVIEW
143	Kowal J, Wilson KG, McWilliams LA, Péloquin K, Duong D. Self-perceived burden in chronic pain: Relevance, prevalence, and predictors. Pain. 2012;153(8):1735-1741. doi:https://doi.org/10.1016/j.pain.2012.05.009	DUPLICATED
144	Čupić B, Hranilovic D, Jernej B, Gabrilovac J. Association study of genes regulating opioid system in autism. Psychiatry Res. 2012;198(1):169-170. doi:https://doi.org/10.1016/j.psychres.2011.11.003	UNFOCUSED
145	Park EM, Suzuki J. Treatment of opioid dependence and cancer pain with sublingual buprenorphine: A case report. The American Journal on Addictions. 2012;21(3):283-284. doi:https://doi.org/10.1111/j.1521-0391.2012.00219.x	CASE
146	Jansson C, Mittendorfer-Rutz E, Alexanderson K. Sickness absence because of musculoskeletal diagnoses and risk of all-cause and cause-specific mortality: A nationwide Swedish cohort study. Pain. 2012;153(5):998-1005. doi:https://doi.org/10.1016/j.pain.2012.01.028	DUPLICATED
147	Johnson TV, Garlow SJ, Brawley OW, Master VA. Peak window of suicides occurs within the first month of diagnosis: Implications for clinical oncology. Psychooncology. 2012;21(4):351-356. doi:https://doi.org/10.1002/pon.1905	DUPLICATED
148	Mental health disorders sourcebook. 5th ed. ed. Omnigraphics; 2012. https://www.proquest.com/books/mental-health-disorders-sourcebook/docview/1803466925/se-2	UNFOCUSED
149	Depression sourcebook. 3rd ed. ed. Omnigraphics; 2012. https://www.proquest.com/books/depression-sourcebook/docview/1803466433/se-2	UNFOCUSED
150	Alternative medicine yearbook, 2010. Nova Biomedical Books; 2012. https://www.proquest.com/books/alternative-medicine-yearbook-2010/docview/1544984470/se-2	UNFOCUSED
151	Koenig HG, King DE, Carson VB. Handbook of religion and health. 2nd ed. ed. Oxford University Press; 2012. https://www.proquest.com/books/handbook-religion-health/docview/1240219129/se-2	UNFOCUSED
152	de la C. Barroso Fernández, Irene, Abalo JG. Eutanasia y cuidados paliativos: ¿Diferentes aristas de un mismo problema? Psicología y Salud. 2012;22(1):5. https://www.proquest.com/scholarly-journals/eutanasia-y-cuidados-paliativos-diferentes/docview/1081621537/se-2	UNFOCUSED
153	Priebe LC. Investigating prevalence rates and biopsychosocial risk factors for suicidality in newly-diagnosed cancer outpatients. Order No. AAI3454207 ed. ; 2012. https://www.proquest.com/dissertations-theses/investigating-prevalence-rates-biopsychosocial/docview/928985502/se-2	UNFOCUSED
154	Madeira N, Albuquerque E, Santos T, Mendes A, Roque M. Death ideation in cancer patients: Contributing factors. J Psychosoc Oncol. 2011;29(6):636-642. doi:https://doi.org/10.1080/07347332.2011.615381	DUPLICATED
155	Trinidad AC, Simopoulos EF, Flosnik DL. The use of antidepressants in cancer treatment. Psychiatric Annals. 2011;41(9):443-446. doi:https://doi.org/10.3928/00485713-20110829-06	CASE
156	Baumrucker SJ, Stolick M, Carter GT, Mingle P, Oertli KA, Morris GM. Legal but not always allowed: "Physician aid in dying". American Journal of Hospice & Palliative Medicine. 2011;28(6):449-454. doi:https://doi.org/10.1177/1049909111412540	CASE
157	Monforte-Royo C, Villavicencio-Chávez C, Tomás-Sábado J, Balaguer A. The wish to hasten death: A review of clinical studies. Psychooncology. 2011;20(8):795-804. doi:https://doi.org/10.1002/pon.1839	DUPLICATED
158	Yun YH, Han KH, Park S, et al. Attitudes of cancer patients, family caregivers, oncologists and members of the general public toward critical interventions at the end of life of terminally ill patients. Can Med Assoc J. 2011;183(10). doi:https://doi.org/10.1503/cmaj.110020	DUPLICATED
159	Schneider S, Berke JH. Freud's atonement. Mental Health, Religion & Culture. 2011;14(6):531. doi:https://doi.org/10.1080/13674676.2010.491967	UNFOCUSED
160	Chambaere K, Bilsen J, Cohen J, Onwuteaka-Philipsen B, Mortier F, Deliens L. Trends in medical end-of-life decision making in Flanders, Belgium 1998–2001–2007. Medical Decision Making. 2011;31(3):500. doi:https://doi.org/10.1177/0272989X10392379	DUPLICATED

161	Dettmore D, Gabriele LC. Don't just do something, stand there: Responding to unrelieved patient suffering. J Psychosoc Nurs Ment Health Serv. 2011;49(4):34. doi:https://doi.org/10.3928/02793695-20110302-01	DUPLICATED
162	Abernethy AP. Critical summaries of innovations in palliative care pharmacotherapy. Journal of Pain & Palliative Care Pharmacotherapy. 2011;25(1):55-60. doi:https://doi.org/10.3109/15360288.2010.549550	REVIEW
163	Muller D. Attention to language in a request for physician aid in dying. American Journal of Hospice & Palliative Medicine. 2011;28(1):63. doi:https://doi.org/10.1177/1049909110381080	EDITORIAL
164	Bowles DJ. Gerontology nursing case studies: 100 narratives for learning. Springer Publishing Company; 2011. https://www.proquest.com/books/gerontology-nursing-case-studies-100-narratives/docview/865692388/se-2	CASE
165	Embitterment: Societal, psychological, and clinical perspectives. Springer-Verlag Publishing/Springer Nature; 2011	UNFOCUSED
166	Bourgeois JA, Hales RE, Shahrokh NC. Study guide to psychosomatic medicine: A companion toThe American Psychiatric Publishing textbook of psychosomatic medicine (2nd ed.). American Psychiatric Publishing, Inc; 2011. https://www.proquest.com/books/study-guide-psychosomatic-medicine-companion-i/docview/817614554/se-2	UNFOCUSED
167	Stress-related disorders sourcebook. 3rd ed. ed. Omnigraphics; 2011. https://www.proquest.com/books/stress-related-disorders-sourcebook/docview/1783421593/se-2	UNFOCUSED
168	van Tol D, Rietjens J, van der Heide A. Judgment of unbearable suffering and willingness to grant a euthanasia request by Dutch general practitioners. Health Policy. 2010;97(2-3):166-172. doi:https://doi.org/10.1016/j.healthpol.2010.04.007	DUPLICATED
169	Parpa E, Mystakidou K, Tsilika E, et al. Attitudes of health care professionals, relatives of advanced cancer patients and public towards euthanasia and physician assisted suicide. Health Policy. 2010;97(2-3):160-165. doi:https://doi.org/10.1016/j.healthpol.2010.04.008	DUPLICATED
170	Botega NJ, Mitsuushi GN, de Azevedo R,Cruz Soares, et al. Depression, alcohol use disorders and nicotine dependence among patients at a general hospital. Revista Brasileira de Psiquiatria. 2010;32(3):250-256. doi:https://doi.org/10.1590/S1516-44462010005000016	DUPLICATED
171	Scott KM, Hwang I, Chiu W, et al. Chronic physical conditions and their association with first onset of suicidal behavior in the world Mental Health Surveys. Psychosom Med. 2010;72(7):712. doi:https://doi.org/10.1097/PSY.0b013e3181e3333d	DUPLICATED
172	Onwuteaka-Philipsen B, Rurup ML, Pasman HR, van der Heide A. The last phase of life: Who requests and who receives euthanasia or physician-assisted suicide? Med Care. 2010;48(7):596-603. doi:https://doi.org/10.1097/MLR.0b013e3181dbea75	DUPLICATED
173	Akechi T, Okamura H, Nakano T, et al. Gender differences in factors associated with suicidal ideation in major depression among cancer patients. Psychooncology. 2010;19(4):384-389. doi:https://doi.org/10.1002/pon.1587	DUPLICATED
174	Dees M, Vernooij-Dassen M, Dekkers W, van Weel C. Review unbearable suffering of patients with a request for euthanasia or physician-assisted suicide: An integrative review. Psychooncology. 2010;19(4):339-352. doi:https://doi.org/10.1002/pon.1612	REVIEW
175	McCown D, Reibel D. Mindfulness and mindfulness-based stress reduction. In: Monti DA, Beitman BD, eds. Integrative psychiatryOxford University Press; 2010:289. https://www.proquest.com/books/mindfulness-based-stress-reduction/docview/622134155/se-2	UNFOCUSED
176	Koh M, Portenoy RK. Cancer pain syndromes. In: Bruera ED, Portenoy RK, eds. Cancer pain: Assessment and management (2nd ed.)2nd ed. ed. Cambridge University Press; 2010:53. https://www.proquest.com/books/cancer-pain-syndromes/docview/1692300170/se-2	UNFOCUSED
177	Psycho-oncology. 2nd ed. ed. Oxford University Press; 2010	UNFOCUSED
178	Stress consequences: Mental, neuropsychological and socioeconomic. Elsevier Academic Press; 2010. https://www.proquest.com/books/stress-consequences-mental-neuropsychological/docview/1751225712/se-2	UNFOCUSED
179	Seale C. Continuous deep sedation in medical practice: A descriptive study. J Pain Symptom Manage. 2010;39(1):44-53. doi:https://doi.org/10.1016/j.jpainsymman.2009.06.007	DUPLICATED
180	LaSalvia EA, Domek GJ, Gitlin DF. Fluoroquinolone-induced suicidal ideation. Gen Hosp Psychiatry. 2010;32(1):108. doi:https://doi.org/10.1016/j.genhosppsych.2009.03.002	CASE
181	Berman L, Silverman M, Joiner T. Edwin S. Shneidman (1918–2009). Am Psychol. 2010;65(1):50-51. doi:https://doi.org/10.1037/a0017792	UNFOCUSED
182	Medoff MH. Biased abortion counseling laws and abortion demand. The Social Science Journal. 2009;46(4):632-643. doi:https://doi.org/10.1016/j.soscij.2009.05.001	UNFOCUSED
183	Pousset G, Bilsen J, De Wilde J, et al. Attitudes of adolescent cancer survivors toward end-of-life decisions for minors. Pediatrics. 2009;124(6):e1142-e1148. doi:https://doi.org/10.1542/peds.2009-0621	DUPLICATED
184	Ganzini L, Goy ER, Dobscha SK, Prigerson H. Mental health outcomes of family members of Oregonians who request physician aid in dying. J Pain Symptom Manage. 2009;38(6):807. doi:https://doi.org/10.1016/j.jpainsymman.2009.04.026	DUPLICATED

185	Ventegodt S, Andersen NJ, Kandel I, Merrick J. Effect, side effects and adverse events of non-pharmaceutical medicine. A review. International Journal on Disability and Human Development. 2009;8(3):227. doi:https://doi.org/10.1515/IJDHD.2009.8.3.227	REVIEW
186	No ai. In memoriam. Suicide and Life-Threatening Behavior. 2009;39(3). doi:https://doi.org/10.1521/suli.2009.39.3.iii	UNFOCUSED
187	Bendiane MK, Bouhnik A, Galinier A, Favre R, Obadia Y, Peretti-Watel P. French hospital nurses' opinion about euthanasia and physician-assisted suicide: A national phone survey. Journal of Medical Ethics: Journal of the Institute of Medical Ethics. 2009;35(4):238-244. doi:https://doi.org/10.1136/jme.2008.025296	EDITORIAL
188	Nuhu FT, Odejide OA, Adebayo KO, Yusuf AJ. Psychological and physical effects of pain on cancer patients in Ibadan, Nigeria. African Journal of Psychiatry. 2009;12(1):64-70. doi:https://doi.org/10.4314/ajpsy.v12i1.30281	DUPLICATED
189	Clinical psychology in practice. Blackwell Publishing British Psychological Society; 2009. https://www.proquest.com/books/clinical-psychology-practice/docview/622154430/se-2	UNFOCUSED
190	Clinical handbook of mindfulness. Springer Science + Business Media; 2009	UNFOCUSED
191	György B. Depresszió és fájdalom daganatosbetegségekben: Új kezelési perspektívák. Psychiatria Hungarica. 2009;24:37. https://www.proquest.com/scholarly-journals/depresszió-és-fájdalom-daganatosbetegségekben-új/docview/852910132/se-2	UNFOCUSED
192	Gilbert JW, Wheeler GR, Storey BB, et al. Suicidality in chronic noncancer pain patients. Int J Neurosci. 2009;119(10):1968-1979. doi:https://doi.org/10.1080/00207450902973336	REVIEW
193	Braden JB, Sullivan MD. Suicidal thoughts and behavior among adults with self-reported pain conditions in the national comorbidity survey replication. The Journal of Pain. 2008;9(12):1106-1115. doi:https://doi.org/10.1016/j.jpain.2008.06.004	UNFOCUSED
194	Pompili M, Serafini G, Forte A, Del Casale A, Innamorati M, Tatarelli R. Rischio di suicidio in pazienti affetti da cancro. Medicina Psicosomatica. 2008;53(3):105-116. https://www.proquest.com/scholarly-journals/rischio-di-suicidio-pazienti-affetti-da-cancro/docview/621605599/se-2	REVIEW
195	de Veer, Anke J. E., Francke AL, Poortvliet E. Nurses' involvement in end-of-life decisions. Cancer Nurs. 2008;31(3):222-228. doi:https://doi.org/10.1097/01.NCC.0000305724.83271.f9	DUPLICATED
196	Moussas GI, Karkanias AP, Papadopoulou A. Psychological and psychiatric problems in cancer patients in the general hospital. Psychiatriki. 2008;19(2):124. https://www.proquest.com/scholarly-journals/psychological-psychiatric-problems-cancer/docview/621762445/se-2	DUPLICATED
197	Cohen J, Bilsen J, Fischer S, et al. "End-of-life decision-making in Belgium, Denmark, Sweden and Switzerland: Does place of death make a difference?": Correction. J Epidemiol Community Health. 2008;62(3):280. https://www.proquest.com/scholarly-journals/end-life-decision-making-belgium-denmark-sweden/docview/622215345/se-2	UNFOCUSED
198	Voaklander DC, Rowe BH, Dryden DM, Pahal J, Saar P, Kelly KD. Medical illness, medication use and suicide in seniors: A population-based case-control study. J Epidemiol Community Health. 2008;62(2):138-146. doi:https://doi.org/10.1136/jech.2006.055533	CASE
199	Pain and palliative care pharmacotherapy literature summaries and analyses. Journal of Pain & Palliative Care Pharmacotherapy. 2008;22(3):226-231. doi:https://doi.org/10.1080/15360280802251249	REVIEW
200	Fiske A, O'Riley AA, Widoe RK. Physical health and suicide in late life: An evaluative review. Clinical Gerontologist: The Journal of Aging and Mental Health. 2008;31(4):31. doi:https://doi.org/10.1080/07317110801947151	REVIEW
201	Cohen J, Bilsen J, Fischer S, et al. End-of-life decision-making in Belgium, Denmark, Sweden and Switzerland: Does place of death make a difference? J Epidemiol Community Health. 2007;61(12):1062-1068. doi:https://doi.org/10.1136/jech.2006.056341	UNFOCUSED
202	1. Poinsot R, Antoine P. Une approche psychothérapique méconnue: La résolution de problèmes sociaux. Annales Médico-Psychologiques. 2007;165(9):638. doi:https://doi.org/10.1016/j.amp.2006.05.017	UNFOCUSED
203	Sareen J, Cox BJ, Stein MB, Afifi TO, Fleet C, Asmundson GJG. Physical and mental comorbidity, disability, and suicidal behavior associated with posttraumatic stress disorder in a large community sample. Psychosom Med. 2007;69(3):242. doi:https://doi.org/10.1097/PSY.0b013e31803146d8	DUPLICATED
204	Carter GL, Clover KA, Parkinson L, et al. Mental health and other clinical correlates of euthanasia attitudes in an Australian outpatient cancer population. Psychooncology. 2007;16(4):295-303. doi:https://doi.org/10.1002/pon.1058	DUPLICATED
205	Societal economic costs and benefits from death: Another look. Death Stud. 2007;31(4):363. doi:https://doi.org/10.1080/07481180601187217	EDITORIAL
206	Psychological challenges in obstetrics and gynecology: The clinical management. Springer Science + Business Media; 2007	UNFOCUSED
207	Rosenfeld B, Breitbart W, Gibson C, et al. Desire for Hastened Death Among Patients with Advanced AIDS. Psychosomatics: Journal of Consultation and Liaison Psychiatry. 2006;47(6):504-512. doi:https://doi.org/10.1176/appi.psy.47.6.504	DUPLICATED

208	Hudson PL, Kristjanson LJ, Ashby M, et al. Desire for hastened death in patients with advanced disease and the evidence base of clinical guidelines: A systematic review. Palliat Med. 2006;20(7):693-701. doi:https://doi.org/10.1177/0269216306071799	REVIEW
209	Ganzini L, Beer TM, Brouns MC. Views on Physician-Assisted Suicide Among Family Members of Oregon Cancer Patients. J Pain Symptom Manage. 2006;32(3):230-236. doi:https://doi.org/10.1016/j.jpainsymman.2006.04.004	UNFOCUSED
210	Parpa E, Mystakidou K, Tsilika E, et al. The Attitudes of Greek Physicians and Lay People on Euthanasia and Physician-Assisted Suicide in Terminally Ill Cancer patients. American Journal of Hospice & Palliative Medicine. 2006;23(4):297-303. doi:https://doi.org/10.1177/1049909106290247	DUPLICATED
211	Iakovidis VI. Depression in cancer patients. Psychiatriki. 2006;17(2):102. https://www.proquest.com/scholarly-journals/depression-cancer-patients/docview/621463453/se-2	UNFOCUSED
212	Bilsen J, Norup M, Deliens L, et al. Drugs Used to Alleviate Symptoms with Life Shortening as a Possible Side Effect: End-of-Life Care in Six European Countries. J Pain Symptom Manage. 2006;31(2):111-121. doi:https://doi.org/10.1016/j.jpainsymman.2005.07.005	UNFOCUSED
213	The handbook of adult clinical psychology: An evidence-based practice approach. Routledge/Taylor & Francis Group; 2006. https://www.proquest.com/books/handbook-adult-clinical-psychology-evidence-based/docview/621429046/se-2	UNFOCUSED
214	Christopherson J. Mortality consequences of low self-control. Order No. AAI3214062 ed. ; 2006. https://www.proquest.com/dissertations-theses/mortality-consequences-low-self-control/docview/621576033/se-2	UNFOCUSED
215	Symons FJ, Danov SE. A prospective clinical analysis of pain behavior and self-injurious behavior. Pain. 2005;117(3):473-477. doi:https://doi.org/10.1016/j.pain.2005.07.010	DUPLICATED
216	Clarke DM, McLeod JE, Smith GC, Trauer T, Kissane DW. A Comparison of Psychosocial and Physical Functioning in Patients With Motor Neurone Disease and Metastatic Cancer. J Palliat Care. 2005;21(3):173-179. doi:https://doi.org/10.1177/082585970502100310	UNFOCUSED
217	Johansen S, Hølen JC, Kaasa S, Loge JH, Materstvedt LJ. Attitudes towards, and wishes for, euthanasia in advanced cancer patients at a palliative medicine unit. Palliat Med. 2005;19(6):454-460. doi:https://doi.org/10.1191/0269216305pm1048oa	DUPLICATED
218	Sullivan MD. The desire for death arises from an intolerable future rather than an intolerable present. Gen Hosp Psychiatry. 2005;27(4):256. doi:https://doi.org/10.1016/j.genhosppsych.2005.04.002	UNFOCUSED
219	O'Mahony S, Goulet J, Kornblith A, et al. Desire for Hastened Death, Cancer Pain and Depression: Report of a Longitudinal Observational Study. J Pain Symptom Manage. 2005;29(5):446-457. doi:https://doi.org/10.1016/j.jpainsymman.2004.08.010	UNFOCUSED
220	Llorente MD, Burke M, Gregory GR, et al. Prostate Cancer: A Significant Risk Factor for Late-Life Suicide. The American Journal of Geriatric Psychiatry. 2005;13(3):195-201. doi:https://doi.org/10.1176/appi.ajgp.13.3.195	DUPLICATED
221	Okereke OI, Folsom DP. Medical Comorbidity in Geriatric Psychiatry. The American Journal of Geriatric Psychiatry. 2005;13(3):177. doi:https://doi.org/10.1176/appi.ajgp.13.3.177	DUPLICATED
222	Taylor BR, Mccann RM. Controlled Sedation for Physical and Existential Suffering? J Palliat Med. 2005;8(1):144-147. doi:https://doi.org/10.1089/jpm.2005.8.144	CASE
223	Lester D. Easing the Legacy of Suicide. In: Yufit RI, Lester D, eds. Assessment, treatment, and prevention of suicidal behaviorJohn Wiley & Sons, Inc; 2005:337. https://www.proquest.com/books/easing-legacy-suicide/docview/620592390/se-2	UNFOCUSED
224	Bourgeois JA, Hales RE, Shahrokh NC. Study Guide to the American Psychiatric Publishing Textbook of Psychosomatic Medicine. American Psychiatric Publishing, Inc; 2005. https://www.proquest.com/books/study-guide-american-psychiatric-publishing/docview/620770336/se-2	UNFOCUSED
225	The American psychiatric publishing textbook of psychosomatic medicine. American Psychiatric Publishing, Inc; 2005. https://www.proquest.com/books/american-psychiatric-publishing-textbook/docview/620599433/se-2	UNFOCUSED
226	Akizuki N, Yamawaki S, Akechi T, Nakano T, Uchitomi Y. Development of an Impact Thermometer for Use in Combination with the Distress Thermometer as a Brief Screening Tool for Adjustment Disorders and/or Major Depression in Cancer Patients. J Pain Symptom Manage. 2005;29(1):91-99. doi:https://doi.org/10.1016/j.jpainsymman.2004.04.016	DUPLICATED
227	Sato K, Higuchi H, Yoshida K, Takahashi H, Shimizu T, Watanabe J. Milnacipran treatment of a terminally ill cancer patient with major depressive disorder. Hum Psychopharmacol Clin Exp. 2004;19(6):431-432. doi:https://doi.org/10.1002/hup.605	EDITORIAL
228	Mayland CR, Mason SR. Suicidal Intent in the Palliative Care Setting. J Palliat Care. 2004;20(2):119-120. https://www.proquest.com/scholarly-journals/suicidal-intent-palliative-care-setting/docview/620455124/se-2	CASE
229	Arnold EM, Artin KA, Person JL, Griffith DL. Consideration of Hastening Death Among Hospice Patients and Their Families. J Pain Symptom Manage. 2004;27(6):523-532. doi:https://doi.org/10.1016/j.jpainsymman.2003.10.010	DUPLICATED

230	Bilsen J, Stichele RV, Mortier F, Bernheim J, Deliens L. The incidence and characteristics of end-of-life decisions by GPs in Belgium. Fam Pract. 2004;21(3):282-289. doi:https://doi.org/10.1093/fampra/cmh312	UNFOCUSED
231	Grassi L, Biancosino B, Marmai L, Righi R. Effect of reboxetine on major depressive disorder in breast cancer patients: An open-label study. J Clin Psychiatry. 2004;65(4):515-520. doi:https://doi.org/10.4088/JCP.v65n0410	DUPLICATED
232	lacovides V, Kallerghis G, Anagnostopoulos P, Bendenides C. Psychiatric emergencies in psycho-oncology: A comparative study. Psychiatriki. 2004;15(2):143-154. https://www.proquest.com/scholarly-journals/psychiatric-emergencies-psycho-oncology/docview/620479923/se-2	UNFOCUSED
233	Smith MT, Perils ML, Haythornthwaite JA. Suicidal Ideation in Outpatients With Chronic Musculoskeletal Pain: An Exploratory Study of the Role of Sleep Onset Insomnia and Pain Intensity. Clin J Pain. 2004;20(2):111-118. doi:https://doi.org/10.1097/00002508-200403000-00008	DUPLICATED
234	Handbook of primary care psychology. Oxford University Press; 2004. https://www.proquest.com/books/handbook-primary-care-psychology/docview/620715505/se-2	UNFOCUSED
235	Khanna P, Bulow K. Attempted Suicide by a Terminally Ill Patient. J Palliat Med. 2003;6(4):629. doi:https://doi.org/10.1089/109662103768253777	UNFOCUSED
236	Caffo E, Belaise C. Psychological aspects of traumatic injury in children and adolescents. Child Adolesc Psychiatr Clin N Am. 2003;12(3):493-535. doi:https://doi.org/10.1016/S1056-4993(03)00004-X	DUPLICATED
237	Morita T, Hirai K, Akechi T, Uchitomi Y. Similarity and Difference Among Standard Medical Care, Palliative Sedation Therapy, and Euthanasia: A Multidimensional Scaling Analysis on Physicians' and the General Population's Opinions. J Pain Symptom Manage. 2003;25(4):357-363. doi:https://doi.org/10.1016/S0885-3924(02)00684-X	REVIEW
238	Essinger DM. Attitudes of Tennessee physicians towards euthanasia and assisted death. Order No. AAI3054093 ed. ; 2002. https://www.proquest.com/dissertations-theses/attitudes-tennessee-physicians-towards-euthanasia/docview/619963807/se-2	UNFOCUSED
239	Hardcastle VG, Stewart RW. Supporting irrational suicide. Bioethics. 2002;16(5):425-438. doi:https://doi.org/10.1111/1467-8519.00300	CASE
240	Oneschuk D, Fainsinger R. Medical and ethical dilemmas when an advanced cancer patient discontinues dialysis. J Palliat Care. 2002;18(2):123-126. https://www.proquest.com/scholarly-journals/medical-ethical-dilemmas-when-advanced-cancer/docview/619905524/se-2	CASE
241	Woodruff R. Legalized Physician-Assisted Suicide in Oregon. J Palliat Med. 2002;5(3):445. doi:https://doi.org/10.1089/109662102320135405	EDITORIAL
242	Crichton P, Moorey S. Treating pain in cancer patients. In: Turk DC, Gatchel RJ, eds. Psychological approaches to pain management: A practitioner's handbook (2nd ed.)2nd ed. ed. The Guilford Press; 2002:501. https://www.proquest.com/books/treating-pain-cancer-patients/docview/620064041/se-2	UNFOCUSED
243	Materstvedt LJ, Kaasa S. Euthanasia and physician-assisted suicide in Scandinavia--With a conceptual suggestion regarding international research in relation to the phenomena. Palliat Med. 2002;16(1):17-32. doi:https://doi.org/10.1191/0269216302pm470oa	REVIEW
244	Tremblay A, Breitbart W. Psychiatric dimensions of palliative care. Neurol Clin. 2001;19(4):949-967. doi:https://doi.org/10.1016/S0733-8619(05)70055-4	REVIEW
245	Filiberti A, Ripamonti C, Totis A, et al. Characteristics of terminal cancer patients who committed suicide during a home palliative care program. J Pain Symptom Manage. 2001;22(1):544-553. doi:https://doi.org/10.1016/S0885-3924(01)00295-0	DUPLICATED
246	Ciaramella A, Poli P. Assessment of depression among cancer patients: The role of pain, cancer type and treatment. Psychooncology. 2001;10(2):156-165. doi:https://doi.org/10.1002/pon.505	DUPLICATED
247	Baumrucker SJ. Palliative care in amyotrophic lateral sclerosis: A new tool in the fight against an old enemy. Am J Hosp Palliat Care. 2001;18(2):81. doi:https://doi.org/10.1177/104990910101800203	UNFOCUSED
248	https://www.proquest.com/books/handbook-cultural-health-psychology/docview/619698968/se-2	UNFOCUSED
249	Koenig HG, McCullough ME, Larson DB. Handbook of religion and health. Oxford University Press; 2001	UNFOCUSED
250	Twillman RK. The role of psychologists in palliative care. J Pharm Care Pain Symptom Control. 2001;9(4):79-83. doi:https://doi.org/10.1300/J088v09n04_08	EDITORIAL
251	Lloyd-Williams M, Friedman T, Rudd N. Criterion validation of the Edinburgh Postnatal Depression Scale as a screening tool for depression in patients with advanced metastatic cancer. J Pain Symptom Manage. 2000;20(4):259-265. doi:https://doi.org/10.1016/S0885-3924(00)00182-2	DUPLICATED
252	Rosenfeld B, Breitbart W, Galietta M, et al. The Schedule of Attitudes toward Hastened Death: Measuring desire for death in terminally ill cancer patients. Cancer. 2000;88(12):2868-2875. doi:3.0.CO;2-K" TARGET="_blank">https://doi.org/10.1002/1097-0142(20000615)88:12<2868::AID-CNCR30>3.0.CO;2-K	DUPLICATED

253	Handbook of psychiatry in palliative medicine. Oxford University Press; 2000. https://www.proquest.com/books/handbook-psychiatry-palliative-medicine/docview/619681445/se-2	UNFOCUSED
254	Family guide to emotional wellness. New Harbinger Publications; 2000. https://www.proquest.com/books/family-guide-emotional-wellness/docview/619515912/se-2	UNFOCUSED
255	Ahronheim JC, Davol SB. Pursuit of assisted dying: A pilot study of inquiries made to a national consumer-based organization. J Pain Symptom Manage. 1999;18(6):401-405. doi:https://doi.org/10.1016/S0885-3924(99)00106-2	DUPLICATED
256	Chochinov HM. Psychiatric dimensions of palliative medicine. (depression, physician assisted suicide, social support, terminal illness). Order No. AAMNQ31969 ed. ; 1999. https://www.proquest.com/dissertations-theses/psychiatric-dimensions-palliative-medicine/docview/619443856/se-2	REVIEW
257	Toverud-Severson K. Dying cancer patients: Variables influencing end-of-life decisions. Order No. AAM9908043 ed. ; 1999. https://www.proquest.com/dissertations-theses/dying-cancer-patients-variables-influencing-end/docview/619443750/se-2	UNFOCUSED
258	Innovations in clinical practice: A source book, Vol. 17. Professional Resource Press/Professional Resource Exchange; 1999. https://www.proquest.com/books/innovations-clinical-practice-source-book-vol-17/docview/619395434/se-2	UNFOCUSED
259	Smith TJ, Schnipper LJ. The American Society of Clinical Oncology Program to Improve End-of-Life Care. J Palliat Med. 1998;1(3):221. doi:https://doi.org/10.1089/jpm.1998.1.221	DUPLICATED
260	McCormack P. Quality of life and the right to die: An ethical dilemma. J Adv Nurs. 1998;28(1):63-69. doi:https://doi.org/10.1046/j.1365-2648.1998.00762.x	DUPLICATED
261	Minear MD. Recurrent cancer and quality of life: A description of the experiences of oncology patients. Order No. AAM9738292 ed. ; 1998. https://www.proquest.com/dissertations-theses/recurrent-cancer-quality-life-description/docview/619374834/se-2	UNFOCUSED
262	Sullivan M, Rapp S, Fitzgibbon D, Chapman CR. Pain and the choice to hasten death in patients with painful metastatic cancer. J Palliat Care. 1997;13(3):18-28. https://www.proquest.com/scholarly-journals/pain-choice-hasten-death-patients-with-painful/docview/619219632/se-2	UNFOCUSED
263	Severson KT. Dying cancer patients: Choices at the end of life. J Pain Symptom Manage. 1997;14(2):94-98. doi:https://doi.org/10.1016/S0885-3924(97)00110-3	DUPLICATED
264	Portenoy RK. "Slow euthanasia": Reply. J Palliat Care. 1997;13(2):56. https://www.proquest.com/scholarly-journals/slow-euthanasia-reply/docview/619120962/se-2	EDITORIAL
265	Quick JC, Quick JD, Nelson DL, Hurrell JJ, Jr. Individual consequences of stress. In: Anonymous Preventive stress management in organizationsAmerican Psychological Association; 1997:65	UNFOCUSED
266	Lester D, Yang B. An approach for examining the rationality of suicide. Psychol Rep. 1996;79(2):405-406. doi:https://doi.org/10.2466/pr0.1996.79.2.405	DUPLICATED
267	Darley JM, Loeb I, Hunter J. Community attitudes on the family of issues surrounding the death of terminal patients. J Soc Iss. 1996;52(2):85-104. doi:https://doi.org/10.1111/j.1540-4560.1996.tb01569.x	UNFOCUSED
268	Cherny NI. The problem of inadequately relieved suffering. J Soc Iss. 1996;52(2):13-30. doi:https://doi.org/10.1111/j.1540-4560.1996.tb01565.x	DUPLICATED
269	Breitbart W, Rosenfeld BD, Passik SD. Interest in physician-assisted suicide among ambulatory HIV-infected patients. Am J Psychiatry. 1996;153(2):238-242. doi:https://doi.org/10.1176/ajp.153.2.238	DUPLICATED
270	Cremens MC, Calabrese LV, Shuster JL, Stern TA. The Massachusetts General Hospital Annotated Bibliography: For residents training in consultation-liaison psychiatry. Psychosomatics: Journal of Consultation and Liaison Psychiatry. 1995;36(3):217-235. doi:https://doi.org/10.1016/S0033-3182(95)71661-9	UNFOCUSED
271	Olszewski ME. The effect of religious coping on depression and anxiety in adolescence. Order No. AAM9434267 ed. ; 1995. https://www.proquest.com/dissertations-theses/effect-religious-coping-on-depression-anxiety/docview/618740745/se-2	UNFOCUSED
272	Breitbart W, Bruera E, Chochinov H, Lynch M. Neuropsychiatric syndrome and psychological symptoms in patients with advanced cancer. J Pain Symptom Manage. 1995;10(2):131-141. doi:https://doi.org/10.1016/0885-3924(94)00075-V	DUPLICATED
273	Men's health and illness: Gender, power, and the body. Sage Publications, Inc; 1995	UNFOCUSED
274	Massie MJ, Gagnon P, Holland JC. Depression and suicide in patients with cancer. J Pain Symptom Manage. 1994;9(5):325-340. doi:https://doi.org/10.1016/0885-3924(94)90192-9	DUPLICATED
275	Wise MG, Rundell JR. Concise guide to consultation psychiatry. 2nd ed. ed. American Psychiatric Association; 1994. https://www.proquest.com/books/concise-guide-consultation-psychiatry/docview/618407198/se-2	UNFOCUSED
276	Logue BJ. When hospice fails: The limits of palliative care. Omega: Journal of Death and Dying. 1994;29(4):291-301. doi:https://doi.org/10.2190/EG0J-T0G5-1LJ4-KTPW	DUPLICATED
277	Lo Presto CT, Sherman MF, DiCarlo MA. Factors affecting the unacceptability of suicide and the effects of evaluator depression and religiosity. Omega: Journal of Death and Dying. 1995;30(3):205-221. doi:https://doi.org/10.2190/XHTH-DH29-PHTD-4WVM	UNFOCUSED

278	MacDonald SM, Sandmaier R, Fainsinger RL. Objective evaluation of spiritual care: A case report. J Palliat Care. 1993;9(2):47-49. https://www.proquest.com/scholarly-journals/objective-evaluation-spiritual-care-case-report/docview/618417437/se-2	CASE
279	Breitbart W. Suicide risk and pain in cancer and AIDS patients. In: Chapman CR, Foley KM, eds. Current and emerging issues in cancer pain: Research and practiceRaven Press; 1993:49-65, 441 Pages. https://www.proquest.com/books/suicide-risk-pain-cancer-aids-patients/docview/618402207/se-2	UNFOCUSED
280	Innovations in clinical practice: A source book, Vol. 12. Professional Resource Press/Professional Resource Exchange; 1993. https://www.proquest.com/books/innovations-clinical-practice-source-book-vol-12/docview/618551800/se-2	UNFOCUSED
281	Current and emerging issues in cancer pain: Research and practice. Raven Press; 1993. https://www.proquest.com/books/current-emerging-issues-cancer-pain-research/docview/618400717/se-2	UNFOCUSED
282	Death, dying & bereavement. Sage Publications, Inc Open University Press; 1993. https://www.proquest.com/books/death-dying-amp-bereavement/docview/618396767/se-2	UNFOCUSED
283	Neuroscience year: Supplement 3 to the "Encyclopedia of Neuroscience.". Birkhäuser; 1993. https://www.proquest.com/books/neuroscience-year-supplement-3-encyclopedia/docview/618408613/se-2	UNFOCUSED
284	Hammond LK, Deluty RH. Attitudes of clinical psychologists, psychiatrists, and oncologists toward suicide. Social Behavior and Personality: An International Journal. 1992;20(4):289. doi:https://doi.org/10.2224/sbp.1992.20.4.289	UNFOCUSED
285	Lester D. Suicide and disease. Loss, Grief & Care. 1992;6(2-3):173-181. https://www.proquest.com/scholarly-journals/suicide-disease/docview/618306974/se-2	UNFOCUSED
286	Coyle N, Adelhardt J, Foley KM, Portenoy RK. "Character of terminal illness in the advanced cancer patient: Pain and other symptoms during the last four weeks of life": Response. J Pain Symptom Manage. 1991;6(7):409-410. doi:https://doi.org/10.1016/0885-3924(91)90038-6	EDITORIAL
287	Glare PA, Krech RL, Walsh TD. "Character of terminal illness in the advanced cancer patient: Pain and other symptoms during the last four weeks of life": Comment. J Pain Symptom Manage. 1991;6(7):408-409. doi:https://doi.org/10.1016/0885-3924(91)90037-5	EDITORIAL
288	Foley KM. The relationship of pain and symptom management to patient requests for physician-assisted suicide. J Pain Symptom Manage. 1991;6(5):289-297. doi:https://doi.org/10.1016/0885-3924(91)90052-6	DUPLICATED
289	Teising M. Psychosomatic illness in aging patients in a psychiatric consultation service of a {German} general hospital. Psychologie Medicale. 1991;23(7):807-910. https://www.proquest.com/scholarly-journals/psychosomatic-illness-aging-patients-psychiatric/docview/619236509/se-2	UNFOCUSED
290	Mermelstein HT, Ostroff JS, Massie MJ. Cancer in the elderly: Psychiatric issues and treatment. In: Myers WA, ed. New techniques in the psychotherapy of older patientsAmerican Psychiatric Association; 1991:143-169, Chapter xix, 290 Pages. https://www.proquest.com/books/cancer-elderly-psychiatric-issues-treatment/docview/618052363/se-2	UNFOCUSED
291	Child and adolescent psychiatry: A comprehensive textbook. Williams & Wilkins Co; 1991. https://www.proquest.com/books/child-adolescent-psychiatry-comprehensive/docview/618047739/se-2	UNFOCUSED
292	Martin SK, Range LM. Extenuating circumstances in perceptions of suicide: Disease diagnosis (AIDS, cancer), pain level, and life expectancy. Omega: Journal of Death and Dying. 1991;22(3):187-197. doi:https://doi.org/10.2190/CX7Q-LQ1Y-BG6K-8XKF	UNFOCUSED
293	Kopp M, Skrabski A. What does the legacy of Hans Selye and Franz Alexander mean today? The psychophysiological approach in medical practice. International Journal of Psychophysiology. 1989;8(2):99-105. doi:https://doi.org/10.1016/0167-8760(89)90001-9	UNFOCUSED
294	Handbook of psychooncology: Psychological care of the patient with cancer. Oxford University Press; 1989. https://www.proquest.com/books/handbook-psychooncology-psychological-care/docview/617687395/se-2	UNFOCUSED
295	Raimbault E, Montange F, Pichard E, Poulain P. Cancers et souffrances rebelles: Aspects psychologiques. Psychologie Medicale. 1988;20(9):1369-1372. https://www.proquest.com/scholarly-journals/cancers-et-souffrances-rebelles-aspects/docview/617842475/se-2	UNFOCUSED
296	Kontaxakis VP, Christodoulou GN, Mavreas VG, Havaki-Kontaxaki B. Attempted suicide in psychiatric outpatients with concurrent physical illness. Psychother Psychosom. 1988;50(4):201-206. doi:https://doi.org/10.1159/000288121	UNFOCUSED
297	Deluty RH. Factors affecting the acceptability of suicide. Omega: Journal of Death and Dying. 1989;19(4):315-326. doi:https://doi.org/10.2190/YX4X-YJBG-45WV-8VW0	UNFOCUSED
298	Deluty RH. Physical illness, psychiatric illness, and the acceptability of suicide. Omega: Journal of Death and Dying. 1989;19(1):79-91. doi:https://doi.org/10.2190/DUHX-5HQ0-4L3X-F96J	UNFOCUSED
299	Kumar L. Psychological supportive care in cancer patients: A review. Indian Journal of Clinical Psychology. 1987;14(1):52-55. https://www.proquest.com/scholarly-journals/psychological-supportive-care-cancer-patients/docview/617624016/se-2	REVIEW
300	Principles of medical psychiatry. Grune & Stratton, Inc/Harcourt, Bra; 1987. https://www.proquest.com/books/principles-medical-psychiatry/docview/617478236/se-2	UNFOCUSED

301	Simpson MA. Dying, death and grief: A critical bibliography. University of Pittsburgh Press; 1987. https://www.proquest.com/books/dying-death-grief-critical-bibliography/docview/617475704/se-2	REVIEW
302	Family-centered medical care: A clinical casebook. Guilford Press; 1987. https://www.proquest.com/books/family-centered-medical-care-clinical-casebook/docview/617356778/se-2	UNFOCUSED
303	Esposito JL. The obsolete self: Philosophical dimensions of aging. University of California Press; 1987. https://www.proquest.com/books/obsolete-self-philosophical-dimensions-aging/docview/617351292/se-2	UNFOCUSED
304	Mackenzie TB, Popkin MK. Suicide in the medical patient. Int J Psychiatry Med. 1987;17(1):3-22. doi:https://doi.org/10.2190/EF4F-9KV7-1MLM-MYXQ	UNFOCUSED
305	Siegel K. Psychosocial aspects of rational suicide. Am J Psychother. 1986;40(3):405-418. https://www.proquest.com/scholarly-journals/psychosocial-aspects-rational-suicide/docview/617215768/se-2	UNFOCUSED
306	Stillion JM. The demise of dualism: Toward a convergence of brain research and therapy. Death Stud. 1986;10(4):313-329. doi:https://doi.org/10.1080/07481188608252830	REVIEW
307	Siegel K, Tuckel P. Rational suicide and the terminally ill cancer patient. Omega: Journal of Death and Dying. 1985;15(3):263-269. doi:https://doi.org/10.2190/7NCK-Q22N-5Y21-49BM	UNFOCUSED
308	Mash EJ, Kaplan BJ. Behavioral auscultation. Contemporary Psychology. 1983;28(7):546. doi:https://doi.org/10.1037/022173	REVIEW
309	Koenig R. Dying vs. well-being. Omega: Journal of Death and Dying. 1973;4(3):181-194. doi:https://doi.org/10.2190/E1X8-TXQL-QABW-TVH4	DUPLICATED
310	Laxenaire M, Bentz L, Chardot C. A psychological approach to the cancer patient: Observations on 80 cases. Annales Médico-Psychologiques. 1972;1(2):195-207. https://www.proquest.com/scholarly-journals/psychological-approach-cancer-patient/docview/615871419/se-2	UNFOCUSED
311	Tabachnick N. Theories of self-destruction. The American Journal of Psychoanalysis. 1972;32(1):53-61. doi:https://doi.org/10.1007/BF01872484	DUPLICATED
312	Nick J, al e. THE SUICIDE OF ABANDON. Annales Médico-Psychologiques. 1966;2(1):5-17. https://www.proquest.com/scholarly-journals/suicide-abandon/docview/615496464/se-2	NOT ENGLISH
313	Baonville H, Ley J, Titeca J. Un cas de tétanie post-opératoire avec troubles mentaux graves. Annales Médico-Psychologiques. 1934;92:26-40. https://www.proquest.com/scholarly-journals/un-cas-de-tétanie-post-opératoire-avec-troubles/docview/615025654/se-2	CASE
314	Clouston TS. Lecture III. States of mental depression—Melancholia (psychalgia)—Continued. In: Anonymous Clinical lectures on mental diseasesHenry C Lea's Son & Co; 1884:90	UNFOCUSED

Table S3. PRISMA 2020 checklist (1).

Section and Topic	Item #	Checklist item	Location where item is reported
**TITLE**			
Title	1	Identify the report as a systematic review.	Pag. 1
**ABSTRACT**			
Abstract	2	See the PRISMA 2020 for Abstracts checklist.	Pag. 1
**INTRODUCTION**			
Rationale	3	Describe the rationale for the review in the context of existing knowledge.	Pag. 3
Objectives	4	Provide an explicit statement of the objective(s) or question(s) the review addresses.	Pag. 2-3
**METHODS**			
Eligibility criteria	5	Specify the inclusion and exclusion criteria for the review and how studies were grouped for the syntheses.	Pag. 3
Information sources	6	Specify all databases, registers, websites, organisations, reference lists and other sources searched or consulted to identify studies. Specify the date when each source was last searched or consulted.	Pag. 3
Search strategy	7	Present the full search strategies for all databases, registers and websites, including any filters and limits used.	Pag. 3
Selection process	8	Specify the methods used to decide whether a study met the inclusion criteria of the review, including how many reviewers screened each record and each report retrieved, whether they worked independently, and if applicable, details of automation tools used in the process.	Pag. 3
Data collection process	9	Specify the methods used to collect data from reports, including how many reviewers collected data from each report, whether they worked independently, any processes for obtaining or confirming data from study investigators, and if applicable, details of automation tools used in the process.	Pag. 4

Section and Topic	Item #	Checklist item	Location where item is reported
Data items	10a	List and define all outcomes for which data were sought. Specify whether all results that were compatible with each outcome domain in each study were sought (e.g. for all measures, time points, analyses), and if not, the methods used to decide which results to collect.	Pag. 4
	10b	List and define all other variables for which data were sought (e.g. participant and intervention characteristics, funding sources). Describe any assumptions made about any missing or unclear information.	Pag. 3-4
Study risk of bias assessment	11	Specify the methods used to assess risk of bias in the included studies, including details of the tool(s) used, how many reviewers assessed each study and whether they worked independently, and if applicable, details of automation tools used in the process.	Pag. 4
Effect measures	12	Specify for each outcome the effect measure(s) (e.g. risk ratio, mean difference) used in the synthesis or presentation of results.	Pag. 4
Synthesis methods	13a	Describe the processes used to decide which studies were eligible for each synthesis (e.g. tabulating the study intervention characteristics and comparing against the planned groups for each synthesis (item #5)).	Pag. 3-4
	13b	Describe any methods required to prepare the data for presentation or synthesis, such as handling of missing summary statistics, or data conversions.	Pag. 3-4
	13c	Describe any methods used to tabulate or visually display results of individual studies and syntheses.	Pag. 3-4
	13d	Describe any methods used to synthesize results and provide a rationale for the choice(s). If meta-analysis was performed, describe the model(s), method(s) to identify the presence and extent of statistical heterogeneity, and software package(s) used.	Pag. 4
	13e	Describe any methods used to explore possible causes of heterogeneity among study results (e.g. subgroup analysis, meta-regression).	Pag. 4
	13f	Describe any sensitivity analyses conducted to assess robustness of the synthesized results.	Pag. 4
Reporting bias assessment	14	Describe any methods used to assess risk of bias due to missing results in a synthesis (arising from reporting biases).	Pag. 4
Certainty assessment	15	Describe any methods used to assess certainty (or confidence) in the body of evidence for an outcome.	Pag 4
**RESULTS**			
Study selection	16a	Describe the results of the search and selection process, from the number of records identified in the search to the number of studies included in the review, ideally using a flow diagram.	Pag. 6
	16b	Cite studies that might appear to meet the inclusion criteria, but which were excluded, and explain why they were excluded.	Suppl.
Study characteristics	17	Cite each included study and present its characteristics.	Pag. 6-10
Risk of bias in studies	18	Present assessments of risk of bias for each included study.	Suppl
Results of individual studies	19	For all outcomes, present, for each study: (a) summary statistics for each group (where appropriate) and (b) an effect estimate and its precision (e.g. confidence/credible interval), ideally using structured tables or plots.	Pag. 11
Results of syntheses	20a	For each synthesis, briefly summarise the characteristics and risk of bias among contributing studies.	Pag. Suppl
	20b	Present results of all statistical syntheses conducted. If meta-analysis was done, present for each the summary estimate and its precision (e.g. confidence/credible interval) and measures of statistical heterogeneity. If comparing groups, describe the direction of the effect.	Pag. 14
	20c	Present results of all investigations of possible causes of heterogeneity among study results.	Pag. 14
	20d	Present results of all sensitivity analyses conducted to assess the robustness of the synthesized results.	Pag. 14
Reporting biases	21	Present assessments of risk of bias due to missing results (arising from reporting biases) for each synthesis assessed.	Pag. Suppl.
Certainty of evidence	22	Present assessments of certainty (or confidence) in the body of evidence for each outcome assessed.	Suppl.
**DISCUSSION**			
Discussion	23a	Provide a general interpretation of the results in the context of other evidence.	Pag. 11-14
	23b	Discuss any limitations of the evidence included in the review.	Pag. 14
	23c	Discuss any limitations of the review processes used.	Pag. 14
	23d	Discuss implications of the results for practice, policy, and future research.	Pag. 14
**OTHER INFORMATION**			
Registration and protocol	24a	Provide registration information for the review, including register name and registration number, or state that the review was not registered.	Pag. 4
	24b	Indicate where the review protocol can be accessed, or state that a protocol was not prepared.	Pag. 4

Section and Topic	Item #	Checklist item	Location where item is reported
	24c	Describe and explain any amendments to information provided at registration or in the protocol.	Pag. 4
Support	25	Describe sources of financial or non-financial support for the review, and the role of the funders or sponsors in the review.	Pag. 14
Competing interests	26	Declare any competing interests of review authors.	Pag. 14
Availability of data, code and other materials	27	Report which of the following are publicly available and where they can be found: template data collection forms; data extracted from included studies; data used for all analyses; analytic code; any other materials used in the review.	Pag. 14

Table S4. Risk of Bias according to the Cochrane ROBINS-E tool.

Author(s), year	Confounding	Measurement Of Exposure	Selection Of Participants	Post-Exposure Interventions	Missing Data	Mesurement Of The Outcome	Selection Of Reported Results	Overall Risk Of Bias
Ciaramella A et al., 2001 [34]	SOME CONCERNS	LOW	HIGH	SOME CONCERNS	LOW	SOME CONCERNS	LOW	HIGH
Akechi T et al., 2002 [35]	SOME CONCERNS	LOW	HIGH	SOME CONCERNS	HIGH	SOME CONCERNS	LOW	HIGH
Latha KS et al., 2005 [36]	HIGH	LOW	SOME CONCERNS	SOME CONCERNS	LOW	LOW	LOW	SOME CONCERNS
Walker J et al., 2008 [37]	SOME CONCERNS	LOW	SOME CONCERNS	LOW	SOME CONCERNS	SOME CONCERNS	LOW	SOME CONCERNS
Nuhu FT et al.,. 2009 [38]	SOME CONCERNS	LOW	SOME CONCERNS	LOW	LOW	SOME CONCERNS	LOW	SOME CONCERNS
Recklitis CJ et al., 2010 [39]	LOW	LOW	SOME CONCERNS	LOW	SOME CONCERNS	SOME CONCERNS	LOW	LOW
Maneeton B et al., 2012 [40]	SOME CONCERNS	LOW	HIGH	LOW	LOW	SOME CONCERNS	LOW	HIGH
Han DH et al., 2018 [41]	LOW	LOW	LOW	LOW	SOME CONCERNS	SOME CONCERNS	LOW	LOW
Recklitis CJ et al., 2014 [42]	LOW	LOW	SOME CONCERNS	LOW	LOW	SOME CONCERNS	LOW	LOW
Park SA et al., 2016 [43]	SOME CONCERNS	LOW	SOME CONCERNS	LOW	LOW	LOW	LOW	LOW
Johnson CC et al., 2020 [44]	SOME CONCERNS	LOW	SOME CONCERNS	LOW	LOW	SOME CONCERNS	LOW	SOME CONCERNS
Zhang Y et al., 2020 [45]	SOME CONCERNS	LOW	SOME CONCERNS	LOW	LOW	LOW	LOW	LOW
Nugent SM et al., 2021 [46]	LOW	LOW	LOW	LOW	LOW	LOW	LOW	LOW
Tuan NV et al., 2024 [47]	SOME CONCERNS	LOW	SOME CONCERNS	LOW	LOW	LOW	LOW	LOW
Espuig A et al., 2024 [48]	SOME CONCERNS	LOW	SOME CONCERNS	LOW	LOW	LOW	LOW	LOW
